# Supplementary material for: Aortic disease in Marfan syndrome is caused by overactivation of sGC-PRKG signaling by NO
Source: Nat Commun. 2021 May 11;12:2628. doi: 10.1038/s41467-021-22933-3 (PMC8113458; doi:10.1038/s41467-021-22933-3)
Supplement: Supplementary file 1 — Supplementary Information [file 41467_2021_22933_MOESM1_ESM.pdf]

Aortic disease in Marfan syndrome is caused by overactivation of sGC-PRKG signaling by NO

[1,2,*]Andrea de la Fuente-Alonso, [1,2,*]Marta Toral, [1,2,3]Álvaro Alfayate, [1,2]María Jesús Ruiz-Rodríguez, [2,3]Elena Bonzón-Kulichenko, [2,4]Gisela Teixido-Tura, [1,2]Sara Martínez-Martínez, [1,2]María José Méndez-Olivares, [1,2]Dolores López-Maderuelo, [3]Ileana González-Valdés, [5]Eusebio Garcia-Izquierdo, [5]Susana Mingo, [6]Carlos E. Martín, [7]Laura Muiño-Mosquera, [7]Julie De Backer, [2,8]J. Francisco Nistal, [6]Alberto Forteza, [2,4]Arturo Evangelista, [2,3]Jesús Vázquez, [2,9,10,#,†]Miguel R. Campanero, and [1,2,#,†]Juan Miguel Redondo.

[1]Gene regulation in cardiovascular remodeling and inflammation group, Centro Nacional de Investigaciones Cardiovasculares (CNIC), Madrid 28029, Spain. [2]Centro de Investigación Biomédica en Red de Enfermedades Cardiovasculares (CIBERCV), Spain. [3]Cardiovascular Proteomics Laboratoy, CNIC, Madrid 28029, Spain. [4]Servei de Cardiologia, Hospital Vall d'Hebron, Barcelona 08035, Spain. [5]Cardiology Department, Hospital Universitario Puerta de Hierro, Madrid 28222, Spain. [6]Cardiac Surgery Department, Hospital Universitario Puerta de Hierro, Madrid 28222, Spain. [7]Center for Medical Genetics Ghent, Ghent University Hospital, Ghent 9000, Belgium. [8]Cardiovascular Surgery and Department of Physiology and Pharmacology, Hospital Universitario Marqués de Valdecilla, IDIVAL, Facultad de Medicina, Universidad de Cantabria, Santander 39005, Spain. [9]Department of Cancer Biology, Instituto de Investigaciones Biomedicas Alberto Sols, Consejo Superior de Investigaciones Científicas–Universidad Autónoma de Madrid, Madrid 28029, Spain. [10]Present address: Centro de Biología Molecular Severo Ochoa, Consejo Superior de Investigaciones Científicas–Universidad Autónoma de Madrid, Madrid 28049, Spain. [*]These authors contributed equally to this work. [#]These authors jointly supervised this work [†]Correspondence should be addressed to M.R.C. (mcampanero@cbm.csic.es) or J.M.R. (jmredondo@cnic.es).

Supplementary information

Table of contents

Item	Pages
Supplementary Figure 1	3
Supplementary Figure 2	4
Supplementary Figure 3	5-6
Supplementary Figure 4	7
Supplementary Figure 5	8
Supplementary Figure 6	9
Supplementary Figure 7	10
Supplementary Figure 8	11
Supplementary Figure 9	12
Supplementary Figure 10	13-14
Supplementary Figure 11	15
Supplementary Figure 12	16-17
Supplementary Figure 13	18
Supplementary Figure 14	19
Supplementary Table 1	20

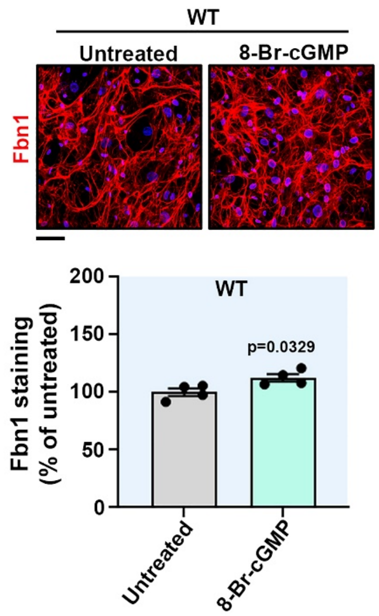

Supplementary Figure 1. **PRKGI activation does not decrease fibrillin-1 fiber formation in WT VSMCs.** Representative images of fibrillin-1 (red) and DAPI-stained nuclei (blue) in WT VSMCs untreated or treated with 8-Br-c GMP for 7 days (4 independent cell batches per condition). Scale bar, 50 µm. The graph shows Fbn1 quantification relative to untreated WT cells as mean ± s.e.m. Each data point denotes the mean of an independent VSMC experiment. Differences were analyzed by unpaired two-tailed Student's t-test (p-value is shown). Source data are provided in the Source Data file.

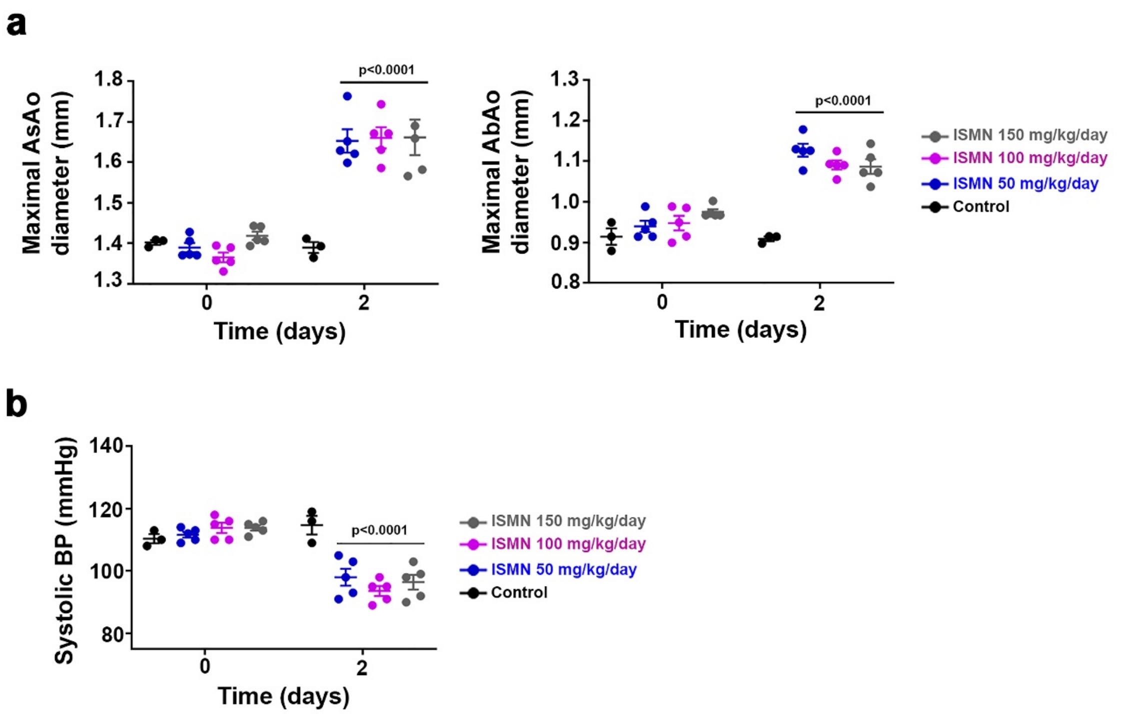

Supplementary Figure 2. **ISMN doses greater than 50mg/kg/day do not increase the effect on BP and aortic dilation.** Twelve-week-old C57BL/6 mice were treated for 2 days (d) with the indicated ISMN doses by osmotic minipump infusion (n=5 per treated group; n=3 for the Control group). (**a)** Maximal AsAo (left) and AbAo (right) diameter before treatment (0) and at 2 d post-treatment. (**b**) Systolic BP before and after treatment. Data are shown as mean ± s.e.m. Each data point denotes an individual mouse. Differences versus Control mice were analyzed by repeated-measurements two-way ANOVA at each time point followed by Tukey's post hoc test (p-values are shown). Source data are provided in the Source Data file.

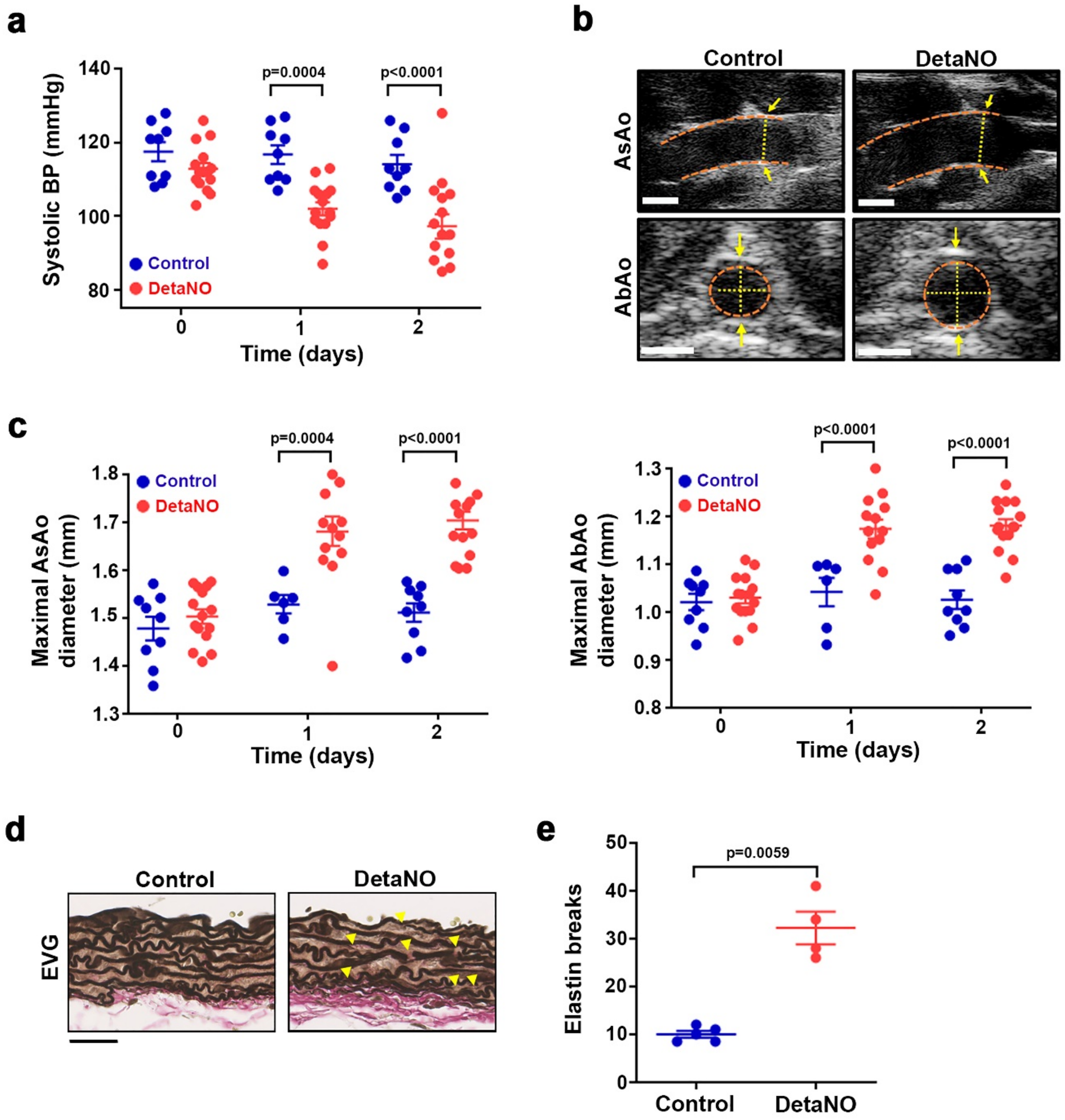

Supplementary Figure 3. DetaNO triggers aortic dilation, decreases blood pressure and induces medial degeneration in WT mice. Twelve-week-old C57BL/6 mice were treated for 2 d with 5mg/kg/day DetaNO by osmotic minipump infusion. (**a**) Systolic BP. (**b**) Representative end-of-experiment ultrasound images of AsAo and AbAo. Orange dashed lines delineate the lumen boundary, and yellow dashed lines mark the lumen diameter. Scale bar, 1 mm. (**c**) Maximal AsAo (left) and AbAo (right) diameter. (a-c) *n=9* Control mice; *n=15* DetaNO-treated mice pooled from 2 independent experiments. Data from before treatment, 1 d and 2 d post-treatment are shown as mean ± s.e.m. Each data point denotes an individual mouse. Differences versus Control mice were analyzed by repeated-measurements two-way ANOVA at each time point followed by Sidak's post hoc test (p-values are shown). **(d)** Representative

images showing elastic van Gieson (EVG) staining in AsAo sections from Control and DetaNO-treated mice. Yellow arrowheads indicate elastin breaks. Scale bar, 50 μm. (**e**) Quantification of elastin breaks in AsAo sections from 5 Control mice and 4 DetaNO-treated mice. Data are mean ± s.e.m. Each data point denotes an individual mouse. Differences were analyzed by unpaired two-tailed t-test with Welch's correction (p-value is shown). Source data are provided in the Source Data file.

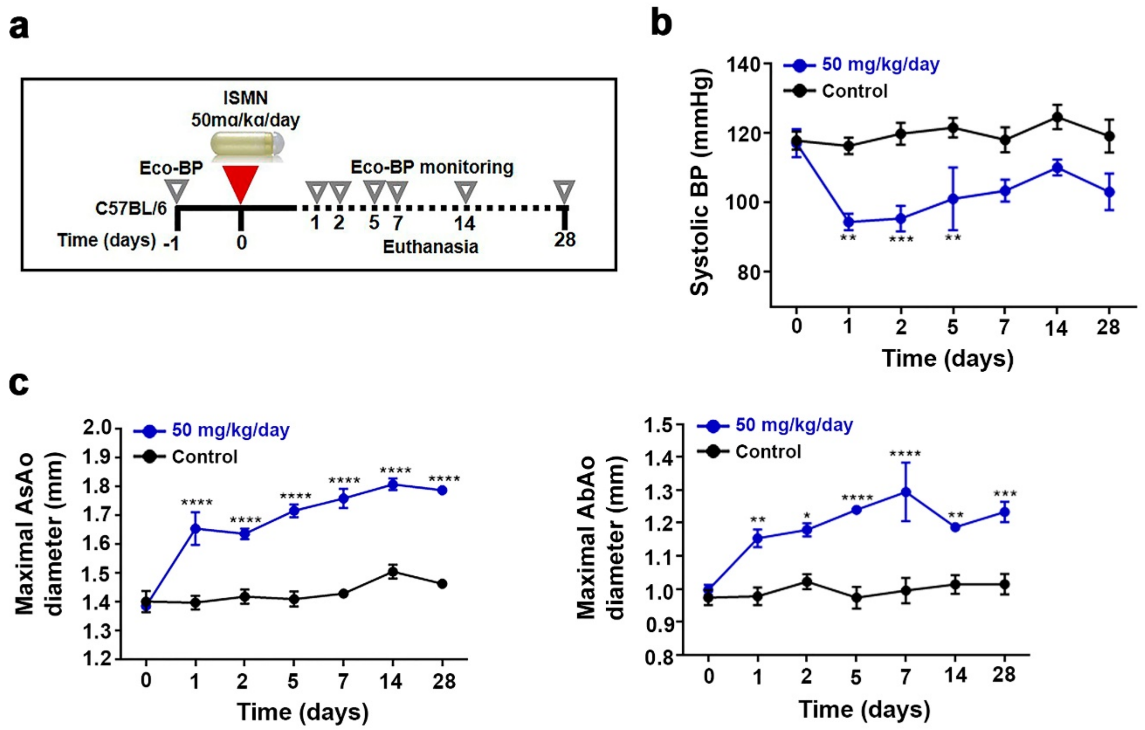

Supplementary Figure 4. Long-term ISMN administration results in sustained aortic dilation. (**a**) Experimental design. 12-week-old C57BL/6 mice were treated with the NO-donor ISMN (50 mg/kg/day) via osmotic minipump infusion. Ultrasound and BP analysis (Eco-BP) was performed at the indicated times (empty triangles). (**b**) Systolic BP and (**c**) Maximal AsAo (left) and AbAo (right) diameter. Data are shown as mean ± s.e.m. (*n*=3 control WT mice and *n*=4 ISMN-treated WT mice in b and c). Each data point denotes an individual mouse. **P*<0.05, ***P*<0.01, ****P*<0.001 and *****P*<0.0001 versus Control mice at each time point by repeated-measurements two-way ANOVA followed by Sidak's post hoc test. Source data are provided in the Source Data file.

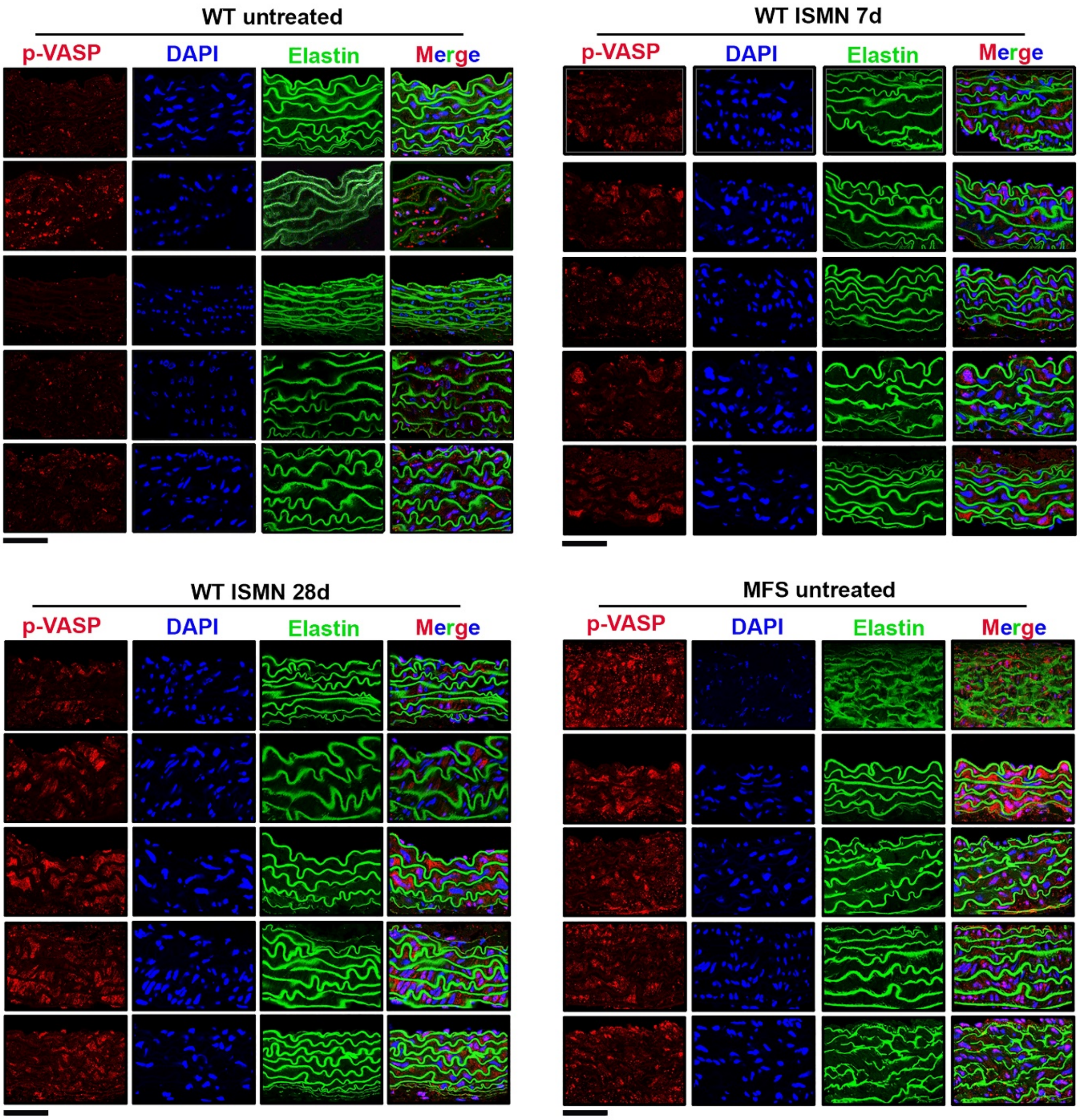

Supplementary Figure 5. Aortic staining of pVASP-S239 in MFS mice and ISMN-treated WT mice. Representative images of pVASP-S239 immunofluorescence (red), elastin autofluorescence (green), and DAPI-stained nuclei (blue) in aortic sections from untreated MFS and WT mice and WT mice treated with 50mg/kg/da ISMN for 7 d and 28 d (*n*=5 mice per group). Scale bar, 50 µm.

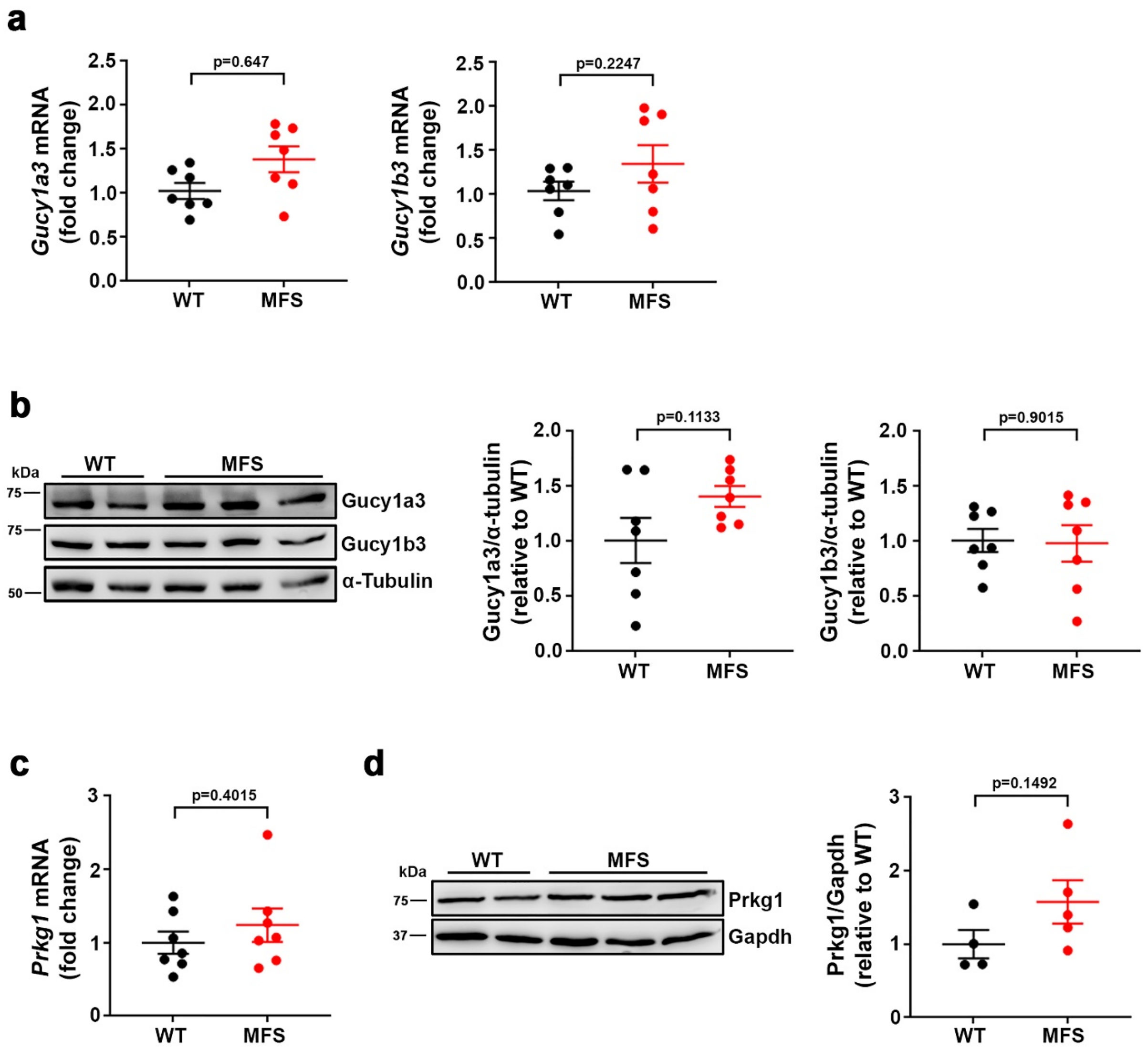

Supplementary Figure 6. Prkg1 and sGC expression is not altered in the aortas of MFS mice. (a) RT-qPCR analysis of *Gucy1a3* and *Gucy1b3* mRNA expression in the aortas of 7 WT and 7 MFS mice. (**b**) Representative Gucy1a3, Gucy1b3 and α-Tubulin (loading control) immunoblot analysis of aortic extracts from 7 WT and 7 MFS mice and quantification of immunoblot signals. **(c)** RT-qPCR analysis of *Prkg1* mRNA expression in the aortas of 7 WT and 7 MFS mice. (**d**) Representative Prkg1 and Gapdh (loading control) immunoblot analysis of aortic extracts from 4 WT and 5 MFS mice. Uncropped blots in Supplementary Figure 13 (a,c) mRNA amounts were normalized to *Gapdh* expression and are shown as mean ± s.e.m. (b,d) Data are shown as mean ± s.e.m. and each data point denotes an individual mouse Differences were analyzed by unpaired two-tailed t-test with Welch's correction (p-values are shown). Source data are provided in the Source Data file.

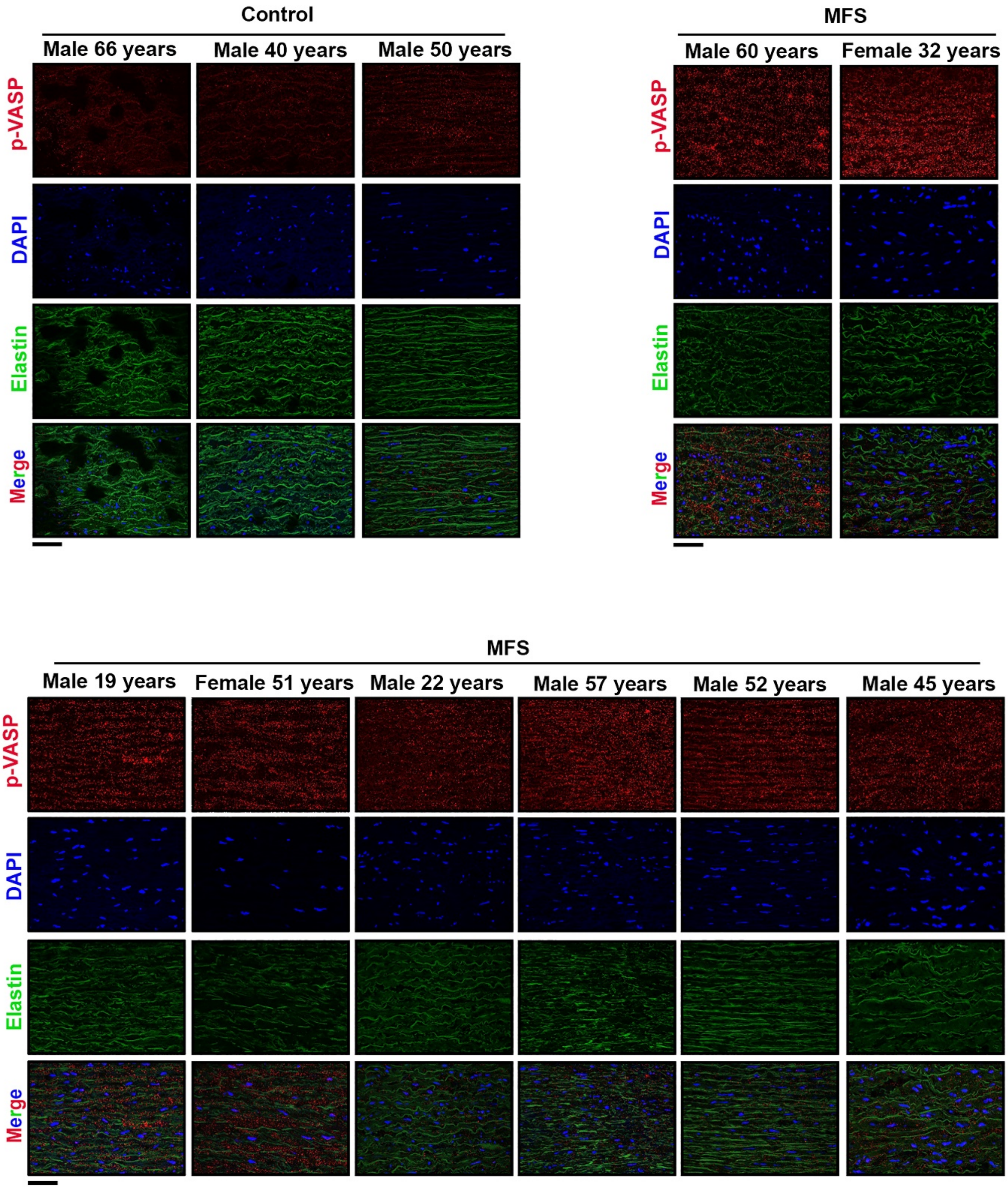

Supplementary Figure 7. VASP-Ser239 phosphorylation is upregulated in aortas of MFS patients. Representative images of pVASP-S239 immunofluorescence (red), elastin autofluorescence (green), and DAPI-stained nuclei (blue) in the medial layer of aortic sections from 3 control donors and 8 MFS patients (*n*=3 independent experiments). Scale bar, 50 μm.

a

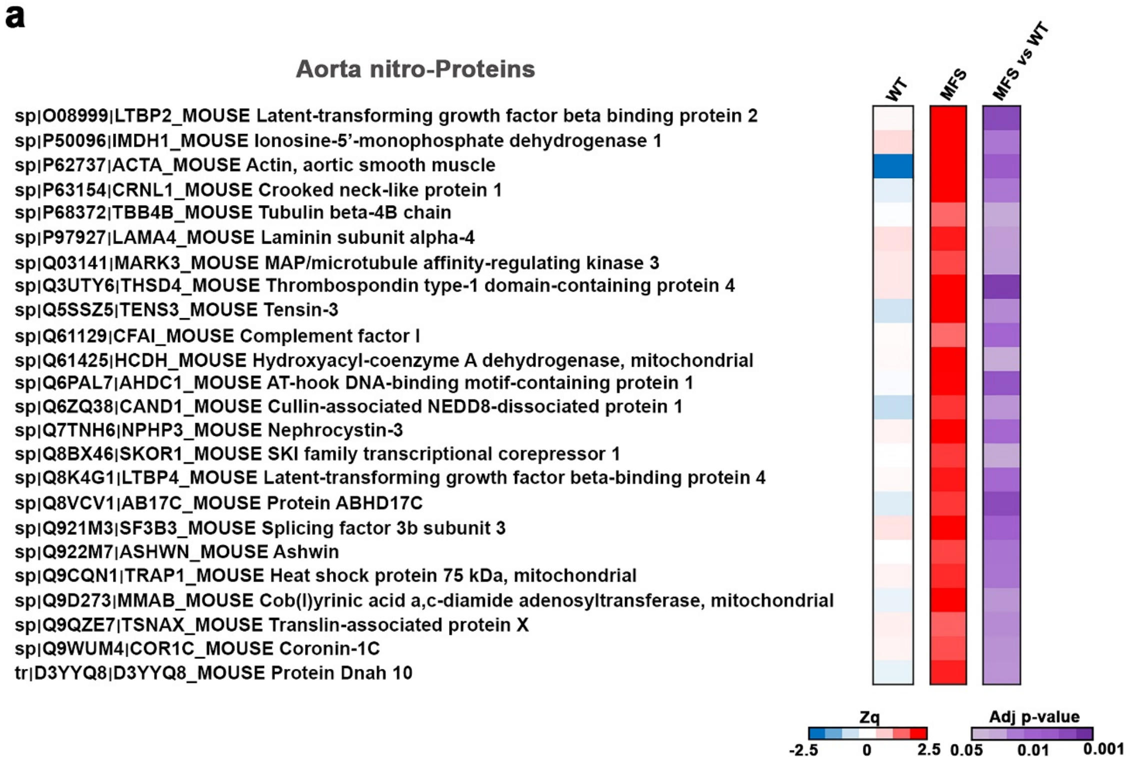

Supplementary Figure 8. MFS aortic tissue proteins showing a significant increase in nitration. Heat-map including all nitrated proteins from WT and MFS AsAo tissue which show significant up-regulation of nitration. The heat-map shows standardized log2ratios (Zq) of nitro-protein values and statistical significance calculated by limma analysis.

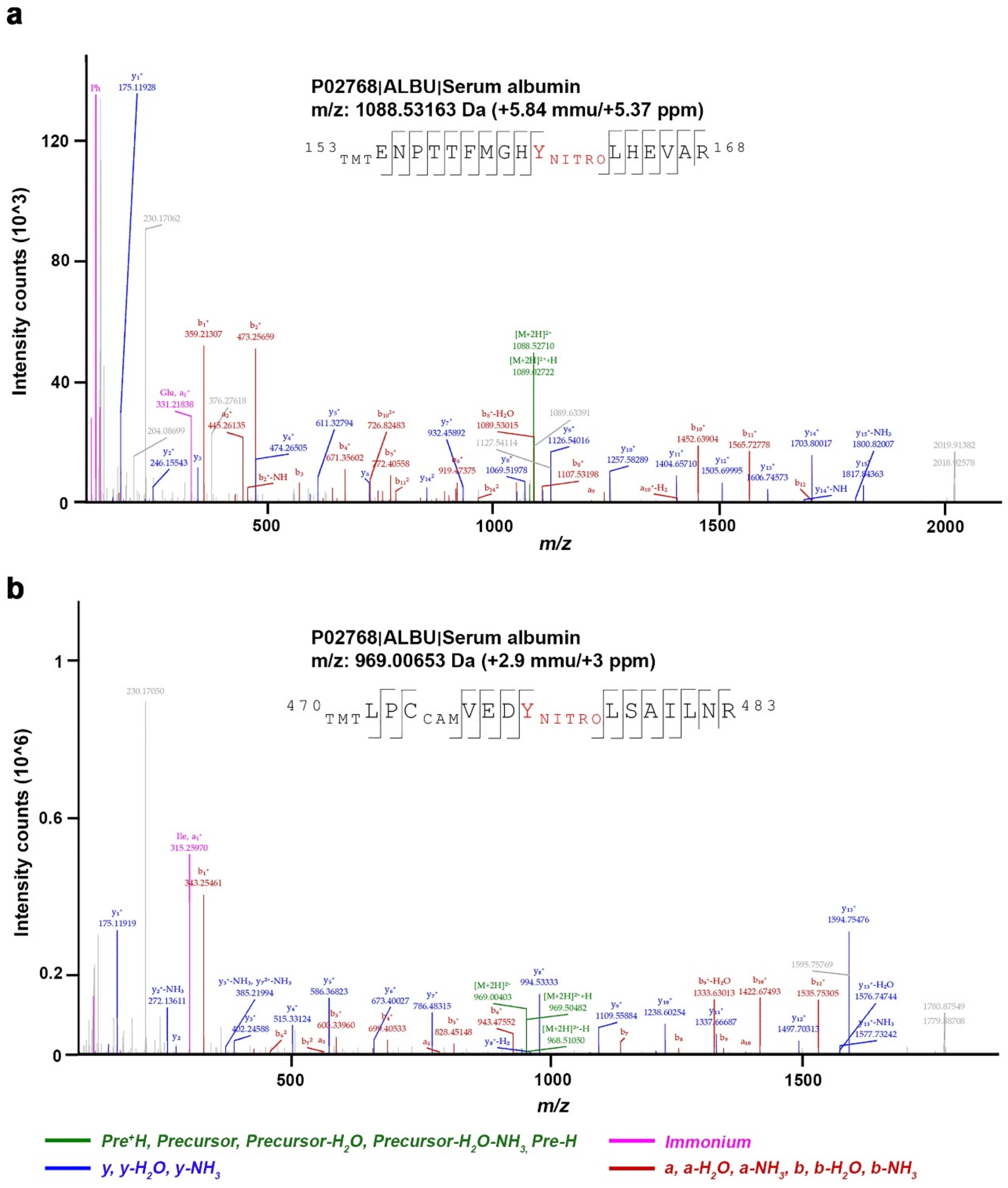

Supplementary Figure 9. Identification of nitrated peptides in mouse plasma. Annotated MS/MS spectra of 2 representative nitro-peptides **(a, b).** Identified b-ions, y-ions, and precursors are marked. TMT, TMT6plex, 229.16293 Da; nitro, 44.98508 Da; CAM, carbamidomethyl, 57.021464 Da.

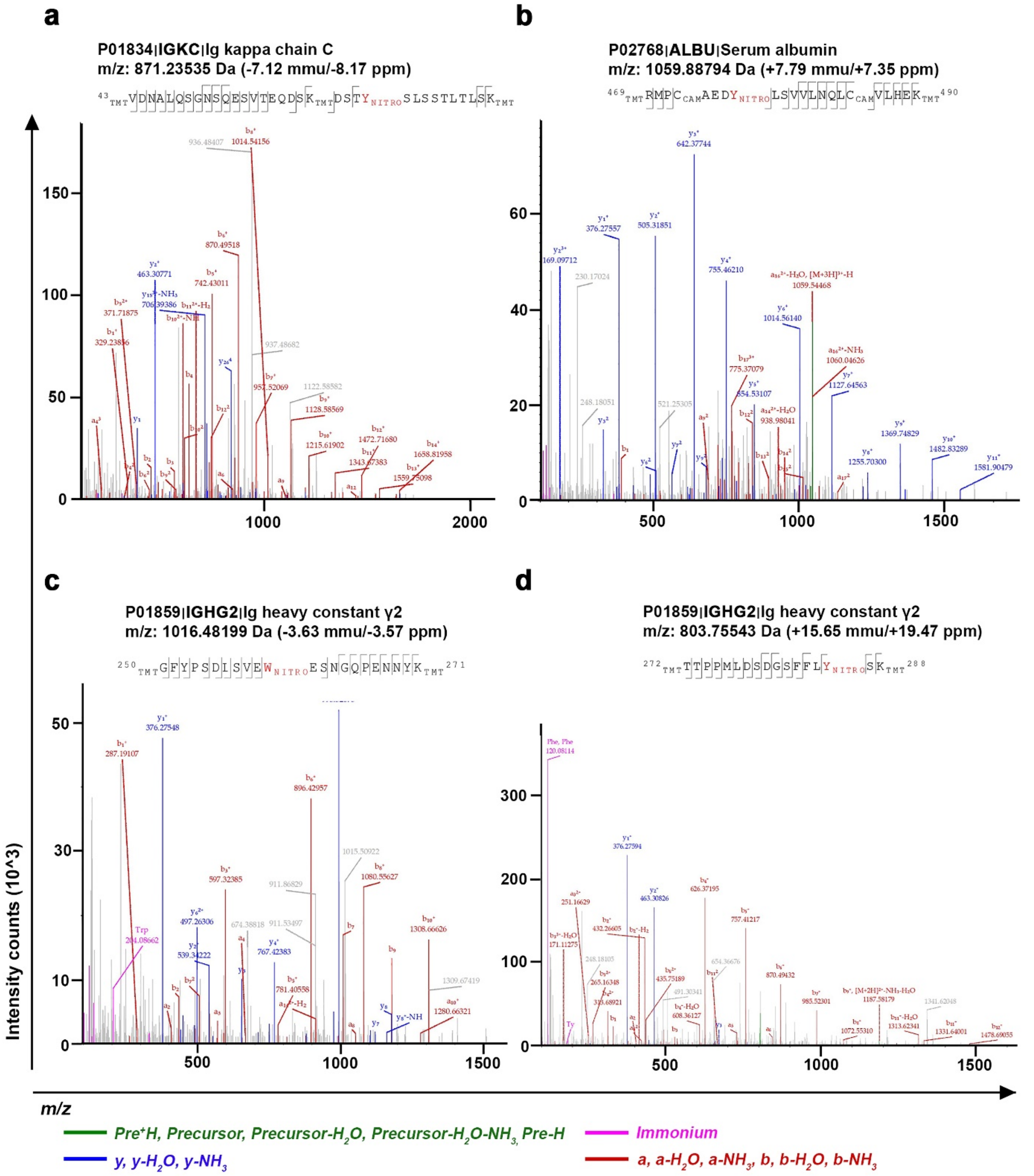
a
P01834|IGKC|Ig kappa chain C
m/z: 871.23535 Da (-7.12 mmu/-8.17 ppm)
b
P02768|ALBU|Serum albumin
m/z: 1059.88794 Da (+7.79 mmu/+7.35 ppm)
c
P01859|IGHG2|Ig heavy constant γ2
m/z: 1016.48199 Da (-3.63 mmu/-3.57 ppm)
d
P01859|IGHG2|Ig heavy constant γ2
m/z: 803.75543 Da (+15.65 mmu/+19.47 ppm)
Intensity counts (10^3)
m/z
Pre+H, Precursor, Precursor-H2O, Precursor-H2O-NH3, Pre-H
Immonium
y, y-H2O, y-NH3
a, a-H2O, a-NH3, b, b-H2O, b-NH3

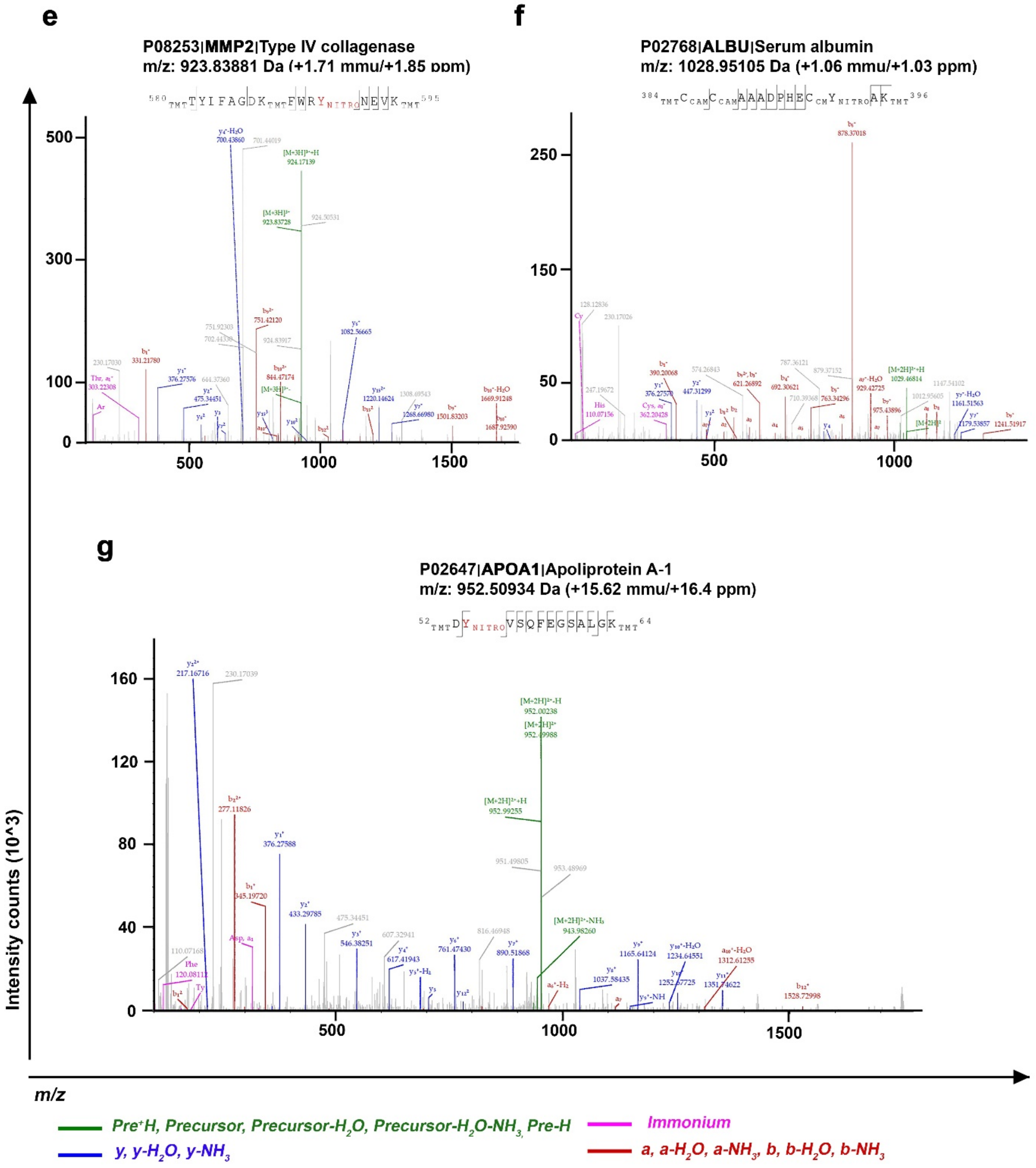

Supplementary Figure 10. Identification of nitrated peptides in human plasma. Annotated MS/MS spectra of the 7 nitro-peptides shown in Figure 6C (**a-g**). Identified b-ions, y-ions, and precursors are marked. TMT, TMT6plex, 229.16293 Da; nitro, 44.98508 Da; CAM: carbamidomethyl, 57.021464 Da; CM, carboxymethyl, 58.005479 Da.

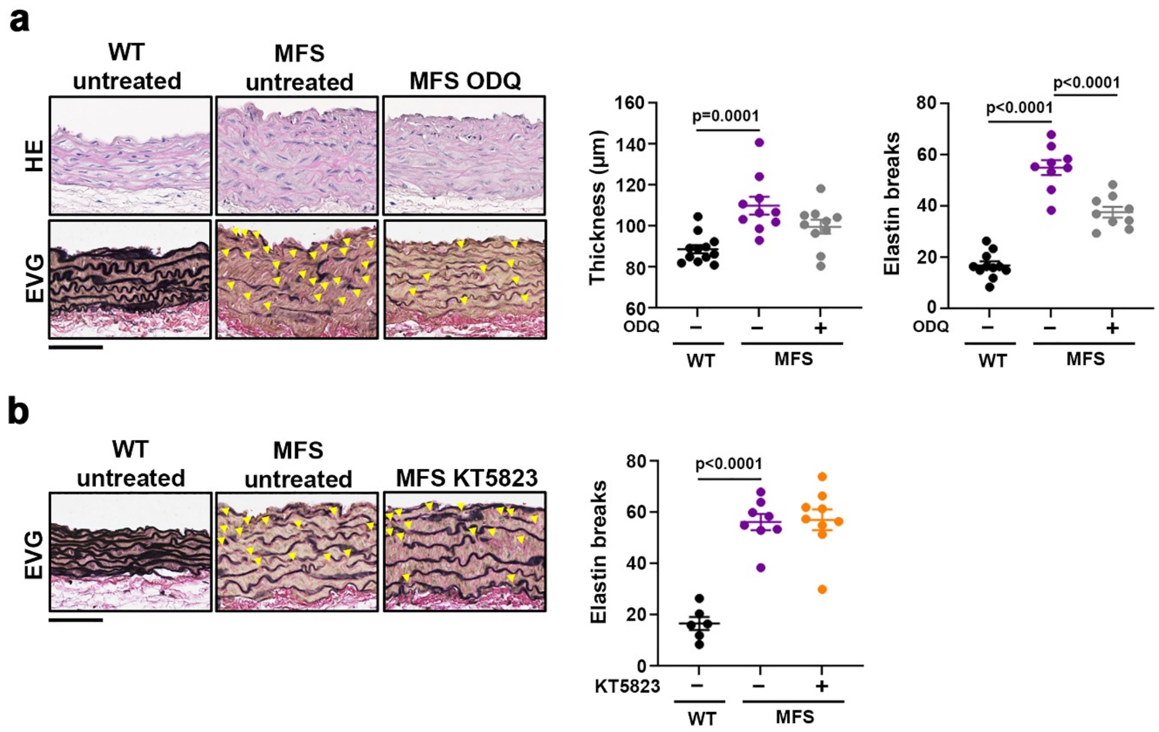

Supplementary Figure 11. Long term inhibition of sGC-PRKGI pathway reverts MFS-like medial degeneration. (**a**) Representative images showing hematoxylin and eosin (HE) and elastic van Gieson (EVG) staining in AsAo from untreated WT and MFS mice and MFS mice treated with ODQ for 21 days. Yellow arrowheads indicate elastin breaks. Scale bar, 50 µm. Graphs show quantification of thickness (*n*=12 WT mice, *n*=10 untreated and ODQ-treated MFS mice) and elastin breaks (*n*=11 WT mice, *n*=9 untreated and ODQ-treated MFS mice). Data are shown relative to untreated WT mice as mean ± s.e.m . Each data point denotes an individual mouse. Statistical significance is determined by one-way ANOVA followed by Dunnett's post hoc test. (**b**) Representative images showing EVG staining and elastin breaks in AsAo sections from untreated WT and MFS mice and MFS mice treated with KT5823 for 7 days. Yellow arrowheads indicate elastin breaks. Scale bar, 50 µm. Graphs show quantification of elastin breaks. Data are shown as mean ± s.e.m (*n*=6 WT mice, *n*=8 untreated MFS mice, and *n*=9 ODQ-treated MFS mice). Each data point denotes an individual mouse. Differences were analyzed by one-way ANOVA followed by Dunnett's post hoc test (p-values are shown). Source data are provided in the Source Data file.

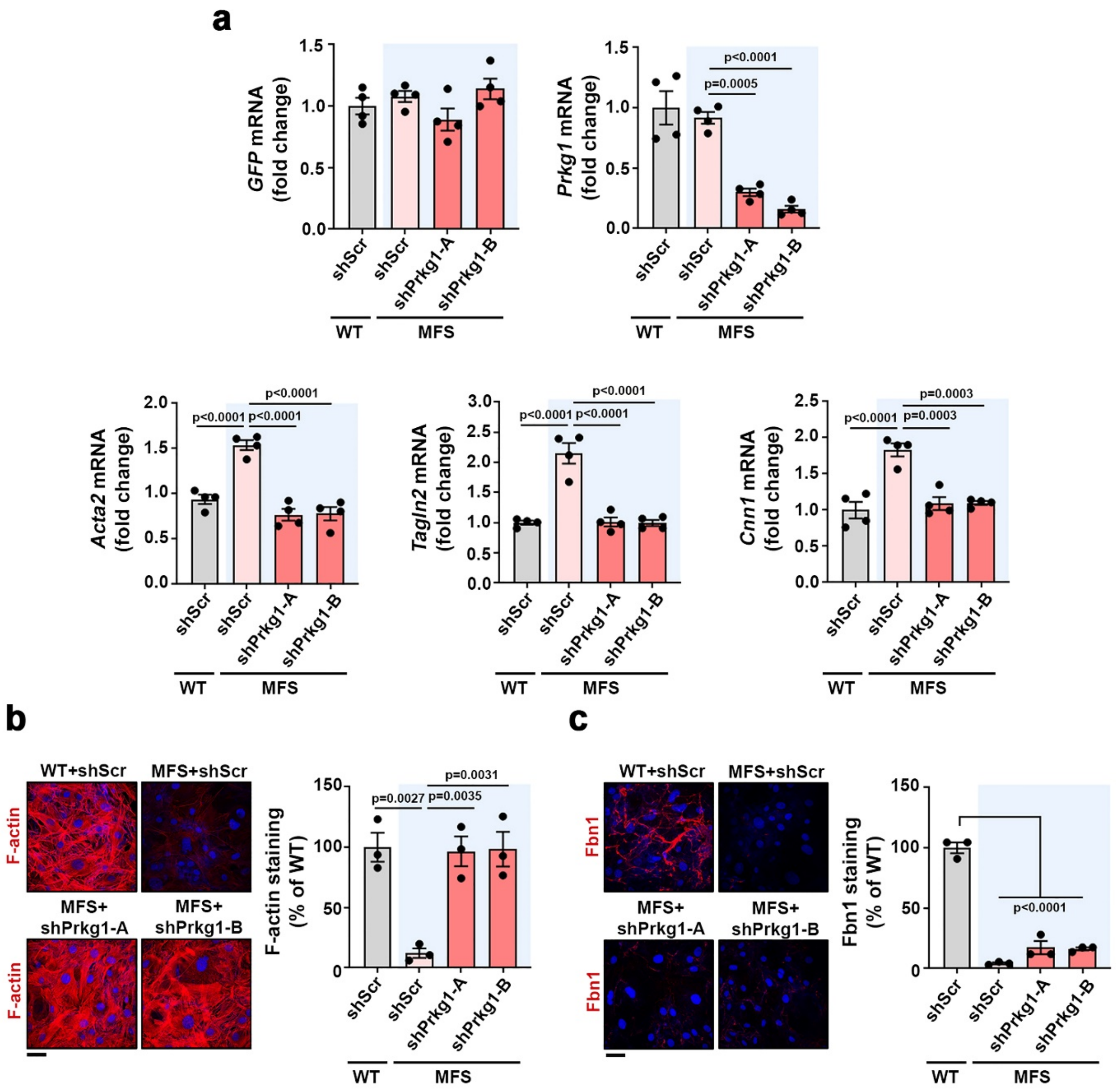

Supplementary Figure 12. ***Prkg1*** **silencing reverses the phenotypic switch in MFS VSMCs.** (**a**) RT-qPCR analysis of *GFP, Prkg1, Acta2, Tagln2* and *Cnn1* mRNA expression in WT and MFS VSMCs transduced with lentivirus (LVi) encoding control shRNA (shScr) or *Prkg1*-specific shRNAs A or B, as indicated (4 independent batches per condition). mRNA amounts were normalized to *Gapdh* expression (mean ± s.e.m.) (**b**) Representative images of F-actin (red) and DAPI-stained nuclei (blue) in VSMCs transduced with LVi encoding control shRNA (shScr) or *Prkg1*-specific shRNAs A or B, as indicated (3 independent batches per condition). The graph shows F-actin quantification. (**c**) Representative images of fibrillin-1 (red) and DAPI-stained nuclei (blue) in VSMCs transduced with LVi encoding control shRNA (shScr)

or *Prkg1*-specific shRNAs A or B, as indicated (3 independent batches per condition). Scale bar, 50 µm. The graph shows fibrillin-1 quantification. Data are shown relative to WT cells infected with shScr as mean ± s.e.m. Differences were analyzed by one-way ANOVA followed by Tukey's post hoc test (p-values are shown). Source data are provided in the Source Data file.

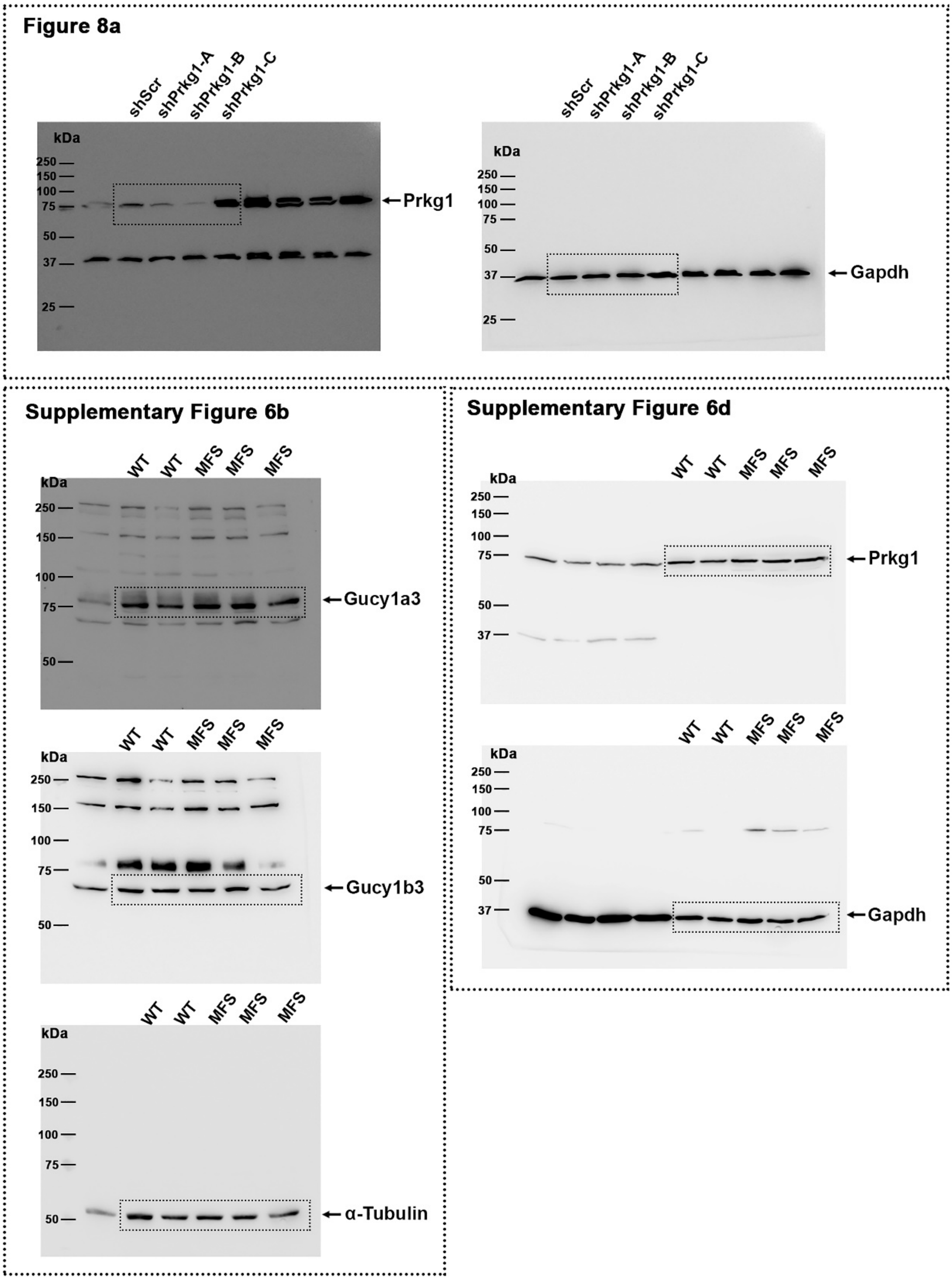

Supplementary Figure 13. Representative uncropped images of immunoblots shown in Figure 8 and Supplementary Figure 6.

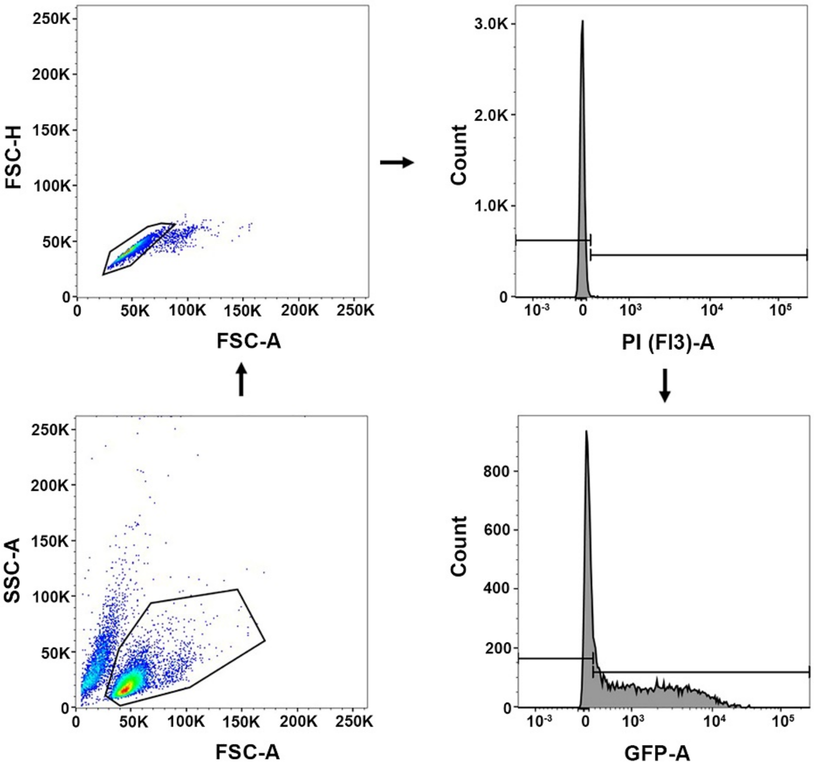

Supplementary Figure 14. Gating strategy for evaluating transduction efficiency (% of GFP-expressing cells) and cell death (propidium iodide incorporation). An initial FSC-A and SSC-A density plot was used to identify Jurkat cell population. This population was gated in a FSC-H vs FSC-A plot to select single cells and exclude doublet cells. Then, alive cells (Propidium Iodide negative cells) were identified with a PI (FI3)-A) histogram. Finally, the alive Jurkat population was gated for GFP-A in a histogram. The threshold for the histogram was determined using non-infected Jurkat cells as negative control.

Supplementary Table 1. Name and sequence of primers used for RT-PCR.

mRNA targets	Descriptions	Sense	Antisense
Acta2	Actin alpha 2 smooth muscle	ATCGTCCACCGCAAATGC	AAGGAACTGGAGGCGCTG
Cnn1	Calponin 1	AACTTCATGGATGGCCTCAAA	ACCCGGCTGCAGCTTGT
Tagln2	Transgelin 2	CCTGGCCGTGAGAACTTCC	GTCCGTGGTGTTAATGCCATAG
Prkg1	cGMP-dependent protein kinase 1	GGCTGATGTCCTCGAAGAGAC	ACCTGCCCTTTACTGATGATGA
Gucy1a3	Guanylate cyclase soluble subunit alpha-1	CCCCTGGTCAGGTTCCTAAG	GGAGACTCCCTTCTGCATTCT
Gucy1b3	Guanylate cyclase soluble subunit beta-1	CTCAACAGATACACGGCACTG	GCTGATACGTGATTCCTGGGT
GFP	Green Fluorescent Protein	CAAGCAGAAGAACGGCATCA	GGTGTTCTGCTGGTAGTGGT
Gadph	Glyceraldehyde-3-Phosphate Dehydrogenase	TGACGTGCCGCCTGGAGAAA	AGTGTAGCCCAAGATGCCCTTCA
