# Supplementary material for: Aortic disease in Marfan syndrome is caused by overactivation of sGC-PRKG signaling by NO
Source: Nat Commun. 2021 May 11;12:2628. doi: 10.1038/s41467-021-22933-3 (PMC8113458; doi:10.1038/s41467-021-22933-3)
Supplement: Supplementary file 3 — Description of Additional Supplementary Files [file 41467_2021_22933_MOESM3_ESM.pdf]

Description of Additional Supplementary Files

Supplementary Data 1.

Description: Information about the nitrated peptide sequences identified in mouse plasma. All the non-modified sequences identified for each nitrated protein are provided in an additional tab ("Additional information non mod") in the excel file. All MS/MS spectra of nitro-peptides identified are freely available at: http://www.peptideatlas.org/PASS/PASS01528.

Supplementary Data 2.

Description: Representative annotated spectra of all nitrated peptides identified in mouse plasma. A fragment ion table is presented per each nitrated peptide, followed by the annotated MS/MS spectrum. The fragment ion table contains the fragment ions automatically assigned by Proteome Discoverer 2.1 (Thermo Fisher Scientific) assigned for the most complete series, extracted from a representative scan among all PSMs. Some fragments are colored in red (assigned b-ions), blue (assigned y-ions) or pink (assigned immonium ions). Note that in some cases the immonium ion from nitroTyr or nitroTrp is observed in the MS/MS spectrum but is not automatically labeled by the program; these cases are indicated in the table. The amino acid sequence and their modifications are shown in the central column. The corresponding annotated MS/MS spectrum contains the header information indicating raw file, scan number, retention time (RT), charge (z), monoisotopic mass and match tolerance. The peptides are ordered according to Supplementary Data 1. The raw data are freely available at: http://www.peptideatlas.org/PASS/PASS01528.

Supplementary Data 3.

Description: Information about the nitrated peptide sequences identified in human plasma. The data are presented as in Supplementary Data 1.

Supplementary Data 4.

Description: Representative annotated spectra of all nitrated peptides identified in human plasma. The annotated MS/MS spectra are presented as in Supplementary Data 2.

Supplementary Data 5.

Description: Information about the nitrated peptide sequences identified in mouse ascending aorta (AsAo). The data are presented as in Supplementary Data 1. The raw data are freely available at: http://www.peptideatlas.org/PASS/PASS01528.
