# Supplementary material for: Aortic disease in Marfan syndrome is caused by overactivation of sGC-PRKG signaling by NO
Source: Nat Commun. 2021 May 11;12:2628. doi: 10.1038/s41467-021-22933-3 (PMC8113458; doi:10.1038/s41467-021-22933-3)
Supplement: Supplementary file 5 — Supplementary Data 2 [file 41467_2021_22933_MOESM5_ESM.pdf]

#1	Immonium	b⁺	b²⁺	b³⁺	Seq.	y⁺	y²⁺	y³⁺	#2
1	273.21241	301.20732	151.10730	101.07396	A-TMT6plex				17
2	102.05496	430.24992	215.62860	144.08816	E	2376.19395	1188.60061	792.73617	16
3	86.09643	543.33398	272.17063	181.78284	L	2247.15136	1124.07932	749.72197	15
4	44.04948	614.37109	307.68918	205.46188	A	2134.06730	1067.53729	712.02728	14
5	330.27026	971.62899	486.31813	324.54785	K-TMT6plex	2063.03018	1032.01873	688.34824	13
6	181.06077	1179.67739	590.34234	393.89732	Y-Nitro	1705.77229	853.38978	569.26228	12
7	104.05285	1310.71788	655.86258	437.57748	M	1497.72388	749.36558	499.91281	11
8	133.04301	1470.74853	735.87790	490.92103	C-Carbamidomethyl	1366.68340	683.84534	456.23265	10
9	102.05496	1599.79112	800.39920	533.93522	E	1206.65275	603.83001	402.88910	9
10	87.05529	1713.83405	857.42066	571.94953	N	1077.61015	539.30872	359.87490	8
11	101.07094	1841.89263	921.44995	614.63573	Q	963.56723	482.28725	321.86059	7
12	44.04948	1912.92974	956.96851	638.31476	A	835.50865	418.25796	279.17440	6
13	74.06004	2013.97742	1007.49235	671.99732	T	764.47154	382.73941	255.49536	5
14	86.09643	2127.06148	1064.03438	709.69201	I	663.42386	332.21557	221.81280	4
15	60.04439	2214.09351	1107.55039	738.70269	S	550.33979	275.67353	184.11812	3
16	60.04439	2301.12554	1151.06641	767.71336	S	463.30776	232.15752	155.10744	2
17	330.27026				K-TMT6plex	376.27574	188.64151	126.09676	1

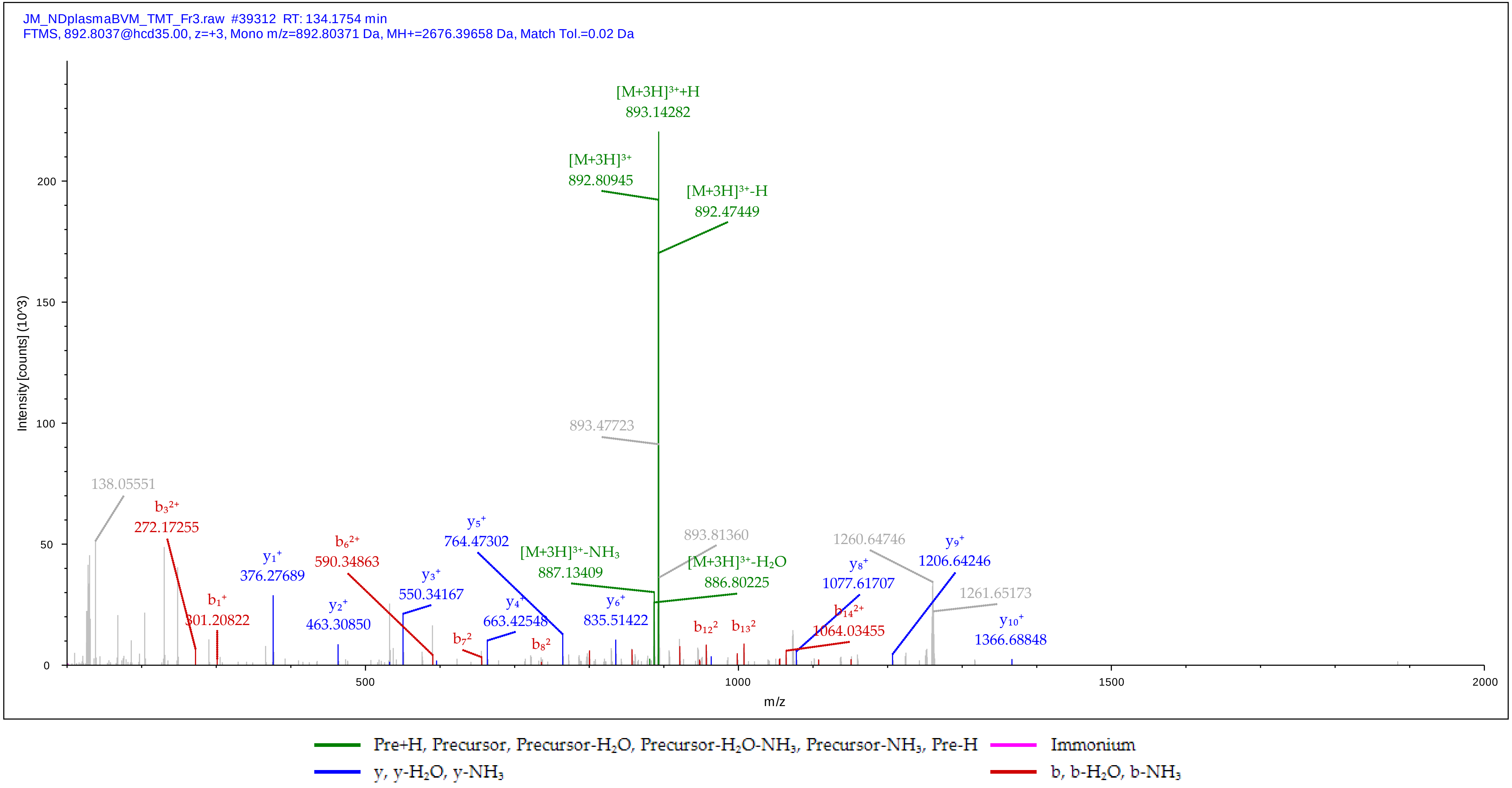
JM_NDplasmaBVM_TMT_Fr3.raw #39312 RT: 134.1754 min
FTMS, 892.8037@hcd35.00, z=+3, Mono m/z=892.80371 Da, MH+=2676.39658 Da, Match Tol.=0.02 Da
[M+3H]3++H 893.14282
[M+3H]3+ 892.80945
[M+3H]3+-H 892.47449
893.47723
138.05551
b3 2+ 272.17255
b1+ 301.20822
y1+ 376.27689
y2+ 463.30850
b6 2+ 590.34863
y3+ 550.34167
y5+ 764.47302
b7 2
y4+ 663.42548
b8 2
[M+3H]3+-NH3 887.13409
y6+ 835.51422
893.81360
[M+3H]3+-H2O 886.80225
b12 2
b13 2
1260.64746
y8+ 1077.61707
b14 2+ 1064.03455
y9+ 1206.64246
1261.65173
y10+ 1366.68848
Intensity [counts] (10^3)
m/z
Pre+H, Precursor, Precursor-H2O, Precursor-H2O-NH3, Precursor-NH3, Pre-H
Immonium
y, y-H2O, y-NH3
b, b-H2O, b-NH3

#1	Immonium	b^{+}	b^{2+}	b^{3+}	Seq.	y^{+}	y^{2+}	y^{3+}	#2
1	315.25936	343.25427	172.13077	115.08961	L-TMT6plex				20
2	133.04301	503.28492	252.14610	168.43316	C-Carbamidomethyl	2558.16351	1279.58539	853.39269	19
3	44.04948	574.32203	287.66466	192.11220	A	2398.13286	1199.57007	800.04914	18
4	86.09643	687.40610	344.20669	229.80688	I	2327.09575	1164.05151	776.37010	17
5	70.06513	784.45886	392.73307	262.15781	P	2214.01168	1107.50948	738.67541	16
6	87.05529	898.50179	449.75453	300.17211	N	2116.95892	1058.98310	706.32449	15
7	86.09643	1011.58585	506.29657	337.86680	L	2002.91599	1001.96163	668.31018	14
8	129.11347	1167.68696	584.34712	389.90051	R	1889.83193	945.41960	630.61549	13
9	102.05496	1296.72956	648.86842	432.91470	E	1733.73082	867.36905	578.58179	12
10	87.05529	1410.77249	705.88988	470.92901	N	1604.68822	802.84775	535.56759	11
11	181.06077	1618.82089	809.91408	540.27848	Y-Nitro	1490.64530	745.82629	497.55328	10
12	30.03383	1675.84236	838.42482	559.28564	G	1282.59689	641.80208	428.20381	9
13	102.05496	1804.88495	902.94611	602.29983	E	1225.57543	613.29135	409.19666	8
14	86.09643	1917.96901	959.48814	639.99452	L	1096.53283	548.77005	366.18246	7
15	44.04948	1989.00613	995.00670	663.67356	A	983.44877	492.22802	328.48777	6
16	88.03930	2104.03307	1052.52017	702.01587	D	912.41165	456.70947	304.80874	5
17	133.04301	2264.06372	1132.53550	755.35942	C-Carbamidomethyl	797.38471	399.19599	266.46642	4
18	133.04301	2424.09437	1212.55082	808.70297	C-Carbamidomethyl	637.35406	319.18067	213.12287	3
19	74.06004	2525.14204	1263.07466	842.38553	T	477.32341	239.16535	159.77932	2
20	330.27026				K-TMT6plex	376.27574	188.64151	126.09676	1

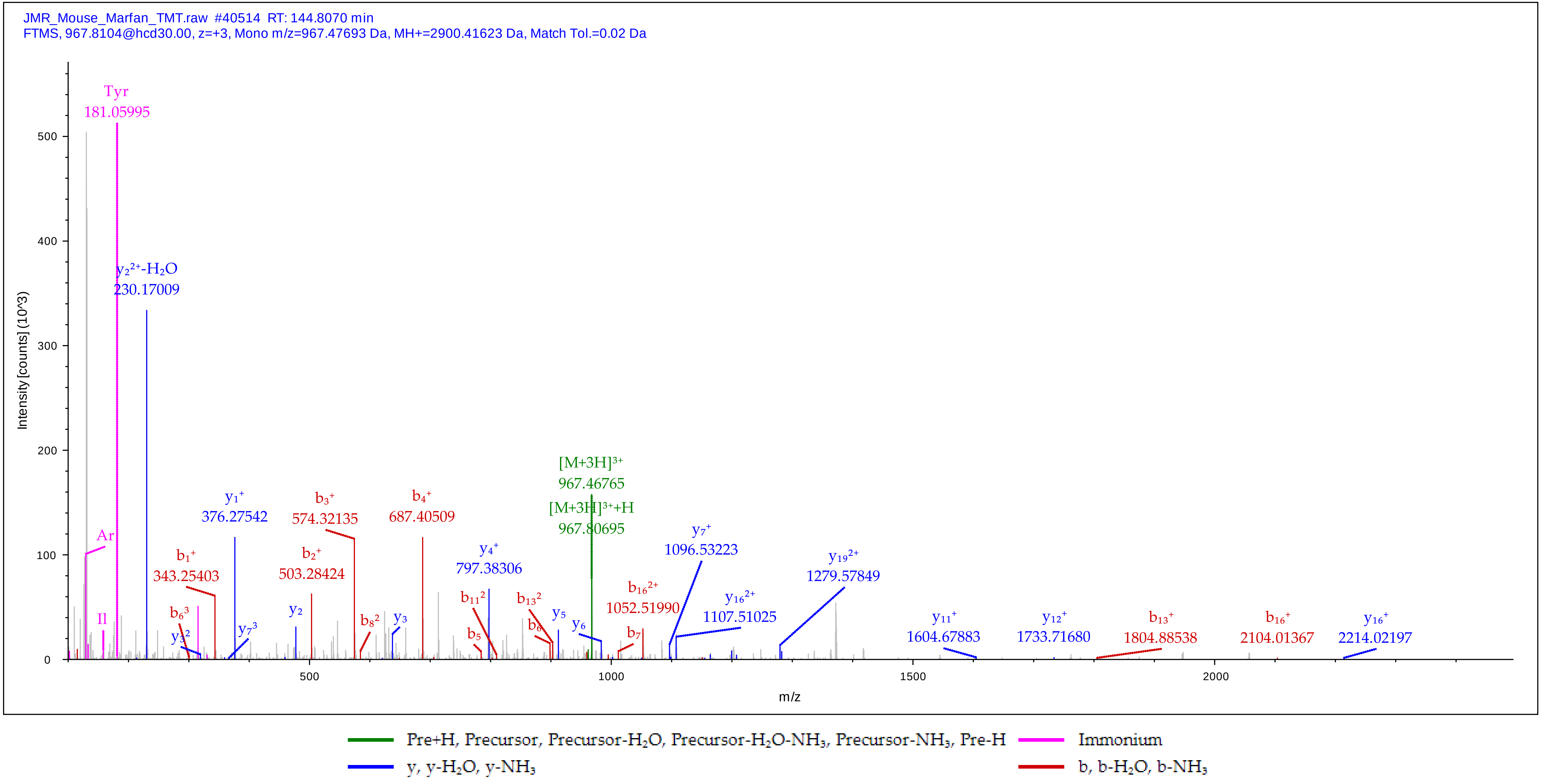

JMR_Mouse_Marfan_TMT.raw #40514 RT: 144.8070 min
FTMS, 967.8104@hcd30.00, z=+3, Mono m/z=967.47693 Da, MH+=2900.41623 Da, Match Tol.=0.02 Da
Intensity [counts] (10^3)
m/z
Tyr 181.05995
y2 2+-H2O 230.17009
Ar
Il
b1+ 343.25403
y1+ 376.27542
b2+ 503.28424
b3+ 574.32135
b4+ 687.40509
y4+ 797.38306
[M+3H]3+ 967.46765
[M+3H]3++H 967.80695
y7+ 1096.53223
b16 2+ 1052.51990
y16 2+ 1107.51025
y19 2+ 1279.57849
y11+ 1604.67883
y12+ 1733.71680
b13+ 1804.88538
b16+ 2104.01367
y16+ 2214.02197
Pre+H, Precursor, Precursor-H2O, Precursor-H2O-NH3, Precursor-NH3, Pre-H
Immonium
y, y-H2O, y-NH3
b, b-H2O, b-NH3

#1	Immonium	b⁺	b^{2+}	b^{3+}	b^{4+}	Seq.	y⁺	y^{2+}	y^{3+}	y^{4+}	#2
1	362.20594	390.20086	195.60407	130.73847	98.30567	C-TMT6plex-Carbamidomethyl					26
2	133.04301	550.23151	275.61939	184.08202	138.31333	C-Carbamidomethyl	3064.45934	1532.73331	1022.15796	766.87029	25
3	44.04948	621.26862	311.13795	207.76106	156.07261	A	2904.42869	1452.71798	968.81441	726.86263	24
4	102.05496	750.31121	375.65924	250.77526	188.33326	E	2833.39158	1417.19943	945.13538	709.10335	23
5	44.04948	821.34833	411.17780	274.45429	206.09254	A	2704.34898	1352.67813	902.12118	676.84270	22
6	87.05529	935.39125	468.19927	312.46860	234.60327	N	2633.31187	1317.15957	878.44214	659.08342	21
7	70.06513	1032.44402	516.72565	344.81952	258.86646	P	2519.26894	1260.13811	840.42783	630.57269	20
8	70.06513	1129.49678	565.25203	377.17044	283.12965	P	2422.21618	1211.61173	808.07691	606.30950	19
9	44.04948	1200.53389	600.77059	400.84948	300.88893	A	2325.16341	1163.08535	775.72599	582.04631	18
10	133.04301	1360.56454	680.78591	454.19303	340.89659	C-Carbamidomethyl	2254.12630	1127.56679	752.04695	564.28703	17
11	181.06077	1568.61295	784.81011	523.54250	392.90869	Y-Nitro	2094.09565	1047.55146	698.70340	524.27937	16
12	30.03383	1625.63441	813.32085	542.54966	407.16406	G	1886.04725	943.52726	629.35393	472.26727	15
13	74.06004	1726.68209	863.84468	576.23222	432.42598	T	1829.02578	915.01653	610.34678	458.01190	14
14	72.08078	1825.75051	913.37889	609.25502	457.19308	V	1727.97810	864.49269	576.66422	432.74998	13
15	86.09643	1938.83457	969.92092	646.94971	485.46410	L	1628.90969	814.95848	543.64141	407.98288	12
16	44.04948	2009.87168	1005.43948	670.62875	503.22338	A	1515.82563	758.41645	505.94673	379.71186	11
17	102.05496	2138.91428	1069.96078	713.64294	535.48403	E	1444.78851	722.89789	482.26769	361.95259	10
18	120.08078	2285.98269	1143.49498	762.66575	572.25113	F	1315.74592	658.37660	439.25349	329.69194	9
19	101.07094	2414.04127	1207.52427	805.35194	604.26577	Q	1168.67751	584.84239	390.23069	292.92483	8
20	70.06513	2511.09403	1256.05065	837.70286	628.52897	P	1040.61893	520.81310	347.54449	260.91019	7
21	86.09643	2624.17810	1312.59269	875.39755	656.79998	L	943.56616	472.28672	315.19357	236.64700	6
22	72.08078	2723.24651	1362.12689	908.42035	681.56708	V	830.48210	415.74469	277.49888	208.37598	5
23	102.05496	2852.28910	1426.64819	951.43455	713.82773	E	731.41369	366.21048	244.47608	183.60888	4
24	102.05496	2981.33170	1491.16949	994.44875	746.08838	E	602.37109	301.68918	201.46188	151.34823	3
25	70.06513	3078.38446	1539.69587	1026.79967	770.35157	P	473.32850	237.16789	158.44768	119.08758	2
26	330.27026					K-TMT6plex	376.27574	188.64151	126.09676	94.82439	1

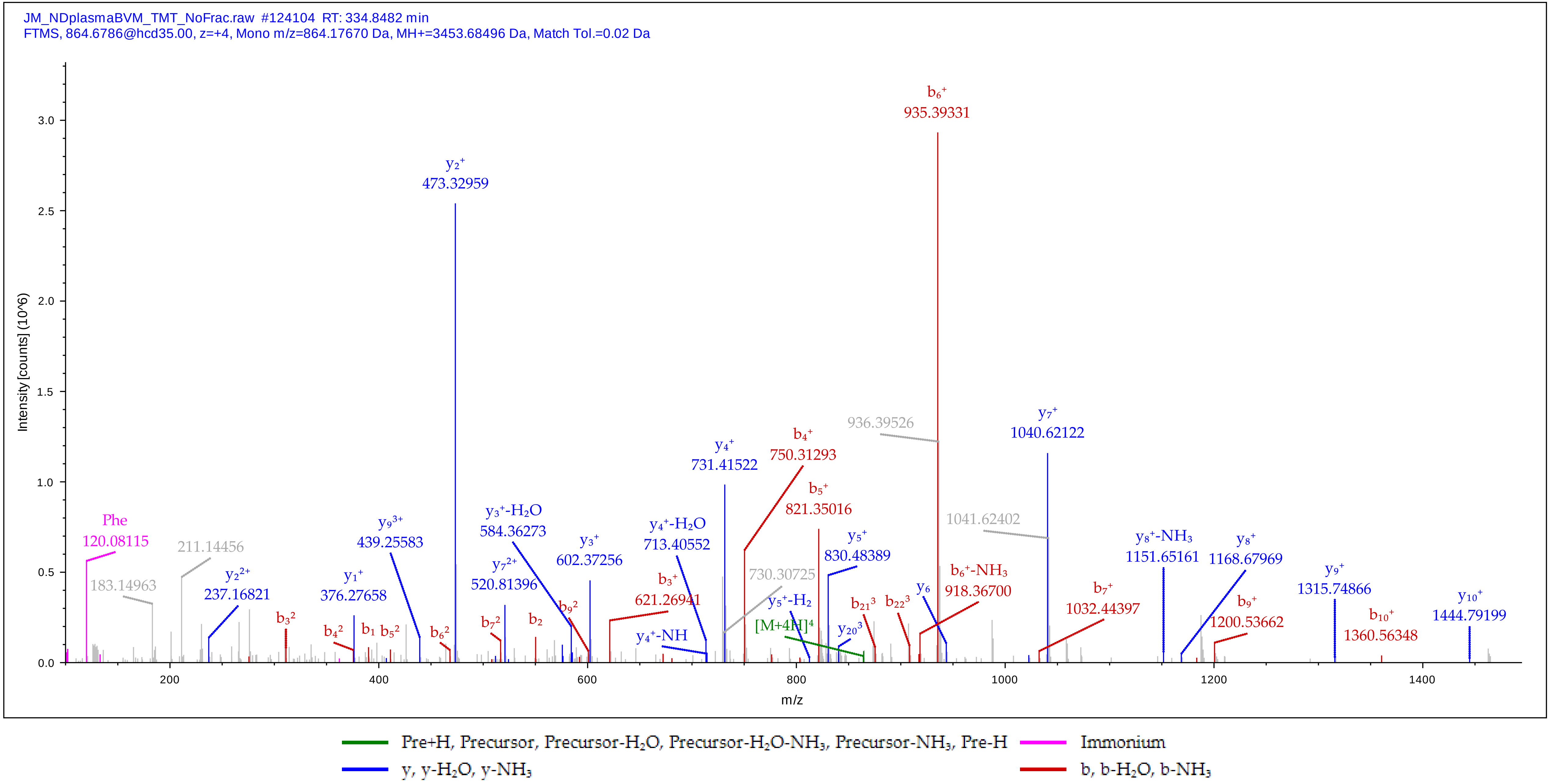

JM_NDplasmaBVM_TMT_NoFrac.raw #124104 RT: 334.8482 min
FTMS, 864.6786@hcd35.00, z=+4, Mono m/z=864.17670 Da, MH+=3453.68496 Da, Match Tol.=0.02 Da
Intensity [counts] (10^6)
m/z
b6+ 935.39331
y2+ 473.32959
936.39526
y7+ 1040.62122
y4+ 731.41522
b4+ 750.31293
b5+ 821.35016
1041.62402
Phe 120.08115
211.14456
183.14963
y2 2+ 237.16821
y1+ 376.27658
y9 3+ 439.25583
y3+-H2O 584.36273
y7 2+ 520.81396
y3+ 602.37256
y4+-H2O 713.40552
b3+ 621.26941
730.30725
y5+ 830.48389
y5+-H2
[M+4H]4
y20 3
b21 3
b22 3
y6
b6+-NH3 918.36700
b7+ 1032.44397
y8+-NH3 1151.65161
y8+ 1168.67969
b9+ 1200.53662
y9+ 1315.74866
b10+ 1360.56348
y10+ 1444.79199
b3 2
b4 2
b1
b5 2
b6 2
b7 2
b2
b9 2
y4+-NH
Pre+H, Precursor, Precursor-H2O, Precursor-H2O-NH3, Precursor-NH3, Pre-H
Immonium
y, y-H2O, y-NH3
b, b-H2O, b-NH3

#1	Immonium	b^+	b^{2+}	Seq.	y^+	y^{2+}	#2
1	331.21789	359.21280	180.11004	E-TMT6plex			16
2	87.05529	473.25573	237.13150	N	1817.83878	909.42303	15
3	70.06513	570.30849	285.65788	P	1703.79585	852.40156	14
4	74.06004	671.35617	336.18172	T	1606.74309	803.87518	13
5	74.06004	772.40385	386.70556	T	1505.69541	753.35134	12
6	120.08078	919.47226	460.23977	F	1404.64773	702.82750	11
7	104.05285	1050.51275	525.76001	M	1257.57932	629.29330	10
8	30.03383	1107.53421	554.27074	G	1126.53883	563.77305	9
9	110.07127	1244.59312	622.80020	H	1069.51737	535.26232	8
10	181.06077	1452.64153	726.82440	Y-Nitro	932.45846	466.73287	7
11	86.09643	1565.72559	783.36644	L	724.41005	362.70866	6
12	110.07127	1702.78451	851.89589	H	611.32598	306.16663	5
13	102.05496	1831.82710	916.41719	E	474.26707	237.63717	4
14	72.08078	1930.89551	965.95139	V	345.22448	173.11588	3
15	44.04948	2001.93263	1001.46995	A	246.15607	123.58167	2
16	129.11347			R	175.11895	88.06311	1

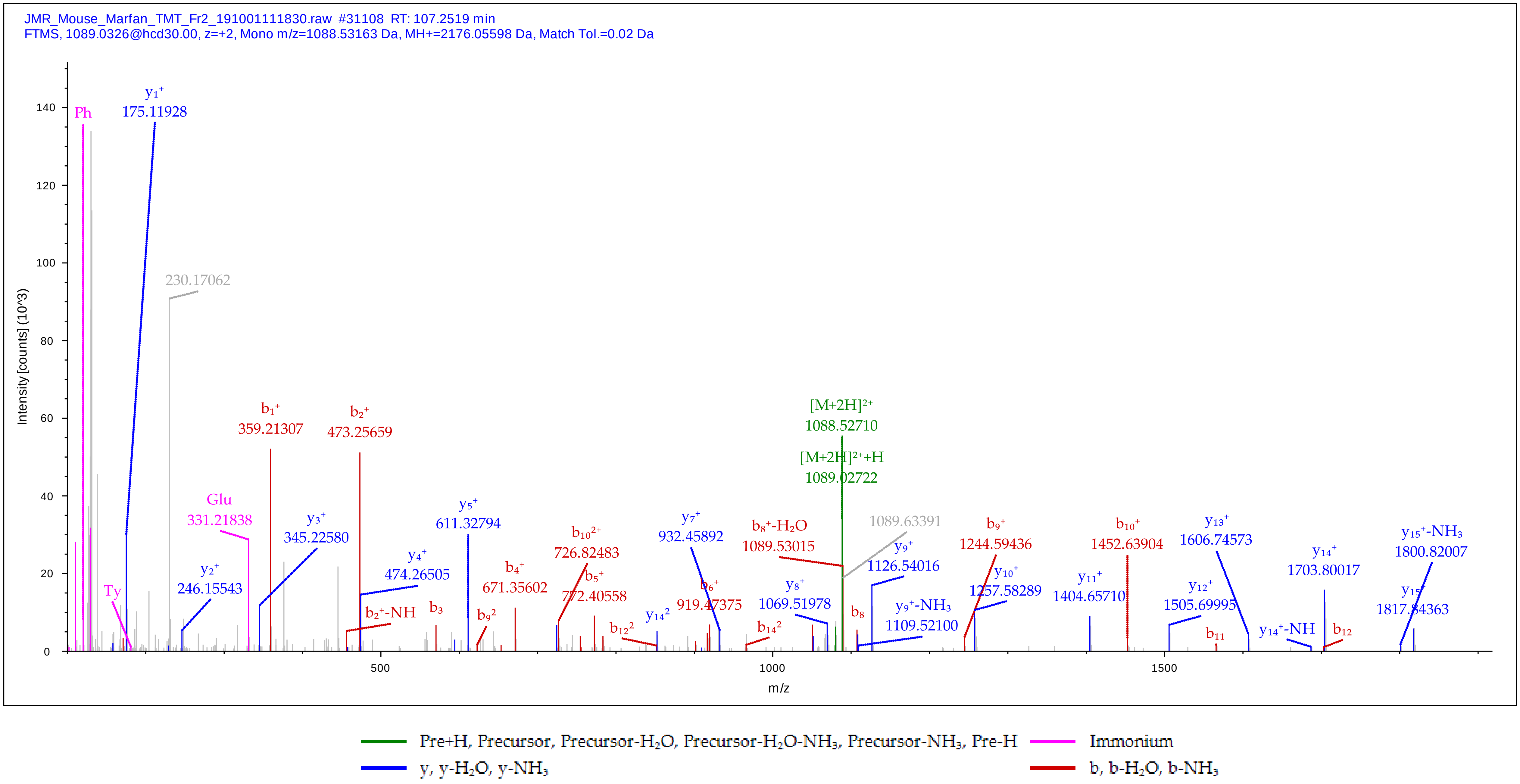
JMR_Mouse_Marfan_TMT_Fr2_191001111830.raw #31108 RT: 107.2519 min
FTMS, 1089.0326@hcd30.00, z=+2, Mono m/z=1088.53163 Da, MH+=2176.05598 Da, Match Tol.=0.02 Da
Intensity [counts] (10^3)
m/z
Ph
y1+ 175.11928
230.17062
Ty
Glu 331.21838
y2+ 246.15543
y3+ 345.22580
b1+ 359.21307
b2+ 473.25659
b2+-NH
y4+ 474.26505
b3
y5+ 611.32794
b9²
b4+ 671.35602
b10²+ 726.82483
b5+ 772.40558
b12²
y14²
y7+ 932.45892
b6+ 919.47375
b14²
b8+-H2O 1089.53015
y8+ 1069.51978
b8
[M+2H]²+ 1088.52710
[M+2H]²++H 1089.02722
1089.63391
y9+ 1126.54016
y9+-NH3 1109.52100
b9+ 1244.59436
y10+ 1257.58289
y11+ 1404.65710
b10+ 1452.63904
y12+ 1505.69995
b11
y13+ 1606.74573
y14+-NH
y14+ 1703.80017
b12
y15+ 1817.84363
y15+-NH3 1800.82007
Pre+H, Precursor, Precursor-H2O, Precursor-H2O-NH3, Precursor-NH3, Pre-H
Immonium
y, y-H2O, y-NH3
b, b-H2O, b-NH3

#1	Immonium	b^{+}	b^{2+}	b^{3+}	b^{4+}	b^{5+}	Seq.	y^{+}	y^{2+}	y^{3+}	y^{4+}	y^{5+}	#2
1	339.23421	367.22912	184.11820	123.08122	92.56274	74.25165	H-TMT6plex						36
2	70.06513	464.28188	232.64458	155.43215	116.82593	93.66220	P	4781.25015	2391.12871	1594.42157	1196.06800	957.05585	35
3	136.07569	627.34521	314.17624	209.78659	157.59176	126.27486	Y	4684.19739	2342.60233	1562.07065	1171.80480	937.64530	34
4	120.08078	774.41363	387.71045	258.80939	194.35886	155.68855	F	4521.13406	2261.07067	1507.71620	1131.03897	905.03263	33
5	136.07569	937.47696	469.24212	313.16384	235.12470	188.30121	Y	4374.06565	2187.53646	1458.69340	1094.27187	875.61895	32
6	44.04948	1008.51407	504.76067	336.84287	252.88397	202.50864	A	4211.00232	2106.00480	1404.33896	1053.50604	843.00628	31
7	70.06513	1105.56683	553.28705	369.19380	277.14717	221.91919	P	4139.96520	2070.48624	1380.65992	1035.74676	828.79886	30
8	102.05496	1234.60943	617.80835	412.20799	309.40781	247.72771	E	4042.91244	2021.95986	1348.30900	1011.48357	809.38831	29
9	86.09643	1347.69349	674.35038	449.90268	337.67883	270.34452	L	3913.86985	1957.43856	1305.29480	979.22292	783.57979	28
10	86.09643	1460.77755	730.89242	487.59737	365.94985	292.96133	L	3800.78578	1900.89653	1267.60011	950.95190	760.96298	27
11	136.07569	1623.84088	812.42408	541.95181	406.71568	325.57400	Y	3687.70172	1844.35450	1229.90542	922.68089	738.34617	26
12	136.07569	1786.90421	893.95574	596.30625	447.48151	358.18666	Y	3524.63839	1762.82283	1175.55098	881.91506	705.73350	25
13	44.04948	1857.94132	929.47430	619.98529	465.24079	372.39409	A	3361.57506	1681.29117	1121.19654	841.14922	673.12083	24
14	102.05496	1986.98392	993.99560	662.99949	497.50144	398.20260	E	3290.53795	1645.77261	1097.51750	823.38994	658.91341	23
15	101.07094	2115.04249	1058.02489	705.68568	529.51608	423.81432	Q	3161.49536	1581.25132	1054.50330	791.12930	633.10489	22
16	181.06077	2323.09090	1162.04909	775.03515	581.52818	465.42400	Y-Nitro	3033.43678	1517.22203	1011.81711	759.11465	607.49318	21
17	87.05529	2437.13383	1219.07055	813.04946	610.03891	488.23259	N	2825.38837	1413.19782	942.46764	707.10255	565.88350	20
18	102.05496	2566.17642	1283.59185	856.06366	642.29956	514.04111	E	2711.34544	1356.17636	904.45333	678.59182	543.07491	19
19	86.09643	2679.26049	1340.13388	893.75835	670.57058	536.65792	I	2582.30285	1291.65506	861.43913	646.33117	517.26639	18
20	86.09643	2792.34455	1396.67591	931.45303	698.84159	559.27473	L	2469.21879	1235.11303	823.74445	618.06015	494.64958	17
21	74.06004	2893.39223	1447.19975	965.13559	724.10351	579.48427	T	2356.13472	1178.57100	786.04976	589.78914	472.03277	16
22	101.07094	3021.45081	1511.22904	1007.82179	756.11816	605.09598	Q	2255.08704	1128.04716	752.36720	564.52722	451.82323	15
23	133.04301	3181.48145	1591.24437	1061.16534	796.12582	637.10211	C-Carbamidomethyl	2127.02847	1064.01787	709.68101	532.51257	426.21151	14
24	133.04301	3341.51210	1671.25969	1114.50889	836.13348	669.10824	C-Carbamidomethyl	1966.99782	984.00255	656.33746	492.50491	394.20539	13
25	44.04948	3412.54922	1706.77825	1138.18792	853.89276	683.31566	A	1806.96717	903.98722	602.99391	452.49725	362.19926	12
26	102.05496	3541.59181	1771.29954	1181.20212	886.15341	709.12418	E	1735.93006	868.46867	579.31487	434.73797	347.99183	11
27	44.04948	3612.62892	1806.81810	1204.88116	903.91269	723.33161	A	1606.88746	803.94737	536.30067	402.47732	322.18331	10
28	88.03930	3727.65587	1864.33157	1243.22347	932.66942	746.33699	D	1535.85035	768.42881	512.62163	384.71805	307.97589	9
29	330.27026	4084.91376	2042.96052	1362.30944	1021.98390	817.78857	K-TMT6plex	1420.82341	710.91534	474.27932	355.96131	284.97050	8
30	102.05496	4213.95635	2107.48182	1405.32364	1054.24455	843.59709	E	1063.56551	532.28639	355.19336	266.64684	213.51892	7
31	60.04439	4300.98838	2150.99783	1434.33431	1076.00255	861.00350	S	934.52292	467.76510	312.17916	234.38619	187.71041	6
32	133.04301	4461.01903	2231.01315	1487.67786	1116.01022	893.00963	C-Carbamidomethyl	847.49089	424.24908	283.16848	212.62818	170.30400	5
33	86.09643	4574.10309	2287.55519	1525.37255	1144.28123	915.62644	L	687.46024	344.23376	229.82493	172.62052	138.29787	4
34	74.06004	4675.15077	2338.07902	1559.05511	1169.54315	935.83598	T	574.37618	287.69173	192.13024	144.34950	115.68106	3
35	70.06513	4772.20354	2386.60541	1591.40603	1193.80634	955.24653	P	473.32850	237.16789	158.44768	119.08758	95.47152	2
36	330.27026						K-TMT6plex	376.27574	188.64151	126.09676	94.82439	76.06097	1

Nitro-Tyr immonium ion is detected in MS/MS spectra and added brown colored in the following spectrum

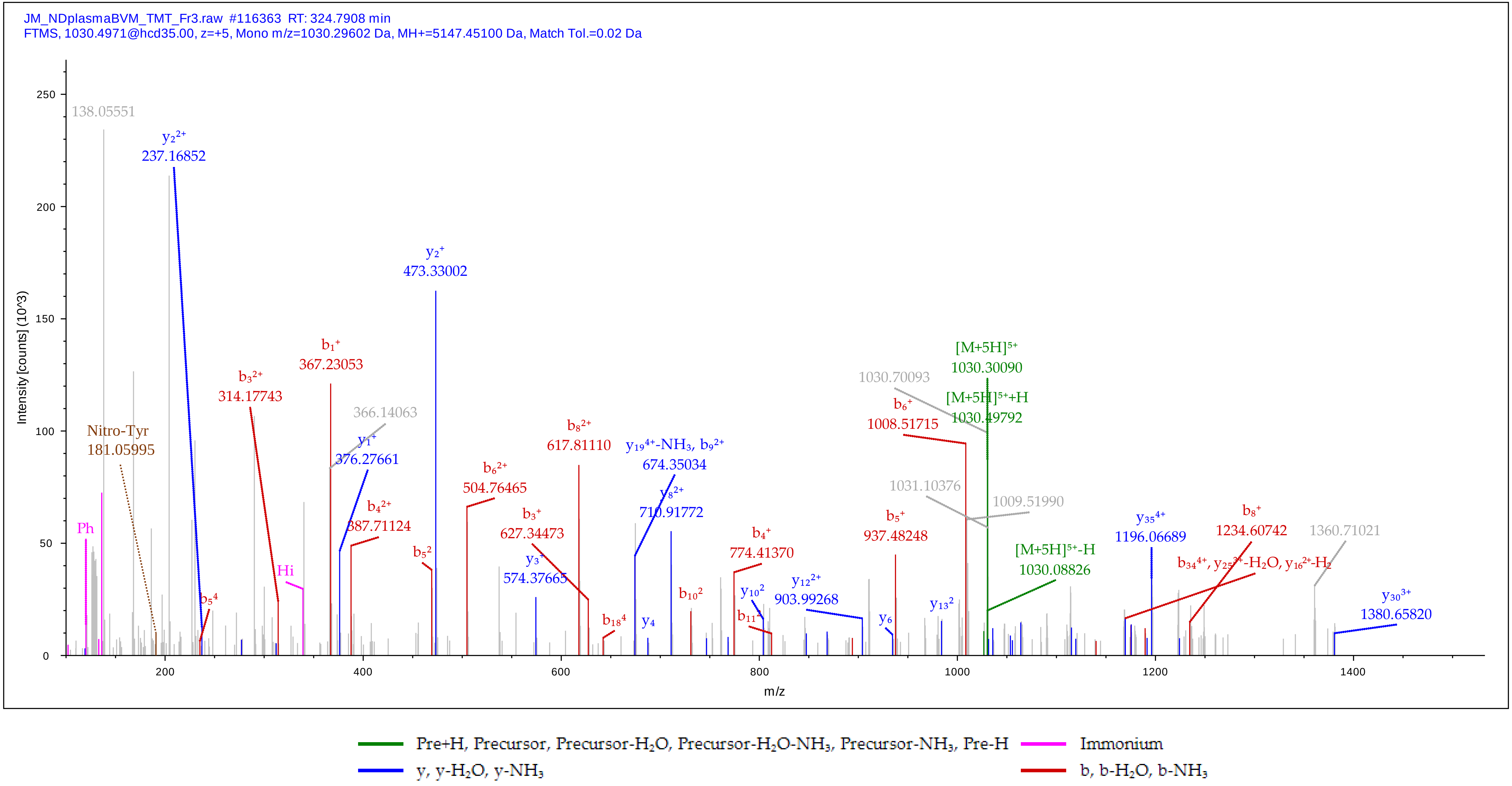
JM_NDplasmaBVM_TMT_Fr3.raw #116363 RT: 324.7908 min
FTMS, 1030.4971@hcd35.00, z=+5, Mono m/z=1030.29602 Da, MH+=5147.45100 Da, Match Tol.=0.02 Da
Intensity [counts] (10^3)
m/z
138.05551
y_2^{2+} 237.16852
y_2^{+} 473.33002
b_1^{+} 367.23053
b_3^{2+} 314.17743
366.14063
y_1^{+} 376.27661
Nitro-Tyr 181.05995
Ph
Hi
b_5^{4}
b_4^{2+} 387.71124
b_5^{2}
b_6^{2+} 504.76465
b_3^{+} 627.34473
y_3^{+} 574.37665
b_8^{2+} 617.81110
b_{18}^{4}
y_{19}^{4+}-NH_3, b_9^{2+} 674.35034
y_8^{2+} 710.91772
y_4
b_{10}^{2}
b_4^{+} 774.41370
y_{10}^{2}
b_{11}^{2}
y_{12}^{2+} 903.99268
b_5^{+} 937.48248
y_6
1030.70093
b_6^{+} 1008.51715
1031.10376
y_{13}^{2}
$[M+5H]^{5+}$ 1030.30090
$[M+5H]^{5+}$+H 1030.49792
1009.51990
$[M+5H]^{5+}$-H 1030.08826
y_{35}^{4+} 1196.06689
b_8^{+} 1234.60742
b_{34}^{4+}, y_{25}^{3+}-H_2O, y_{16}^{2+}-H_2
1360.71021
y_{30}^{3+} 1380.65820
Pre+H, Precursor, Precursor-H_2O, Precursor-H_2O-NH_3, Precursor-NH_3, Pre-H
Immonium
y, y-H_2O, y-NH_3
b, b-H_2O, b-NH_3

#1	Immonium	b^+	b^{2+}	b^{3+}	Seq.	y^+	y^{2+}	y^{3+}	#2
1	259.19676	287.19167	144.09947	96.40208	G-TMT6plex				13
2	86.09643	400.27574	200.64151	134.09676	L	1696.98352	848.99540	566.33269	12
3	72.08078	499.34415	250.17571	167.11957	V	1583.89946	792.45337	528.63800	11
4	86.09643	612.42821	306.71775	204.81426	L	1484.83105	742.91916	495.61520	10
5	86.09643	725.51228	363.25978	242.50894	I	1371.74698	686.37713	457.92051	9
6	44.04948	796.54939	398.77833	266.18798	A	1258.66292	629.83510	420.22582	8
7	120.08078	943.61781	472.31254	315.21079	F	1187.62580	594.31654	396.54679	7
8	60.04439	1030.64983	515.82856	344.22146	S	1040.55739	520.78233	347.52398	6
9	101.07094	1158.70841	579.85784	386.90765	Q	953.52536	477.26632	318.51330	5
10	181.06077	1366.75682	683.88205	456.25712	Y-Nitro	825.46678	413.23703	275.82711	4
11	86.09643	1479.84088	740.42408	493.95181	L	617.41838	309.21283	206.47764	3
12	101.07094	1607.89946	804.45337	536.63800	Q	504.33431	252.67080	168.78296	2
13	330.27026				K-TMT6plex	376.27574	188.64151	126.09676	1

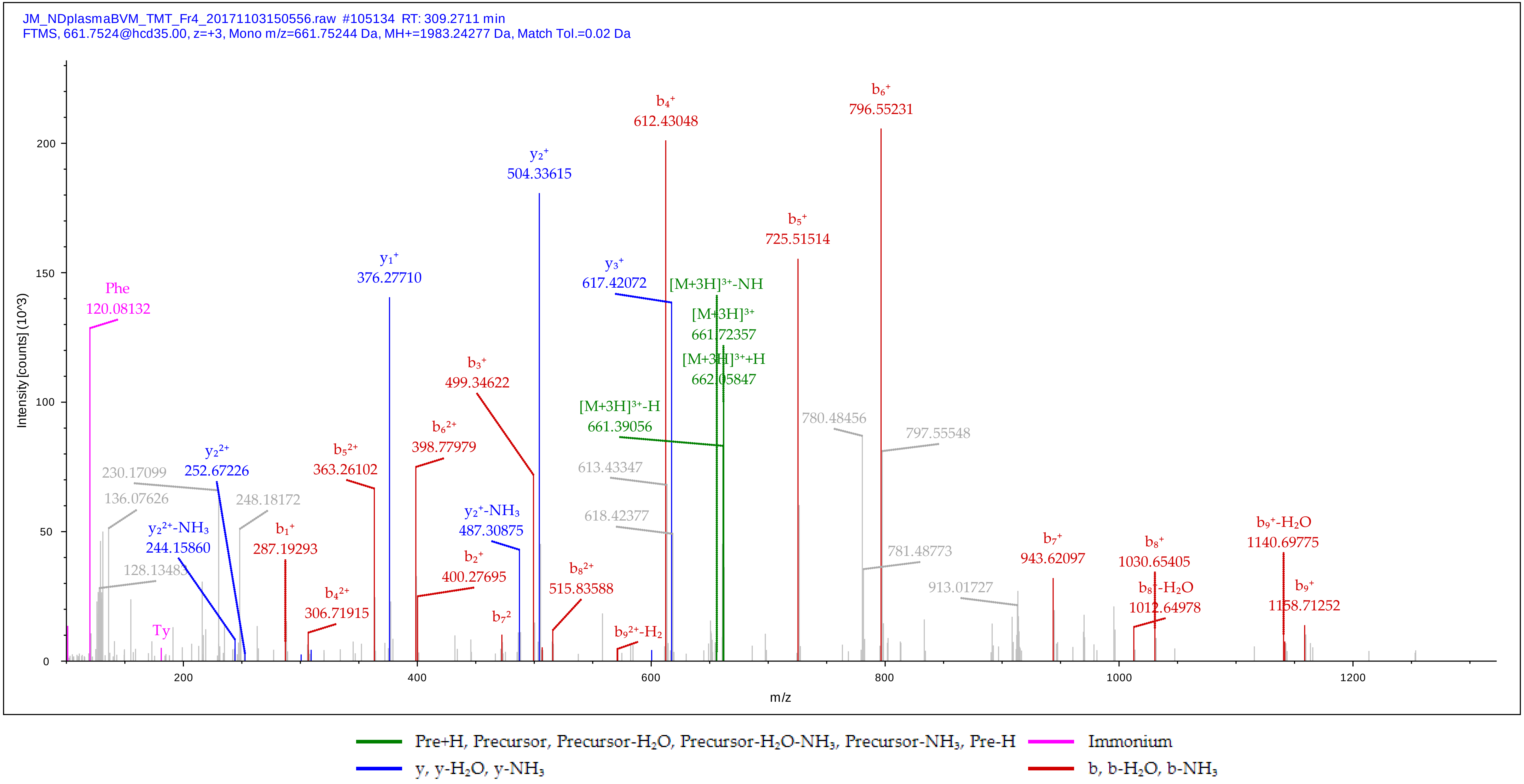

JM_NDplasmaBVM_TMT_Fr4_20171103150556.raw #105134 RT: 309.2711 min
FTMS, 661.7524@hcd35.00, z=+3, Mono m/z=661.75244 Da, MH+=1983.24277 Da, Match Tol.=0.02 Da
Intensity [counts] (10^3)
m/z
Phe 120.08132
Ty
y1+ 376.27710
y2+ 504.33615
y3+ 617.42072
y2 2+ 252.67226
y2 2+-NH3 244.15860
y2+-NH3 487.30875
b1+ 287.19293
b3+ 499.34622
b4+ 612.43048
b5+ 725.51514
b6+ 796.55231
b7+ 943.62097
b8+ 1030.65405
b8+-H2O 1012.64978
b9+-H2O 1140.69775
b9+ 1158.71252
b4 2+ 306.71915
b5 2+ 363.26102
b6 2+ 398.77979
b2+ 400.27695
b7 2
b8 2+ 515.83588
b9 2+-H2
[M+3H]3+-NH
[M+3H]3+ 661.72357
[M+3H]3++H 662.05847
[M+3H]3+-H 661.39056
230.17099
136.07626
248.18172
128.13483
613.43347
618.42377
780.48456
797.55548
781.48773
913.01727
Pre+H, Precursor, Precursor-H2O, Precursor-H2O-NH3, Precursor-NH3, Pre-H
Immonium
y, y-H2O, y-NH3
b, b-H2O, b-NH3

#1	Immonium	b⁺	b²⁺	b³⁺	Seq.	y⁺	y²⁺	y³⁺	#2
1	315.25936	343.25427	172.13077	115.08961	L-TMT6plex				14
2	70.06513	440.30704	220.65716	147.44053	P	1594.75298	797.88013	532.25585	13
3	133.04301	600.33768	300.67248	200.78408	Carbamidometl	1497.70022	749.35375	499.90492	12
4	72.08078	699.40610	350.20669	233.80688	V	1337.66957	669.33842	446.56137	11
5	102.05496	828.44869	414.72798	276.82108	E	1238.60116	619.80422	413.53857	10
6	88.03930	943.47563	472.24146	315.16340	D	1109.55856	555.28292	370.52437	9
7	181.06077	1151.52404	576.26566	384.51286	Y-Nitro	994.53162	497.76945	332.18206	8
8	86.09643	1264.60811	632.80769	422.20755	L	786.48321	393.74525	262.83259	7
9	60.04439	1351.64013	676.32371	451.21823	S	673.39915	337.20321	225.13790	6
10	44.04948	1422.67725	711.84226	474.89727	A	586.36712	293.68720	196.12722	5
11	86.09643	1535.76131	768.38429	512.59195	I	515.33001	258.16864	172.44819	4
12	86.09643	1648.84538	824.92633	550.28664	L	402.24594	201.62661	134.75350	3
13	87.05529	1762.88830	881.94779	588.30095	N	289.16188	145.08458	97.05881	2
14	129.11347				R	175.11895	88.06311	59.04450	1

Nitro-Tyr immonium ion is detected in MS/MS spectra and added brown colored in the following spectrum

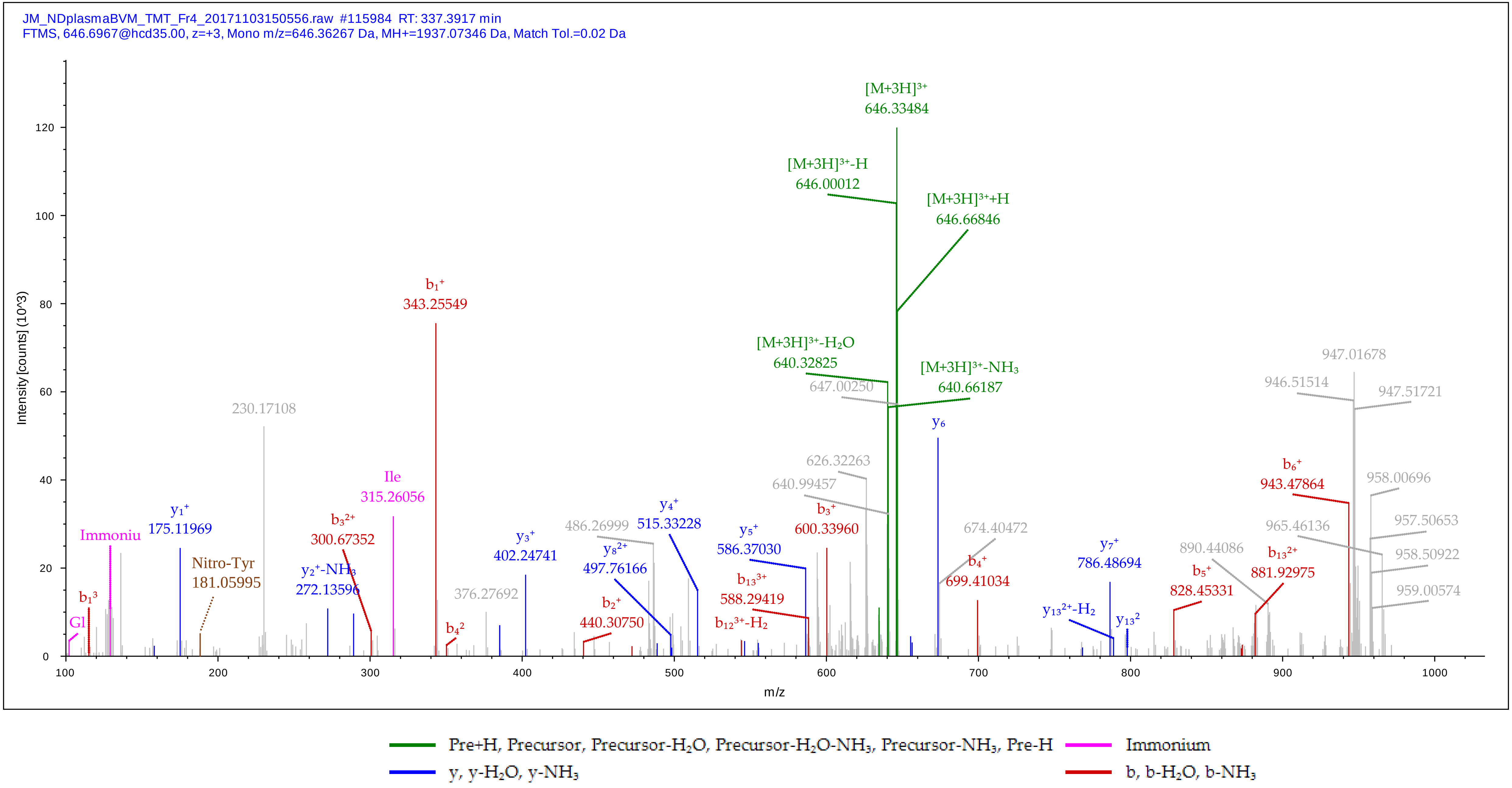

JM_NDplasmaBVM_TMT_Fr4_20171103150556.raw #115984 RT: 337.3917 min
FTMS, 646.6967@hcd35.00, z=+3, Mono m/z=646.36267 Da, MH+=1937.07346 Da, Match Tol.=0.02 Da
Intensity [counts] (10^3)
m/z
[M+3H]3+ 646.33484
[M+3H]3+-H 646.00012
[M+3H]3+ +H 646.66846
[M+3H]3+-H2O 640.32825
[M+3H]3+-NH3 640.66187
b1+ 343.25549
230.17108
Ile 315.26056
Immoniu
y1+ 175.11969
Nitro-Tyr 181.05995
b3 2+ 300.67352
y2+-NH3 272.13596
y3+ 402.24741
y4+ 515.33228
y8 2+ 497.76166
b2+ 440.30750
y5+ 586.37030
b3+ 600.33960
b13 3+ 588.29419
b12 3+-H2
y6
b4+ 699.41034
y7+ 786.48694
y13 2+-H2
b5+ 828.45331
b13 2+ 881.92975
b6+ 943.47864
947.01678
946.51514
947.51721
958.00696
957.50653
958.50922
959.00574
965.46136
890.44086
674.40472
647.00250
626.32263
640.99457
486.26999
376.27692
Pre+H, Precursor, Precursor-H2O, Precursor-H2O-NH3, Precursor-NH3, Pre-H
Immonium
y, y-H2O, y-NH3
b, b-H2O, b-NH3

#1	Immonium	b^+	b^{2+}	b^{3+}	b^{4+}	Seq.	y^+	y^{2+}	y^{3+}	y^{4+}	#2
1	358.27640	386.27132	193.63930	129.42862	97.32329	R-TMT6plex					16
2	70.06513	483.32408	242.16568	161.77955	121.58648	P	2000.98366	1000.99547	667.66607	501.00137	15
3	133.04301	643.35473	322.18100	215.12310	161.59414	C-Carbamidomethyl	1903.93090	952.46909	635.31515	476.73818	14
4	120.08078	790.42315	395.71521	264.14590	198.36124	F	1743.90025	872.45376	581.97160	436.73052	13
5	60.04439	877.45517	439.23123	293.15658	220.11925	S	1596.83183	798.91955	532.94880	399.96342	12
6	44.04948	948.49229	474.74978	316.83561	237.87853	A	1509.79980	755.40354	503.93812	378.20541	11
7	86.09643	1061.57635	531.29181	354.53030	266.14955	L	1438.76269	719.88498	480.25908	360.44613	10
8	74.06004	1162.62403	581.81565	388.21286	291.41147	T	1325.67863	663.34295	442.56439	332.17511	9
9	72.08078	1261.69244	631.34986	421.23567	316.17857	V	1224.63095	612.81911	408.88183	306.91319	8
10	88.03930	1376.71939	688.86333	459.57798	344.93530	D	1125.56253	563.28491	375.85903	282.14609	7
11	102.05496	1505.76198	753.38463	502.59218	377.19595	E	1010.53559	505.77143	337.51672	253.38936	6
12	74.06004	1606.80966	803.90847	536.27474	402.45787	T	881.49300	441.25014	294.50252	221.12871	5
13	181.06077	1814.85806	907.93267	605.62421	454.46997	Y-Nitro	780.44532	390.72630	260.81996	195.86679	4
14	72.08078	1913.92648	957.46688	638.64701	479.23708	V	572.39691	286.70210	191.47049	143.85469	3
15	70.06513	2010.97924	1005.99326	670.99793	503.50027	P	473.32850	237.16789	158.44768	119.08758	2
16	330.27026					K-TMT6plex	376.27574	188.64151	126.09676	94.82439	1

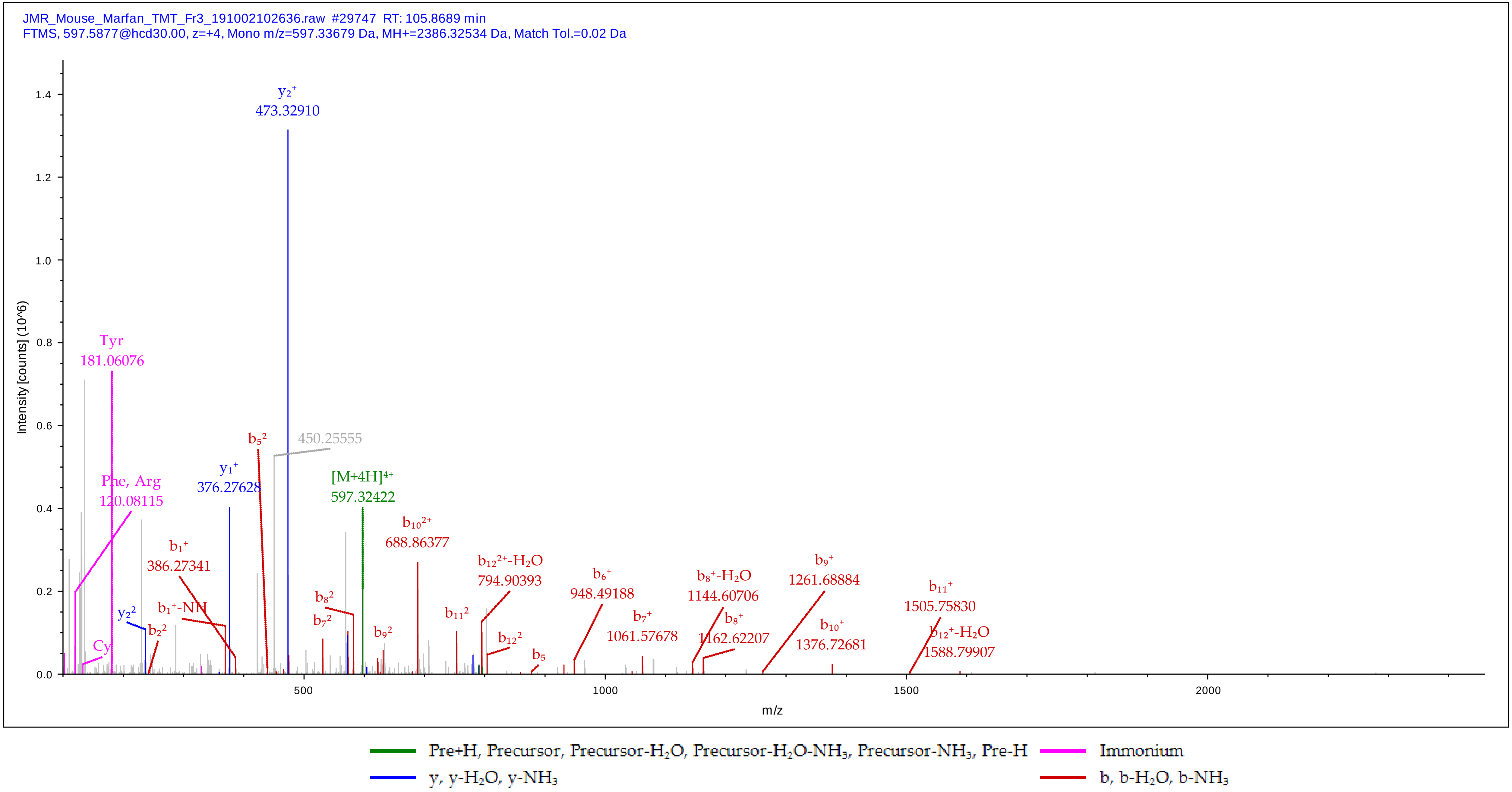

JMR_Mouse_Marfan_TMT_Fr3_191002102636.raw #29747 RT: 105.8689 min
FTMS, 597.5877@hcd30.00, z=+4, Mono m/z=597.33679 Da, MH+=2386.32534 Da, Match Tol.=0.02 Da
Intensity [counts] (10^6)
m/z
y2+ 473.32910
Tyr 181.06076
Phe, Arg 120.08115
y1+ 376.27628
450.25555
[M+4H]4+ 597.32422
b10 2+ 688.86377
b1+ 386.27341
b12 2+-H2O 794.90393
b6+ 948.49188
b8+-H2O 1144.60706
b9+ 1261.68884
b11+ 1505.75830
b7+ 1061.57678
b8+ 1162.62207
b10+ 1376.72681
b12+-H2O 1588.79907
Pre+H, Precursor, Precursor-H2O, Precursor-H2O-NH3, Precursor-NH3, Pre-H
Immonium
y, y-H2O, y-NH3
b, b-H2O, b-NH3

#1	Immonium	b⁺	b²⁺	b³⁺	Seq.	y⁺	y²⁺	y³⁺	#2
1	330.23387	358.22879	179.61803	120.08111	Q-TMT6plex				23
2	44.04948	429.26590	215.13659	143.76015	A	2820.23303	1410.62015	940.74919	22
3	88.03930	544.29284	272.65006	182.10247	D	2749.19591	1375.10159	917.07016	21
4	30.03383	601.31431	301.16079	201.10962	G	2634.16897	1317.58812	878.72784	20
5	70.06513	698.36707	349.68717	233.46054	P	2577.14751	1289.07739	859.72069	19
6	88.03930	813.39401	407.20064	271.80286	D	2480.09474	1240.55101	827.36977	18
7	104.05285	944.43450	472.72089	315.48302	M	2365.06780	1183.03754	789.02745	17
8	101.07094	1072.49308	536.75018	358.16921	Q	2234.02731	1117.51730	745.34729	16
9	60.04439	1159.52510	580.26619	387.17989	S	2105.96874	1053.48801	702.66110	15
10	86.09643	1272.60917	636.80822	424.87457	L	2018.93671	1009.97199	673.65042	14
11	120.08078	1419.67758	710.34243	473.89738	F	1905.85265	953.42996	635.95573	13
12	74.06004	1520.72526	760.86627	507.57994	T	1758.78423	879.89575	586.93293	12
13	101.07094	1648.78384	824.89556	550.26613	Q	1657.73655	829.37191	553.25037	11
14	181.06077	1856.83224	928.91976	619.61560	Y-Nitro	1529.67798	765.34263	510.56418	10
15	120.08078	2003.90066	1002.45397	668.63840	F	1321.62957	661.31842	441.21471	9
16	101.07094	2131.95924	1066.48326	711.32460	Q	1174.56116	587.78422	392.19190	8
17	60.04439	2218.99126	1109.99927	740.33527	S	1046.50258	523.75493	349.50571	7
18	120.04776	2366.02666	1183.51697	789.34707	M-Oxidation	959.47055	480.23891	320.49503	6
19	74.06004	2467.07434	1234.04081	823.02963	T	812.43515	406.72121	271.48323	5
20	88.03930	2582.10128	1291.55428	861.37195	D	711.38747	356.19737	237.80067	4
21	136.07569	2745.16461	1373.08594	915.72639	Y	596.36053	298.68390	199.45836	3
22	30.03383	2802.18608	1401.59668	934.73354	G	433.29720	217.15224	145.10392	2
23	330.27026				K-TMT6plex	376.27574	188.64151	126.09676	1

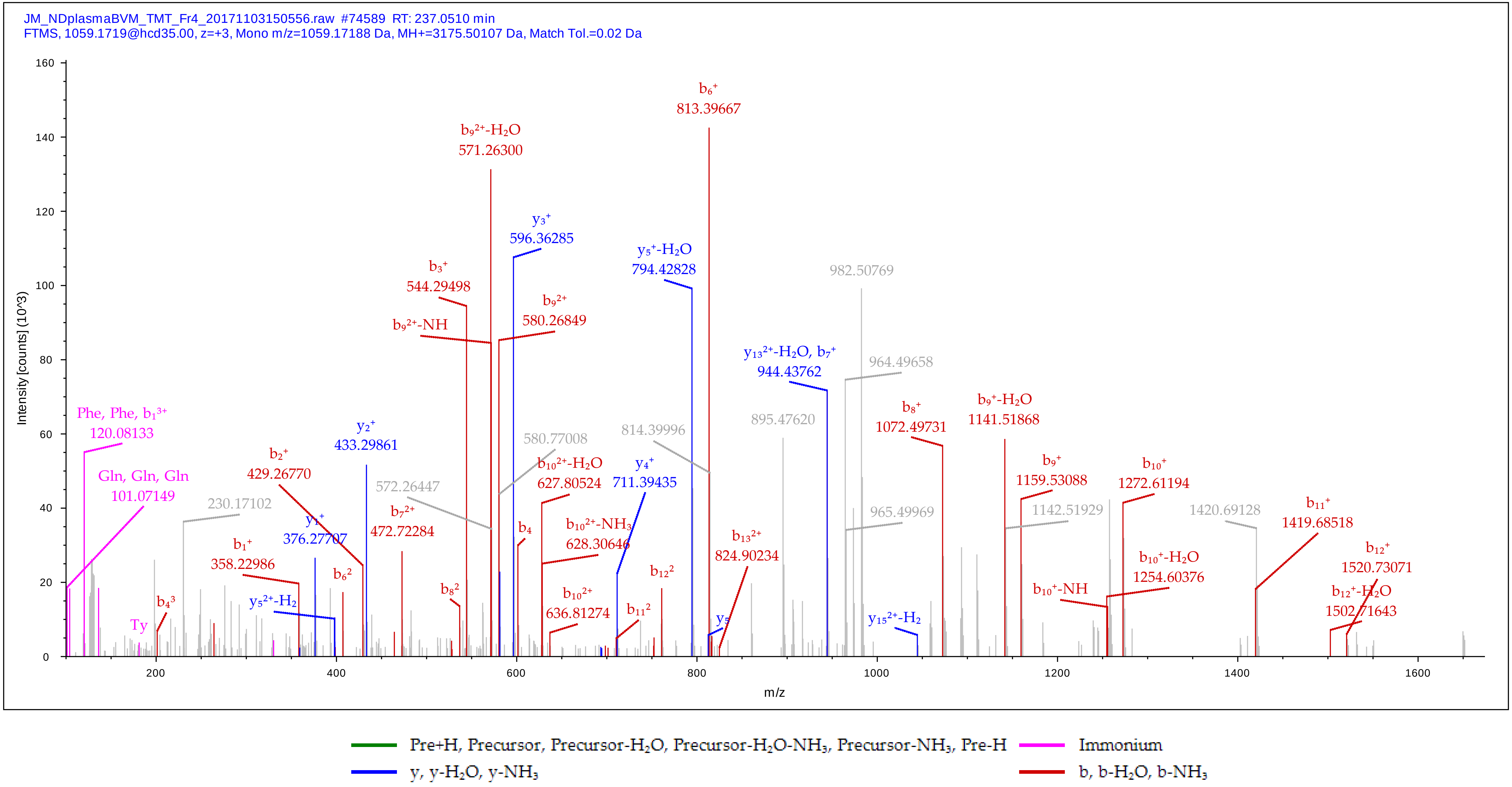
JM_NDplasmaBVM_TMT_Fr4_20171103150556.raw #74589 RT: 237.0510 min
FTMS, 1059.1719@hcd35.00, z=+3, Mono m/z=1059.17188 Da, MH+=3175.50107 Da, Match Tol.=0.02 Da
Intensity [counts] (10^3)
m/z
b6+ 813.39667
b9 2+-H2O 571.26300
y3+ 596.36285
y5+-H2O 794.42828
982.50769
b3+ 544.29498
b9 2+ 580.26849
b9 2+-NH
y13 2+-H2O, b7+ 944.43762
964.49658
Phe, Phe, b1 3+ 120.08133
y2+ 433.29861
895.47620
b8+ 1072.49731
b9+-H2O 1141.51868
580.77008
814.39996
b2+ 429.26770
b10 2+-H2O 627.80524
y4+ 711.39435
b9+ 1159.53088
b10+ 1272.61194
Gln, Gln, Gln 101.07149
572.26447
230.17102
b7 2+ 472.72284
965.49969
1142.51929
1420.69128
b11+ 1419.68518
y1+ 376.27707
b4
b10 2+-NH3 628.30646
b13 2+ 824.90234
b1+ 358.22986
b6 2
b12 2
b10+-H2O 1254.60376
b12+ 1520.73071
b8 2
b10+-NH
b12+-H2O 1502.71643
b4 3
y5 2+-H2
b10 2+ 636.81274
b11 2
Ty
y5
y15 2+-H2
Pre+H, Precursor, Precursor-H2O, Precursor-H2O-NH3, Precursor-NH3, Pre-H
Immonium
y, y-H2O, y-NH3
b, b-H2O, b-NH3

#1	Immonium	b^{+}	b^{2+}	b^{3+}	b^{4+}	Seq.	y^{+}	y^{2+}	y^{3+}	y^{4+}	#2
1	273.21241	301.20732	151.10730	101.07396	76.05729	A-TMT6plex					30
2	72.08078	400.27574	200.64151	134.09676	100.82439	V	3085.64896	1543.32812	1029.22117	772.16770	29
3	86.09643	513.35980	257.18354	171.79145	129.09541	L	2986.58055	1493.79391	996.19837	747.40059	28
4	74.06004	614.40748	307.70738	205.47401	154.35733	T	2873.49649	1437.25188	958.50368	719.12958	27
5	86.09643	727.49154	364.24941	243.16870	182.62834	I	2772.44881	1386.72804	924.82112	693.86766	26
6	88.03930	842.51849	421.76288	281.51101	211.38508	D	2659.36474	1330.18601	887.12643	665.59664	25
7	102.05496	971.56108	486.28418	324.52521	243.64573	E	2544.33780	1272.67254	848.78412	636.83991	24
8	74.06004	1072.60876	536.80802	358.20777	268.90765	T	2415.29521	1208.15124	805.76992	604.57926	23
9	30.03383	1129.63022	565.31875	377.21492	283.16301	G	2314.24753	1157.62740	772.08736	579.31734	22
10	74.06004	1230.67790	615.84259	410.89748	308.42493	T	2257.22607	1129.11667	753.08021	565.06197	21
11	102.05496	1359.72049	680.36388	453.91168	340.68558	E	2156.17839	1078.59283	719.39765	539.80005	20
12	44.04948	1430.75761	715.88244	477.59072	358.44486	A	2027.13579	1014.07154	676.38345	507.53941	19
13	44.04948	1501.79472	751.40100	501.26976	376.20414	A	1956.09868	978.55298	652.70441	489.78013	18
14	44.04948	1572.83183	786.91955	524.94880	393.96342	A	1885.06157	943.03442	629.02537	472.02085	17
15	72.08078	1671.90025	836.45376	557.97160	418.73052	V	1814.02445	907.51586	605.34634	454.26157	16
16	74.06004	1772.94793	886.97760	591.65416	443.99244	T	1714.95604	857.98166	572.32353	429.49447	15
17	72.08078	1872.01634	936.51181	624.67696	468.75954	V	1613.90836	807.45782	538.64097	404.23255	14
18	86.09643	1985.10040	993.05384	662.37165	497.03056	L	1514.83995	757.92361	505.61817	379.46544	13
19	86.09643	2098.18447	1049.59587	700.06634	525.30157	L	1401.75588	701.38158	467.92348	351.19443	12
20	44.04948	2169.22158	1085.11443	723.74538	543.06085	A	1288.67182	644.83955	430.22879	322.92341	11
21	72.08078	2268.28999	1134.64864	756.76818	567.82796	V	1217.63470	609.32099	406.54975	305.16413	10
22	70.06513	2365.34276	1183.17502	789.11910	592.09115	P	1118.56629	559.78678	373.52695	280.39703	9
23	181.06077	2573.39117	1287.19922	858.46857	644.10325	Y-Nitro	1021.51353	511.26040	341.17603	256.13384	8
24	60.04439	2660.42319	1330.71524	887.47925	665.86126	S	813.46512	407.23620	271.82656	204.12174	7
25	104.05285	2791.46368	1396.23548	931.15941	698.62138	M	726.43309	363.72018	242.81588	182.36373	6
26	70.06513	2888.51644	1444.76186	963.51033	722.88457	P	595.39261	298.19994	199.13572	149.60361	5
27	70.06513	2985.56921	1493.28824	995.86125	747.14776	P	498.33984	249.67356	166.78480	125.34042	4
28	86.09643	3098.65327	1549.83027	1033.55594	775.41877	I	401.28708	201.14718	134.43388	101.07723	3
29	86.09643	3211.73733	1606.37231	1071.25063	803.68979	L	288.20302	144.60515	96.73919	72.80621	2
30	129.11347					R	175.11895	88.06311	59.04450	44.53520	1

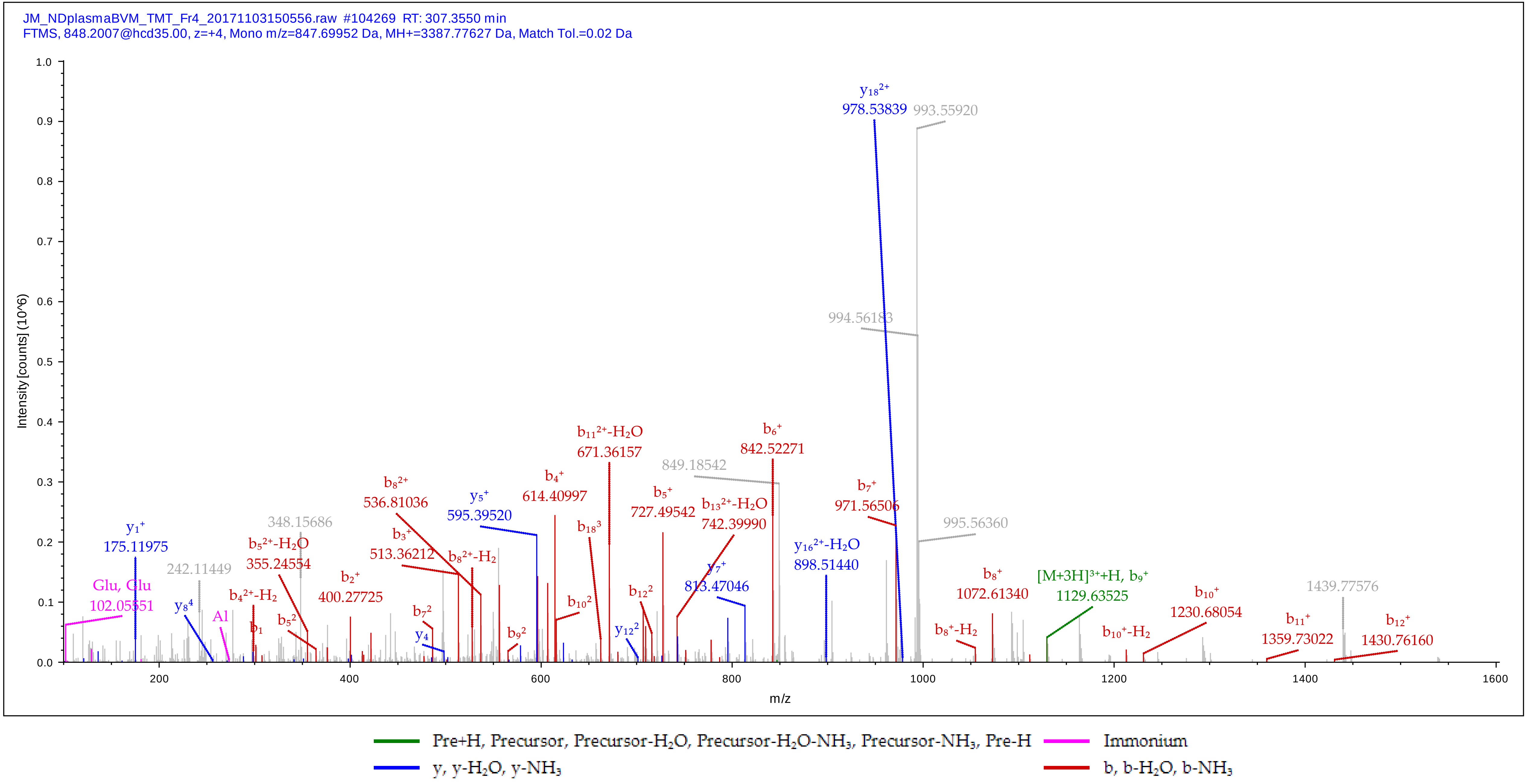

JM_NDplasmaBVM_TMT_Fr4_20171103150556.raw #104269 RT: 307.3550 min
FTMS, 848.2007@hcd35.00, z=+4, Mono m/z=847.69952 Da, MH+=3387.77627 Da, Match Tol.=0.02 Da
Intensity [counts] (10^6)
m/z
978.53839
993.55920
994.56183
995.56360
849.18542
842.52271
671.36157
614.40997
595.39520
536.81036
513.36212
727.49542
742.39990
971.56506
898.51440
813.47046
1072.61340
1129.63525
1230.68054
1359.73022
1430.76160
1439.77576
348.15686
242.11449
175.11975
355.24554
400.27725
102.05551
Glu, Glu
Al
Pre+H, Precursor, Precursor-H2O, Precursor-H2O-NH3, Precursor-NH3, Pre-H
Immonium
y, y-H2O, y-NH3
b, b-H2O, b-NH3

#1	Immonium	b⁺	b^{2+}	b^{3+}	Seq.	y⁺	y^{2+}	y^{3+}	#2
1	273.21241	301.20732	151.10730	101.07396	A-TMT6plex				30
2	72.08078	400.27574	200.64151	134.09676	V	3078.56636	1539.78682	1026.86030	29
3	86.09643	513.35980	257.18354	171.79145	L	2979.49794	1490.25261	993.83750	28
4	74.06004	614.40748	307.70738	205.47401	T	2866.41388	1433.71058	956.14281	27
5	86.09643	727.49154	364.24941	243.16870	I	2765.36620	1383.18674	922.46025	26
6	88.03930	842.51849	421.76288	281.51101	D	2652.28214	1326.64471	884.76556	25
7	102.05496	971.56108	486.28418	324.52521	E	2537.25519	1269.13123	846.42325	24
8	74.06004	1072.60876	536.80802	358.20777	T	2408.21260	1204.60994	803.40905	23
9	30.03383	1129.63022	565.31875	377.21492	G	2307.16492	1154.08610	769.72649	22
10	74.06004	1230.67790	615.84259	410.89748	T	2250.14346	1125.57537	750.71934	21
11	102.05496	1359.72049	680.36388	453.91168	E	2149.09578	1075.05153	717.03678	20
12	44.04948	1430.75761	715.88244	477.59072	A	2020.05319	1010.53023	674.02258	19
13	44.04948	1501.79472	751.40100	501.26976	A	1949.01607	975.01167	650.34354	18
14	44.04948	1572.83183	786.91955	524.94880	A	1877.97896	939.49312	626.66450	17
15	44.04948	1643.86895	822.43811	548.62783	A	1806.94185	903.97456	602.98547	16
16	74.06004	1744.91663	872.96195	582.31039	T	1735.90473	868.45600	579.30643	15
17	72.08078	1843.98504	922.49616	615.33320	V	1634.85705	817.93216	545.62387	14
18	86.09643	1957.06910	979.03819	653.02789	L	1535.78864	768.39796	512.60106	13
19	101.07094	2085.12768	1043.06748	695.71408	Q	1422.70458	711.85593	474.90638	12
20	72.08078	2184.19609	1092.60169	728.73688	V	1294.64600	647.82664	432.22018	11
21	44.04948	2255.23321	1128.12024	752.41592	A	1195.57758	598.29243	399.19738	10
22	74.06004	2356.28089	1178.64408	786.09848	T	1124.54047	562.77387	375.51834	9
23	181.06077	2564.32929	1282.66828	855.44795	Y-Nitro	1023.49279	512.25003	341.83578	8
24	60.04439	2651.36132	1326.18430	884.45862	S	815.44439	408.22583	272.48631	7
25	120.04776	2798.39672	1399.70200	933.47042	M-Oxidation	728.41236	364.70982	243.47564	6
26	70.06513	2895.44948	1448.22838	965.82135	P	581.37696	291.19212	194.46384	5
27	70.06513	2992.50225	1496.75476	998.17227	P	484.32419	242.66574	162.11292	4
28	86.09643	3105.58631	1553.29679	1035.86696	I	387.27143	194.13935	129.76199	3
29	72.08078	3204.65473	1602.83100	1068.88976	V	274.18737	137.59732	92.06731	2
30	129.11347				R	175.11895	88.06311	59.04450	1

Nitro-Tyr immonium ion is detected in MS/MS spectra and added brown colored in the following spectrum

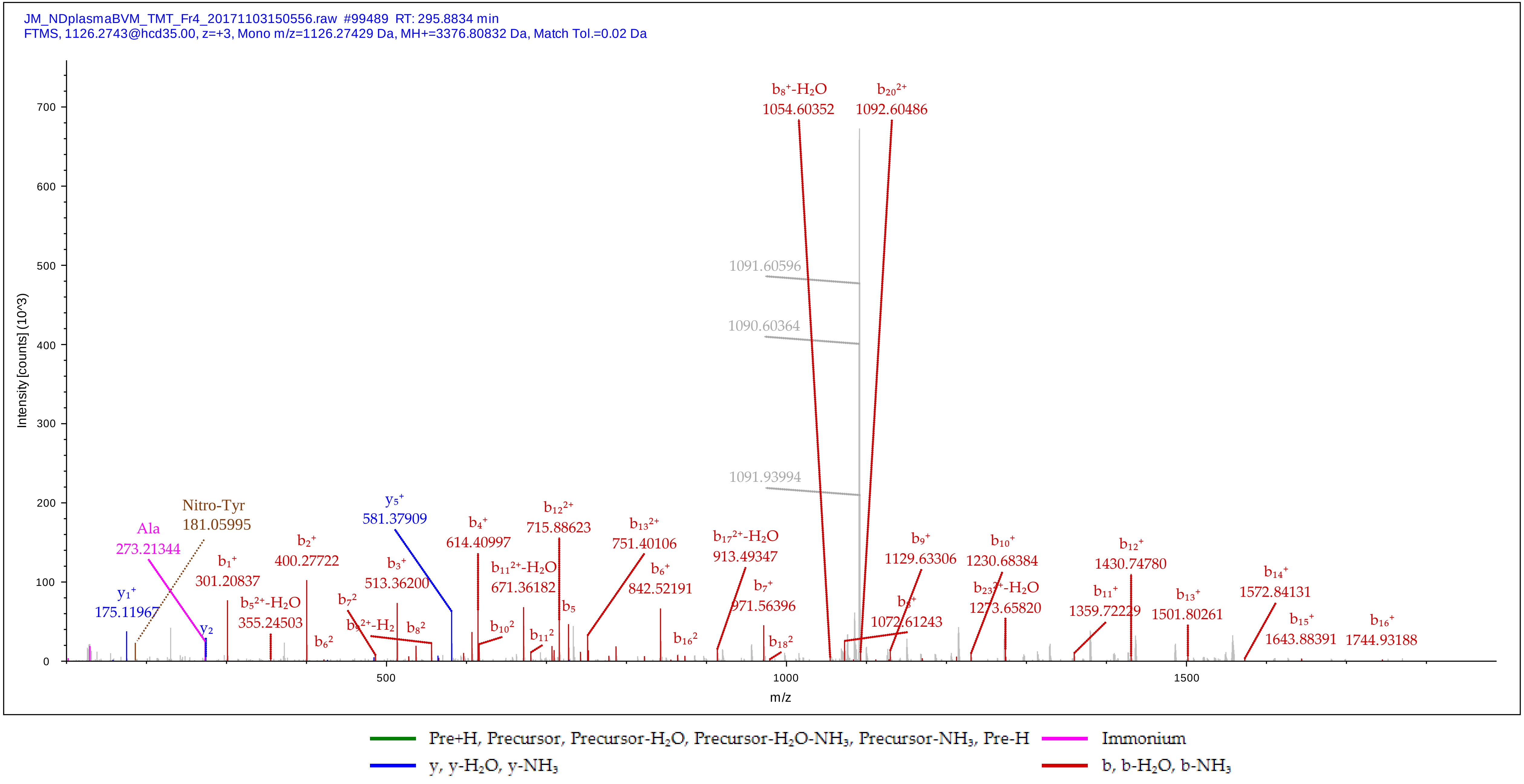
JM_NDplasmaBVM_TMT_Fr4_20171103150556.raw #99489 RT: 295.8834 min
FTMS, 1126.2743@hcd35.00, z=+3, Mono m/z=1126.27429 Da, MH+=3376.80832 Da, Match Tol.=0.02 Da
Intensity [counts] (10^3)
m/z
b8+-H2O 1054.60352
b20 2+ 1092.60486
1091.60596
1090.60364
1091.93994
Nitro-Tyr 181.05995
Ala 273.21344
y1+ 175.11967
y2
b1+ 301.20837
b2+ 400.27722
b5 2+-H2O 355.24503
y5+ 581.37909
b3+ 513.36200
b4+ 614.40997
b12 2+ 715.88623
b11 2+-H2O 671.36182
b13 2+ 751.40106
b6+ 842.52191
b17 2+-H2O 913.49347
b7+ 971.56396
b9+ 1129.63306
b10+ 1230.68384
b23 2+-H2O 1273.65820
b12+ 1430.74780
b11+ 1359.72229
b13+ 1501.80261
b14+ 1572.84131
b15+ 1643.88391
b16+ 1744.93188
1072.61243
Pre+H, Precursor, Precursor-H2O, Precursor-H2O-NH3, Precursor-NH3, Pre-H
Immonium
y, y-H2O, y-NH3
b, b-H2O, b-NH3

#1	Immonium	b^{+}	b^{2+}	b^{3+}	b^{4+}	b^{5+}	Seq.	y^{+}	y^{2+}	y^{3+}	y^{4+}	y^{5+}	#2
1	365.23862	393.23354	197.12041	131.74936	99.06384	79.45253	Y-TMT6plex						27
2	102.05496	522.27613	261.64170	174.76356	131.32449	105.26105	E	3883.96358	1942.48543	1295.32604	971.74635	777.59854	26
3	133.04301	682.30678	341.65703	228.10711	171.33215	137.26718	C-Carbamidomethyl	3754.92098	1877.96413	1252.31185	939.48570	751.79002	25
4	87.05529	796.34971	398.67849	266.12142	199.84288	160.07576	N	3594.89034	1797.94881	1198.96830	899.47804	719.78389	24
5	330.27026	1153.60760	577.30744	385.20738	289.15736	231.52734	K-TMT6plex	3480.84741	1740.92734	1160.95399	870.96731	696.97530	23
6	70.06513	1250.66036	625.83382	417.55831	313.42055	250.93789	P	3123.58951	1562.29840	1041.86802	781.65284	625.52372	22
7	86.09643	1363.74443	682.37585	455.25299	341.69156	273.55471	L	3026.53675	1513.77201	1009.51710	757.38964	606.11317	21
8	102.05496	1492.78702	746.89715	498.26719	373.95221	299.36323	E	2913.45269	1457.22998	971.82241	729.11863	583.49636	20
9	86.09643	1605.87109	803.43918	535.96188	402.22323	321.98004	L	2784.41009	1392.70868	928.80822	696.85798	557.68784	19
10	120.08078	1752.93950	876.97339	584.98468	438.99033	351.39372	F	2671.32603	1336.16665	891.11353	668.58696	535.07103	18
11	30.03383	1809.96096	905.48412	603.99184	453.24570	362.79801	G	2524.25762	1262.63245	842.09072	631.81986	505.65734	17
12	101.07094	1938.01954	969.51341	646.67803	485.26034	388.40973	Q	2467.23615	1234.12171	823.08357	617.56450	494.25305	16
13	72.08078	2037.08795	1019.04762	679.70084	510.02745	408.22341	V	2339.17757	1170.09243	780.39738	585.54985	468.64134	15
14	102.05496	2166.13055	1083.56891	722.71503	542.28809	434.03193	E	2240.10916	1120.55822	747.37457	560.78275	448.82765	14
15	72.08078	2265.19896	1133.10312	755.73784	567.05520	453.84561	V	2111.06657	1056.03692	704.36037	528.52210	423.01913	13
16	120.04776	2412.23436	1206.62082	804.74964	603.81405	483.25269	M-Oxidation	2011.99815	1006.50272	671.33757	503.75500	403.20545	12
17	133.04301	2572.26501	1286.63614	858.09319	643.82171	515.25882	C-Carbamidomethyl	1864.96275	932.98502	622.32577	466.99615	373.79837	11
18	102.05496	2701.30760	1351.15744	901.10739	676.08236	541.06734	E	1704.93211	852.96969	568.98222	426.98848	341.79224	10
19	87.05529	2815.35053	1408.17890	939.12169	704.59309	563.87593	N	1575.88951	788.44839	525.96802	394.72784	315.98372	9
20	30.03383	2872.37199	1436.68964	958.12885	718.84846	575.28022	G	1461.84658	731.42693	487.95371	366.21710	293.17514	8
21	86.09643	2985.45606	1493.23167	995.82354	747.11947	597.89703	I	1404.82512	702.91620	468.94656	351.96174	281.77085	7
22	204.07675	3216.52045	1608.76386	1072.84500	804.88557	644.10991	W-Nitro	1291.74106	646.37417	431.25187	323.69072	259.15403	6
23	74.06004	3317.56813	1659.28770	1106.52756	830.14749	664.31945	T	1060.67667	530.84197	354.23041	265.92462	212.94115	5
24	102.05496	3446.61072	1723.80900	1149.54176	862.40814	690.12797	E	959.62899	480.31813	320.54785	240.66270	192.73162	4
25	330.27026	3803.86861	1902.43795	1268.62772	951.72261	761.57954	K-TMT6plex	830.58640	415.79684	277.53365	208.40206	166.92310	3
26	70.06513	3900.92138	1950.96433	1300.97864	975.98580	780.99010	P	473.32850	237.16789	158.44768	119.08758	95.47152	2
27	330.27026						K-TMT6plex	376.27574	188.64151	126.09676	94.82439	76.06097	1

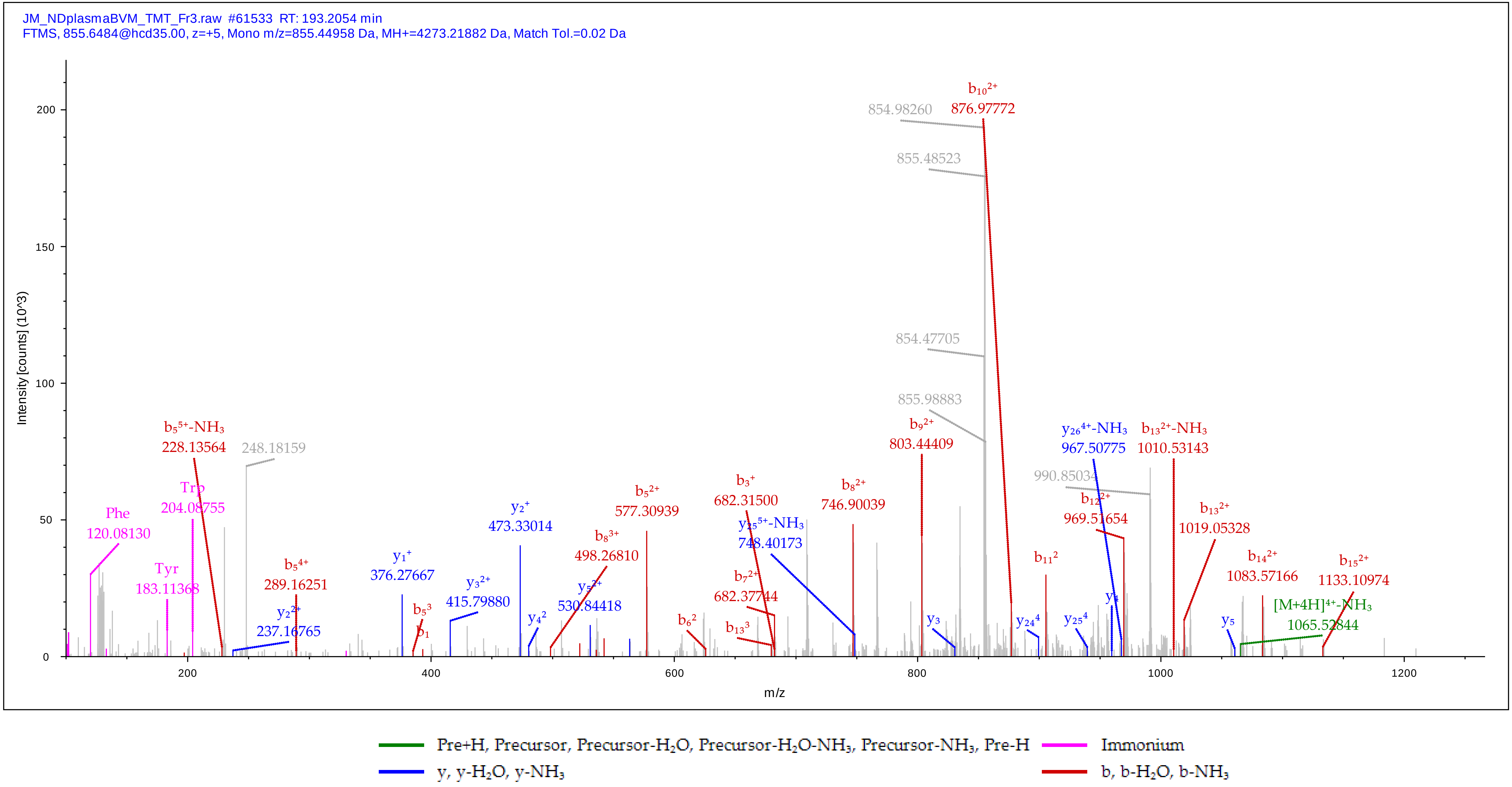

JM_NDplasmaBVM_TMT_Fr3.raw #61533 RT: 193.2054 min
FTMS, 855.6484@hcd35.00, z=+5, Mono m/z=855.44958 Da, MH+=4273.21882 Da, Match Tol.=0.02 Da
Intensity [counts] (10^3)
m/z
0
50
100
150
200
200
400
600
800
1000
1200
b10 2+ 876.97772
854.98260
855.48523
854.47705
855.98883
b9 2+ 803.44409
b5 5+-NH3 228.13564
248.18159
Phe 120.08130
Trp 204.08755
Tyr 183.11368
b5 4+ 289.16251
y2 2+ 237.16765
y1+ 376.27667
y2+ 473.33014
y3 2+ 415.79880
y4 2
b5 3
b1
b8 3+ 498.26810
y5 2+ 530.84418
b5 2+ 577.30939
b6 2
b3+ 682.31500
y25 5+-NH3 748.40173
b7 2+ 682.37744
b13 3
b8 2+ 746.90039
y3
y24 4
y25 4
y26 4+-NH3 967.50775
b13 2+-NH3 1010.53143
990.85034
b12 2+ 969.51654
b11 2
y4
b13 2+ 1019.05328
b14 2+ 1083.57166
b15 2+ 1133.10974
y5
[M+4H]4+-NH3 1065.52844
Pre+H, Precursor, Precursor-H2O, Precursor-H2O-NH3, Precursor-NH3, Pre-H
Immonium
y, y-H2O, y-NH3
b, b-H2O, b-NH3

#1	Immonium	b^{+}	b^{2+}	b^{3+}	b^{4+}	b^{5+}	Seq.	y^{+}	y^{2+}	y^{3+}	y^{4+}	y^{5+}	#2
1	339.23421	367.22912	184.11820	123.08122	92.56274	74.25165	H-TMT6plex						35
2	86.09643	480.31318	240.66023	160.77591	120.83375	96.86846	L	4120.00270	2060.50499	1374.00575	1030.75613	824.80636	34
3	86.09643	593.39725	297.20226	198.47060	149.10477	119.48527	I	4006.91864	2003.96296	1336.31106	1002.48512	802.18955	33
4	72.08078	692.46566	346.73647	231.49341	173.87187	139.29895	V	3893.83457	1947.42093	1298.61638	974.21410	779.57274	32
5	74.06004	793.51334	397.26031	265.17596	199.13379	159.50849	T	3794.76616	1897.88672	1265.59357	949.44700	759.75905	31
6	70.06513	890.56610	445.78669	297.52689	223.39698	178.91904	P	3693.71848	1847.36288	1231.91101	924.18508	739.54952	30
7	44.04948	961.60322	481.30525	321.20592	241.15626	193.12646	A	3596.66572	1798.83650	1199.56009	899.92189	720.13897	29
8	30.03383	1018.62468	509.81598	340.21308	255.41163	204.53076	G	3525.62861	1763.31794	1175.88105	882.16261	705.93154	28
9	133.04301	1178.65533	589.83130	393.55663	295.41929	236.53689	Carbamidometl	3468.60714	1734.80721	1156.87390	867.90724	694.52725	27
10	30.03383	1235.67679	618.34204	412.56378	309.67466	247.94118	G	3308.57649	1654.79188	1103.53035	827.89958	662.52112	26
11	102.05496	1364.71939	682.86333	455.57798	341.93530	273.74970	E	3251.55503	1626.28115	1084.52319	813.64421	651.11683	25
12	101.07094	1492.77796	746.89262	498.26417	373.94995	299.36141	Q	3122.51244	1561.75986	1041.50900	781.38357	625.30831	24
13	87.05529	1606.82089	803.91408	536.27848	402.46068	322.17000	N	2994.45386	1497.73057	998.82280	749.36892	599.69659	23
14	104.05285	1737.86138	869.43433	579.95864	435.22080	348.37810	M	2880.41093	1440.70910	960.80849	720.85819	576.88801	22
15	86.09643	1850.94544	925.97636	617.65333	463.49182	370.99491	I	2749.37045	1375.18886	917.12833	688.09807	550.67991	21
16	30.03383	1907.96690	954.48709	636.66049	477.74718	382.39920	G	2636.28638	1318.64683	879.43365	659.82705	528.06310	20
17	120.04776	2055.00230	1028.00479	685.67229	514.50603	411.80628	M-Oxidation	2579.26492	1290.13610	860.42649	645.57169	516.65881	19
18	74.06004	2156.04998	1078.52863	719.35485	539.76795	432.01582	T	2432.22952	1216.61840	811.41469	608.81284	487.25173	18
19	70.06513	2253.10275	1127.05501	751.70577	564.03114	451.42637	P	2331.18184	1166.09456	777.73213	583.55092	467.04219	17
20	74.06004	2354.15042	1177.57885	785.38833	589.29306	471.63591	T	2234.12908	1117.56818	745.38121	559.28773	447.63164	16
21	72.08078	2453.21884	1227.11306	818.41113	614.06017	491.44959	V	2133.08140	1067.04434	711.69865	534.02581	427.42210	15
22	86.09643	2566.30290	1283.65509	856.10582	642.33118	514.06640	I	2034.01299	1017.51013	678.67585	509.25870	407.60842	14
23	44.04948	2637.34002	1319.17365	879.78486	660.09046	528.27382	A	1920.92892	960.96810	640.98116	480.98769	384.99161	13
24	72.08078	2736.40843	1368.70785	912.80766	684.85756	548.08751	V	1849.89181	925.44954	617.30212	463.22841	370.78418	12
25	110.07127	2873.46734	1437.23731	958.49396	719.12229	575.49929	H	1750.82339	875.91534	584.27932	438.46131	350.97050	11
26	181.06077	3081.51575	1541.26151	1027.84343	771.13439	617.10897	Y-Nitro	1613.76448	807.38588	538.59301	404.19658	323.55872	10
27	86.09643	3194.59981	1597.80354	1065.53812	799.40541	639.72578	L	1405.71608	703.36168	469.24354	352.18448	281.94904	9
28	88.03930	3309.62675	1655.31702	1103.88044	828.16215	662.73117	D	1292.63201	646.81964	431.54885	323.91346	259.33222	8
29	101.07094	3437.68533	1719.34630	1146.56663	860.17679	688.34289	Q	1177.60507	589.30617	393.20654	295.15672	236.32683	7
30	74.06004	3538.73301	1769.87014	1180.24919	885.43871	708.55242	T	1049.54649	525.27688	350.52035	263.14208	210.71512	6
31	102.05496	3667.77560	1834.39144	1223.26339	917.69936	734.36094	E	948.49881	474.75304	316.83779	237.88016	190.50558	5
32	101.07094	3795.83418	1898.42073	1265.94958	949.71400	759.97266	Q	819.45622	410.23175	273.82359	205.61951	164.69707	4
33	159.09167	3981.91349	1991.46039	1327.97602	996.23383	797.18852	W	691.39764	346.20246	231.13740	173.60487	139.08535	3
34	102.05496	4110.95609	2055.98168	1370.99021	1028.49448	822.99704	E	505.31833	253.16280	169.11096	127.08504	101.86949	2
35	330.27026						K-TMT6plex	376.27574	188.64151	126.09676	94.82439	76.06097	1

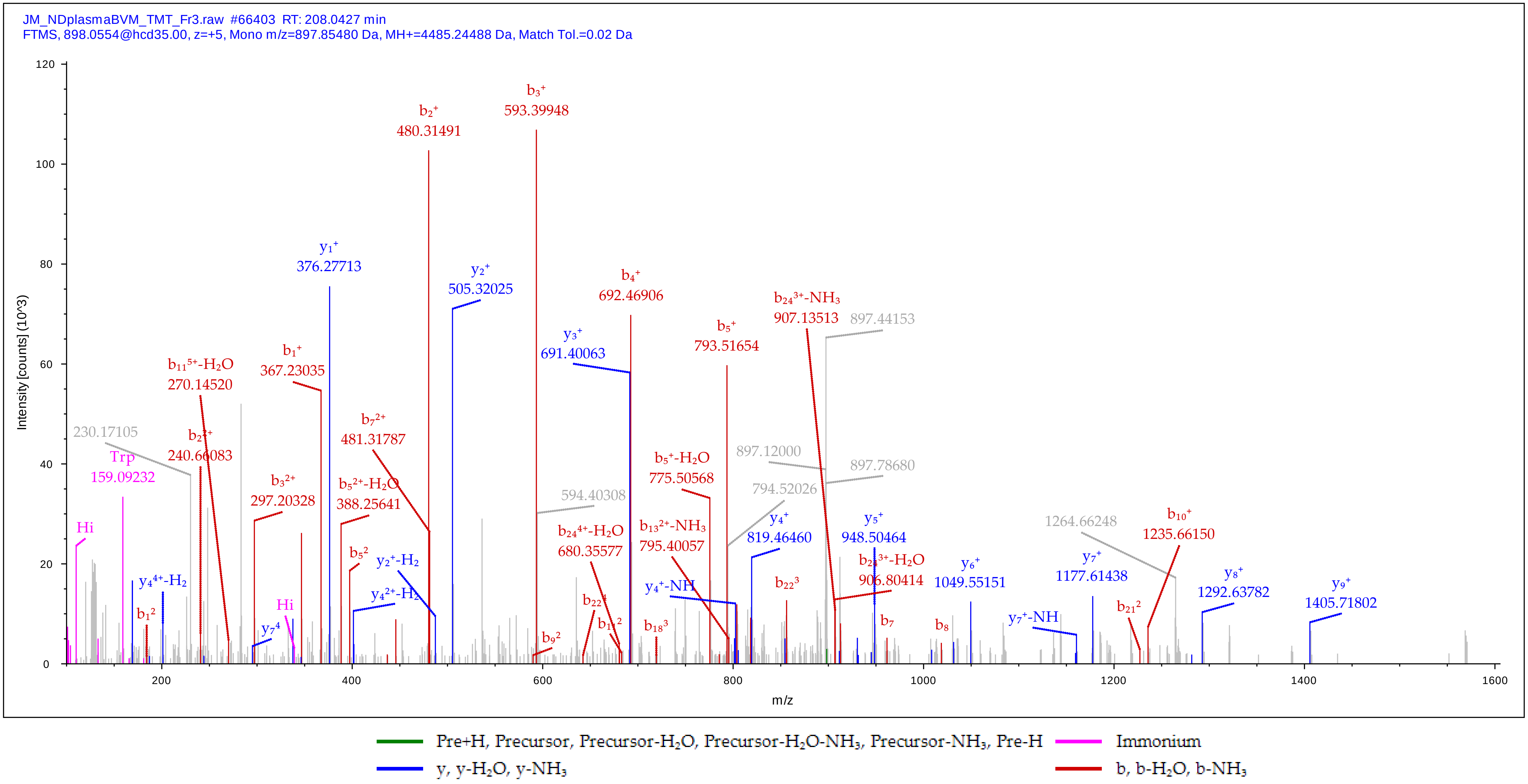

JM_NDplasmaBVM_TMT_Fr3.raw #66403 RT: 208.0427 min
FTMS, 898.0554@hcd35.00, z=+5, Mono m/z=897.85480 Da, MH+=4485.24488 Da, Match Tol.=0.02 Da
Intensity [counts] (10^3)
m/z
b2+ 480.31491
b3+ 593.39948
y1+ 376.27713
y2+ 505.32025
b4+ 692.46906
b24 3+-NH3 907.13513
897.44153
b5+ 793.51654
y3+ 691.40063
b1+ 367.23035
b11 5+-H2O 270.14520
230.17105
b2 2+ 240.66083
Trp 159.09232
b7 2+ 481.31787
b5+-H2O 775.50568
897.12000
897.78680
794.52026
594.40308
b3 2+ 297.20328
b5 2+-H2O 388.25641
Hi
b24 4+-H2O 680.35577
b13 2+-NH3 795.40057
y4+ 819.46460
y5+ 948.50464
1264.66248
b10+ 1235.66150
b24 3+-H2O 906.80414
y6+ 1049.55151
y7+ 1177.61438
y8+ 1292.63782
y9+ 1405.71802
y4 4+-H2
y2+-H2
y4 2+-H2
y4+-NH
y7+-NH
y7 4
b1 2
b5 2
b9 2
b22 3
b18 3
b21 2
b7
b8
Pre+H, Precursor, Precursor-H2O, Precursor-H2O-NH3, Precursor-NH3, Pre-H
Immonium
y, y-H2O, y-NH3
b, b-H2O, b-NH3

#1	Immonium	b^{+}	b^{2+}	b^{3+}	b^{4+}	Seq.	y^{+}	y^{2+}	y^{3+}	y^{4+}	#2
1	559.43319	587.42810	294.21769	196.48089	147.61248	K-TMT6plex-TMT6plex					21
2	30.03383	644.44957	322.72842	215.48804	161.86785	G	2559.25983	1280.13356	853.75813	640.57042	20
3	30.03383	701.47103	351.23915	234.49519	176.12322	G	2502.23837	1251.62282	834.75097	626.31505	19
4	102.05496	830.51362	415.76045	277.50939	208.38386	E	2445.21691	1223.11209	815.74382	612.05968	18
5	74.06004	931.56130	466.28429	311.19195	233.64578	T	2316.17431	1158.59079	772.72962	579.79904	17
6	60.04439	1018.59333	509.80030	340.20263	255.40379	S	2215.12663	1108.06696	739.04706	554.53712	16
7	102.05496	1147.63592	574.32160	383.21683	287.66444	E	2128.09461	1064.55094	710.03639	532.77911	15
8	104.05285	1278.67641	639.84184	426.89699	320.42456	M	1999.05201	1000.02964	667.02219	500.51846	14
9	181.06077	1486.72481	743.86605	496.24646	372.43666	Y-Nitro	1868.01153	934.50940	623.34203	467.75834	13
10	86.09643	1599.80888	800.40808	533.94114	400.70768	L	1659.96312	830.48520	553.99256	415.74624	12
11	86.09643	1712.89294	856.95011	571.63583	428.97869	I	1546.87906	773.94317	516.29787	387.47522	11
12	101.07094	1840.95152	920.97940	614.32202	460.99334	Q	1433.79499	717.40114	478.60318	359.20421	10
13	70.06513	1938.00428	969.50578	646.67295	485.25653	P	1305.73642	653.37185	435.91699	327.18956	9
14	88.03930	2053.03123	1027.01925	685.01526	514.01326	D	1208.68365	604.84546	403.56607	302.92637	8
15	74.06004	2154.07891	1077.54309	718.69782	539.27518	T	1093.65671	547.33199	365.22375	274.16964	7
16	60.04439	2241.11093	1121.05911	747.70850	561.03319	S	992.60903	496.80815	331.54120	248.90772	6
17	86.09643	2354.19500	1177.60114	785.40318	589.30421	I	905.57700	453.29214	302.53052	227.14971	5
18	330.27026	2711.45289	1356.23008	904.48915	678.61868	K-TMT6plex	792.49294	396.75011	264.83583	198.87869	4
19	70.06513	2808.50566	1404.75647	936.84007	702.88187	P	435.23504	218.12116	145.74987	109.56422	3
20	136.07569	2971.56898	1486.28813	991.19451	743.64770	Y	338.18228	169.59478	113.39894	85.30103	2
21	129.11347					R	175.11895	88.06311	59.04450	44.53520	1

Nitro-Tyr immonium ion is detected in MS/MS spectra and added brown colored in the following spectrum

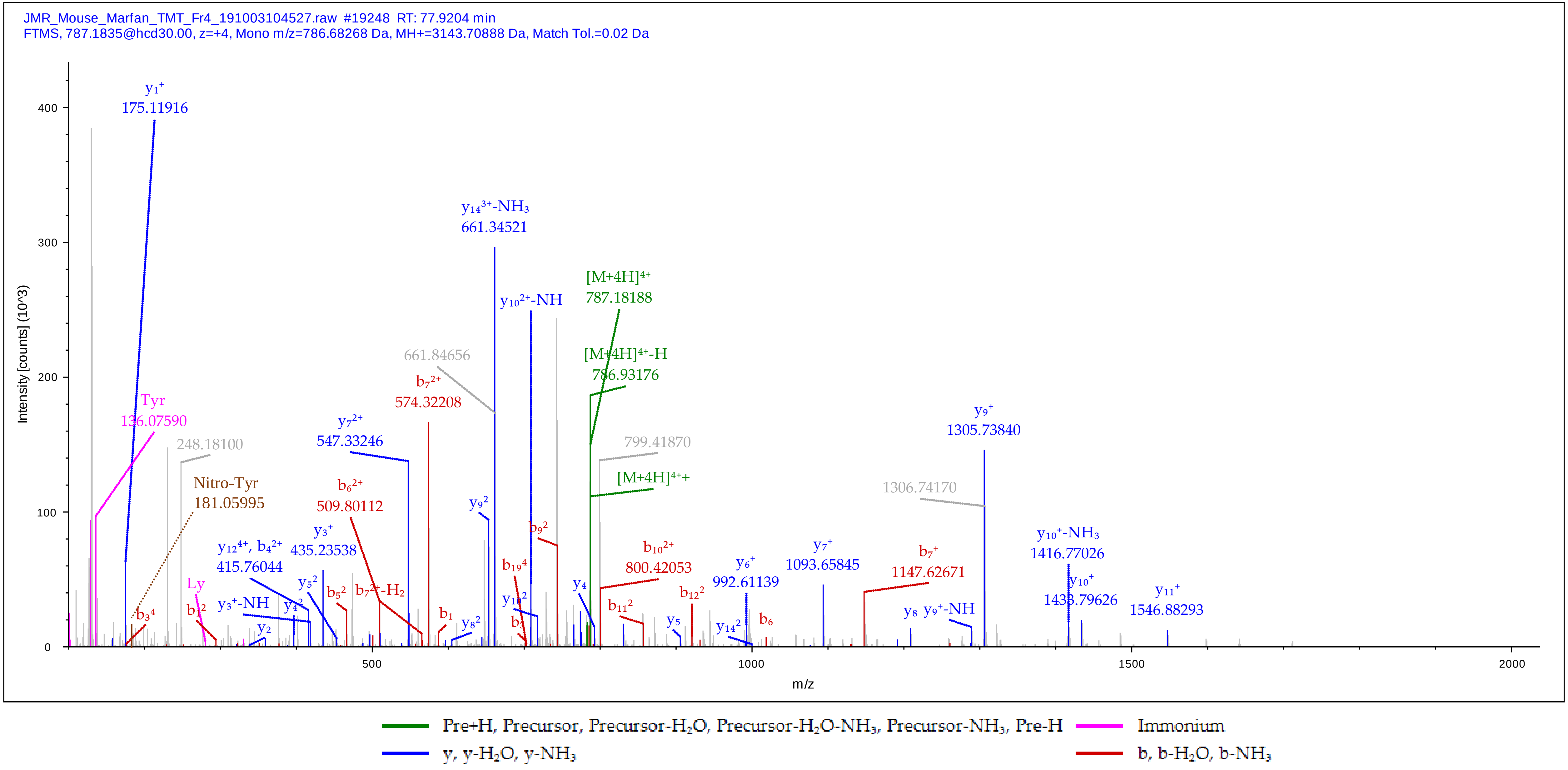
JMR_Mouse_Marfan_TMT_Fr4_191003104527.raw #19248 RT: 77.9204 min
FTMS, 787.1835@hcd30.00, z=+4, Mono m/z=786.68268 Da, MH+=3143.70888 Da, Match Tol.=0.02 Da
Intensity [counts] (10^3)
m/z
y1+ 175.11916
Tyr 136.07590
248.18100
Nitro-Tyr 181.05995
y12 4+, b4 2+ 415.76044
y3+ 435.23538
y7 2+ 547.33246
b6 2+ 509.80112
b7 2+ 574.32208
661.84656
y14 3+-NH3 661.34521
y10 2+-NH
[M+4H]4+ 787.18188
[M+4H]4+-H 786.93176
799.41870
[M+4H]4++
b10 2+ 800.42053
y6+ 992.61139
y7+ 1093.65845
b7+ 1147.62671
1306.74170
y9+ 1305.73840
y10+-NH3 1416.77026
y10+ 1433.79626
y11+ 1546.88293
Pre+H, Precursor, Precursor-H2O, Precursor-H2O-NH3, Precursor-NH3, Pre-H
Immonium
y, y-H2O, y-NH3
b, b-H2O, b-NH3

#1	Immonium	b^{+}	b^{2+}	b^{3+}	b^{4+}	b^{5+}	Seq.	y^{+}	y^{2+}	y^{3+}	y^{4+}	y^{5+}	#2
1	273.21241	301.20732	151.10730	101.07396	76.05729	61.04729	A-TMT6plex						30
2	72.08078	400.27574	200.64151	134.09676	100.82439	80.86097	V	3833.01955	1917.01341	1278.34470	959.01034	767.40973	29
3	88.03930	515.30268	258.15498	172.43908	129.58113	103.86636	D	3733.95113	1867.47920	1245.32190	934.24324	747.59605	28
4	101.07094	643.36126	322.18427	215.12527	161.59577	129.47807	Q	3618.92419	1809.96573	1206.97958	905.48650	724.59066	27
5	60.04439	730.39328	365.70028	244.13595	183.35378	146.88448	S	3490.86561	1745.93644	1164.29339	873.47186	698.97894	26
6	72.08078	829.46170	415.23449	277.15875	208.12088	166.69816	V	3403.83358	1702.42043	1135.28271	851.71385	681.57254	25
7	86.09643	942.54576	471.77652	314.85344	236.39190	189.31497	L	3304.76517	1652.88622	1102.25991	826.94675	661.75886	24
8	86.09643	1055.62983	528.31855	352.54813	264.66291	211.93179	L	3191.68111	1596.34419	1064.56522	798.67573	639.14204	23
9	86.09643	1168.71389	584.86058	390.24281	292.93393	234.54860	L	3078.59704	1539.80216	1026.87053	770.40472	616.52523	22
10	330.27026	1525.97179	763.48953	509.32878	382.24840	306.00018	K-TMT6plex	2965.51298	1483.26013	989.17584	742.13370	593.90842	21
11	70.06513	1623.02455	812.01591	541.67970	406.51159	325.41073	P	2608.25508	1304.63118	870.08988	652.81923	522.45684	20
12	102.05496	1752.06714	876.53721	584.69390	438.77224	351.21925	E	2511.20232	1256.10480	837.73896	628.55604	503.04629	19
13	60.04439	1839.09917	920.05322	613.70457	460.53025	368.62566	S	2382.15973	1191.58350	794.72476	596.29539	477.23777	18
14	102.05496	1968.14176	984.57452	656.71877	492.79090	394.43417	E	2295.12770	1148.06749	765.71408	574.53738	459.83136	17
15	86.09643	2081.22583	1041.11655	694.41346	521.06191	417.05099	L	2166.08510	1083.54619	722.69989	542.27673	434.02284	16
16	60.04439	2168.25786	1084.63257	723.42414	542.81992	434.45739	S	2053.00104	1027.00416	685.00520	514.00572	411.40603	15
17	70.06513	2265.31062	1133.15895	755.77506	567.08311	453.86795	P	1965.96901	983.48814	655.99452	492.24771	393.99962	14
18	60.04439	2352.34265	1176.67496	784.78573	588.84112	471.27435	S	1868.91625	934.96176	623.64360	467.98452	374.58907	13
19	159.09167	2538.42196	1269.71462	846.81217	635.36095	508.49021	W	1781.88422	891.44575	594.63292	446.22651	357.18267	12
20	86.09643	2651.50603	1326.25665	884.50686	663.63196	531.10703	I	1595.80491	798.40609	532.60649	399.70668	319.96680	11
21	181.06077	2859.55443	1430.28085	953.85633	715.64407	572.71671	Y-Nitro	1482.72084	741.86406	494.91180	371.43567	297.34999	10
22	87.05529	2973.59736	1487.30232	991.87064	744.15480	595.52529	N	1274.67244	637.83986	425.56233	319.42357	255.74031	9
23	86.09643	3086.68142	1543.84435	1029.56533	772.42581	618.14211	L	1160.62951	580.81839	387.54802	290.91283	232.93172	8
24	70.06513	3183.73419	1592.37073	1061.91625	796.68900	637.55266	P	1047.54545	524.27636	349.85333	262.64182	210.31491	7
25	30.03383	3240.75565	1620.88146	1080.92340	810.94437	648.95695	G	950.49268	475.74998	317.50241	238.37863	190.90436	6
26	120.04776	3387.79105	1694.39916	1129.93520	847.70322	678.36403	M-Oxidation	893.47122	447.23925	298.49526	224.12326	179.50006	5
27	101.07094	3515.84963	1758.42845	1172.62139	879.71786	703.97575	Q	746.43582	373.72155	249.48346	187.36441	150.09299	4
28	101.07094	3643.90820	1822.45774	1215.30759	911.73251	729.58746	Q	618.37724	309.69226	206.79726	155.34977	124.48127	3
29	87.05529	3757.95113	1879.47920	1253.32190	940.24324	752.39605	N	490.31866	245.66297	164.11107	123.33512	98.86955	2
30	330.27026						K-TMT6plex	376.27574	188.64151	126.09676	94.82439	76.06097	1

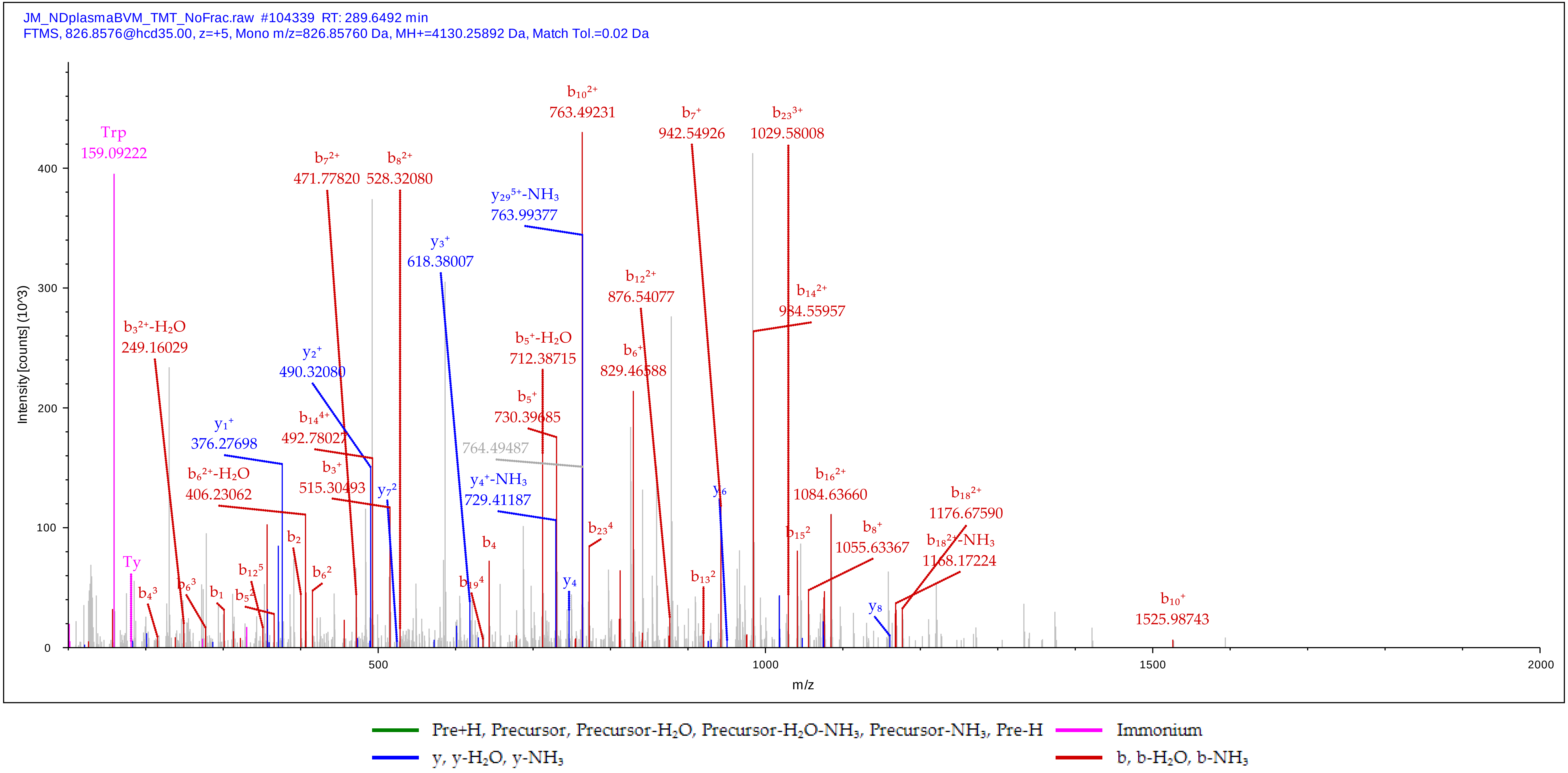

JM_NDplasmaBVM_TMT_NoFrac.raw #104339 RT: 289.6492 min
FTMS, 826.8576@hcd35.00, z=+5, Mono m/z=826.85760 Da, MH+=4130.25892 Da, Match Tol.=0.02 Da
Intensity [counts] (10^3)
m/z
Trp 159.09222
b10 2+ 763.49231
b7+ 942.54926
b23 3+ 1029.58008
b7 2+ 471.77820
b8 2+ 528.32080
y29 5+-NH3 763.99377
y3+ 618.38007
b12 2+ 876.54077
b14 2+ 984.55957
b3 2+-H2O 249.16029
y2+ 490.32080
b5+-H2O 712.38715
b6+ 829.46588
b5+ 730.39685
y1+ 376.27698
b14 4+ 492.78027
764.49487
b6 2+-H2O 406.23062
b3+ 515.30493
y7 2
y4+-NH3 729.41187
y6
b16 2+ 1084.63660
b18 2+ 1176.67590
b8+ 1055.63367
b18 2+-NH3 1168.17224
b15 2
b23 4
b4
b2
b6 2
Ty
b12 5
b4 3
b6 3
b1
b5 2
b19 4
y4
b13 2
y8
b10+ 1525.98743
Pre+H, Precursor, Precursor-H2O, Precursor-H2O-NH3, Precursor-NH3, Pre-H
Immonium
y, y-H2O, y-NH3
b, b-H2O, b-NH3

#1	Immonium	b^{+}	b^{2+}	b^{3+}	b^{4+}	Seq.	y^{+}	y^{2+}	y^{3+}	y^{4+}	#2
1	273.21241	301.20732	151.10730	101.07396	76.05729	A-TMT6plex					30
2	72.08078	400.27574	200.64151	134.09676	100.82439	V	3817.02463	1909.01595	1273.01306	955.01162	29
3	88.03930	515.30268	258.15498	172.43908	129.58113	D	3717.95622	1859.48175	1239.99026	930.24451	28
4	101.07094	643.36126	322.18427	215.12527	161.59577	Q	3602.92927	1801.96828	1201.64794	901.48778	27
5	60.04439	730.39328	365.70028	244.13595	183.35378	S	3474.87070	1737.93899	1158.96175	869.47313	26
6	72.08078	829.46170	415.23449	277.15875	208.12088	V	3387.83867	1694.42297	1129.95107	847.71512	25
7	86.09643	942.54576	471.77652	314.85344	236.39190	L	3288.77025	1644.88877	1096.92827	822.94802	24
8	86.09643	1055.62983	528.31855	352.54813	264.66291	L	3175.68619	1588.34673	1059.23358	794.67701	23
9	86.09643	1168.71389	584.86058	390.24281	292.93393	L	3062.60213	1531.80470	1021.53889	766.40599	22
10	330.27026	1525.97179	763.48953	509.32878	382.24840	K-TMT6plex	2949.51806	1475.26267	983.84421	738.13497	21
11	70.06513	1623.02455	812.01591	541.67970	406.51159	P	2592.26017	1296.63372	864.75824	648.82050	20
12	102.05496	1752.06714	876.53721	584.69390	438.77224	E	2495.20740	1248.10734	832.40732	624.55731	19
13	60.04439	1839.09917	920.05322	613.70457	460.53025	S	2366.16481	1183.58604	789.39312	592.29666	18
14	102.05496	1968.14176	984.57452	656.71877	492.79090	E	2279.13278	1140.07003	760.38245	570.53865	17
15	86.09643	2081.22583	1041.11655	694.41346	521.06191	L	2150.09019	1075.54873	717.36825	538.27800	16
16	60.04439	2168.25786	1084.63257	723.42414	542.81992	S	2037.00613	1019.00670	679.67356	510.00699	15
17	70.06513	2265.31062	1133.15895	755.77506	567.08311	P	1949.97410	975.49069	650.66288	488.24898	14
18	60.04439	2352.34265	1176.67496	784.78573	588.84112	S	1852.92133	926.96431	618.31196	463.98579	13
19	204.07675	2583.40704	1292.20716	861.80720	646.60722	W-Nitro	1765.88931	883.44829	589.30129	442.22778	12
20	86.09643	2696.49110	1348.74919	899.50189	674.87823	I	1534.82491	767.91610	512.27982	384.46169	11
21	136.07569	2859.55443	1430.28085	953.85633	715.64407	Y	1421.74085	711.37406	474.58513	356.19067	10
22	87.05529	2973.59736	1487.30232	991.87064	744.15480	N	1258.67752	629.84240	420.23069	315.42484	9
23	86.09643	3086.68142	1543.84435	1029.56533	772.42581	L	1144.63459	572.82094	382.21638	286.91411	8
24	70.06513	3183.73419	1592.37073	1061.91625	796.68900	P	1031.55053	516.27890	344.52169	258.64309	7
25	30.03383	3240.75565	1620.88146	1080.92340	810.94437	G	934.49777	467.75252	312.17077	234.37990	6
26	104.05285	3371.79613	1686.40171	1124.60356	843.70449	M	877.47630	439.24179	293.16362	220.12453	5
27	101.07094	3499.85471	1750.43099	1167.28976	875.71914	Q	746.43582	373.72155	249.48346	187.36441	4
28	101.07094	3627.91329	1814.46028	1209.97595	907.73378	Q	618.37724	309.69226	206.79726	155.34977	3
29	87.05529	3741.95622	1871.48175	1247.99026	936.24451	N	490.31866	245.66297	164.11107	123.33512	2
30	330.27026					K-TMT6plex	376.27574	188.64151	126.09676	94.82439	1

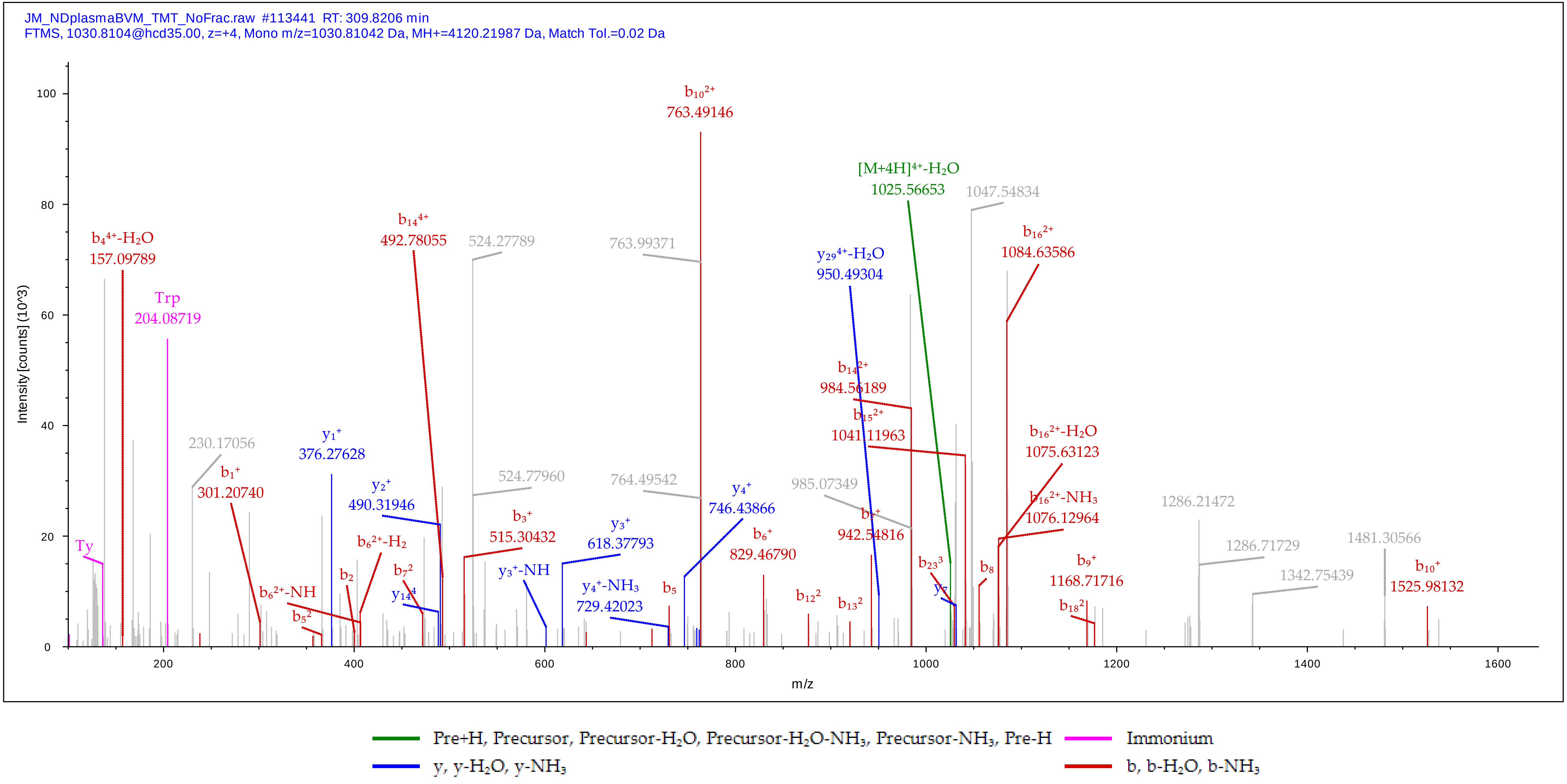
JM_NDplasmaBVM_TMT_NoFrac.raw #113441 RT: 309.8206 min
FTMS, 1030.8104@hcd35.00, z=+4, Mono m/z=1030.81042 Da, MH+=4120.21987 Da, Match Tol.=0.02 Da
Intensity [counts] (10^3)
m/z
b10 2+ 763.49146
[M+4H]4+-H2O 1025.56653
1047.54834
b4 4+-H2O 157.09789
b14 4+ 492.78055
524.27789
763.99371
y29 4+-H2O 950.49304
b16 2+ 1084.63586
Trp 204.08719
b14 2+ 984.56189
b15 2+ 1041.11963
b16 2+-H2O 1075.63123
230.17056
y1+ 376.27628
b1+ 301.20740
y2+ 490.31946
524.77960
764.49542
985.07349
y4+ 746.43866
b16 2+-NH3 1076.12964
1286.21472
b3+ 515.30432
b7+ 942.54816
b6+ 829.46790
Ty
b6 2+-H2
y3+ 618.37793
1286.71729
1481.30566
b2
b7 2
y3+-NH
b23 3
b8
b9+ 1168.71716
b10+ 1525.98132
b6 2+-NH
y4+-NH3 729.42023
b5
1342.75439
y14 4
b12 2
b13 2
y7
b5 2
b18 2
Pre+H, Precursor, Precursor-H2O, Precursor-H2O-NH3, Precursor-NH3, Pre-H
Immonium
y, y-H2O, y-NH3
b, b-H2O, b-NH3

#1	Immonium	b+	b2+	b3+	b4+	Seq.	y+	y2+	y3+	y4+	#2
1	259.19676	287.19167	144.09947	96.40208	72.55338	G-TMT6plex					31
2	30.03383	344.21314	172.61021	115.40923	86.80874	G	3465.73305	1733.37016	1155.91587	867.18872	30
3	72.08078	443.28155	222.14441	148.43203	111.57584	V	3408.71159	1704.85943	1136.90871	852.93335	29
4	88.03930	558.30849	279.65788	186.77435	140.33258	D	3309.64318	1655.32523	1103.88591	828.16625	28
5	88.03930	673.33544	337.17136	225.11666	169.08932	D	3194.61623	1597.81175	1065.54360	799.40952	27
6	102.05496	802.37803	401.69265	268.13086	201.34996	E	3079.58929	1540.29828	1027.20128	770.65278	26
7	104.05285	933.41851	467.21289	311.81102	234.11009	M	2950.54670	1475.77699	984.18708	738.39213	25
8	74.06004	1034.46619	517.73673	345.49358	259.37201	T	2819.50621	1410.25674	940.50692	705.63201	24
9	86.09643	1147.55026	574.27877	383.18827	287.64302	L	2718.45853	1359.73291	906.82436	680.37009	23
10	60.04439	1234.58228	617.79478	412.19895	309.40103	S	2605.37447	1303.19087	869.12967	652.09907	22
11	44.04948	1305.61940	653.31334	435.87798	327.16031	A	2518.34244	1259.67486	840.11900	630.34107	21
12	181.06077	1513.66780	757.33754	505.22745	379.17241	Y-Nitro	2447.30533	1224.15630	816.43996	612.58179	20
13	86.09643	1626.75187	813.87957	542.92214	407.44342	I	2239.25692	1120.13210	747.09049	560.56969	19
14	74.06004	1727.79955	864.40341	576.60470	432.70534	T	2126.17286	1063.59007	709.39580	532.29867	18
15	120.04776	1874.83495	937.92111	625.61650	469.46419	M-Oxidation	2025.12518	1013.06623	675.71324	507.03675	17
16	44.04948	1945.87206	973.43967	649.29554	487.22347	A	1878.08978	939.54853	626.70144	470.27790	16
17	86.09643	2058.95612	1029.98170	686.99023	515.49449	L	1807.05267	904.02997	603.02241	452.51862	15
18	86.09643	2172.04019	1086.52373	724.68491	543.76550	L	1693.96860	847.48794	565.32772	424.24761	14
19	102.05496	2301.08278	1151.04503	767.69911	576.02615	E	1580.88454	790.94591	527.63303	395.97659	13
20	60.04439	2388.11481	1194.56104	796.70979	597.78416	S	1451.84194	726.42461	484.61883	363.71594	12
21	60.04439	2475.14684	1238.07706	825.72046	619.54217	S	1364.80992	682.90860	455.60816	341.95794	11
22	86.09643	2588.23090	1294.61909	863.41515	647.81318	L	1277.77789	639.39258	426.59748	320.19993	10
23	70.06513	2685.28367	1343.14547	895.76607	672.07637	P	1164.69382	582.85055	388.90279	291.92891	9
24	44.04948	2756.32078	1378.66403	919.44511	689.83565	A	1067.64106	534.32417	356.55187	267.66572	8
25	74.06004	2857.36846	1429.18787	953.12767	715.09757	T	996.60395	498.80561	332.87283	249.90644	7
26	110.07127	2994.42737	1497.71732	998.81397	749.36230	H	895.55627	448.28177	299.19027	224.64452	6
27	70.06513	3091.48013	1546.24370	1031.16490	773.62549	P	758.49736	379.75232	253.50397	190.37980	5
28	72.08078	3190.54855	1595.77791	1064.18770	798.39259	V	661.44459	331.22593	221.15305	166.11661	4
29	72.08078	3289.61696	1645.31212	1097.21050	823.15970	V	562.37618	281.69173	188.13024	141.34950	3
30	60.04439	3376.64899	1688.82813	1126.22118	844.91770	S	463.30776	232.15752	155.10744	116.58240	2
31	330.27026					K-TMT6plex	376.27574	188.64151	126.09676	94.82439	1

Nitro-Tyr immonium ion is detected in MS/MS spectra and added brown colored in the following spectrum

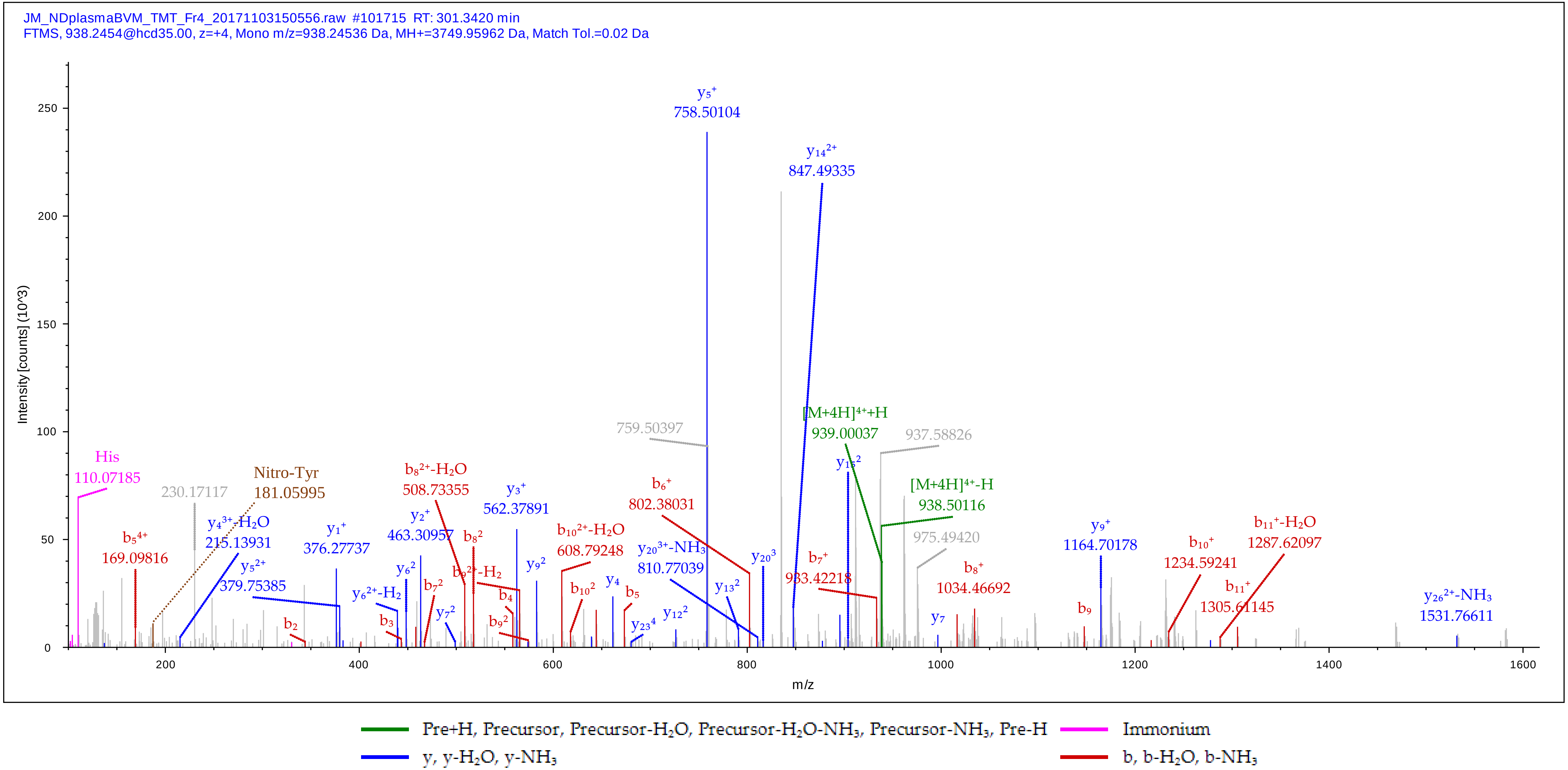

JM_NDplasmaBVM_TMT_Fr4_20171103150556.raw #101715 RT: 301.3420 min
FTMS, 938.2454@hcd35.00, z=+4, Mono m/z=938.24536 Da, MH+=3749.95962 Da, Match Tol.=0.02 Da
Intensity [counts] (10^3)
m/z
y_5^+ 758.50104
y_{14}^{2+} 847.49335
759.50397
$[M+4H]^{4+}$+H 939.00037
937.58826
His 110.07185
230.17117
Nitro-Tyr 181.05995
b_8^{2+}-H_2O 508.73355
y_3^+ 562.37891
b_6^+ 802.38031
y_{15}^2
$[M+4H]^{4+}$-H 938.50116
y_4^{3+}-H_2O 215.13931
b_5^{4+} 169.09816
y_1^+ 376.27737
y_2^+ 463.30957
b_8^2
b_{10}^{2+}-H_2O 608.79248
y_{20}^{3+}-NH_3 810.77039
y_{20}^3
975.49420
y_9^+ 1164.70178
b_{10}^+ 1234.59241
b_{11}^+-H_2O 1287.62097
y_5^{2+} 379.75385
y_6^2
b_8^{2+}-H_2
y_9^2
b_7^+ 933.42218
b_8^+ 1034.46692
y_6^{2+}-H_2
b_7^2
b_4
b_{10}^2
y_4
b_5
y_{13}^2
b_{11}^+ 1305.61145
y_{26}^{2+}-NH_3 1531.76611
b_2
b_3
y_7^2
b_9^2
y_{23}^4
y_{12}^2
b_9
y_7
Pre+H, Precursor, Precursor-H_2O, Precursor-H_2O-NH_3, Precursor-NH_3, Pre-H
Immonium
y, y-H_2O, y-NH_3
b, b-H_2O, b-NH_3

#1	Immonium	b⁺	b²⁺	b³⁺	Seq.	y⁺	y²⁺	y³⁺	#2
1	315.25936	343.25427	172.13077	115.08961	L-TMT6plex				24
2	74.06004	444.30195	222.65461	148.77217	T	2808.39970	1404.70349	936.80475	23
3	44.04948	515.33906	258.17317	172.45121	A	2707.35202	1354.17965	903.12219	22
4	101.07094	643.39764	322.20246	215.13740	Q	2636.31491	1318.66109	879.44315	21
5	70.06513	740.45041	370.72884	247.48832	P	2508.25633	1254.63180	836.75696	20
6	44.04948	811.48752	406.24740	271.16736	A	2411.20356	1206.10542	804.40604	19
7	70.06513	908.54028	454.77378	303.51828	P	2340.16645	1170.58686	780.72700	18
8	60.04439	995.57231	498.28979	332.52896	S	2243.11369	1122.06048	748.37608	17
9	70.06513	1092.62508	546.81618	364.87988	P	2156.08166	1078.54447	719.36540	16
10	102.05496	1221.66767	611.33747	407.89407	E	2059.02889	1030.01809	687.01448	15
11	88.03930	1336.69461	668.85094	446.23639	D	1929.98630	965.49679	644.00029	14
12	86.09643	1449.77868	725.39298	483.93108	L	1814.95936	907.98332	605.65797	13
13	74.06004	1550.82635	775.91682	517.61364	T	1701.87530	851.44129	567.96328	12
14	86.09643	1663.91042	832.45885	555.30832	L	1600.82762	800.91745	534.28072	11
15	60.04439	1750.94245	875.97486	584.31900	S	1487.74355	744.37541	496.58604	10
16	104.05285	1881.98293	941.49510	627.99916	M	1400.71152	700.85940	467.57536	9
17	60.04439	1969.01496	985.01112	657.00984	S	1269.67104	635.33916	423.89520	8
18	74.06004	2070.06264	1035.53496	690.69240	T	1182.63901	591.82314	394.88452	7
19	86.09643	2183.14670	1092.07699	728.38708	I	1081.59133	541.29930	361.20196	6
20	120.04776	2330.18210	1165.59469	777.39888	M-Oxidation	968.50727	484.75727	323.50727	5
21	204.07675	2561.24649	1281.12688	854.42035	W-Nitro	821.47187	411.23957	274.49547	4
22	86.09643	2674.33056	1337.66892	892.11504	L-TMT6plex	590.40748	295.70738	197.47401	3
23	74.06004	2775.37823	1388.19276	925.79760	T	477.32341	239.16535	159.77932	2
24	330.27026				K-TMT6plex	376.27574	188.64151	126.09676	1

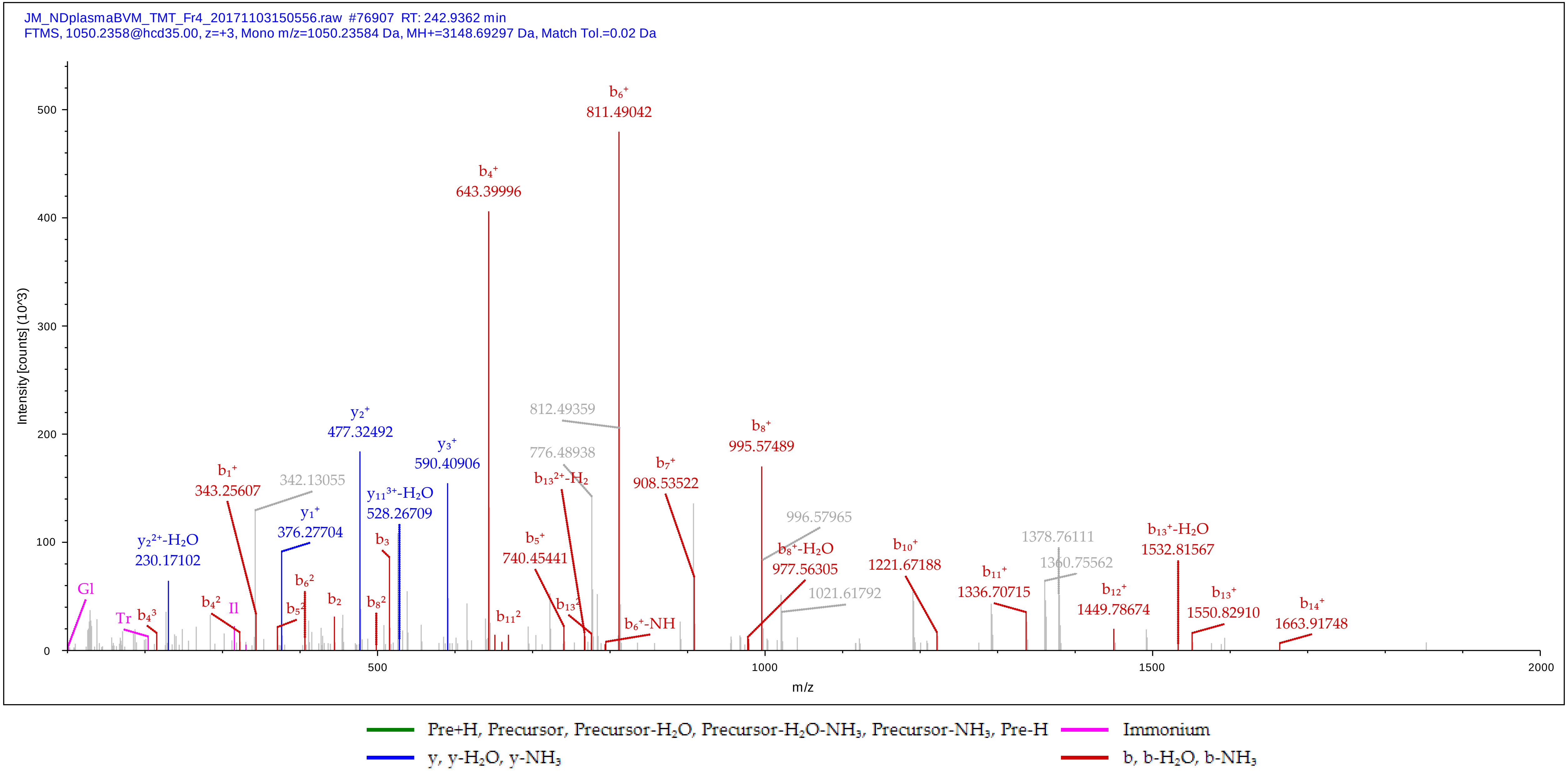

JM_NDplasmaBVM_TMT_Fr4_20171103150556.raw #76907 RT: 242.9362 min
FTMS, 1050.2358@hcd35.00, z=+3, Mono m/z=1050.23584 Da, MH+=3148.69297 Da, Match Tol.=0.02 Da
Intensity [counts] (10^3)
m/z
b6+ 811.49042
b4+ 643.39996
812.49359
y2+ 477.32492
y3+ 590.40906
776.48938
b8+ 995.57489
b1+ 343.25607
342.13055
b13 2+-H2
b7+ 908.53522
y11 3+-H2O 528.26709
y1+ 376.27704
996.57965
y2 2+-H2O 230.17102
b3
b5+ 740.45441
b8+-H2O 977.56305
b10+ 1221.67188
1378.76111
b13+-H2O 1532.81567
1360.75562
b11+ 1336.70715
1021.61792
b12+ 1449.78674
b13+ 1550.82910
b14+ 1663.91748
Gl
Tr
b4 3
b4 2
Il
b6 2
b5 2
b2
b8 2
b11 2
b13 2
b6+-NH
Pre+H, Precursor, Precursor-H2O, Precursor-H2O-NH3, Precursor-NH3, Pre-H
Immonium
y, y-H2O, y-NH3
b, b-H2O, b-NH3

#1	Immonium	b⁺	b²⁺	b³⁺	b⁴⁺	b⁵⁺	Seq.	y⁺	y²⁺	y³⁺	y⁴⁺	y⁵⁺	#2
1	301.24371	329.23862	165.12295	110.41773	83.06511	66.65355	V-TMT6plex						18
2	87.05529	443.28155	222.14441	148.43203	111.57584	89.46213	N	2574.01690	1287.51209	858.67715	644.25968	515.60920	17
3	110.07127	580.34046	290.67387	194.11834	145.84057	116.87391	H	2459.97398	1230.49063	820.66284	615.74895	492.80062	16
4	330.27026	937.59836	469.30282	313.20430	235.15505	188.32549	K-TMT6plex	2322.91506	1161.96117	774.97654	581.48422	465.38883	15
5	102.05496	1066.64095	533.82411	356.21850	267.41569	214.13401	E	1965.65717	983.33222	655.89057	492.16975	393.93726	14
6	102.05496	1195.68354	598.34541	399.23270	299.67634	239.94253	E	1836.61458	918.81093	612.87638	459.90910	368.12874	13
7	88.03930	1310.71049	655.85888	437.57501	328.43308	262.94792	D	1707.57198	854.28963	569.86218	427.64845	342.32022	12
8	60.04439	1397.74251	699.37490	466.58569	350.19109	280.35432	S	1592.54504	796.77616	531.51986	398.89172	319.31483	11
9	133.04301	1557.77316	779.39022	519.92924	390.19875	312.36045	C-Carbamidomethyl	1505.51301	753.26014	502.50919	377.13371	301.90842	10
10	60.04439	1644.80519	822.90623	548.93991	411.95676	329.76686	S	1345.48236	673.24482	449.16564	337.12605	269.90229	9
11	136.07569	1807.86852	904.43790	603.29436	452.72259	362.37953	Y	1258.45034	629.72881	420.15496	315.36804	252.49589	8
12	120.08078	1954.93693	977.97210	652.31716	489.48969	391.79321	F	1095.38701	548.19714	365.80052	274.60221	219.88322	7
13	181.06077	2162.98534	1081.99631	721.66663	541.50179	433.40289	Y-Nitro	948.31859	474.66293	316.77772	237.83511	190.46954	6
14	133.04301	2323.01599	1162.01163	775.01018	581.50945	465.40902	C-Carbamidomethyl	740.27019	370.63873	247.42825	185.82300	148.85986	5
15	120.04776	2470.05139	1235.52933	824.02198	618.26830	494.81610	M-Oxidation	580.23954	290.62341	194.08470	145.81534	116.85373	4
16	102.05496	2599.09398	1300.05063	867.03618	650.52895	520.62462	E	433.20414	217.10571	145.07290	109.05649	87.44665	3
17	102.05496	2728.13657	1364.57193	910.05038	682.78960	546.43314	E	304.16155	152.58441	102.05870	76.79584	61.63813	2
18	129.11347						R	175.11895	88.06311	59.04450	44.53520	35.82961	1

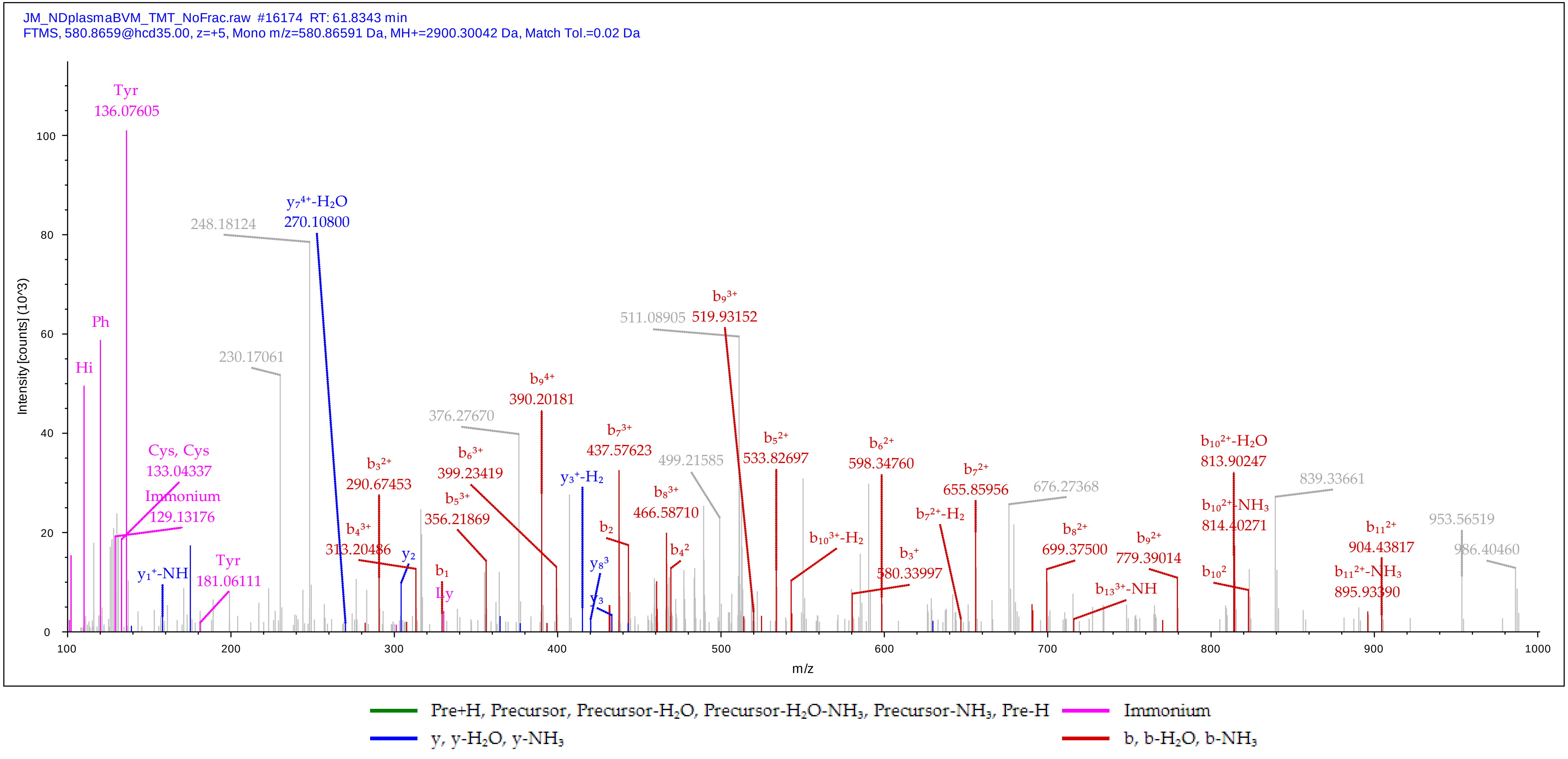
JM_NDplasmaBVM_TMT_NoFrac.raw #16174 RT: 61.8343 min
FTMS, 580.8659@hcd35.00, z=+5, Mono m/z=580.86591 Da, MH+=2900.30042 Da, Match Tol.=0.02 Da
Intensity [counts] (10^3)
m/z
Tyr 136.07605
y7 4+-H2O 270.10800
248.18124
b9 3+ 519.93152
511.08905
b9 4+ 390.20181
Pre+H, Precursor, Precursor-H2O, Precursor-H2O-NH3, Precursor-NH3, Pre-H
Immonium
y, y-H2O, y-NH3
b, b-H2O, b-NH3

#1	Immonium	b^{+}	b^{2+}	b^{3+}	Seq.	y^{+}	y^{2+}	y^{3+}	#2
1	301.24371	329.23862	165.12295	110.41773	V-TMT6plex				23
2	136.07569	492.30195	246.65461	164.77217	Y	2946.18072	1473.59400	982.73176	22
3	88.03930	607.32889	304.16809	203.11448	D	2783.11739	1392.06233	928.37731	21
4	136.07569	770.39222	385.69975	257.46893	Y	2668.09044	1334.54886	890.03500	20
5	136.07569	933.45555	467.23141	311.82337	Y	2505.02712	1253.01720	835.68056	19
6	102.05496	1062.49814	531.75271	354.83757	E	2341.96379	1171.48553	781.32611	18
7	74.06004	1163.54582	582.27655	388.52013	T	2212.92119	1106.96424	738.31192	17
8	88.03930	1278.57277	639.79002	426.86244	D	2111.87352	1056.44040	704.62936	16
9	102.05496	1407.61536	704.31132	469.87664	E	1996.84657	998.92692	666.28704	15
10	104.05285	1538.65584	769.83156	513.55680	M	1867.80398	934.40563	623.27284	14
11	44.04948	1609.69296	805.35012	537.23584	A	1736.76350	868.88539	579.59268	13
12	120.08078	1756.76137	878.88432	586.25864	F	1665.72638	833.36683	555.91365	12
13	44.04948	1827.79848	914.40288	609.93768	A	1518.65797	759.83262	506.89084	11
14	102.05496	1956.84108	978.92418	652.95188	E	1447.62085	724.31407	483.21180	10
15	181.06077	2164.88948	1082.94838	722.30135	Y-Nitro	1318.57826	659.79277	440.19760	9
16	60.04439	2251.92151	1126.46439	751.31202	S	1110.52985	555.76857	370.84814	8
17	60.04439	2338.95354	1169.98041	780.32270	S	1023.49783	512.25255	341.83746	7
18	70.06513	2436.00630	1218.50679	812.67362	P	936.46580	468.73654	312.82678	6
19	133.04301	2596.03695	1298.52211	866.01717	C-Carbamidomethyl	839.41303	420.21016	280.47586	5
20	60.04439	2683.06898	1342.03813	895.02784	S	679.38239	340.19483	227.13231	4
21	74.06004	2784.11666	1392.56197	928.71040	T	592.35036	296.67882	198.12164	3
22	88.03930	2899.14360	1450.07544	967.05272	D	491.30268	246.15498	164.43908	2
23	330.27026				K-TMT6plex	376.27574	188.64151	126.09676	1

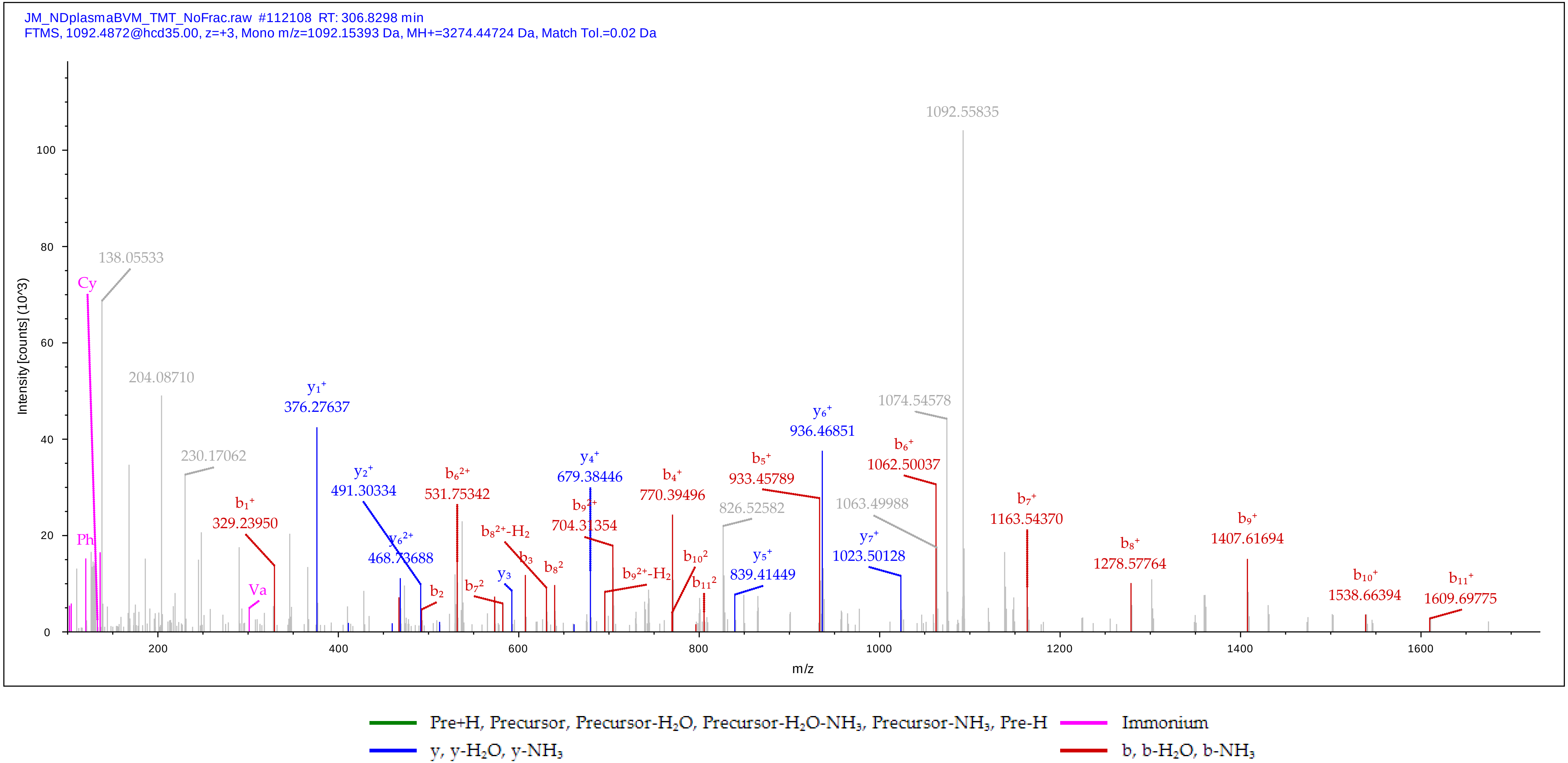
JM_NDplasmaBVM_TMT_NoFrac.raw #112108 RT: 306.8298 min
FTMS, 1092.4872@hcd35.00, z=+3, Mono m/z=1092.15393 Da, MH+=3274.44724 Da, Match Tol.=0.02 Da
Intensity [counts] (10^3)
m/z
1092.55835
138.05533
204.08710
230.17062
y1+ 376.27637
y2+ 491.30334
y6 2+ 468.73688
b1+ 329.23950
b6 2+ 531.75342
y4+ 679.38446
b9 2+ 704.31354
b4+ 770.39496
b5+ 933.45789
y6+ 936.46851
b6+ 1062.50037
1074.54578
1063.49988
826.52582
y7+ 1023.50128
y5+ 839.41449
b7+ 1163.54370
b8+ 1278.57764
b9+ 1407.61694
b10+ 1538.66394
b11+ 1609.69775
Cy
Ph
Va
b2
b3
y3
b7 2
b8 2
b8 2+-H2
b9 2+-H2
b10 2
b11 2
Pre+H, Precursor, Precursor-H2O, Precursor-H2O-NH3, Precursor-NH3, Pre-H
Immonium
y, y-H2O, y-NH3
b, b-H2O, b-NH3

#1	Immonium	b⁺	b²⁺	b³⁺	Seq.	y⁺	y²⁺	y³⁺	#2
1	365.23862	393.23354	197.12041	131.74936	Y-TMT6plex				16
2	104.05285	524.27402	262.64065	175.42952	M	2009.04626	1005.02677	670.35360	15
3	72.08078	623.34244	312.17486	208.45233	V	1878.00577	939.50653	626.67344	14
4	86.09643	736.42650	368.71689	246.14702	L	1778.93736	889.97232	593.65064	13
5	72.08078	835.49491	418.25109	279.16982	V	1665.85330	833.43029	555.95595	12
6	70.06513	932.54768	466.77748	311.52074	P	1566.78488	783.89608	522.93315	11
7	60.04439	1019.57971	510.29349	340.53142	S	1469.73212	735.36970	490.58222	10
8	101.07094	1147.63828	574.32278	383.21761	Q	1382.70009	691.85368	461.57155	9
9	86.09643	1260.72235	630.86481	420.91230	L	1254.64151	627.82439	418.88536	8
10	181.06077	1468.77075	734.88901	490.26177	Y-Nitro	1141.55745	571.28236	381.19067	7
11	74.06004	1569.81843	785.41285	523.94433	T	933.50904	467.25816	311.84120	6
12	102.05496	1698.86102	849.93415	566.95853	E	832.46136	416.73432	278.15864	5
13	74.06004	1799.90870	900.45799	600.64109	T	703.41877	352.21302	235.14444	4
14	70.06513	1896.96147	948.98437	632.99201	P	602.37109	301.68918	201.46188	3
15	102.05496	2026.00406	1013.50567	676.00620	E	505.31833	253.16280	169.11096	2
16	330.27026				K-TMT6plex	376.27574	188.64151	126.09676	1

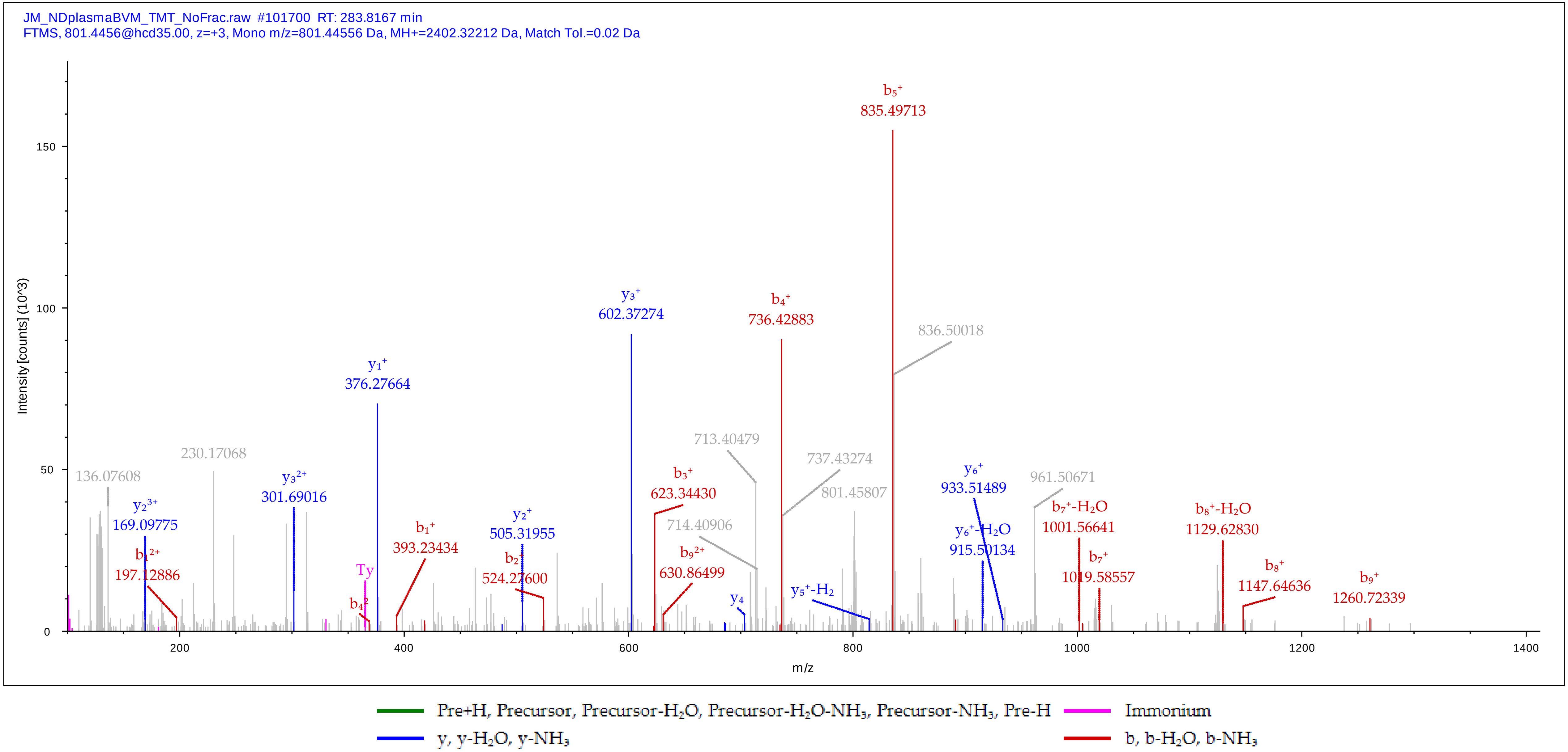
JM_NDplasmaBVM_TMT_NoFrac.raw #101700 RT: 283.8167 min
FTMS, 801.4456@hcd35.00, z=+3, Mono m/z=801.44556 Da, MH+=2402.32212 Da, Match Tol.=0.02 Da
Intensity [counts] (10^3)
m/z
b5+ 835.49713
b4+ 736.42883
y3+ 602.37274
y1+ 376.27664
836.50018
713.40479
737.43274
801.45807
961.50671
230.17068
136.07608
y3 2+ 301.69016
y2 3+ 169.09775
b1 2+ 197.12886
b1+ 393.23434
y2+ 505.31955
b2+ 524.27600
b3+ 623.34430
714.40906
b9 2+ 630.86499
y6+ 933.51489
y6+-H2O 915.50134
b7+-H2O 1001.56641
b7+ 1019.58557
b8+-H2O 1129.62830
b8+ 1147.64636
b9+ 1260.72339
y5+-H2
y4
Ty
b4 2+
Pre+H, Precursor, Precursor-H2O, Precursor-H2O-NH3, Precursor-NH3, Pre-H
Immonium
y, y-H2O, y-NH3
b, b-H2O, b-NH3

#1	Immonium	b+	b2+	b3+	b4+	Seq.	y+	y2+	y3+	y4+	#2
1	362.20594	390.20086	195.60407	130.73847	98.30567	C-TMT6plex-Carbamidomethyl					34
2	60.04439	477.23289	239.12008	159.74915	120.06368	S	3691.60607	1846.30668	1231.20688	923.65698	33
3	102.05496	606.27548	303.64138	202.76334	152.32433	E	3604.57405	1802.79066	1202.19620	901.89897	32
4	74.06004	707.32316	354.16522	236.44590	177.58625	T	3475.53145	1738.26936	1159.18200	869.63832	31
5	30.03383	764.34462	382.67595	255.45306	191.84161	G	3374.48377	1687.74553	1125.49944	844.37640	30
6	30.03383	821.36608	411.18668	274.46021	206.09698	G	3317.46231	1659.23479	1106.49229	830.12104	29
7	60.04439	908.39811	454.70269	303.47089	227.85499	S	3260.44085	1630.72406	1087.48513	815.86567	28
8	72.08078	1007.46653	504.23690	336.49369	252.62209	V	3173.40882	1587.20805	1058.47446	794.10766	27
9	72.08078	1106.53494	553.77111	369.51650	277.38919	V	3074.34040	1537.67384	1025.45165	769.34056	26
10	102.05496	1235.57753	618.29240	412.53070	309.64984	E	2975.27199	1488.13963	992.42885	744.57346	25
11	86.09643	1348.66160	674.83444	450.22538	337.92086	L	2846.22940	1423.61834	949.41465	712.31281	24
12	70.06513	1445.71436	723.36082	482.57630	362.18405	P	2733.14533	1367.07631	911.71996	684.04179	23
13	74.06004	1546.76204	773.88466	516.25886	387.44597	T	2636.09257	1318.54992	879.36904	659.77860	22
14	72.08078	1645.83045	823.41886	549.28167	412.21307	V	2535.04489	1268.02608	845.68648	634.51668	21
15	60.04439	1732.86248	866.93488	578.29234	433.97108	S	2435.97648	1218.49188	812.66368	609.74958	20
16	101.07094	1860.92106	930.96417	620.97854	465.98572	Q	2348.94445	1174.97586	783.65300	587.99157	19
17	102.05496	1989.96365	995.48546	663.99274	498.24637	E	2220.88587	1110.94657	740.96681	555.97693	18
18	70.06513	2087.01642	1044.01185	696.34366	522.50956	P	2091.84328	1046.42528	697.95261	523.71628	17
19	60.04439	2174.04844	1087.52786	725.35433	544.26757	S	1994.79052	997.89890	665.60169	499.45309	16
20	30.03383	2231.06991	1116.03859	744.36149	558.52293	G	1907.75849	954.38288	636.59101	477.69508	15
21	70.06513	2328.12267	1164.56497	776.71241	582.78613	P	1850.73702	925.87215	617.58386	463.43971	14
22	60.04439	2415.15470	1208.08099	805.72308	604.54413	S	1753.68426	877.34577	585.23294	439.17652	13
23	88.03930	2530.18164	1265.59446	844.06540	633.30087	D	1666.65223	833.82975	556.22226	417.41852	12
24	60.04439	2617.21367	1309.11047	873.07607	655.05888	S	1551.62529	776.31628	517.87995	388.66178	11
25	102.05496	2746.25626	1373.63177	916.09027	687.31952	E	1464.59326	732.80027	488.86927	366.90377	10
26	74.06004	2847.30394	1424.15561	949.77283	712.58144	T	1335.55067	668.27897	445.85507	334.64312	9
27	88.03930	2962.33089	1481.66908	988.11515	741.33818	D	1234.50299	617.75513	412.17251	309.38120	8
28	133.04301	3122.36153	1561.68441	1041.45870	781.34584	C-Carbamidomethyl	1119.47605	560.24166	373.83020	280.62447	7
29	120.04776	3269.39693	1635.20211	1090.47050	818.10469	M-Oxidation	959.44540	480.22634	320.48665	240.61681	6
30	181.06077	3477.44534	1739.22631	1159.81996	870.11679	Y-Nitro	812.41000	406.70864	271.47485	203.85796	5
31	30.03383	3534.46680	1767.73704	1178.82712	884.37216	G	604.36159	302.68443	202.12538	151.84586	4
32	87.05529	3648.50973	1824.75850	1216.84143	912.88289	N	547.34013	274.17370	183.11823	137.59049	3
33	30.03383	3705.53120	1853.26924	1235.84858	927.13826	G	433.29720	217.15224	145.10392	109.07976	2
34	330.27026					K-TMT6plex	376.27574	188.64151	126.09676	94.82439	1

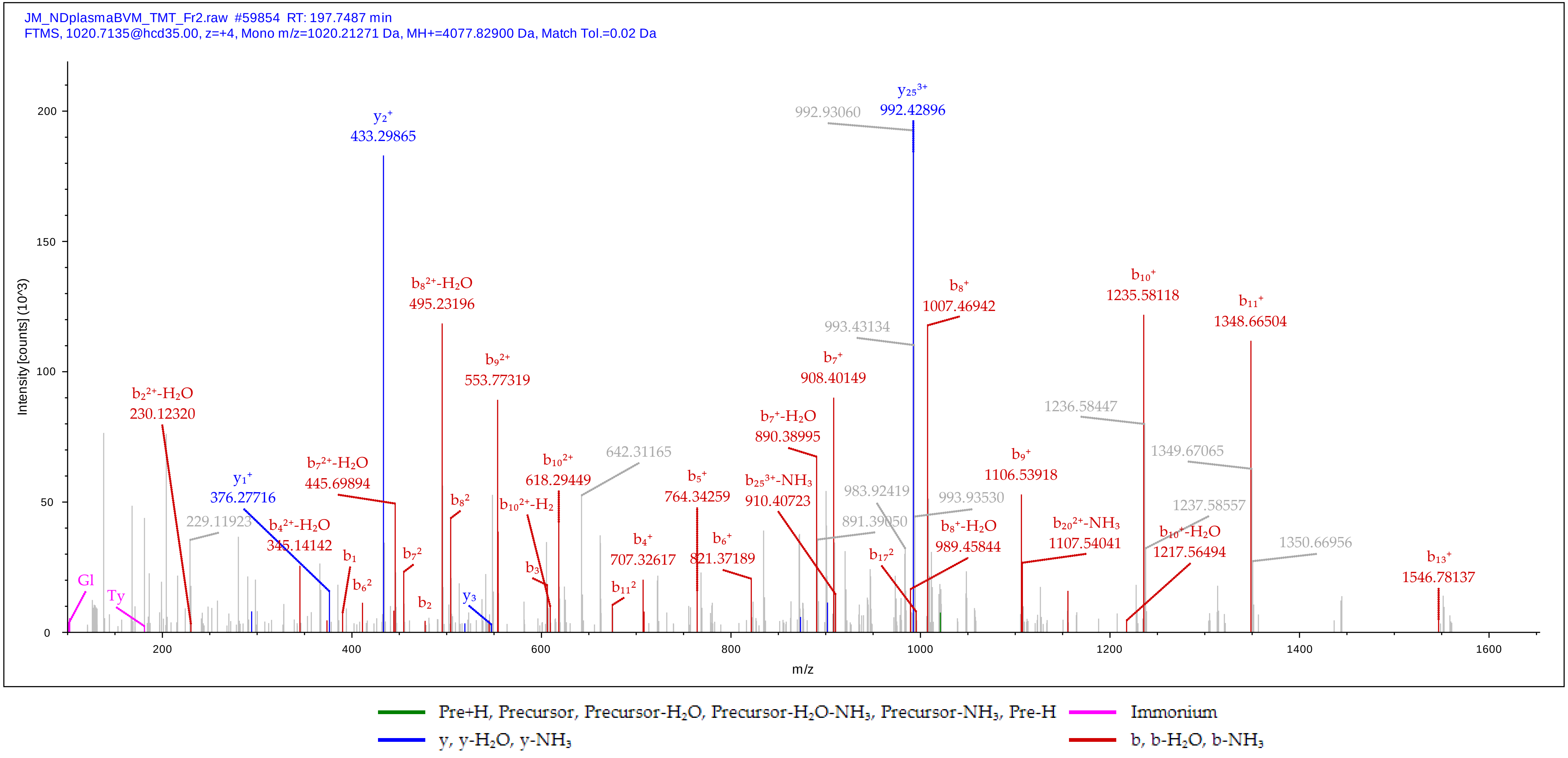

JM_NDplasmaBVM_TMT_Fr2.raw #59854 RT: 197.7487 min
FTMS, 1020.7135@hcd35.00, z=+4, Mono m/z=1020.21271 Da, MH+=4077.82900 Da, Match Tol.=0.02 Da
Intensity [counts] (10^3)
m/z
y2+ 433.29865
y25 3+ 992.42896
992.93060
993.43134
b8 2+-H2O 495.23196
b9 2+ 553.77319
b8+ 1007.46942
b10+ 1235.58118
b11+ 1348.66504
b7+ 908.40149
b2 2+-H2O 230.12320
b7+-H2O 890.38995
1236.58447
1349.67065
b9+ 1106.53918
b7 2+-H2O 445.69894
y1+ 376.27716
b10 2+ 618.29449
642.31165
b5+ 764.34259
b25 3+-NH3 910.40723
983.92419
993.93530
229.11923
b4 2+-H2O 345.14142
b8 2
b10 2+-H2
891.39050
1237.58557
b8+-H2O 989.45844
b20 2+-NH3 1107.54041
b10+-H2O 1217.56494
b4+ 707.32617
b6+ 821.37189
1350.66956
b1
b7 2
b17 2
b13+ 1546.78137
b3
b6 2
b11 2
Gl
Ty
y3
b2
Pre+H, Precursor, Precursor-H2O, Precursor-H2O-NH3, Precursor-NH3, Pre-H
Immonium
y, y-H2O, y-NH3
b, b-H2O, b-NH3

#1	Immonium	b+	b2+	b3+	Seq.	y+	y2+	y3+	#2
1	365.23862	393.23354	197.12041	131.74936	Y-TMT6plex				20
2	88.03930	508.26048	254.63388	170.09168	D	2763.01862	1382.01295	921.67772	19
3	136.07569	671.32381	336.16554	224.44612	Y	2647.99168	1324.49948	883.33541	18
4	133.04301	831.35446	416.18087	277.78967	C-Carbamidomethyl	2484.92835	1242.96781	828.98097	17
5	87.05529	945.39738	473.20233	315.80398	N	2324.89770	1162.95249	775.63742	16
6	86.09643	1058.48145	529.74436	353.49867	I	2210.85477	1105.93102	737.62311	15
7	70.06513	1155.53421	578.27074	385.84959	P	2097.77071	1049.38899	699.92842	14
8	102.05496	1284.57681	642.79204	428.86379	E	2000.71794	1000.86261	667.57750	13
9	133.04301	1444.60745	722.80737	482.20734	C-Carbamidomethyl	1871.67535	936.34131	624.56330	12
10	102.05496	1573.65005	787.32866	525.22153	E	1711.64470	856.32599	571.21975	11
11	102.05496	1702.69264	851.84996	568.23573	E	1582.60211	791.80469	528.20555	10
12	102.05496	1831.73523	916.37125	611.24993	E	1453.55952	727.28340	485.19136	9
13	133.04301	1991.76588	996.38658	664.59348	C-Carbamidomethyl	1324.51692	662.76210	442.17716	8
14	120.04776	2138.80128	1069.90428	713.60528	M-Oxidation	1164.48628	582.74678	388.83361	7
15	181.06077	2346.84969	1173.92848	782.95475	Y-Nitro	1017.45088	509.22908	339.82181	6
16	133.04301	2506.88034	1253.94381	836.29830	C-Carbamidomethyl	809.40247	405.20487	270.47234	5
17	60.04439	2593.91236	1297.45982	865.30897	S	649.37182	325.18955	217.12879	4
18	30.03383	2650.93383	1325.97055	884.31613	G	562.33979	281.67353	188.11812	3
19	102.05496	2779.97642	1390.49185	927.33032	E	505.31833	253.16280	169.11096	2
20	330.27026				K-TMT6plex	376.27574	188.64151	126.09676	1

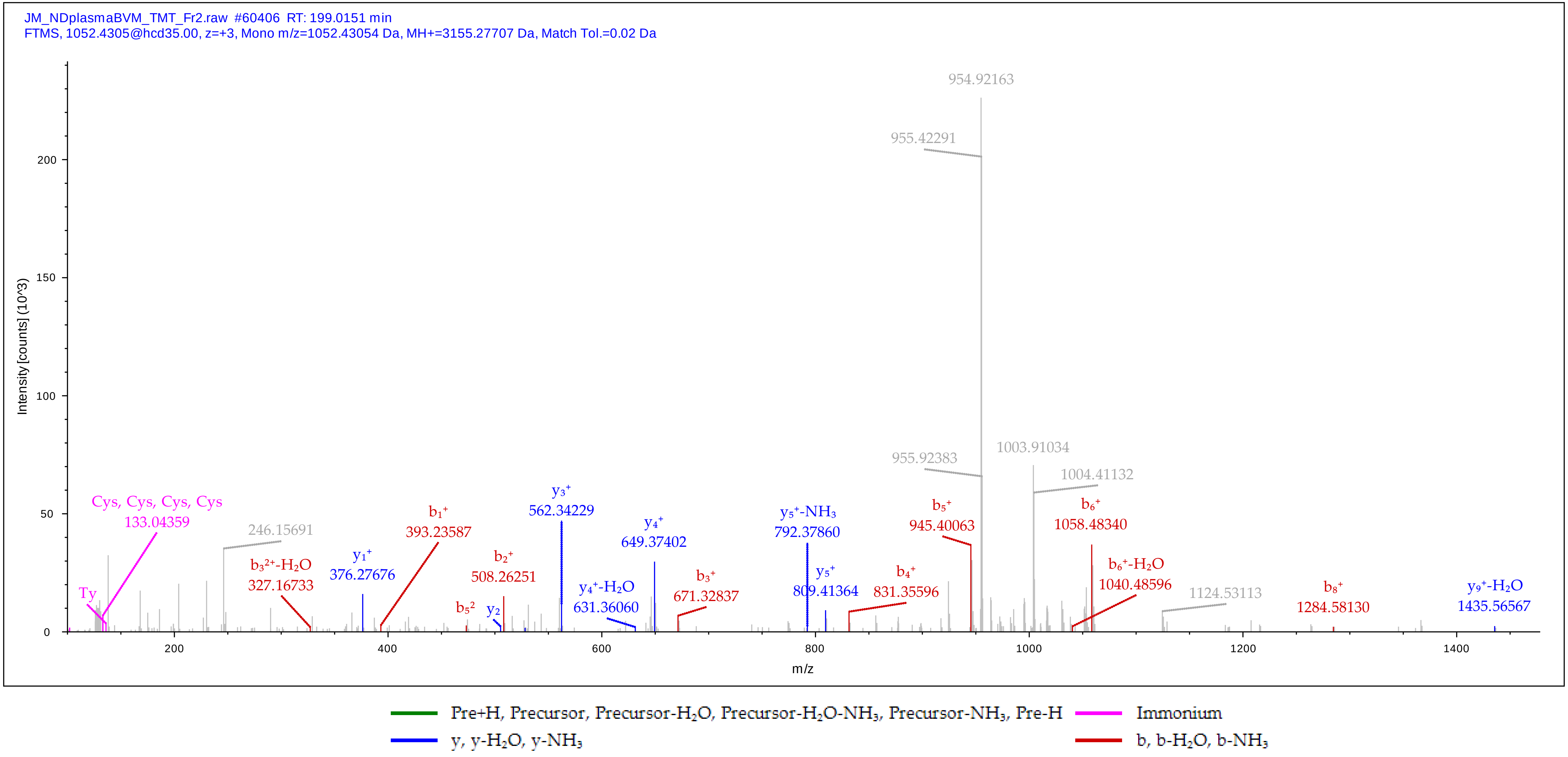
JM_NDplasmaBVM_TMT_Fr2.raw #60406 RT: 199.0151 min
FTMS, 1052.4305@hcd35.00, z=+3, Mono m/z=1052.43054 Da, MH+=3155.27707 Da, Match Tol.=0.02 Da
Intensity [counts] (10^3)
m/z
954.92163
955.42291
955.92383
1003.91034
1004.41132
246.15691
1124.53113
Cys, Cys, Cys, Cys
133.04359
Ty
b3²⁺-H2O
327.16733
y1⁺
376.27676
b1⁺
393.23587
b2⁺
508.26251
b5²
y2
y3⁺
562.34229
y4⁺-H2O
631.36060
y4⁺
649.37402
b3⁺
671.32837
y5⁺-NH3
792.37860
y5⁺
809.41364
b4⁺
831.35596
b5⁺
945.40063
b6⁺
1058.48340
b6⁺-H2O
1040.48596
b8⁺
1284.58130
y9⁺-H2O
1435.56567
Pre+H, Precursor, Precursor-H2O, Precursor-H2O-NH3, Precursor-NH3, Pre-H
Immonium
y, y-H2O, y-NH3
b, b-H2O, b-NH3

#1	Immonium	b+	b2+	b3+	Seq.	y+	y2+	y3+	#2
1	273.21241	301.20732	151.10730	101.07396	A-TMT6plex				22
2	104.05285	432.24781	216.62754	144.75412	M	2427.09889	1214.05308	809.70448	21
3	30.03383	489.26927	245.13827	163.76127	G	2296.05841	1148.53284	766.02432	20
4	72.08078	588.33768	294.67248	196.78408	V	2239.03694	1120.02211	747.01717	19
5	70.06513	685.39045	343.19886	229.13500	P	2139.96853	1070.48790	713.99436	18
6	104.05285	816.43093	408.71910	272.81516	M	2042.91577	1021.96152	681.64344	17
7	104.05285	947.47142	474.23935	316.49532	M	1911.87528	956.44128	637.96328	16
8	30.03383	1004.49288	502.75008	335.50248	G	1780.83480	890.92104	594.28312	15
9	86.09643	1117.57695	559.29211	373.19717	L	1723.81333	862.41030	575.27596	14
10	88.03930	1232.60389	616.80558	411.53948	D	1610.72927	805.86827	537.58127	13
11	181.06077	1440.65229	720.82979	480.88895	Y-Nitro	1495.70233	748.35480	499.23896	12
12	60.04439	1527.68432	764.34580	509.89963	S	1287.65392	644.33060	429.88949	11
13	88.03930	1642.71127	821.85927	548.24194	D	1200.62189	600.81458	400.87881	10
14	102.05496	1771.75386	886.38057	591.25614	E	1085.59495	543.30111	362.53650	9
15	86.09643	1884.83792	942.92260	628.95083	I	956.55236	478.77982	319.52230	8
16	87.05529	1998.88085	999.94406	666.96513	N	843.46829	422.23778	281.82761	7
17	101.07094	2126.93943	1063.97335	709.65133	Q	729.42536	365.21632	243.81331	6
18	72.08078	2226.00784	1113.50756	742.67413	V	601.36679	301.18703	201.12711	5
19	72.08078	2325.07626	1163.04177	775.69694	V	502.29837	251.65282	168.10431	4
20	102.05496	2454.11885	1227.56306	818.71113	E	403.22996	202.11862	135.08150	3
21	72.08078	2553.18726	1277.09727	851.73394	V	274.18737	137.59732	92.06731	2
22	129.11347				R	175.11895	88.06311	59.04450	1

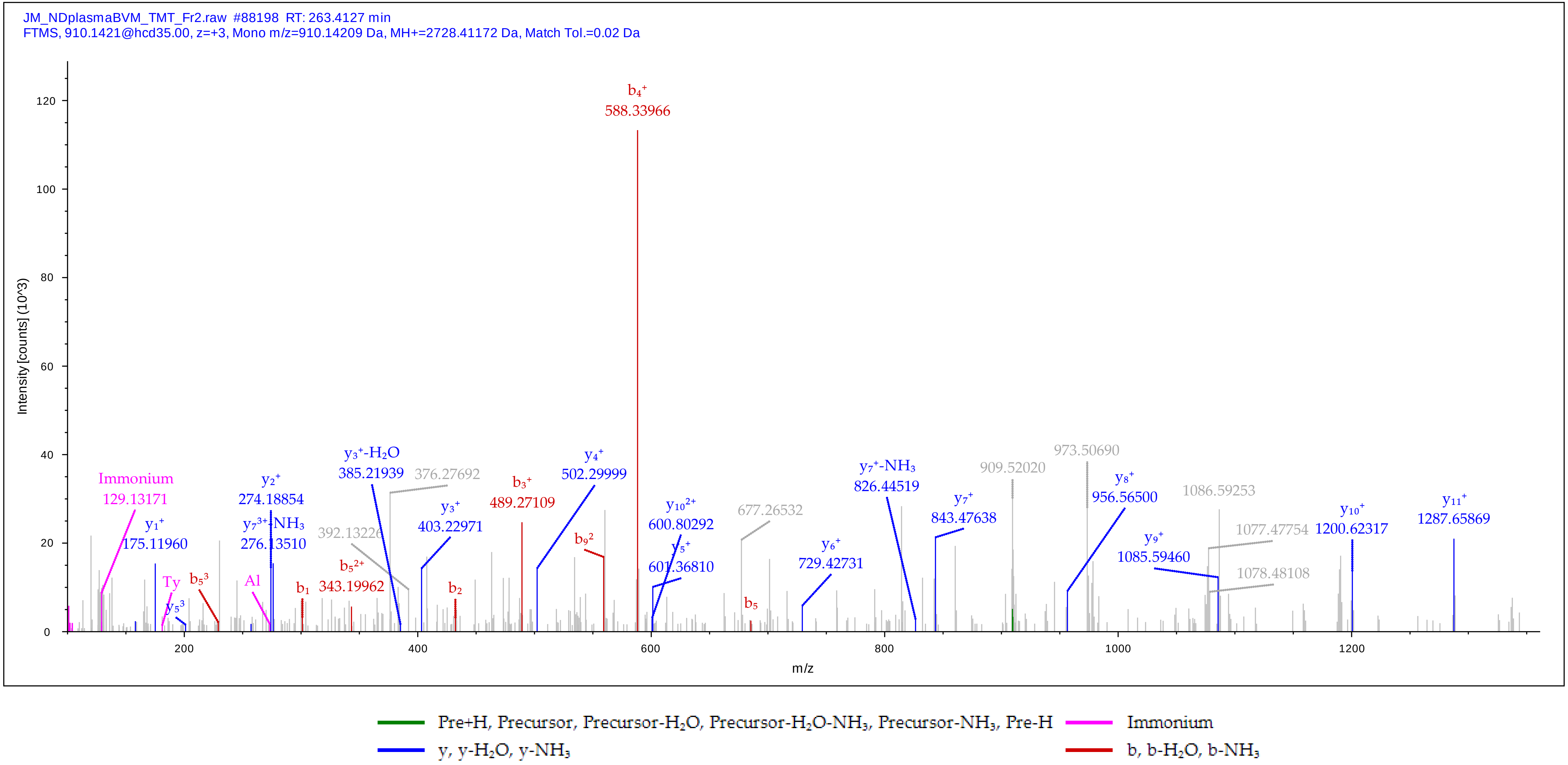

JM_NDplasmaBVM_TMT_Fr2.raw #88198 RT: 263.4127 min
FTMS, 910.1421@hcd35.00, z=+3, Mono m/z=910.14209 Da, MH+=2728.41172 Da, Match Tol.=0.02 Da
Intensity [counts] (10^3)
m/z
b4+ 588.33966
y3+-H2O 385.21939
376.27692
b3+ 489.27109
y4+ 502.29999
Immonium 129.13171
y1+ 175.11960
y2+ 274.18854
y7 3+-NH3 276.13510
y3+ 403.22971
392.13226
b5 2+ 343.19962
Ty
Al
b1
b2
b5
b9 2
y10 2+ 600.80292
y5+ 601.36810
677.26532
y6+ 729.42731
y7+-NH3 826.44519
y7+ 843.47638
909.52020
973.50690
y8+ 956.56500
1086.59253
y9+ 1085.59460
1077.47754
1078.48108
y10+ 1200.62317
y11+ 1287.65869
Pre+H, Precursor, Precursor-H2O, Precursor-H2O-NH3, Precursor-NH3, Pre-H
Immonium
y, y-H2O, y-NH3
b, b-H2O, b-NH3

#1	Immonium	b^{+}	b^{2+}	b^{3+}	Seq.	y^{+}	y^{2+}	y^{3+}	#2
1	331.21789	359.21280	180.11004	120.40912	E-TMT6plex				17
2	87.05529	473.25573	237.13150	158.42343	N	2134.98474	1067.99601	712.33310	16
3	30.03383	530.27719	265.64223	177.43058	G	2020.94181	1010.97454	674.31879	15
4	133.04301	690.30784	345.65756	230.77413	C-Carbamidomethyl	1963.92035	982.46381	655.31163	14
5	120.08078	837.37626	419.19177	279.79694	F	1803.88970	902.44849	601.96808	13
6	101.07094	965.43483	483.22105	322.48313	Q	1656.82129	828.91428	552.94528	12
7	101.07094	1093.49341	547.25034	365.16932	Q	1528.76271	764.88499	510.25909	11
8	60.04439	1180.52544	590.76636	394.18000	S	1400.70413	700.85570	467.57289	10
9	30.03383	1237.54690	619.27709	413.18715	G	1313.67210	657.33969	438.56222	9
10	181.06077	1445.59531	723.30129	482.53662	Y-Nitro	1256.65064	628.82896	419.55506	8
11	86.09643	1558.67937	779.84332	520.23131	L	1048.60223	524.80475	350.20560	7
12	86.09643	1671.76344	836.38536	557.92600	L	935.51817	468.26272	312.51091	6
13	87.05529	1785.80636	893.40682	595.94031	N	822.43410	411.72069	274.81622	5
14	87.05529	1899.84929	950.42828	633.95461	N	708.39118	354.69923	236.80191	4
15	44.04948	1970.88640	985.94684	657.63365	A	594.34825	297.67776	198.78760	3
16	120.04776	2117.92180	1059.46454	706.64545	M-Oxidation	523.31114	262.15921	175.10856	2
17	330.27026				K-TMT6plex	376.27574	188.64151	126.09676	1

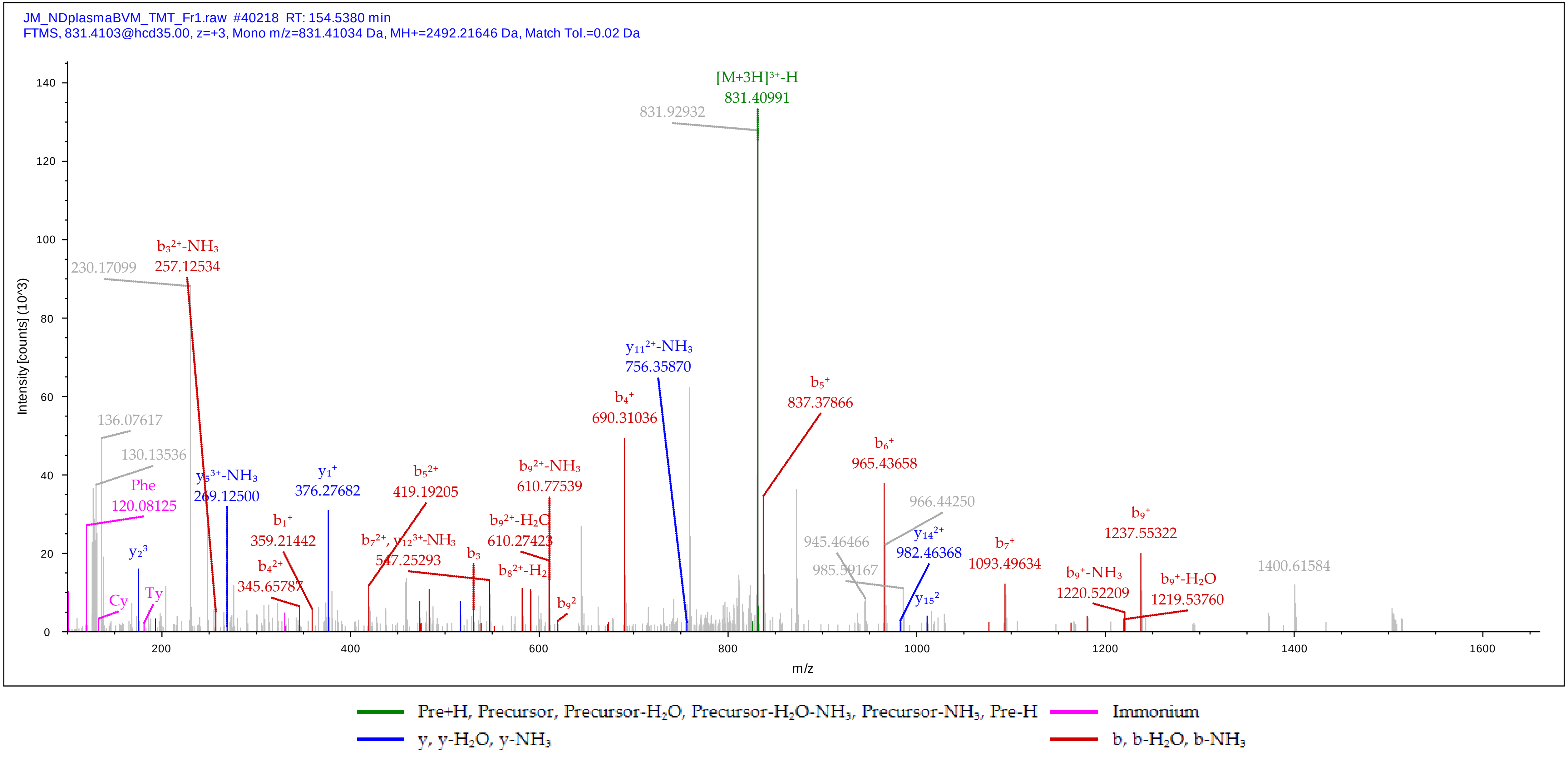

JM_NDplasmaBVM_TMT_Fr1.raw #40218 RT: 154.5380 min
FTMS, 831.4103@hcd35.00, z=+3, Mono m/z=831.41034 Da, MH+=2492.21646 Da, Match Tol.=0.02 Da
Intensity [counts] (10^3)
m/z
[M+3H]3+-H 831.40991
831.92932
230.17099
b3 2+-NH3 257.12534
y11 2+-NH3 756.35870
b4+ 690.31036
b5+ 837.37866
136.07617
130.13536
b6+ 965.43658
y5 3+-NH3 269.12500
y1+ 376.27682
b5 2+ 419.19205
b9 2+-NH3 610.77539
966.44250
Phe 120.08125
b1+ 359.21442
b7 2+, y12 3+-NH3 547.25293
b9 2+-H2O 610.27423
b3
945.46466
y14 2+ 982.46368
b7+ 1093.49634
b9+ 1237.55322
y2 3
b4 2+ 345.65787
b8 2+-H2
985.59167
b9+-NH3 1220.52209
b9+-H2O 1219.53760
1400.61584
Cy
Ty
b9 2
y15 2
Pre+H, Precursor, Precursor-H2O, Precursor-H2O-NH3, Precursor-NH3, Pre-H
Immonium
y, y-H2O, y-NH3
b, b-H2O, b-NH3

#1	Immonium	b^+	b^{2+}	b^{3+}	Seq.	y^+	y^{2+}	y^{3+}	#2
1	331.21789	359.21280	180.11004	120.40912	E-TMT6plex				33
2	101.07094	487.27138	244.13933	163.09531	Q	3732.74461	1866.87594	1244.91972	32
3	74.06004	588.31906	294.66317	196.77787	T	3604.68603	1802.84665	1202.23353	31
4	136.07569	751.38239	376.19483	251.13231	Y	3503.63835	1752.32281	1168.55097	30
5	87.05529	865.42531	433.21629	289.14662	N	3340.57502	1670.79115	1114.19653	29
6	74.06004	966.47299	483.74013	322.82918	T	3226.53210	1613.76969	1076.18222	28
7	86.09643	1079.55706	540.28217	360.52387	L	3125.48442	1563.24585	1042.49966	27
8	86.09643	1192.64112	596.82420	398.21856	L	3012.40035	1506.70382	1004.80497	26
9	133.04301	1352.67177	676.83952	451.56211	C-Carbamidomethyl	2899.31629	1450.16178	967.11028	25
10	70.06513	1449.72453	725.36590	483.91303	P	2739.28564	1370.14646	913.76673	24
11	101.07094	1577.78311	789.39519	526.59922	Q	2642.23288	1321.62008	881.41581	23
12	88.03930	1692.81005	846.90866	564.94154	D	2514.17430	1257.59079	838.72962	22
13	74.06004	1793.85773	897.43250	598.62409	T	2399.14736	1200.07732	800.38730	21
14	102.05496	1922.90032	961.95380	641.63829	E	2298.09968	1149.55348	766.70474	20
15	86.09643	2035.98439	1018.49583	679.33298	L	2169.05709	1085.03218	723.69055	19
16	101.07094	2164.04297	1082.52512	722.01917	Q	2055.97302	1028.49015	685.99586	18
17	88.03930	2279.06991	1140.03859	760.36149	D	1927.91444	964.46086	643.30967	17
18	87.05529	2393.11284	1197.06006	798.37580	N	1812.88750	906.94739	604.96735	16
19	204.07675	2624.17723	1312.59225	875.39726	W-Nitro	1698.84457	849.92593	566.95304	15
20	60.04439	2711.20925	1356.10827	904.40794	S	1467.78018	734.39373	489.93158	14
21	86.09643	2824.29332	1412.65030	942.10262	L	1380.74815	690.87772	460.92090	13
22	102.05496	2953.33591	1477.17159	985.11682	E	1267.66409	634.33568	423.22621	12
23	86.09643	3066.41998	1533.71363	1022.81151	L	1138.62150	569.81439	380.21202	11
24	70.06513	3163.47274	1582.24001	1055.16243	P	1025.53743	513.27236	342.51733	10
25	70.06513	3260.52550	1630.76639	1087.51335	P	928.48467	464.74597	310.16641	9
26	87.05529	3374.56843	1687.78785	1125.52766	N	831.43191	416.21959	277.81549	8
27	72.08078	3473.63684	1737.32206	1158.55047	V	717.38898	359.19813	239.80118	7
28	72.08078	3572.70526	1786.85627	1191.57327	V	618.32056	309.66392	206.77837	6
29	102.05496	3701.74785	1851.37756	1234.58747	E	519.25215	260.12971	173.75557	5
30	30.03383	3758.76931	1879.88830	1253.59462	G	390.20956	195.60842	130.74137	4
31	60.04439	3845.80134	1923.40431	1282.60530	S	333.18809	167.09769	111.73422	3
32	44.04948	3916.83846	1958.92287	1306.28434	A	246.15607	123.58167	82.72354	2
33	129.11347				R	175.11895	88.06311	59.04450	1

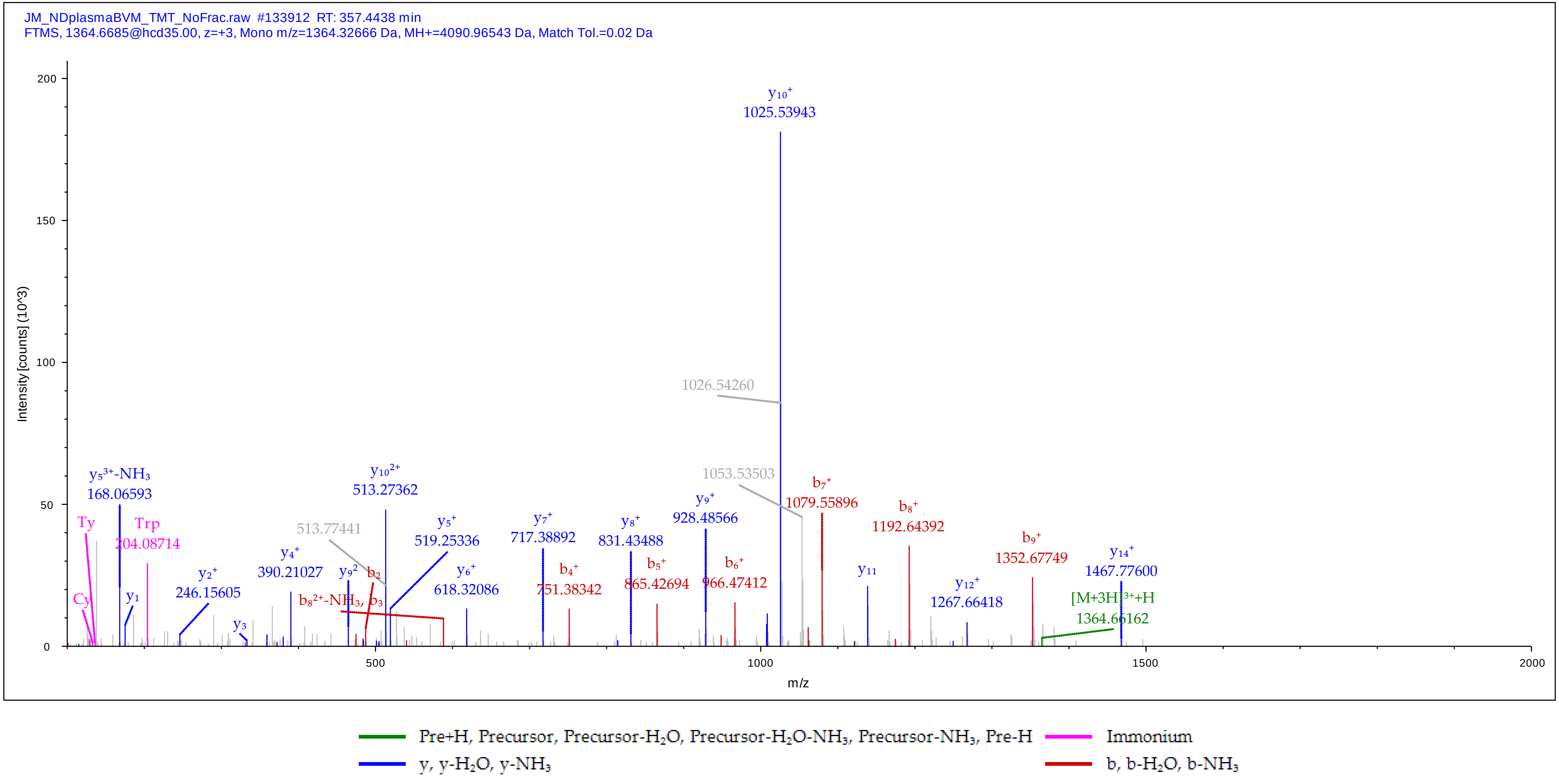
JM_NDplasmaBVM_TMT_NoFrac.raw #133912 RT: 357.4438 min
FTMS, 1364.6685@hcd35.00, z=+3, Mono m/z=1364.32666 Da, MH+=4090.96543 Da, Match Tol.=0.02 Da
Intensity [counts] (10^3)
m/z
y10+ 1025.53943
1026.54260
1053.53503
b7+ 1079.55896
b8+ 1192.64392
b9+ 1352.67749
y14+ 1467.77600
[M+3H]3+-H 1364.66162
y12+ 1267.66418
y11
y9+ 928.48566
b6+ 966.47412
y8+ 831.43488
b5+ 865.42694
y7+ 717.38892
b4+ 751.38342
y6+ 618.32086
y5+ 519.25336
y10 2+ 513.27362
513.77441
y9 2
b2
b8 2+-NH3, b3
y4+ 390.21027
y3
y2+ 246.15605
y5 3+-NH3 168.06593
y1
Ty
Trp 204.08714
Cy
Pre+H, Precursor, Precursor-H2O, Precursor-H2O-NH3, Precursor-NH3, Pre-H
Immonium
y, y-H2O, y-NH3
b, b-H2O, b-NH3

#1	Immonium	b⁺	b²⁺	b³⁺	Seq.	y⁺	y²⁺	y³⁺	#2
1	349.24371	377.23862	189.12295	126.41773	F-TMT6plex				31
2	133.04301	537.26927	269.13827	179.76127	Carbamidometl	3152.43128	1576.71928	1051.48194	30
3	101.07094	665.32785	333.16756	222.44747	Q	2992.40063	1496.70395	998.13839	29
4	102.05496	794.37044	397.68886	265.46166	E	2864.34205	1432.67466	955.45220	28
5	120.08078	941.43886	471.22307	314.48447	F	2735.29946	1368.15337	912.43800	27
6	101.07094	1069.49743	535.25235	357.17066	Q	2588.23104	1294.61916	863.41520	26
7	110.07127	1206.55634	603.78181	402.85697	H	2460.17247	1230.58987	820.72901	25
8	181.06077	1414.60475	707.80601	472.20643	Y-Nitro	2323.11356	1162.06042	775.04270	24
9	70.06513	1511.65751	756.33240	504.55736	P	2115.06515	1058.03621	705.69323	23
10	44.04948	1582.69463	791.85095	528.23639	A	2018.01238	1009.50983	673.34231	22
11	120.04776	1729.73003	865.36865	577.24819	M-Oxidation	1946.97527	973.99127	649.66327	21
12	30.03383	1786.75149	893.87938	596.25535	G	1799.93987	900.47357	600.65147	20
13	30.03383	1843.77296	922.39012	615.26250	G	1742.91841	871.96284	581.64432	19
14	72.08078	1942.84137	971.92432	648.28531	V	1685.89694	843.45211	562.63717	18
15	44.04948	2013.87848	1007.44288	671.96435	A	1586.82853	793.91790	529.61436	17
16	70.06513	2110.93125	1055.96926	704.31527	P	1515.79142	758.39935	505.93532	16
17	101.07094	2238.98982	1119.99855	747.00146	Q	1418.73865	709.87296	473.58440	15
18	44.04948	2310.02694	1155.51711	770.68050	A	1290.68008	645.84368	430.89821	14
19	86.09643	2423.11100	1212.05914	808.37519	L	1219.64296	610.32512	407.21917	13
20	44.04948	2494.14812	1247.57770	832.05422	A	1106.55890	553.78309	369.52448	12
21	72.08078	2593.21653	1297.11190	865.07703	V	1035.52178	518.26453	345.84545	11
22	44.04948	2664.25364	1332.63046	888.75607	A	936.45337	468.73032	312.82264	10
23	44.04948	2735.29076	1368.14902	912.43510	A	865.41626	433.21177	289.14360	9
24	60.04439	2822.32279	1411.66503	941.44578	S	794.37914	397.69321	265.46457	8
25	30.03383	2879.34425	1440.17576	960.45293	G	707.34711	354.17720	236.45389	7
26	70.06513	2976.39701	1488.70214	992.80386	P	650.32565	325.66646	217.44673	6
27	30.03383	3033.41848	1517.21288	1011.81101	G	553.27289	277.14008	185.09581	5
28	60.04439	3120.45051	1560.72889	1040.82169	S	496.25142	248.62935	166.08866	4
29	60.04439	3207.48253	1604.24491	1069.83236	S	409.21939	205.11334	137.07798	3
30	120.08078	3354.55095	1677.77911	1118.85517	F	322.18737	161.59732	108.06731	2
31	129.11347				R	175.11895	88.06311	59.04450	1

Nitro-Tyr immonium ion is detected in MS/MS spectra and added brown colored in the following spectrum

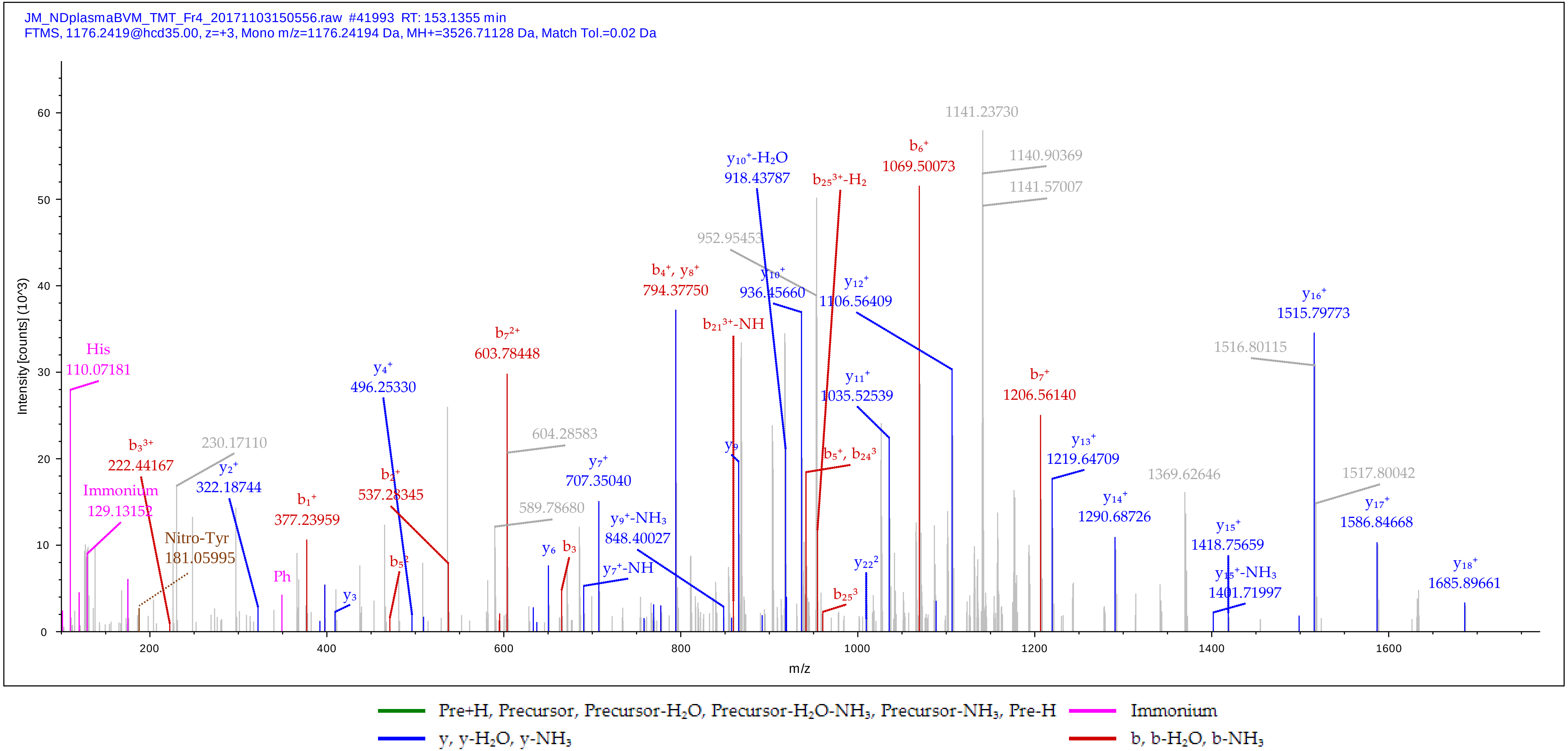
JM_NDplasmaBVM_TMT_Fr4_20171103150556.raw #41993 RT: 153.1355 min
FTMS, 1176.2419@hcd35.00, z=+3, Mono m/z=1176.24194 Da, MH+=3526.71128 Da, Match Tol.=0.02 Da
Intensity [counts] (10^3)
m/z
His 110.07181
Immonium 129.13152
b3 3+ 222.44167
230.17110
y2+ 322.18744
Nitro-Tyr 181.05995
Ph
b1+ 377.23959
y4+ 496.25330
b2+ 537.28345
b5 2
y3
b7 2+ 603.78448
604.28583
589.78680
y6
b3
y7+ 707.35040
y7+-NH
y9+-NH3 848.40027
b4+, y8+ 794.37750
b21 3+-NH
952.95453
y10+-H2O 918.43787
y10+ 936.45660
y9
b25 3+-H2
y12+ 1106.56409
y11+ 1035.52539
b5+, b24 3
y22 2
b25 3
b6+ 1069.50073
1141.23730
1140.90369
1141.57007
b7+ 1206.56140
y13+ 1219.64709
y14+ 1290.68726
1369.62646
y15+ 1418.75659
y15+-NH3 1401.71997
1516.80115
y16+ 1515.79773
1517.80042
y17+ 1586.84668
y18+ 1685.89661
Pre+H, Precursor, Precursor-H2O, Precursor-H2O-NH3, Precursor-NH3, Pre-H
Immonium
y, y-H2O, y-NH3
b, b-H2O, b-NH3

#1	Immonium	b+	b2+	b3+	b4+	Seq.	y+	y2+	y3+	y4+	#2
1	259.19676	287.19167	144.09947	96.40208	72.55338	G-TMT6plex					30
2	30.03383	344.21314	172.61021	115.40923	86.80874	G	3171.62421	1586.31574	1057.87959	793.66151	29
3	72.08078	443.28155	222.14441	148.43203	111.57584	V	3114.60274	1557.80501	1038.87243	779.40614	28
4	88.03930	558.30849	279.65788	186.77435	140.33258	D	3015.53433	1508.27080	1005.84963	754.63904	27
5	88.03930	673.33544	337.17136	225.11666	169.08932	D	2900.50739	1450.75733	967.50731	725.88230	26
6	102.05496	802.37803	401.69265	268.13086	201.34996	E	2785.48044	1393.24386	929.16500	697.12557	25
7	72.08078	901.44644	451.22686	301.15367	226.11707	V	2656.43785	1328.72256	886.15080	664.86492	24
8	74.06004	1002.49412	501.75070	334.83622	251.37899	T	2557.36944	1279.18836	853.12800	640.09782	23
9	86.09643	1115.57819	558.29273	372.53091	279.65000	L	2456.32176	1228.66452	819.44544	614.83590	22
10	60.04439	1202.61021	601.80874	401.54159	301.40801	S	2343.23769	1172.12248	781.75075	586.56488	21
11	44.04948	1273.64733	637.32730	425.22063	319.16729	A	2256.20566	1128.60647	752.74007	564.80687	20
12	181.06077	1481.69573	741.35151	494.57010	371.17939	Y-Nitro	2185.16855	1093.08791	729.06103	547.04760	19
13	86.09643	1594.77980	797.89354	532.26478	399.45041	I	1977.12014	989.06371	659.71157	495.03549	18
14	74.06004	1695.82748	848.41738	565.94734	424.71233	T	1864.03608	932.52168	622.01688	466.76448	17
15	86.09643	1808.91154	904.95941	603.64203	452.98334	I	1762.98840	881.99784	588.33432	441.50256	16
16	44.04948	1879.94865	940.47797	627.32107	470.74262	A	1649.90434	825.45581	550.63963	413.23154	15
17	86.09643	1993.03272	997.02000	665.01576	499.01364	L	1578.86722	789.93725	526.96059	395.47226	14
18	86.09643	2106.11678	1053.56203	702.71044	527.28465	L	1465.78316	733.39522	489.26590	367.20125	13
19	102.05496	2235.15937	1118.08333	745.72464	559.54530	E	1352.69910	676.85319	451.57122	338.93023	12
20	120.04776	2382.19477	1191.60103	794.73644	596.30415	M-Oxidation	1223.65650	612.33189	408.55702	306.66958	11
21	70.06513	2479.24754	1240.12741	827.08736	620.56734	P	1076.62110	538.81419	359.54522	269.91073	10
22	86.09643	2592.33160	1296.66944	864.78205	648.83836	I	979.56834	490.28781	327.19430	245.64754	9
23	70.06513	2689.38437	1345.19582	897.13297	673.10155	P	866.48428	433.74578	289.49961	217.37653	8
24	72.08078	2788.45278	1394.73003	930.15578	697.86865	V	769.43151	385.21939	257.14869	193.11334	7
25	74.06004	2889.50046	1445.25387	963.83834	723.13057	T	670.36310	335.68519	224.12588	168.34623	6
26	110.07127	3026.55937	1513.78332	1009.52464	757.39530	H	569.31542	285.16135	190.44332	143.08431	5
27	60.04439	3113.59140	1557.29934	1038.53532	779.15331	S	432.25651	216.63189	144.75702	108.81958	4
28	44.04948	3184.62851	1592.81789	1062.21436	796.91259	A	345.22448	173.11588	115.74634	87.06158	3
29	72.08078	3283.69693	1642.35210	1095.23716	821.67969	V	274.18737	137.59732	92.06731	69.30230	2
30	129.11347					R	175.11895	88.06311	59.04450	44.53520	1

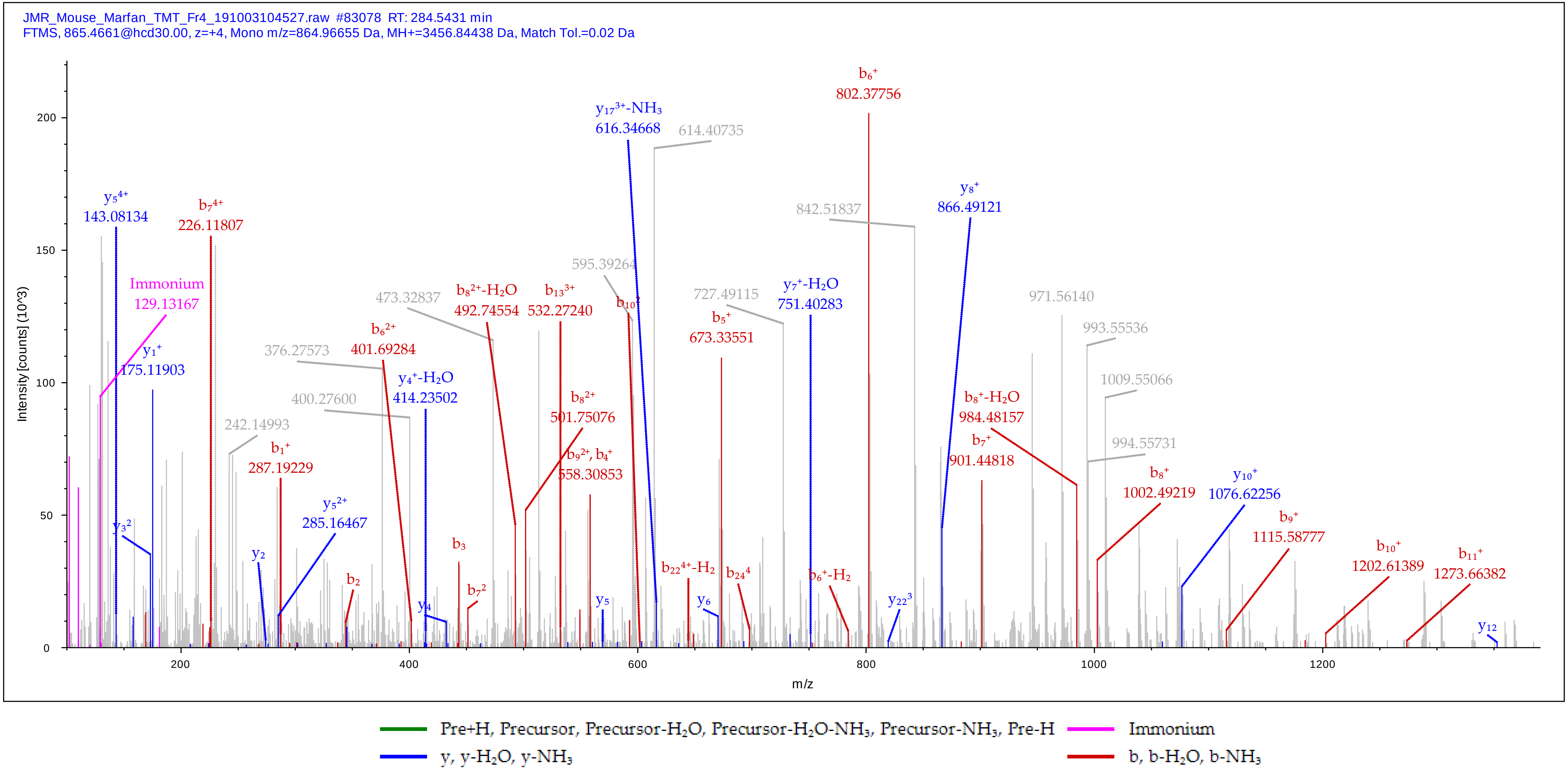

JMR_Mouse_Marfan_TMT_Fr4_191003104527.raw #83078 RT: 284.5431 min
FTMS, 865.4661@hcd30.00, z=+4, Mono m/z=864.96655 Da, MH+=3456.84438 Da, Match Tol.=0.02 Da
Intensity [counts] (10^3)
m/z
Immonium 129.13167
y5 4+ 143.08134
b7 4+ 226.11807
y1+ 175.11903
242.14993
b1+ 287.19229
y5 2+ 285.16467
376.27573
400.27600
473.32837
b6 2+ 401.69284
y4+-H2O 414.23502
b8 2+-H2O 492.74554
b13 3+ 532.27240
b8 2+ 501.75076
b9 2+, b4+ 558.30853
595.39264
y17 3+-NH3 616.34668
614.40735
727.49115
b5+ 673.33551
y7+-H2O 751.40283
b6+ 802.37756
842.51837
y8+ 866.49121
b8+-H2O 984.48157
b7+ 901.44818
971.56140
993.55536
1009.55066
994.55731
b8+ 1002.49219
y10+ 1076.62256
b9+ 1115.58777
b10+ 1202.61389
b11+ 1273.66382
Pre+H, Precursor, Precursor-H2O, Precursor-H2O-NH3, Precursor-NH3, Pre-H
Immonium
y, y-H2O, y-NH3
b, b-H2O, b-NH3

#1	Immonium	b^{+}	b^{2+}	b^{3+}	Seq.	y^{+}	y^{2+}	y^{3+}	#2
1	315.25936	343.25427	172.13077	115.08961	L-TMT6plex				13
2	60.04439	430.28630	215.64679	144.10028	S	1590.86889	795.93808	530.96115	12
3	70.06513	527.33906	264.17317	176.45121	P	1503.83686	752.42207	501.95047	11
4	101.07094	655.39764	328.20246	219.13740	Q	1406.78410	703.89569	469.59955	10
5	60.04439	742.42967	371.71847	248.14807	S	1278.72552	639.86640	426.91336	9
6	86.09643	855.51373	428.26051	285.84276	I	1191.69349	596.35038	397.90268	8
7	181.06077	1063.56214	532.28471	355.19223	Y-Nitro	1078.60943	539.80835	360.20799	7
8	87.05529	1177.60507	589.30617	393.20654	N	870.56102	435.78415	290.85852	6
9	86.09643	1290.68913	645.84820	430.90123	L	756.51809	378.76268	252.84421	5
10	86.09643	1403.77320	702.39024	468.59592	L	643.43403	322.22065	215.14953	4
11	70.06513	1500.82596	750.91662	500.94684	P	530.34996	265.67862	177.45484	3
12	30.03383	1557.84742	779.42735	519.95399	G	433.29720	217.15224	145.10392	2
13	330.27026				K-TMT6plex	376.27574	188.64151	126.09676	1

Nitro-Tyr immonium ion is detected in MS/MS spectra and added brown colored in the following spectrum

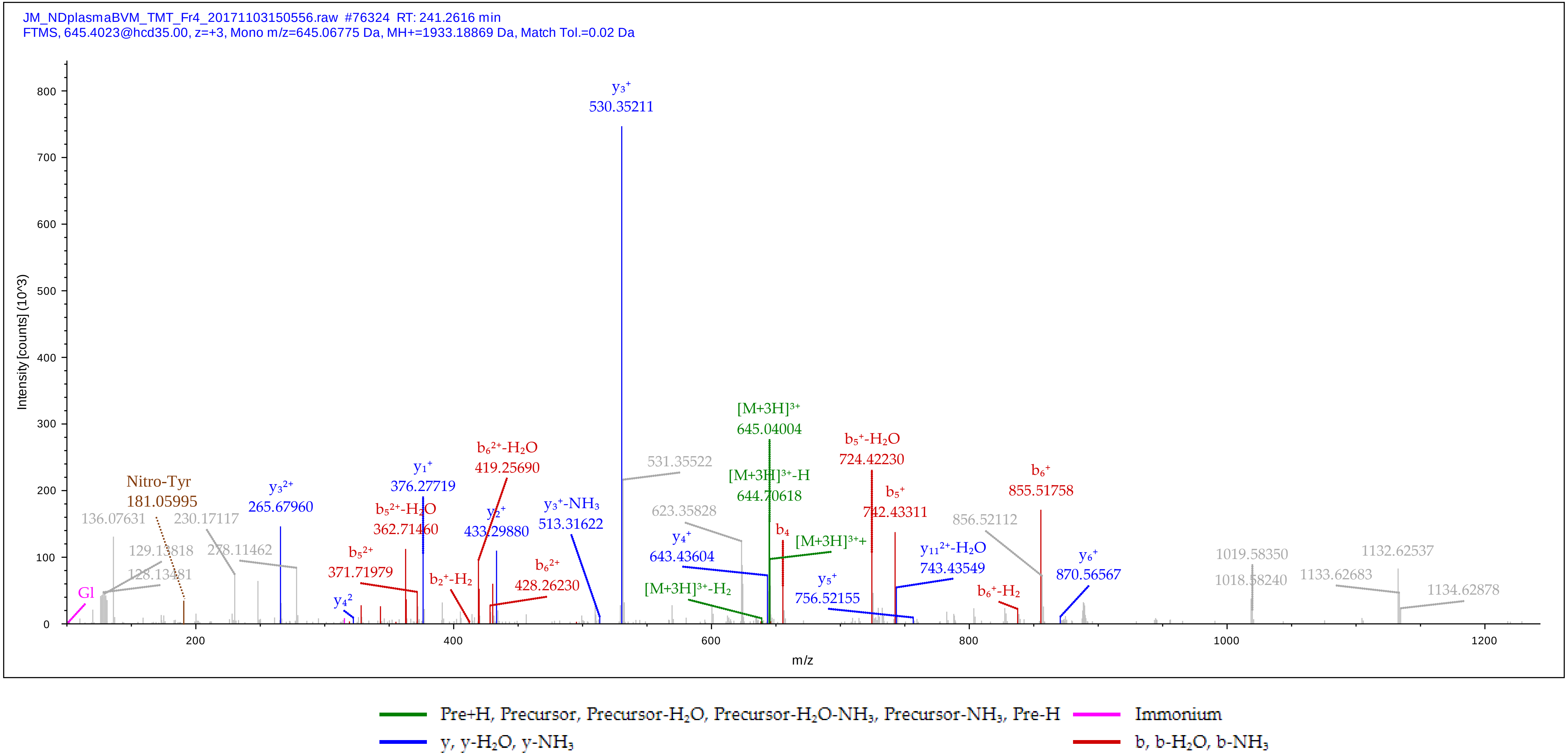
JM_NDplasmaBVM_TMT_Fr4_20171103150556.raw #76324 RT: 241.2616 min
FTMS, 645.4023@hcd35.00, z=+3, Mono m/z=645.06775 Da, MH+=1933.18869 Da, Match Tol.=0.02 Da
Intensity [counts] (10^3)
m/z
y3+ 530.35211
[M+3H]3+ 645.04004
[M+3H]3+-H 644.70618
b5+-H2O 724.42230
b6+ 855.51758
b5+ 742.43311
b6 2+-H2O 419.25690
y1+ 376.27719
y3 2+ 265.67960
Nitro-Tyr 181.05995
b5 2+-H2O 362.71460
y2+ 433.29880
y3+-NH3 513.31622
531.35522
623.35828
856.52112
y4+ 643.43604
b4
[M+3H]3+
y11 2+-H2O 743.43549
y6+ 870.56567
b5 2+ 371.71979
b2+-H2
b6 2+ 428.26230
[M+3H]3+-H2
y5+ 756.52155
b6+-H2
y4 2
Gl
136.07631
230.17117
129.13818
278.11462
128.13481
1019.58350
1018.58240
1132.62537
1133.62683
1134.62878
Pre+H, Precursor, Precursor-H2O, Precursor-H2O-NH3, Precursor-NH3, Pre-H
Immonium
y, y-H2O, y-NH3
b, b-H2O, b-NH3

#1	Immonium	b^{+}	b^{2+}	b^{3+}	b^{4+}	b^{5+}	Seq.	y^{+}	y^{2+}	y^{3+}	y^{4+}	y^{5+}	#2
1	303.22297	331.21789	166.11258	111.07748	83.55993	67.04940	T-TMT6plex						34
2	72.08078	430.28630	215.64679	144.10028	108.32703	86.86308	V	4195.09590	2098.05159	1399.03682	1049.52943	839.82500	33
3	101.07094	558.34488	279.67608	186.78648	140.34168	112.47480	Q	4096.02748	2048.51738	1366.01401	1024.76233	820.01132	32
4	30.03383	615.36634	308.18681	205.79363	154.59704	123.87909	G	3967.96891	1984.48809	1323.32782	992.74768	794.39960	31
5	44.04948	686.40346	343.70537	229.47267	172.35632	138.08651	A	3910.94744	1955.97736	1304.32067	978.49232	782.99531	30
6	120.08078	833.47187	417.23957	278.49547	209.12342	167.50020	F	3839.91033	1920.45880	1280.64163	960.73304	768.78789	29
7	120.08078	980.54028	490.77378	327.51828	245.89053	196.91388	F	3692.84192	1846.92460	1231.61882	923.96594	739.37420	28
8	30.03383	1037.56175	519.28451	346.52543	260.14589	208.31817	G	3545.77350	1773.39039	1182.59602	887.19883	709.96052	27
9	72.08078	1136.63016	568.81872	379.54824	284.91300	228.13185	V	3488.75204	1744.87966	1163.58886	872.94347	698.55623	26
10	70.06513	1233.68292	617.34510	411.89916	309.17619	247.54241	P	3389.68362	1695.34545	1130.56606	848.17636	678.74255	25
11	72.08078	1332.75134	666.87931	444.92196	333.94329	267.35609	V	3292.63086	1646.81907	1098.21514	823.91317	659.33199	24
12	181.06077	1540.79975	770.90351	514.27143	385.95539	308.96577	Y-Nitro	3193.56245	1597.28486	1065.19233	799.14607	639.51831	23
13	330.27026	1898.05764	949.53246	633.35740	475.26987	380.41735	K-TMT6plex	2985.51404	1493.26066	995.84286	747.13397	597.90863	22
14	88.03930	2013.08458	1007.04593	671.69971	504.02660	403.42274	D	2628.25615	1314.63171	876.75690	657.81949	526.45705	21
15	110.07127	2150.14349	1075.57539	717.38602	538.29133	430.83452	H	2513.22920	1257.11824	838.41459	629.06276	503.45166	20
16	102.05496	2279.18609	1140.09668	760.40021	570.55198	456.64304	E	2376.17029	1188.58878	792.72828	594.79803	476.03988	19
17	87.05529	2393.22902	1197.11815	798.41452	599.06271	479.45162	N	2247.12770	1124.06749	749.71408	562.53738	450.23136	18
18	133.04301	2553.25966	1277.13347	851.75807	639.07037	511.45775	C-Carbamidomethyl	2133.08477	1067.04602	711.69977	534.02665	427.42278	17
19	86.09643	2666.34373	1333.67550	889.45276	667.34139	534.07457	I	1973.05412	987.03070	658.35623	494.01899	395.41665	16
20	60.04439	2753.37576	1377.19152	918.46344	689.09940	551.48097	S	1859.97006	930.48867	620.66154	465.74797	372.79983	15
21	30.03383	2810.39722	1405.70225	937.47059	703.35476	562.88527	G	1772.93803	886.97265	591.65086	443.98996	355.39343	14
22	102.05496	2939.43981	1470.22354	980.48479	735.61541	588.69378	E	1715.91657	858.46192	572.64371	429.73460	343.98913	13
23	88.03930	3054.46676	1527.73702	1018.82710	764.37215	611.69917	D	1586.87397	793.94062	529.62951	397.47395	318.18062	12
24	86.09643	3167.55082	1584.27905	1056.52179	792.64316	634.31599	I	1471.84703	736.42715	491.28719	368.71721	295.17523	11
25	74.06004	3268.59850	1634.80289	1090.20435	817.90508	654.52552	T	1358.76297	679.88512	453.59251	340.44620	272.55841	10
26	110.07127	3405.65741	1703.33234	1135.89065	852.16981	681.93730	H	1257.71529	629.36128	419.90995	315.18428	252.34888	9
27	87.05529	3519.70034	1760.35381	1173.90496	880.68054	704.74589	N	1120.65638	560.83183	374.22364	280.91955	224.93710	8
28	30.03383	3576.72180	1788.86454	1192.91212	894.93591	716.15018	G	1006.61345	503.81036	336.20933	252.40882	202.12851	7
29	86.09643	3689.80586	1845.40657	1230.60681	923.20692	738.76699	I	949.59198	475.29963	317.20218	238.15345	190.72422	6
30	72.08078	3788.87428	1894.94078	1263.62961	947.97403	758.58068	V	836.50792	418.75760	279.50749	209.88244	168.10741	5
31	136.07569	3951.93761	1976.47244	1317.98405	988.73986	791.19334	Y	737.43951	369.22339	246.48469	185.11533	148.29372	4
32	74.06004	4052.98529	2026.99628	1351.66661	1014.00178	811.40288	T	574.37618	287.69173	192.13024	144.34950	115.68106	3
33	70.06513	4150.03805	2075.52266	1384.01753	1038.26497	830.81343	P	473.32850	237.16789	158.44768	119.08758	95.47152	2
34	330.27026						K-TMT6plex	376.27574	188.64151	126.09676	94.82439	76.06097	1

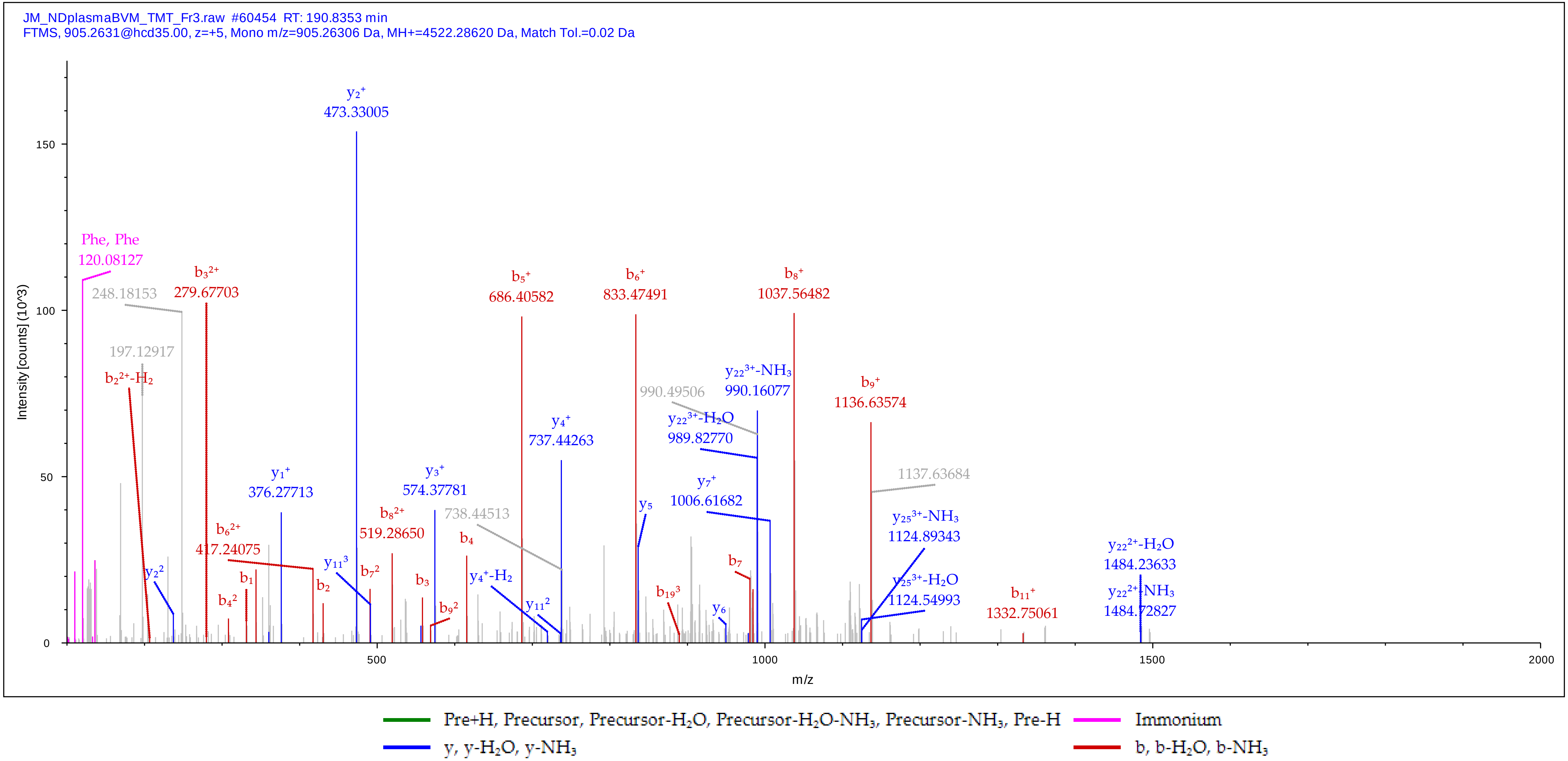
JM_NDplasmaBVM_TMT_Fr3.raw #60454 RT: 190.8353 min
FTMS, 905.2631@hcd35.00, z=+5, Mono m/z=905.26306 Da, MH+=4522.28620 Da, Match Tol.=0.02 Da
Intensity [counts] (10^3)
150
100
50
0
500
1000
1500
2000
m/z
Phe, Phe
120.08127
248.18153
197.12917
b_2^{2+}-H_2
b_3^{2+}
279.67703
y_2^+
473.33005
b_5^+
686.40582
b_6^+
833.47491
b_8^+
1037.56482
y_{22}^{3+}-NH_3
990.16077
990.49506
b_9^+
1136.63574
y_4^+
737.44263
y_{22}^{3+}-H_2O
989.82770
1137.63684
y_1^+
376.27713
y_3^+
574.37781
b_8^{2+}
519.28650
738.44513
y_5
y_7^+
1006.61682
y_{25}^{3+}-NH_3
1124.89343
y_{22}^{2+}-H_2O
1484.23633
b_6^{2+}
417.24075
b_4
y_{11}^3
b_7^2
b_7
y_2^2
b_1
b_2
b_3
y_4^+-H_2
y_{25}^{3+}-H_2O
1124.54993
b_{19}^3
b_{11}^+
1332.75061
y_{22}^{2+}-NH_3
1484.72827
b_4^2
b_9^2
y_{11}^2
y_6
Pre+H, Precursor, Precursor-H_2O, Precursor-H_2O-NH_3, Precursor-NH_3, Pre-H
Immonium
y, y-H_2O, y-NH_3
b, b-H_2O, b-NH_3

#1	Immonium	b^{+}	b^{2+}	b^{3+}	b^{4+}	Seq.	y^{+}	y^{2+}	y^{3+}	y^{4+}	#2
1	303.22297	331.21789	166.11258	111.07748	83.55993	T-TMT6plex					32
2	72.08078	430.28630	215.64679	144.10028	108.32703	V	3807.96276	1904.48502	1269.99244	952.74615	31
3	60.04439	517.31833	259.16280	173.11096	130.08504	S	3708.89435	1854.95081	1236.96963	927.97904	30
4	204.07675	748.38272	374.69500	250.13242	187.85114	W-Nitro	3621.86232	1811.43480	1207.95896	906.22104	29
5	44.04948	819.41983	410.21356	273.81146	205.61042	A	3390.79793	1695.90260	1130.93749	848.45494	28
6	72.08078	918.48825	459.74776	306.83427	230.37752	V	3319.76081	1660.38404	1107.25846	830.69566	27
7	74.06004	1019.53593	510.27160	340.51683	255.63944	T	3220.69240	1610.84984	1074.23565	805.92856	26
8	70.06513	1116.58869	558.79798	372.86775	279.90263	P	3119.64472	1560.32600	1040.55309	780.66664	25
9	330.27026	1473.84658	737.42693	491.95371	369.21710	K-TMT6plex	3022.59196	1511.79962	1008.20217	756.40345	24
10	60.04439	1560.87861	780.94294	520.96439	390.97511	S	2665.33406	1333.17067	889.11620	667.08897	23
11	86.09643	1673.96268	837.48498	558.65908	419.24613	L	2578.30203	1289.65465	860.10553	645.33097	22
12	30.03383	1730.98414	865.99571	577.66623	433.50149	G	2465.21797	1233.11262	822.41084	617.05995	21
13	102.05496	1860.02673	930.51701	620.68043	465.76214	E	2408.19651	1204.60189	803.40369	602.80458	20
14	72.08078	1959.09515	980.05121	653.70323	490.52924	V	2279.15391	1140.08059	760.38949	570.54394	19
15	87.05529	2073.13808	1037.07268	691.71754	519.03998	N	2180.08550	1090.54639	727.36668	545.77683	18
16	120.08078	2220.20649	1110.60688	740.74035	555.80708	F	2066.04257	1033.52492	689.35237	517.26610	17
17	74.06004	2321.25417	1161.13072	774.42291	581.06900	T	1918.97416	959.99072	640.32957	480.49900	16
18	44.04948	2392.29128	1196.64928	798.10194	598.82828	A	1817.92648	909.46688	606.64701	455.23708	15
19	74.06004	2493.33896	1247.17312	831.78450	624.09020	T	1746.88937	873.94832	582.96797	437.47780	14
20	44.04948	2564.37607	1282.69168	855.46354	641.84948	A	1645.84169	823.42448	549.28541	412.21588	13
21	102.05496	2693.41867	1347.21297	898.47774	674.11012	E	1574.80457	787.90592	525.60638	394.45660	12
22	44.04948	2764.45578	1382.73153	922.15678	691.86940	A	1445.76198	723.38463	482.59218	362.19595	11
23	86.09643	2877.53984	1439.27356	959.85147	720.14042	L	1374.72487	687.86607	458.91314	344.43667	10
24	101.07094	3005.59842	1503.30285	1002.53766	752.15506	Q	1261.64080	631.32404	421.21845	316.16566	9
25	60.04439	3092.63045	1546.81886	1031.54833	773.91307	S	1133.58222	567.29475	378.53226	284.15101	8
26	70.06513	3189.68321	1595.34525	1063.89926	798.17626	P	1046.55020	523.77874	349.52158	262.39301	7
27	102.05496	3318.72581	1659.86654	1106.91345	830.43691	E	949.49743	475.25235	317.17066	238.12982	6
28	86.09643	3431.80987	1716.40857	1144.60814	858.70793	L	820.45484	410.73106	274.15646	205.86917	5
29	133.04301	3591.84052	1796.42390	1197.95169	898.71559	C-Carbamidomethyl	707.37078	354.18903	236.46178	177.59815	4
30	30.03383	3648.86198	1824.93463	1216.95885	912.97095	G	547.34013	274.17370	183.11823	137.59049	3
31	87.05529	3762.90491	1881.95609	1254.97315	941.48169	N	490.31866	245.66297	164.11107	123.33512	2
32	330.27026					K-TMT6plex	376.27574	188.64151	126.09676	94.82439	1

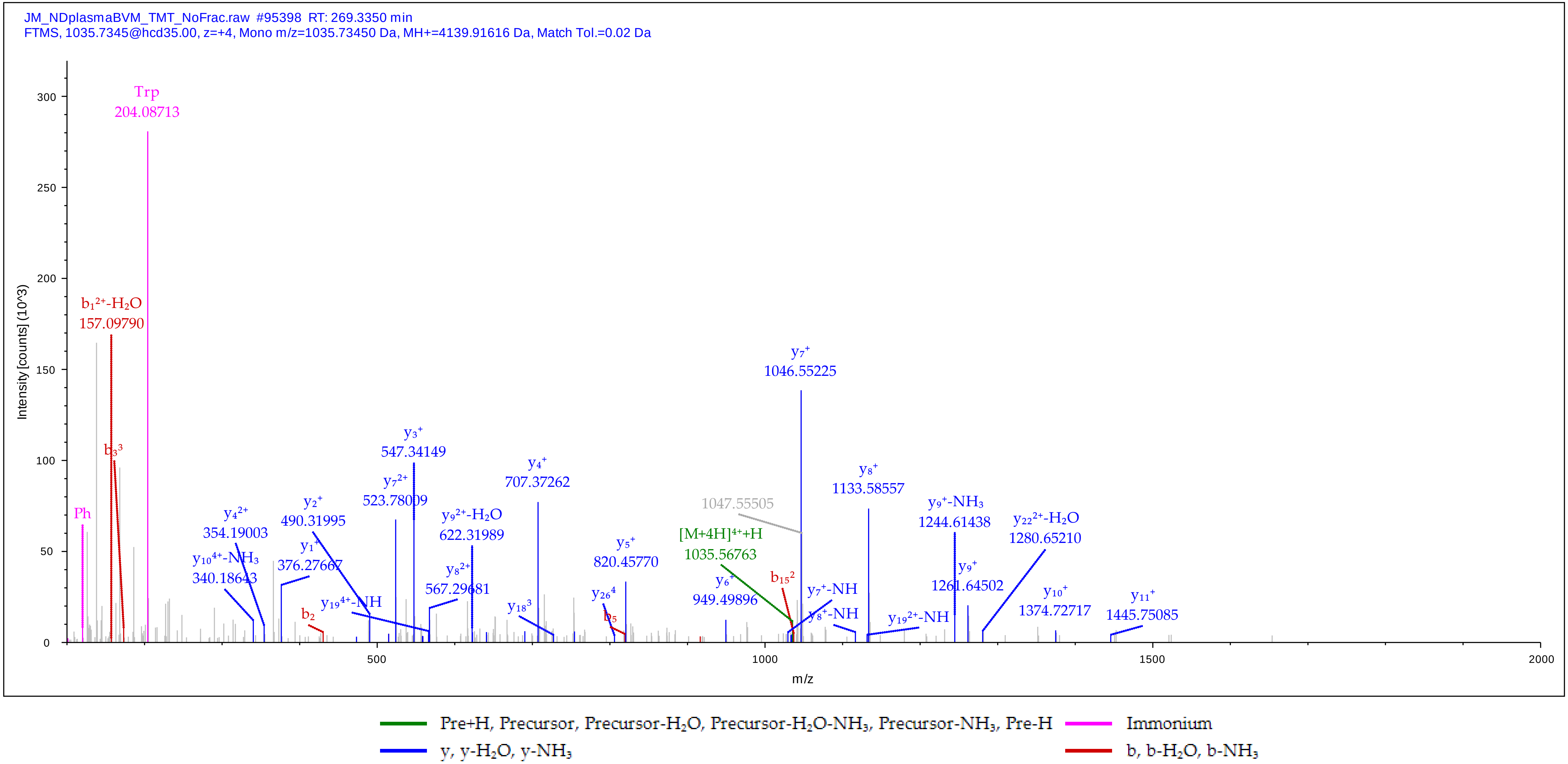
JM_NDplasmaBVM_TMT_NoFrac.raw #95398 RT: 269.3350 min
FTMS, 1035.7345@hcd35.00, z=+4, Mono m/z=1035.73450 Da, MH+=4139.91616 Da, Match Tol.=0.02 Da
Intensity [counts] (10^3)
m/z
Trp 204.08713
b1 2+-H2O 157.09790
b3 3
Ph
y4 2+ 354.19003
y10 4+-NH3 340.18643
y2+ 490.31995
y1+ 376.27667
b2
y19 4+-NH
y3+ 547.34149
y7 2+ 523.78009
y9 2+-H2O 622.31989
y8 2+ 567.29681
y4+ 707.37262
y18 3
y26 4
b5
y5+ 820.45770
1047.55505
[M+4H]4+ +H 1035.56763
y6+ 949.49896
b15 2
y7+ 1046.55225
y7+-NH
y8+-NH
y8+ 1133.58557
y19 2+-NH
y9+-NH3 1244.61438
y9+ 1261.64502
y22 2+-H2O 1280.65210
y10+ 1374.72717
y11+ 1445.75085
Pre+H, Precursor, Precursor-H2O, Precursor-H2O-NH3, Precursor-NH3, Pre-H
Immonium
y, y-H2O, y-NH3
b, b-H2O, b-NH3

#1	Immonium	b^{+}	b^{2+}	b^{3+}	b^{4+}	b^{5+}	Seq.	y^{+}	y^{2+}	y^{3+}	y^{4+}	y^{5+}	#2
1	315.25936	343.25427	172.13077	115.08961	86.56903	69.45668	I-TMT6plex						48
2	72.08078	442.32269	221.66498	148.11241	111.33613	89.27036	V	5290.44272	2645.72500	1764.15243	1323.36614	1058.89437	47
3	87.05529	556.36561	278.68645	186.12672	139.84686	112.07894	N	5191.37431	2596.19079	1731.12962	1298.59904	1039.08068	46
4	30.03383	613.38708	307.19718	205.13388	154.10223	123.48324	G	5077.33138	2539.16933	1693.11531	1270.08830	1016.27210	45
5	102.05496	742.42967	371.71847	248.14807	186.36288	149.29176	E	5020.30992	2510.65860	1674.10816	1255.83294	1004.86781	44
6	44.04948	813.46678	407.23703	271.82711	204.12215	163.49918	A	4891.26733	2446.13730	1631.09396	1223.57229	979.05929	43
7	88.03930	928.49373	464.75050	310.16943	232.87889	186.50457	D	4820.23021	2410.61874	1607.41492	1205.81301	964.85186	42
8	44.04948	999.53084	500.26906	333.84846	250.63817	200.71199	A	4705.20327	2353.10527	1569.07261	1177.05627	941.84648	41
9	104.05285	1130.57133	565.78930	377.52863	283.39829	226.92009	M	4634.16616	2317.58672	1545.39357	1159.29700	927.63905	40
10	74.06004	1231.61900	616.31314	411.21119	308.66021	247.12962	T	4503.12567	2252.06647	1501.71341	1126.53688	901.43096	39
11	86.09643	1344.70307	672.85517	448.90587	336.93122	269.74643	L	4402.07799	2201.54263	1468.03085	1101.27496	881.22142	38
12	88.03930	1459.73001	730.36864	487.24819	365.68796	292.75182	D	4288.99393	2145.00060	1430.33616	1073.00394	858.60461	37
13	30.03383	1516.75147	758.87938	506.25534	379.94333	304.15612	G	4173.96699	2087.48713	1391.99385	1044.24720	835.59922	36
14	30.03383	1573.77294	787.39011	525.26250	394.19869	315.56041	G	4116.94552	2058.97640	1372.98669	1029.99184	824.19493	35
15	110.07127	1710.83185	855.91956	570.94880	428.46342	342.97219	H	4059.92406	2030.46567	1353.97954	1015.73647	812.79063	34
16	44.04948	1781.86896	891.43812	594.62784	446.22270	357.17961	A	3922.86515	1961.93621	1308.29323	981.47174	785.37885	33
17	136.07569	1944.93229	972.96978	648.98228	486.98853	389.79228	Y	3851.82803	1926.41765	1284.61420	963.71247	771.17143	32
18	86.09643	2058.01636	1029.51182	686.67697	515.25955	412.40909	I	3688.76470	1844.88599	1230.25975	922.94663	738.55876	31
19	44.04948	2129.05347	1065.03037	710.35601	533.01882	426.61652	A	3575.68064	1788.34396	1192.56506	894.67562	715.94195	30
20	30.03383	2186.07493	1093.54111	729.36316	547.27419	438.02081	G	3504.64353	1752.82540	1168.88603	876.91634	701.73453	29
21	101.07094	2314.13351	1157.57039	772.04935	579.28884	463.63252	Q	3447.62206	1724.31467	1149.87887	862.66097	690.33023	28
22	133.04301	2474.16416	1237.58572	825.39290	619.29650	495.63865	C-Carbamidomethyl	3319.56349	1660.28538	1107.19268	830.64633	664.71852	27
23	30.03383	2531.18562	1266.09645	844.40006	633.55186	507.04295	G	3159.53284	1580.27006	1053.84913	790.63867	632.71239	26
24	86.09643	2644.26969	1322.63848	882.09475	661.82288	529.65976	L	3102.51137	1551.75933	1034.84198	776.38330	621.30810	25
25	72.08078	2743.33810	1372.17269	915.11755	686.58998	549.47344	V	2989.42731	1495.21729	997.14729	748.11228	598.69128	24
26	70.06513	2840.39086	1420.69907	947.46847	710.85317	568.88399	P	2890.35890	1445.68309	964.12448	723.34518	578.87760	23
27	72.08078	2939.45928	1470.23328	980.49128	735.62028	588.69768	V	2793.30613	1397.15670	931.77356	699.08199	559.46705	22
28	104.05285	3070.49976	1535.75352	1024.17144	768.38040	614.90577	M	2694.23772	1347.62250	898.75076	674.31489	539.65336	21
29	44.04948	3141.53688	1571.27208	1047.85048	786.13968	629.11320	A	2563.19723	1282.10226	855.07060	641.55477	513.44527	20
30	102.05496	3270.57947	1635.79337	1090.86467	818.40032	654.92172	E	2492.16012	1246.58370	831.39156	623.79549	499.23785	19
31	181.06077	3478.62788	1739.81758	1160.21414	870.41243	696.53140	Y-Nitro	2363.11753	1182.06240	788.37736	591.53484	473.42933	18
32	136.07569	3641.69120	1821.34924	1214.56859	911.17826	729.14406	Y	2155.06912	1078.03820	719.02789	539.52274	431.81965	17
33	102.05496	3770.73380	1885.87054	1257.58278	943.43891	754.95258	E	1992.00579	996.50653	664.67345	498.75691	399.20698	16
34	60.04439	3857.76583	1929.38655	1286.59346	965.19691	772.35899	S	1862.96320	931.98524	621.65925	466.49626	373.39846	15
35	60.04439	3944.79785	1972.90257	1315.60414	986.95492	789.76539	S	1775.93117	888.46922	592.64857	444.73825	355.99206	14
36	87.05529	4058.84078	2029.92403	1353.61845	1015.46565	812.57398	N	1688.89914	844.95321	563.63790	422.98024	338.58565	13
37	133.04301	4218.87143	2109.93935	1406.96199	1055.47332	844.58011	C-Carbamidomethyl	1574.85621	787.93175	525.62359	394.46951	315.77706	12
38	44.04948	4289.90854	2145.45791	1430.64103	1073.23259	858.78753	A	1414.82557	707.91642	472.28004	354.46185	283.77093	11
39	86.09643	4402.99261	2201.99994	1468.33572	1101.50361	881.40434	I	1343.78845	672.39786	448.60100	336.70257	269.56351	10
40	70.06513	4500.04537	2250.52632	1500.68664	1125.76680	900.81490	P	1230.70439	615.85583	410.90631	308.43155	246.94670	9
41	60.04439	4587.07740	2294.04234	1529.69732	1147.52481	918.22130	S	1133.65162	567.32945	378.55539	284.16836	227.53615	8
42	101.07094	4715.13598	2358.07163	1572.38351	1179.53945	943.83302	Q	1046.61960	523.81344	349.54472	262.41036	210.12974	7
43	101.07094	4843.19456	2422.10092	1615.06970	1211.55410	969.44473	Q	918.56102	459.78415	306.85852	230.39571	184.51803	6
44	30.03383	4900.21602	2450.61165	1634.07686	1225.80946	980.84903	G	790.50244	395.75486	264.17233	198.38107	158.90631	5
45	86.09643	5013.30008	2507.15368	1671.77155	1254.08048	1003.46584	I	733.48098	367.24413	245.16518	184.12570	147.50202	4
46	120.08078	5160.36850	2580.68789	1720.79435	1290.84758	1032.87952	F	620.39691	310.70210	207.47049	155.85469	124.88520	3
47	70.06513	5257.42126	2629.21427	1753.14527	1315.11077	1052.29007	P	473.32850	237.16789	158.44768	119.08758	95.47152	2
48	330.27026						K-TMT6plex	376.27574	188.64151	126.09676	94.82439	76.06097	1

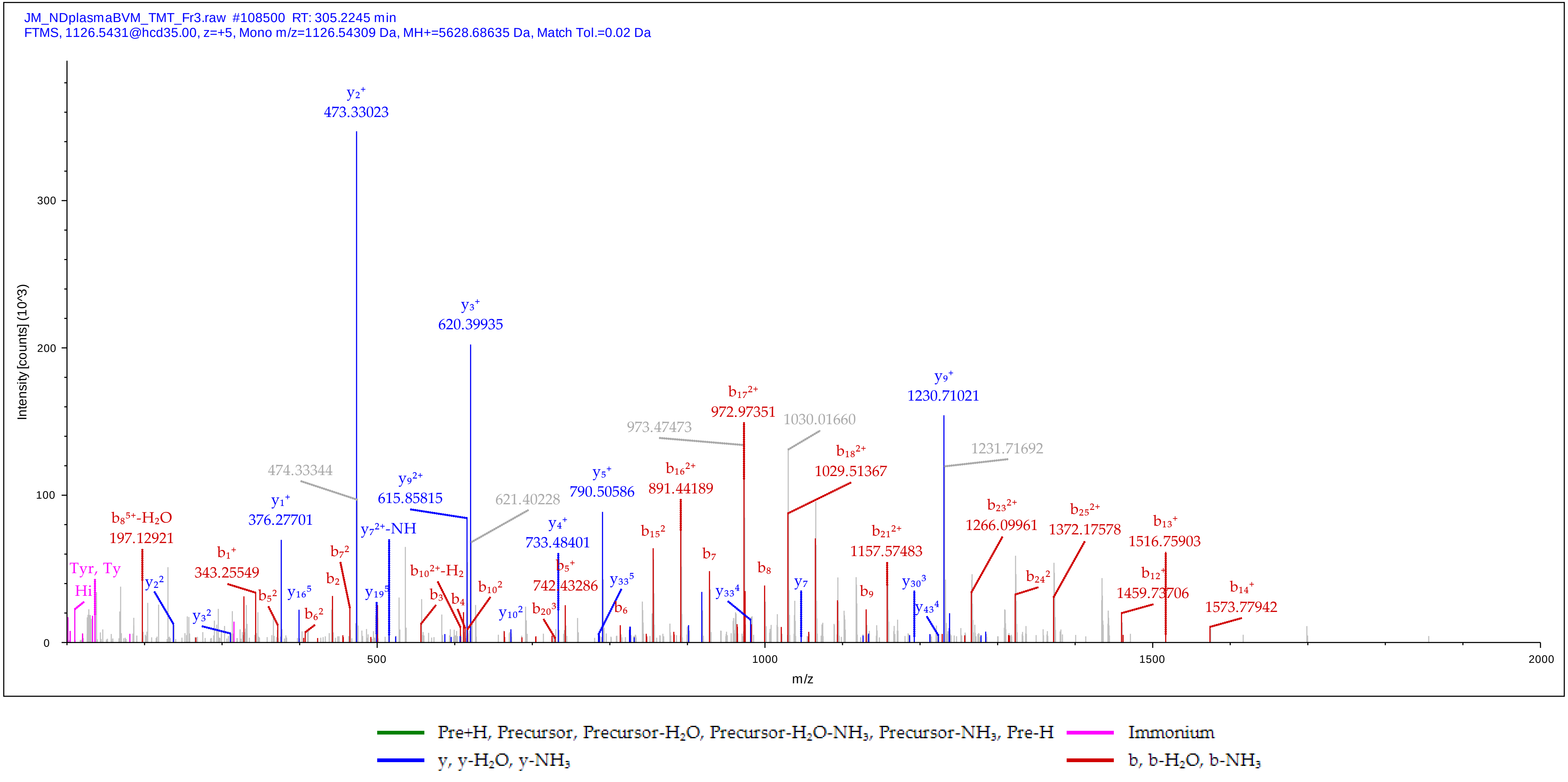

JM_NDplasmaBVM_TMT_Fr3.raw #108500 RT: 305.2245 min
FTMS, 1126.5431@hcd35.00, z=+5, Mono m/z=1126.54309 Da, MH+=5628.68635 Da, Match Tol.=0.02 Da
Intensity [counts] (10^3)
m/z
y2+ 473.33023
y3+ 620.39935
y9+ 1230.71021
b17 2+ 972.97351
973.47473
1030.01660
1231.71692
b18 2+ 1029.51367
474.33344
y9 2+ 615.85815
621.40228
y5+ 790.50586
b16 2+ 891.44189
y1+ 376.27701
b8 5+-H2O 197.12921
y7 2+-NH
y4+ 733.48401
b1+ 343.25549
b5+ 742.43286
b23 2+ 1266.09961
b25 2+ 1372.17578
b13+ 1516.75903
b21 2+ 1157.57483
b12+ 1459.73706
b14+ 1573.77942
Tyr, Ty
Hi
Pre+H, Precursor, Precursor-H2O, Precursor-H2O-NH3, Precursor-NH3, Pre-H
Immonium
y, y-H2O, y-NH3
b, b-H2O, b-NH3

#1	Immonium	b⁺	b²⁺	b³⁺	Seq.	y⁺	y²⁺	y³⁺	#2
1	362.20594	390.20086	195.60407	130.73847	C-TMT6plex-Carbamidomethyl				25
2	133.04301	550.23151	275.61939	184.08202	C-Carbamidomethyl	2790.19029	1395.59878	930.73495	24
3	60.04439	637.26353	319.13541	213.09270	S	2630.15964	1315.58346	877.39140	23
4	86.09643	750.34760	375.67744	250.78738	I	2543.12761	1272.06744	848.38072	22
5	87.05529	864.39053	432.69890	288.80169	N	2430.04355	1215.52541	810.68603	21
6	60.04439	951.42255	476.21492	317.81237	S	2316.00062	1158.50395	772.67172	20
7	70.06513	1048.47532	524.74130	350.16329	P	2228.96859	1114.98793	743.66105	19
8	70.06513	1145.52808	573.26768	382.51421	P	2131.91583	1066.46155	711.31013	18
9	86.09643	1258.61215	629.80971	420.20890	L	2034.86306	1017.93517	678.95921	17
10	181.06077	1466.66055	733.83391	489.55837	Y-Nitro	1921.77900	961.39314	641.26452	16
11	133.04301	1626.69120	813.84924	542.90192	C-Carbamidomethyl	1713.73059	857.36893	571.91505	15
12	60.04439	1713.72323	857.36525	571.91259	S	1553.69994	777.35361	518.57150	14
13	60.04439	1800.75526	900.88127	600.92327	S	1466.66792	733.83760	489.56082	13
14	101.07094	1928.81383	964.91056	643.60946	Q	1379.63589	690.32158	460.55015	12
15	86.09643	2041.89790	1021.45259	681.30415	I	1251.57731	626.29229	417.86395	11
16	88.03930	2156.92484	1078.96606	719.64646	D	1138.49325	569.75026	380.16927	10
17	44.04948	2227.96195	1114.48462	743.32550	A	1023.46630	512.23679	341.82695	9
18	102.05496	2357.00455	1179.00591	786.33970	E	952.42919	476.71823	318.14791	8
19	120.04776	2504.03995	1252.52361	835.35150	M-Oxidation	823.38660	412.19694	275.13372	7
20	86.09643	2617.12401	1309.06564	873.04619	I	676.35120	338.67924	226.12192	6
21	88.03930	2732.15095	1366.57912	911.38850	D	563.26713	282.13720	188.42723	5
22	74.06004	2833.19863	1417.10295	945.07106	T	448.24019	224.62373	150.08491	4
23	86.09643	2946.28270	1473.64499	982.76575	L	347.19251	174.09989	116.40235	3
24	101.07094	3074.34127	1537.67428	1025.45194	Q	234.10845	117.55786	78.70767	2
25	60.04439				S	106.04987	53.52857	36.02147	1

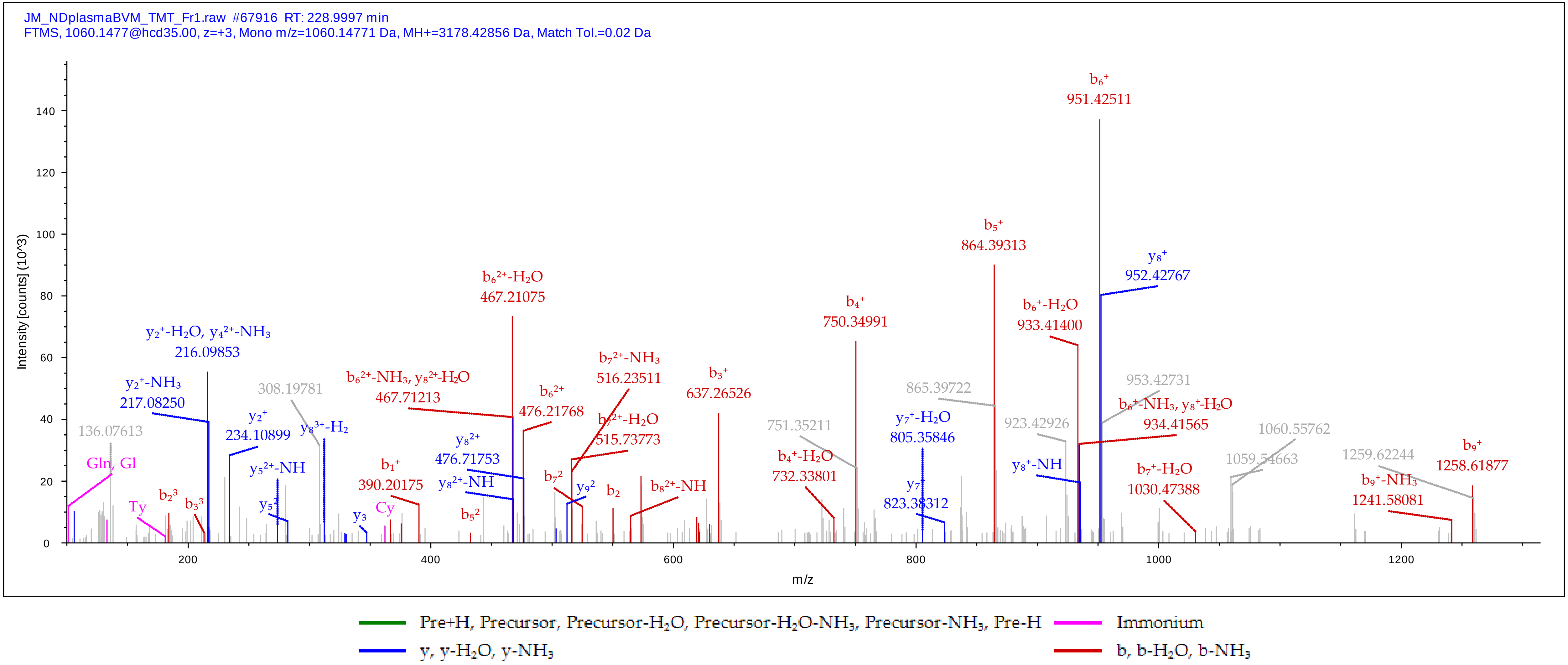

JM_NDplasmaBVM_TMT_Fr1.raw #67916 RT: 228.9997 min
FTMS, 1060.1477@hcd35.00, z=+3, Mono m/z=1060.14771 Da, MH+=3178.42856 Da, Match Tol.=0.02 Da
Intensity [counts] (10^3)
m/z
b6+ 951.42511
b5+ 864.39313
y8+ 952.42767
b6+-H2O 933.41400
b4+ 750.34991
b6 2+-H2O 467.21075
y2+-H2O, y4 2+-NH3 216.09853
y2+-NH3 217.08250
308.19781
b6 2+-NH3, y8 2+-H2O 467.71213
b7 2+-NH3 516.23511
b3+ 637.26526
865.39722
953.42731
b6 2+ 476.21768
b6+-NH3, y8+-H2O 934.41565
136.07613
y2+ 234.10899
y8 3+-H2
b7 2+-H2O 515.73773
751.35211
y7+-H2O 805.35846
923.42926
1060.55762
y8 2+ 476.71753
b4+-H2O 732.33801
1059.54663
1259.62244
b9+ 1258.61877
Gln, Gl
y5 2+-NH
b1+ 390.20175
y8 2+-NH
b7 2
y9 2
b2
b8 2+-NH
y8+-NH
b7+-H2O 1030.47388
y7+ 823.38312
b9+-NH3 1241.58081
b2 3
b3 3
Ty
y5 2
Cy
y3
b5 2
Pre+H, Precursor, Precursor-H2O, Precursor-H2O-NH3, Precursor-NH3, Pre-H
Immonium
y, y-H2O, y-NH3
b, b-H2O, b-NH3

#1	Immonium	b^+	b^{2+}	b^{3+}	b^{4+}	b^{5+}	Seq.	y^+	y^{2+}	y^{3+}	y^{4+}	y^{5+}	#2
1	349.24371	377.23862	189.12295	126.41773	95.06511	76.25355	F-TMT6plex						32
2	102.05496	506.28122	253.64425	169.43192	127.32576	102.06206	E	4023.79668	2012.40198	1341.93708	1006.70463	805.56516	31
3	102.05496	635.32381	318.16554	212.44612	159.58641	127.87058	E	3894.75408	1947.88068	1298.92288	974.44398	779.75664	30
4	133.04301	795.35446	398.18087	265.78967	199.59407	159.87671	C-Carbamidomethyl	3765.71149	1883.35938	1255.90868	942.18333	753.94812	29
5	133.04301	955.38511	478.19619	319.13322	239.60173	191.88284	C-Carbamidomethyl	3605.68084	1803.34406	1202.56513	902.17567	721.94199	28
6	101.07094	1083.44368	542.22548	361.81941	271.61638	217.49456	Q	3445.65019	1723.32873	1149.22158	862.16801	689.93586	27
7	102.05496	1212.48628	606.74678	404.83361	303.87703	243.30308	E	3317.59161	1659.29945	1106.53539	830.15336	664.32414	26
8	87.05529	1326.52920	663.76824	442.84792	332.38776	266.11166	N	3188.54902	1594.77815	1063.52119	797.89271	638.51563	25
9	74.06004	1427.57688	714.29208	476.53048	357.64968	286.32120	T	3074.50609	1537.75669	1025.50688	769.38198	615.70704	24
10	70.06513	1524.62965	762.81846	508.88140	381.91287	305.73175	P	2973.45842	1487.23285	991.82432	744.12006	595.49750	23
11	104.05285	1655.67013	828.33870	552.56156	414.67299	331.93985	M	2876.40565	1438.70646	959.47340	719.85687	576.08695	22
12	87.05529	1769.71306	885.36017	590.57587	443.18372	354.74843	N	2745.36517	1373.18622	915.79324	687.09675	549.87885	21
13	86.09643	1882.79712	941.90220	628.27056	471.45474	377.36525	I	2631.32224	1316.16476	877.77893	658.58602	527.07027	20
14	120.08078	2029.86554	1015.43641	677.29336	508.22184	406.77893	F	2518.23818	1259.62273	840.08424	630.31500	504.45346	19
15	104.05285	2160.90602	1080.95665	720.97352	540.98196	432.98703	M	2371.16976	1186.08852	791.06144	593.54790	475.03977	18
16	133.04301	2320.93667	1160.97197	774.31707	580.98962	464.99315	C-Carbamidomethyl	2240.12928	1120.56828	747.38128	560.78778	448.83168	17
17	74.06004	2421.98435	1211.49581	807.99963	606.25154	485.20269	T	2080.09863	1040.55295	694.03773	520.78011	416.82555	16
18	181.06077	2630.03275	1315.52001	877.34910	658.26365	526.81237	Y-Nitro	1979.05095	990.02911	660.35517	495.51820	396.61601	15
19	120.08078	2777.10117	1389.05422	926.37191	695.03075	556.22605	F	1771.00254	886.00491	591.00570	443.50609	355.00633	14
20	120.04776	2924.13657	1462.57192	975.38371	731.78960	585.63313	M-Oxidation	1623.93413	812.47070	541.98289	406.73899	325.59265	13
21	70.06513	3021.18933	1511.09830	1007.73463	756.05279	605.04369	P	1476.89873	738.95300	492.97109	369.98014	296.18557	12
22	44.04948	3092.22644	1546.61686	1031.41367	773.81207	619.25111	A	1379.84597	690.42662	460.62017	345.71695	276.77501	11
23	44.04948	3163.26356	1582.13542	1055.09270	791.57135	633.45853	A	1308.80885	654.90807	436.94114	327.95767	262.56759	10
24	102.05496	3292.30615	1646.65671	1098.10690	823.83200	659.26705	E	1237.77174	619.38951	413.26210	310.19839	248.36017	9
25	70.06513	3389.35891	1695.18310	1130.45782	848.09519	678.67760	P	1108.72915	554.86821	370.24790	277.93774	222.55165	8
26	86.09643	3502.44298	1751.72513	1168.15251	876.36620	701.29442	L	1011.67638	506.34183	337.89698	253.67455	203.14110	7
27	101.07094	3630.50156	1815.75442	1210.83870	908.38085	726.90613	Q	898.59232	449.79980	300.20229	225.40354	180.52429	6
28	86.09643	3743.58562	1872.29645	1248.53339	936.65186	749.52295	L	770.53374	385.77051	257.51610	193.38889	154.91257	5
29	70.06513	3840.63838	1920.82283	1280.88431	960.91505	768.93350	P	657.44968	329.22848	219.82141	165.11788	132.29576	4
30	44.04948	3911.67550	1956.34139	1304.56335	978.67433	783.14092	A	560.39691	280.70210	187.47049	140.85469	112.88520	3
31	86.09643	4024.75956	2012.88342	1342.25804	1006.94535	805.75773	I	489.35980	245.18354	163.79145	123.09541	98.67778	2
32	330.27026						K-TMT6plex	376.27574	188.64151	126.09676	94.82439	76.06097	1

Nitro-Tyr immonium ion is detected in MS/MS spectra and added brown colored in the following spectrum

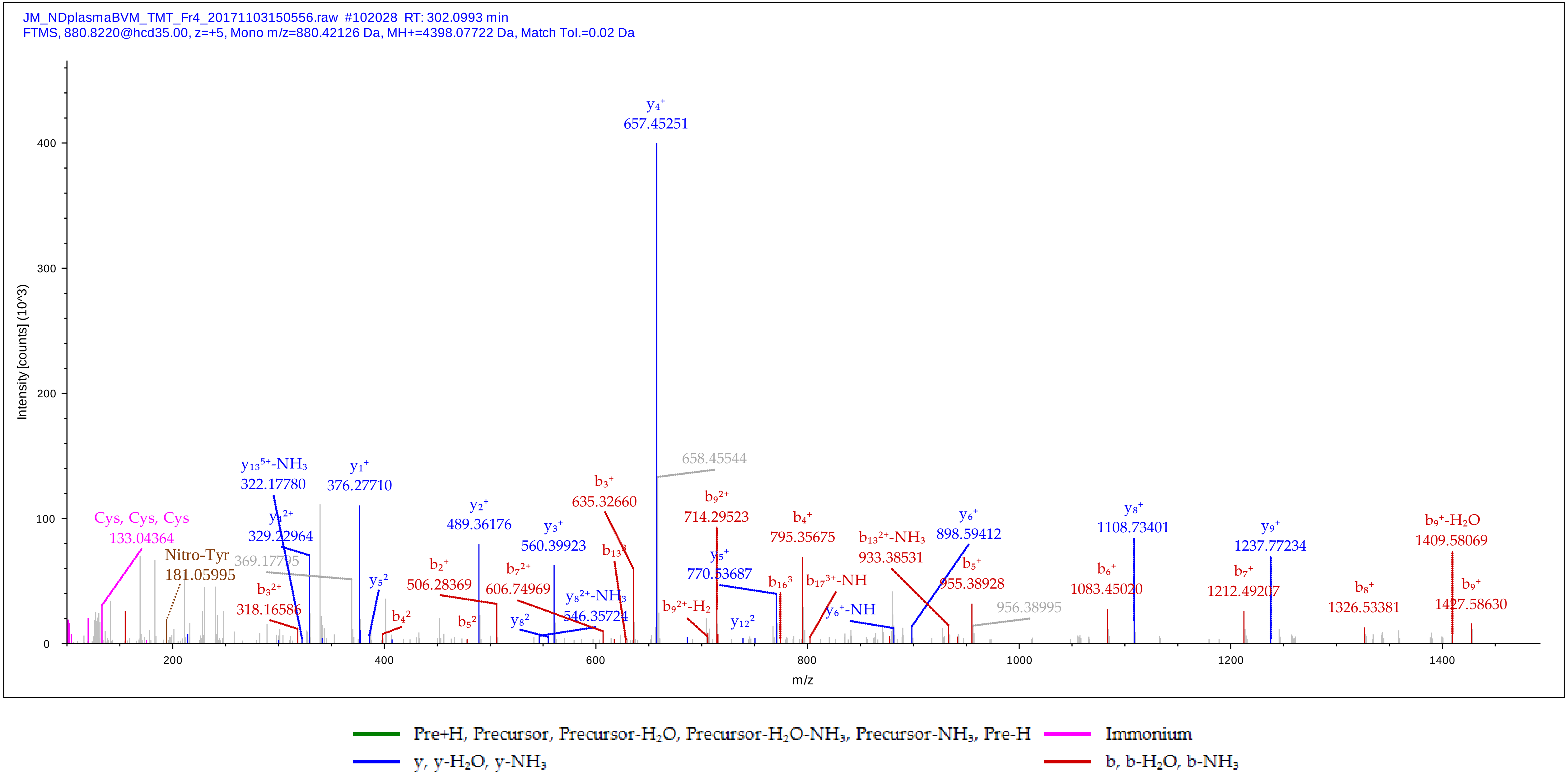

JM_NDplasmaBVM_TMT_Fr4_20171103150556.raw #102028 RT: 302.0993 min
FTMS, 880.8220@hcd35.00, z=+5, Mono m/z=880.42126 Da, MH+=4398.07722 Da, Match Tol.=0.02 Da
Intensity [counts] (10^3)
m/z
y4+ 657.45251
658.45544
y13 5+-NH3 322.17780
y1+ 376.27710
b3+ 635.32660
b9 2+ 714.29523
y2+ 489.36176
y3+ 560.39923
Cys, Cys, Cys 133.04364
y4 2+ 329.22964
Nitro-Tyr 181.05995
369.17795
b4+ 795.35675
b13 2+-NH3 933.38531
y6+ 898.59412
y8+ 1108.73401
y9+ 1237.77234
b9+-H2O 1409.58069
y5+ 770.53687
b5+ 955.38928
b6+ 1083.45020
b7+ 1212.49207
b8+ 1326.53381
b9+ 1427.58630
956.38995
b3 2+ 318.16586
b2+ 506.28369
b7 2+ 606.74969
y8 2+-NH3 546.35724
b9 2+-H2
y12 2
b16 3
b17 3+-NH
y6+-NH
Pre+H, Precursor, Precursor-H2O, Precursor-H2O-NH3, Precursor-NH3, Pre-H
Immonium
y, y-H2O, y-NH3
b, b-H2O, b-NH3

#1	Immonium	b^{+}	b^{2+}	b^{3+}	b^{4+}	Seq.	y^{+}	y^{2+}	y^{3+}	y^{4+}	#2
1	317.20224	345.19715	173.10221	115.73723	87.05475	D-TMT6plex					37
2	86.09643	458.28122	229.64425	153.43192	115.32576	I	3998.95646	1999.98187	1333.65701	1000.49457	36
3	74.06004	559.32889	280.16809	187.11448	140.58768	T	3885.87240	1943.43984	1295.96232	972.22356	35
4	74.06004	660.37657	330.69192	220.79704	165.84960	T	3784.82472	1892.91600	1262.27976	946.96164	34
5	72.08078	759.44499	380.22613	253.81985	190.61670	V	3683.77704	1842.39216	1228.59720	921.69972	33
6	87.05529	873.48791	437.24760	291.83416	219.12744	N	3584.70863	1792.85795	1195.57439	896.93261	32
7	120.08078	1020.55633	510.78180	340.85696	255.89454	F	3470.66570	1735.83649	1157.56009	868.42188	31
8	70.06513	1117.60909	559.30818	373.20788	280.15773	P	3323.59729	1662.30228	1108.53728	831.65478	30
9	70.06513	1214.66186	607.83457	405.55880	304.42092	P	3226.54452	1613.77590	1076.18636	807.39159	29
10	44.04948	1285.69897	643.35312	429.23784	322.18020	A	3129.49176	1565.24952	1043.83544	783.12840	28
11	86.09643	1398.78303	699.89515	466.93253	350.45122	L	3058.45465	1529.73096	1020.15640	765.36912	27
12	44.04948	1469.82015	735.41371	490.61157	368.21049	A	2945.37058	1473.18893	982.46171	737.09810	26
13	60.04439	1556.85217	778.92973	519.62224	389.96850	S	2874.33347	1437.67037	958.78267	719.33882	25
14	30.03383	1613.87364	807.44046	538.62940	404.22387	G	2787.30144	1394.15436	929.77200	697.58082	24
15	30.03383	1670.89510	835.95119	557.63655	418.47923	G	2730.27998	1365.64363	910.76484	683.32545	23
16	30.03383	1727.91657	864.46192	576.64371	432.73460	G	2673.25851	1337.13290	891.75769	669.07009	22
17	181.06077	1935.96497	968.48612	645.99318	484.74670	Y-Nitro	2616.23705	1308.62216	872.75053	654.81472	21
18	74.06004	2037.01265	1019.00996	679.67573	510.00862	T	2408.18864	1204.59796	803.40107	602.80262	20
19	120.04776	2184.04805	1092.52766	728.68753	546.76747	M-Oxidation	2307.14096	1154.07412	769.71851	577.54070	19
20	60.04439	2271.08008	1136.04368	757.69821	568.52548	S	2160.10557	1080.55642	720.70671	540.78185	18
21	60.04439	2358.11211	1179.55969	786.70889	590.28348	S	2073.07354	1037.04041	691.69603	519.02384	17
22	101.07094	2486.17068	1243.58898	829.39508	622.29813	Q	1986.04151	993.52439	662.68535	497.26583	16
23	86.09643	2599.25475	1300.13101	867.08977	650.56914	L	1857.98293	929.49510	619.99916	465.25119	15
24	74.06004	2700.30243	1350.65485	900.77233	675.83106	T	1744.89887	872.95307	582.30447	436.98017	14
25	86.09643	2813.38649	1407.19688	938.46701	704.10208	L	1643.85119	822.42923	548.62191	411.71825	13
26	70.06513	2910.43925	1455.72327	970.81794	728.36527	P	1530.76712	765.88720	510.92723	383.44724	12
27	44.04948	2981.47637	1491.24182	994.49697	746.12455	A	1433.71436	717.36082	478.57630	359.18405	11
28	72.08078	3080.54478	1540.77603	1027.51978	770.89165	V	1362.67725	681.84226	454.89727	341.42477	10
29	102.05496	3209.58738	1605.29733	1070.53398	803.15230	E	1263.60883	632.30806	421.87446	316.65767	9
30	133.04301	3369.61802	1685.31265	1123.87753	843.15996	C-Carbamidomethyl	1134.56624	567.78676	378.86026	284.39702	8
31	70.06513	3466.67079	1733.83903	1156.22845	867.42315	P	974.53559	487.77143	325.51672	244.38936	7
32	102.05496	3595.71338	1798.36033	1199.24264	899.68380	E	877.48283	439.24505	293.16579	220.12616	6
33	30.03383	3652.73484	1826.87106	1218.24980	913.93917	G	748.44024	374.72376	250.15160	187.86552	5
34	102.05496	3781.77744	1891.39236	1261.26400	946.19982	E	691.41877	346.21302	231.14444	173.61015	4
35	60.04439	3868.80947	1934.90837	1290.27467	967.95782	S	562.37618	281.69173	188.13024	141.34950	3
36	72.08078	3967.87788	1984.44258	1323.29748	992.72493	V	475.34415	238.17571	159.11957	119.59150	2
37	330.27026					K-TMT6plex	376.27574	188.64151	126.09676	94.82439	1

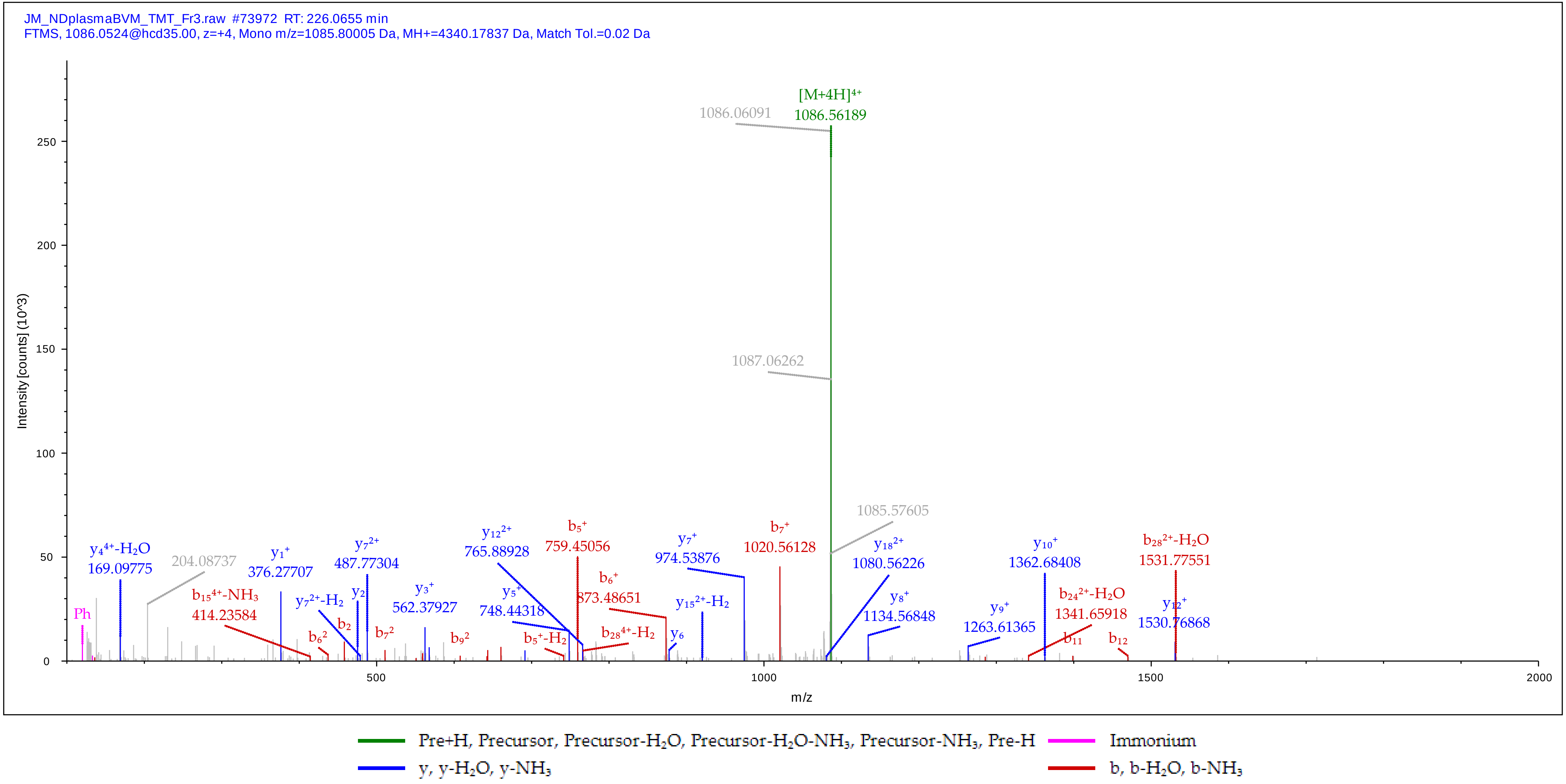
JM_NDplasmaBVM_TMT_Fr3.raw #73972 RT: 226.0655 min
FTMS, 1086.0524@hcd35.00, z=+4, Mono m/z=1085.80005 Da, MH+=4340.17837 Da, Match Tol.=0.02 Da
Intensity [counts] (10^3)
m/z
[M+4H]4+
1086.56189
1086.06091
1087.06262
1085.57605
y4 4+-H2O
169.09775
204.08737
y1+
376.27707
y7 2+
487.77304
y12 2+
765.88928
b5+
759.45056
y7+
974.53876
b7+
1020.56128
y18 2+
1080.56226
y10+
1362.68408
b28 2+-H2O
1531.77551
Ph
b15 4+-NH3
414.23584
y7 2+-H2
y3+
562.37927
y5+
748.44318
b6+
873.48651
y15 2+-H2
y8+
1134.56848
y9+
1263.61365
b24 2+-H2O
1341.65918
y12+
1530.76868
b6 2
b2
b7 2
b9 2
b5+-H2
b28 4+-H2
y6
b11
b12
Pre+H, Precursor, Precursor-H2O, Precursor-H2O-NH3, Precursor-NH3, Pre-H
Immonium
y, y-H2O, y-NH3
b, b-H2O, b-NH3

#1	Immonium	b+	b2+	b3+	b4+	Seq.	y+	y2+	y3+	y4+	#2
1	303.22297	331.21789	166.11258	111.07748	83.55993	T-TMT6plex					29
2	44.04948	402.25500	201.63114	134.75652	101.31921	A	3186.48956	1593.74842	1062.83471	797.37785	28
3	60.04439	489.28703	245.14715	163.76719	123.07721	S	3115.45245	1558.22986	1039.15567	779.61857	27
4	70.06513	586.33979	293.67353	196.11812	147.34041	P	3028.42042	1514.71385	1010.14499	757.86056	26
5	88.03930	701.36674	351.18701	234.46043	176.09714	D	2931.36766	1466.18747	977.79407	733.59737	25
6	101.07094	829.42531	415.21629	277.14662	208.11179	Q	2816.34071	1408.67400	939.45176	704.84064	24
7	74.06004	930.47299	465.74013	310.82918	233.37371	T	2688.28214	1344.64471	896.76556	672.82599	23
8	102.05496	1059.51558	530.26143	353.84338	265.63435	E	2587.23446	1294.12087	863.08300	647.56407	22
9	120.04776	1206.55098	603.77913	402.85518	302.39320	M-Oxidation	2458.19186	1229.59957	820.06881	615.30342	21
10	74.06004	1307.59866	654.30297	436.53774	327.65512	T	2311.15647	1156.08187	771.05701	578.54457	20
11	86.09643	1420.68273	710.84500	474.23243	355.92614	I	2210.10879	1105.55803	737.37445	553.28265	19
12	102.05496	1549.72532	775.36630	517.24662	388.18679	E	2097.02472	1049.01600	699.67976	525.01164	18
13	30.03383	1606.74678	803.87703	536.25378	402.44215	G	1967.98213	984.49470	656.66556	492.75099	17
14	86.09643	1719.83085	860.41906	573.94847	430.71317	L	1910.96067	955.98397	637.65841	478.49562	16
15	101.07094	1847.88942	924.44835	616.63466	462.72781	Q	1797.87660	899.44194	599.96372	450.22461	15
16	70.06513	1944.94219	972.97473	648.98558	486.99100	P	1669.81802	835.41265	557.27753	418.20996	14
17	74.06004	2045.98987	1023.49857	682.66814	512.25292	T	1572.76526	786.88627	524.92660	393.94677	13
18	72.08078	2145.05828	1073.03278	715.69094	537.02003	V	1471.71758	736.36243	491.24405	368.68485	12
19	102.05496	2274.10087	1137.55408	758.70514	569.28068	E	1372.64917	686.82822	458.22124	343.91775	11
20	181.06077	2482.14928	1241.57828	828.05461	621.29278	Y-Nitro	1243.60658	622.30693	415.20704	311.65710	10
21	72.08078	2581.21769	1291.11249	861.07742	646.05988	V	1035.55817	518.28272	345.85757	259.64500	9
22	72.08078	2680.28611	1340.64669	894.10022	670.82698	V	936.48976	468.74852	312.83477	234.87790	8
23	60.04439	2767.31814	1384.16271	923.11090	692.58499	S	837.42134	419.21431	279.81196	210.11079	7
24	72.08078	2866.38655	1433.69691	956.13370	717.35210	V	750.38931	375.69829	250.80129	188.35279	6
25	136.07569	3029.44988	1515.22858	1010.48814	758.11793	Y	651.32090	326.16409	217.77848	163.58568	5
26	44.04948	3100.48699	1550.74713	1034.16718	775.87721	A	488.25757	244.63242	163.42404	122.81985	4
27	101.07094	3228.54557	1614.77642	1076.85337	807.89185	Q	417.22046	209.11387	139.74500	105.06057	3
28	87.05529	3342.58850	1671.79789	1114.86768	836.40258	N	289.16188	145.08458	97.05881	73.04593	2
29	129.11347					R	175.11895	88.06311	59.04450	44.53520	1

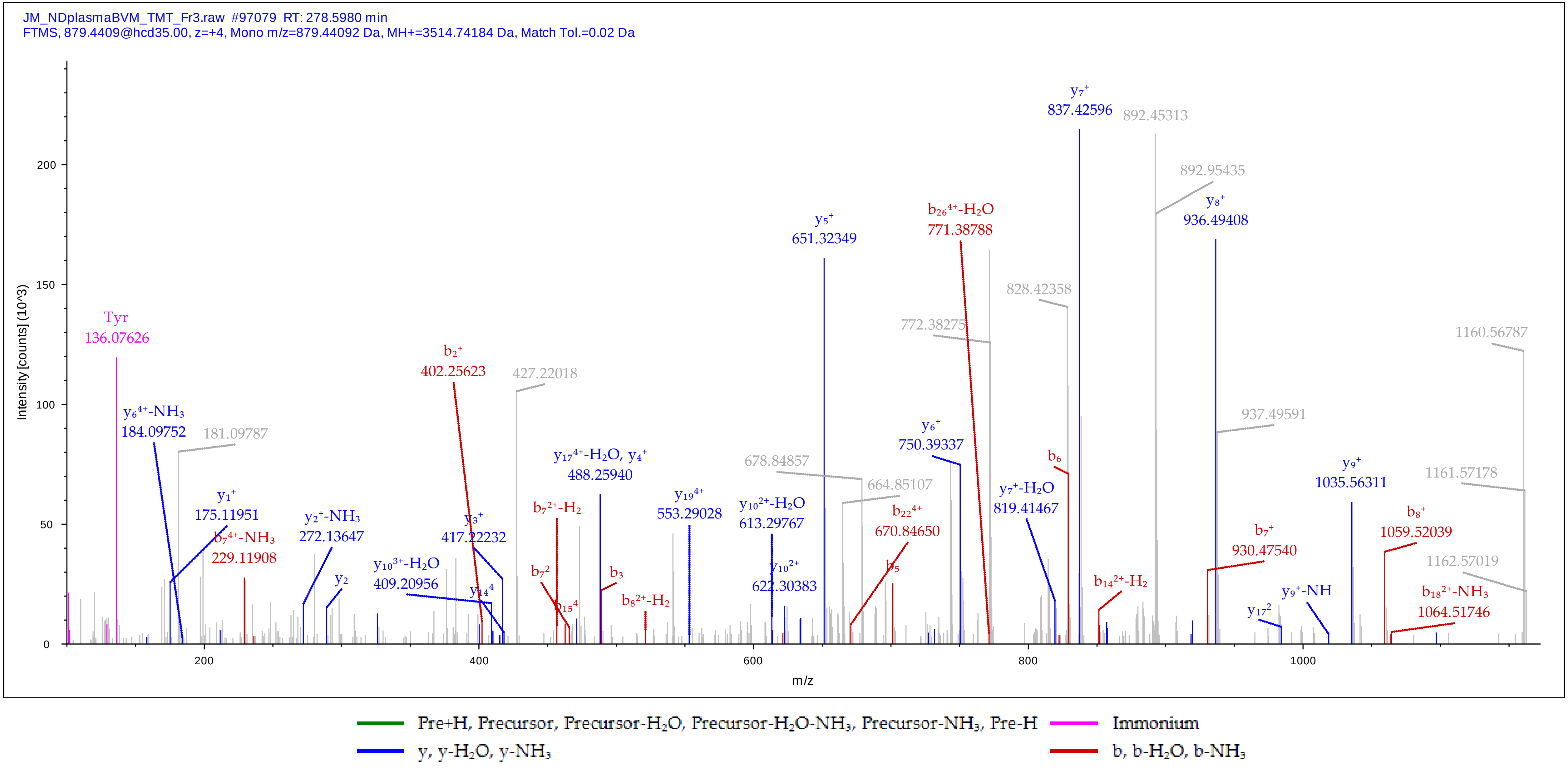

JM_NDplasmaBVM_TMT_Fr3.raw #97079 RT: 278.5980 min
FTMS, 879.4409@hcd35.00, z=+4, Mono m/z=879.44092 Da, MH+=3514.74184 Da, Match Tol.=0.02 Da
Intensity [counts] (10^3)
m/z
Tyr 136.07626
y_6^{4+}-NH_3 184.09752
181.09787
y_1^+ 175.11951
b_7^{4+}-NH_3 229.11908
y_2^+-NH_3 272.13647
y_2
b_2^+ 402.25623
427.22018
y_{10}^{3+}-H_2O 409.20956
y_3^+ 417.22232
y_{14}^4
y_{17}^{4+}-H_2O, y_4^+ 488.25940
b_7^{2+}-H_2
b_7^2
b_{15}^4
b_3
b_8^{2+}-H_2
y_{19}^{4+} 553.29028
y_{10}^{2+}-H_2O 613.29767
y_{10}^{2+} 622.30383
678.84857
664.85107
y_5^+ 651.32349
b_{22}^{4+} 670.84650
b_5
b_{26}^{4+}-H_2O 771.38788
772.38275
y_6^+ 750.39337
828.42358
b_6
y_7^+-H_2O 819.41467
y_7^+ 837.42596
892.45313
892.95435
b_{14}^{2+}-H_2
y_8^+ 936.49408
937.49591
b_7^+ 930.47540
y_{17}^2
y_9^+-NH
y_9^+ 1035.56311
b_8^+ 1059.52039
b_{18}^{2+}-NH_3 1064.51746
1160.56787
1161.57178
1162.57019
Pre+H, Precursor, Precursor-H_2O, Precursor-H_2O-NH_3, Precursor-NH_3, Pre-H
Immonium
y, y-H_2O, y-NH_3
b, b-H_2O, b-NH_3

#1	Immonium	b⁺	b²⁺	b³⁺	Seq.	y⁺	y²⁺	y³⁺	#2
1	315.25936	343.25427	172.13077	115.08961	I-TMT6plex				18
2	60.04439	430.28630	215.64679	144.10028	S	2371.04513	1186.02620	791.01990	17
3	120.08078	577.35471	289.18100	193.12309	F	2284.01310	1142.51019	762.00922	16
4	88.03930	692.38166	346.69447	231.46540	D	2136.94469	1068.97598	712.98641	15
5	70.06513	789.43442	395.22085	263.81632	P	2021.91775	1011.46251	674.64410	14
6	101.07094	917.49300	459.25014	306.50252	Q	1924.86498	962.93613	642.29318	13
7	88.03930	1032.51994	516.76361	344.84483	D	1796.80641	898.90684	599.60699	12
8	74.06004	1133.56762	567.28745	378.52739	T	1681.77946	841.39337	561.26467	11
9	120.08078	1280.63603	640.82166	427.55020	F	1580.73178	790.86953	527.58211	10
10	102.05496	1409.67863	705.34295	470.56439	E	1433.66337	717.33532	478.55931	9
11	60.04439	1496.71066	748.85897	499.57507	S	1304.62078	652.81403	435.54511	8
12	102.05496	1625.75325	813.38026	542.58927	E	1217.58875	609.29801	406.53443	7
13	120.08078	1772.82166	886.91447	591.61207	F	1088.54616	544.77672	363.52024	6
14	181.06077	1980.87007	990.93867	660.96154	Y-Nitro	941.47774	471.24251	314.49743	5
15	86.09643	2093.95413	1047.48070	698.65623	L	733.42934	367.21831	245.14796	4
16	88.03930	2208.98108	1104.99418	736.99854	D	620.34527	310.67627	207.45328	3
17	102.05496	2338.02367	1169.51547	780.01274	E	505.31833	253.16280	169.11096	2
18	330.27026				K-TMT6plex	376.27574	188.64151	126.09676	1

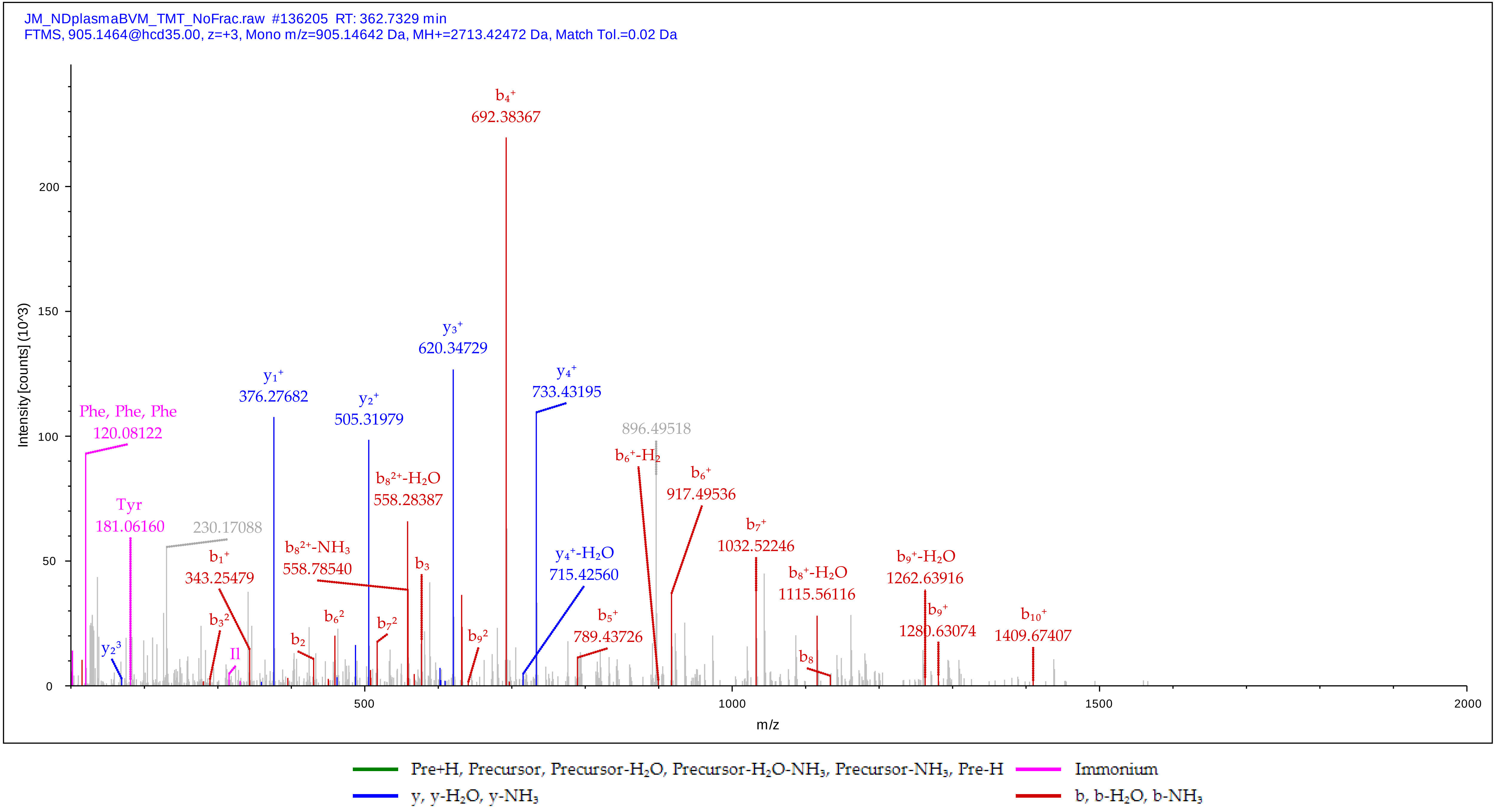
JM_NDplasmaBVM_TMT_NoFrac.raw #136205 RT: 362.7329 min
FTMS, 905.1464@hcd35.00, z=+3, Mono m/z=905.14642 Da, MH+=2713.42472 Da, Match Tol.=0.02 Da
Intensity[counts] (10^3)
m/z
b4+ 692.38367
y3+ 620.34729
y4+ 733.43195
y1+ 376.27682
y2+ 505.31979
Phe, Phe, Phe 120.08122
896.49518
b6+-H2
b6+ 917.49536
b8 2+-H2O 558.28387
Tyr 181.06160
230.17088
b1+ 343.25479
b8 2+-NH3 558.78540
b3
y4+-H2O 715.42560
b7+ 1032.52246
b9+-H2O 1262.63916
b8+-H2O 1115.56116
b3 2
b6 2
b7 2
b9 2
b5+ 789.43726
b9+ 1280.63074
b10+ 1409.67407
b2
b8
y2 3
Il
Pre+H, Precursor, Precursor-H2O, Precursor-H2O-NH3, Precursor-NH3, Pre-H
Immonium
y, y-H2O, y-NH3
b, b-H2O, b-NH3

#1	Immonium	b+	b2+	b3+	Seq.	y+	y2+	y3+	#2
1	273.21241	301.20732	151.10730	101.07396	A-TMT6plex				19
2	86.09643	414.29139	207.64933	138.76865	L	2362.14004	1181.57366	788.05153	18
3	181.06077	622.33979	311.67353	208.11812	Y-Nitro	2249.05597	1125.03162	750.35684	17
4	101.07094	750.39837	375.70282	250.80431	Q	2041.00757	1021.00742	681.00737	16
5	74.06004	851.44605	426.22666	284.48687	T	1912.94899	956.97813	638.32118	15
6	102.05496	980.48864	490.74796	327.50106	E	1811.90131	906.45429	604.63862	14
7	44.04948	1051.52576	526.26652	351.18010	A	1682.85872	841.93300	561.62442	13
8	120.08078	1198.59417	599.80072	400.20291	F	1611.82160	806.41444	537.94539	12
9	74.06004	1299.64185	650.32456	433.88547	T	1464.75319	732.88023	488.92258	11
10	44.04948	1370.67896	685.84312	457.56450	A	1363.70551	682.35639	455.24002	10
11	88.03930	1485.70590	743.35659	495.90682	D	1292.66840	646.83784	431.56098	9
12	120.08078	1632.77432	816.89080	544.92962	F	1177.64145	589.32437	393.21867	8
13	101.07094	1760.83290	880.92009	587.61582	Q	1030.57304	515.79016	344.19586	7
14	101.07094	1888.89147	944.94937	630.30201	Q	902.51446	451.76087	301.50967	6
15	70.06513	1985.94424	993.47576	662.65293	P	774.45589	387.73158	258.82348	5
16	74.06004	2086.99192	1043.99960	696.33549	T	677.40312	339.20520	226.47256	4
17	102.05496	2216.03451	1108.52089	739.34969	E	576.35544	288.68136	192.79000	3
18	44.04948	2287.07162	1144.03945	763.02873	A	447.31285	224.16006	149.77580	2
19	330.27026				K-TMT6plex	376.27574	188.64151	126.09676	1

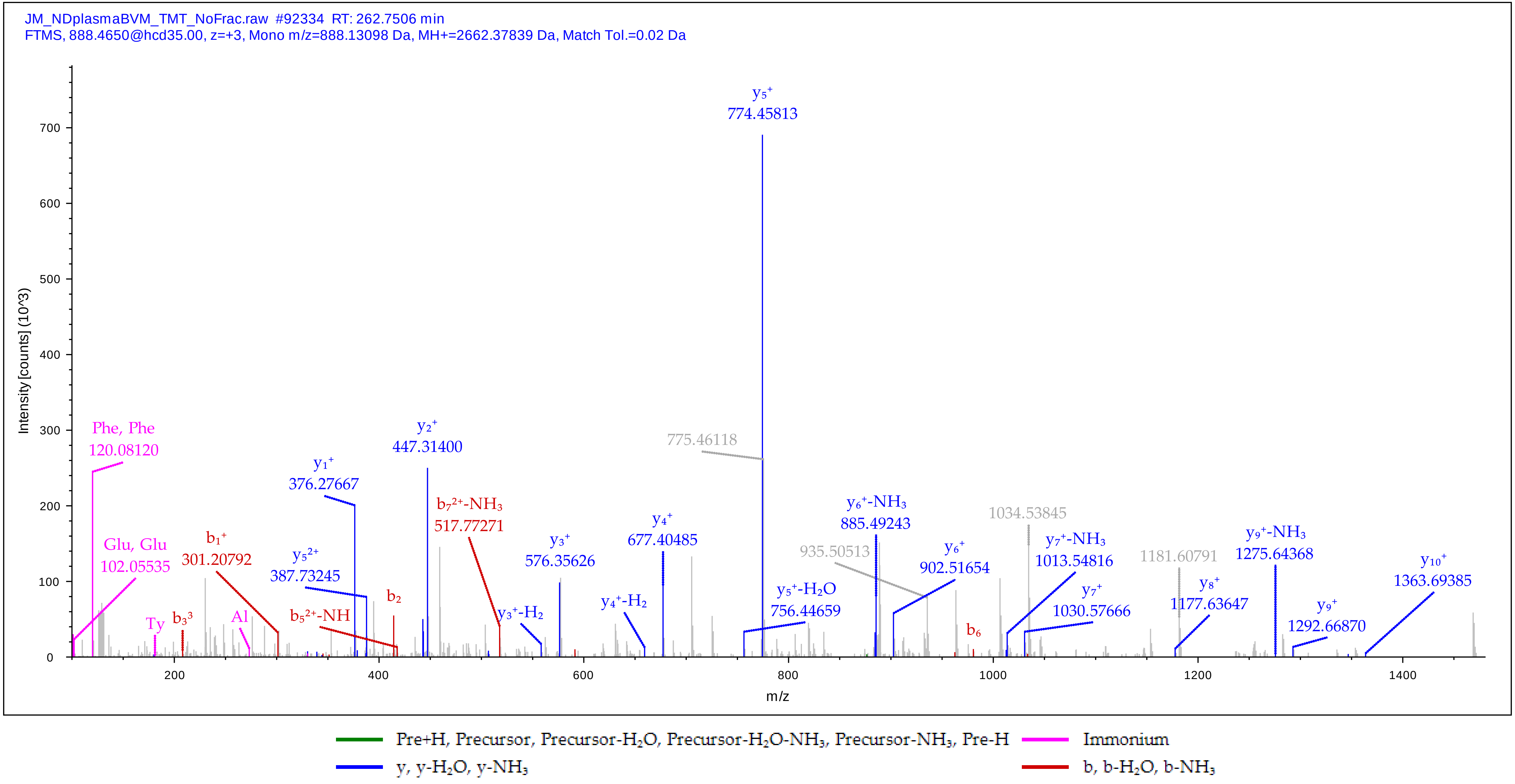

JM_NDplasmaBVM_TMT_NoFrac.raw #92334 RT: 262.7506 min
FTMS, 888.4650@hcd35.00, z=+3, Mono m/z=888.13098 Da, MH+=2662.37839 Da, Match Tol.=0.02 Da
Intensity [counts] (10^3)
m/z
y5+ 774.45813
775.46118
Phe, Phe 120.08120
y2+ 447.31400
y1+ 376.27667
b72+-NH3 517.77271
y6+-NH3 885.49243
1034.53845
Glu, Glu 102.05535
b1+ 301.20792
y52+ 387.73245
y3+ 576.35626
y4+ 677.40485
935.50513
y6+ 902.51654
y7+-NH3 1013.54816
1181.60791
y9+-NH3 1275.64368
y10+ 1363.69385
b2
b52+-NH
b33
Ty
Al
y3+-H2
y4+-H2
y5+-H2O 756.44659
b6
y7+ 1030.57666
y8+ 1177.63647
y9+ 1292.66870
Pre+H, Precursor, Precursor-H2O, Precursor-H2O-NH3, Precursor-NH3, Pre-H
Immonium
y, y-H2O, y-NH3
b, b-H2O, b-NH3

#1	Immonium	b⁺	b²⁺	b³⁺	Seq.	y⁺	y²⁺	y³⁺	#2
1	317.20224	345.19715	173.10221	115.73723	D-TMT6plex				34
2	30.03383	402.21862	201.61295	134.74439	G	3518.62026	1759.81377	1173.54494	33
3	120.08078	549.28703	275.14715	183.76719	F	3461.59880	1731.30304	1154.53778	32
4	72.08078	648.35544	324.68136	216.79000	V	3314.53038	1657.76883	1105.51498	31
5	101.07094	776.41402	388.71065	259.47619	Q	3215.46197	1608.23462	1072.49217	30
6	30.03383	833.43548	417.22138	278.48335	G	3087.40339	1544.20533	1029.80598	29
7	102.05496	962.47808	481.74268	321.49754	E	3030.38193	1515.69460	1010.79883	28
8	30.03383	1019.49954	510.25341	340.50470	G	2901.33933	1451.17331	967.78463	27
9	74.06004	1120.54722	560.77725	374.18726	T	2844.31787	1422.66257	948.77747	26
10	120.04776	1267.58262	634.29495	423.19906	M-Oxidation	2743.27019	1372.13873	915.09491	25
11	72.08078	1366.65103	683.82915	456.22186	V	2596.23479	1298.62103	866.08312	24
12	70.06513	1463.70380	732.35554	488.57278	P	2497.16638	1249.08683	833.06031	23
13	72.08078	1562.77221	781.88974	521.59559	V	2400.11361	1200.56045	800.70939	22
14	30.03383	1619.79367	810.40048	540.60274	G	2301.04520	1151.02624	767.68658	21
15	101.07094	1747.85225	874.42976	583.28893	Q	2244.02374	1122.51551	748.67943	20
16	60.04439	1834.88428	917.94578	612.29961	S	2115.96516	1058.48622	705.99324	19
17	72.08078	1933.95269	967.47999	645.32242	V	2028.93313	1014.97020	676.98256	18
18	72.08078	2033.02111	1017.01419	678.34522	V	1929.86472	965.43600	643.95976	17
19	181.06077	2241.06951	1121.03840	747.69469	Y-Nitro	1830.79630	915.90179	610.93695	16
20	44.04948	2312.10663	1156.55695	771.37373	A	1622.74790	811.87759	541.58748	15
21	133.04301	2472.13728	1236.57228	824.71728	C-Carbamidomethyl	1551.71078	776.35903	517.90845	14
22	88.03930	2587.16422	1294.08575	863.05959	D	1391.68013	696.34371	464.56490	13
23	102.05496	2716.20681	1358.60704	906.07379	E	1276.65319	638.83023	426.22258	12
24	30.03383	2773.22828	1387.11778	925.08094	G	1147.61060	574.30894	383.20838	11
25	136.07569	2936.29160	1468.64944	979.43539	Y	1090.58914	545.79821	364.20123	10
26	60.04439	3023.32363	1512.16545	1008.44606	S	927.52581	464.26654	309.84679	9
27	86.09643	3136.40770	1568.70749	1046.14075	L	840.49378	420.75053	280.83611	8
28	86.09643	3249.49176	1625.24952	1083.83544	I	727.40971	364.20850	243.14142	7
29	30.03383	3306.51322	1653.76025	1102.84259	G	614.32565	307.66646	205.44673	6
30	88.03930	3421.54017	1711.27372	1141.18491	D	557.30419	279.15573	186.43958	5
31	70.06513	3518.59293	1759.80010	1173.53583	P	442.27724	221.64226	148.09727	4
32	72.08078	3617.66134	1809.33431	1206.55863	V	345.22448	173.11588	115.74634	3
33	44.04948	3688.69846	1844.85287	1230.23767	A	246.15607	123.58167	82.72354	2
34	129.11347				R	175.11895	88.06311	59.04450	1

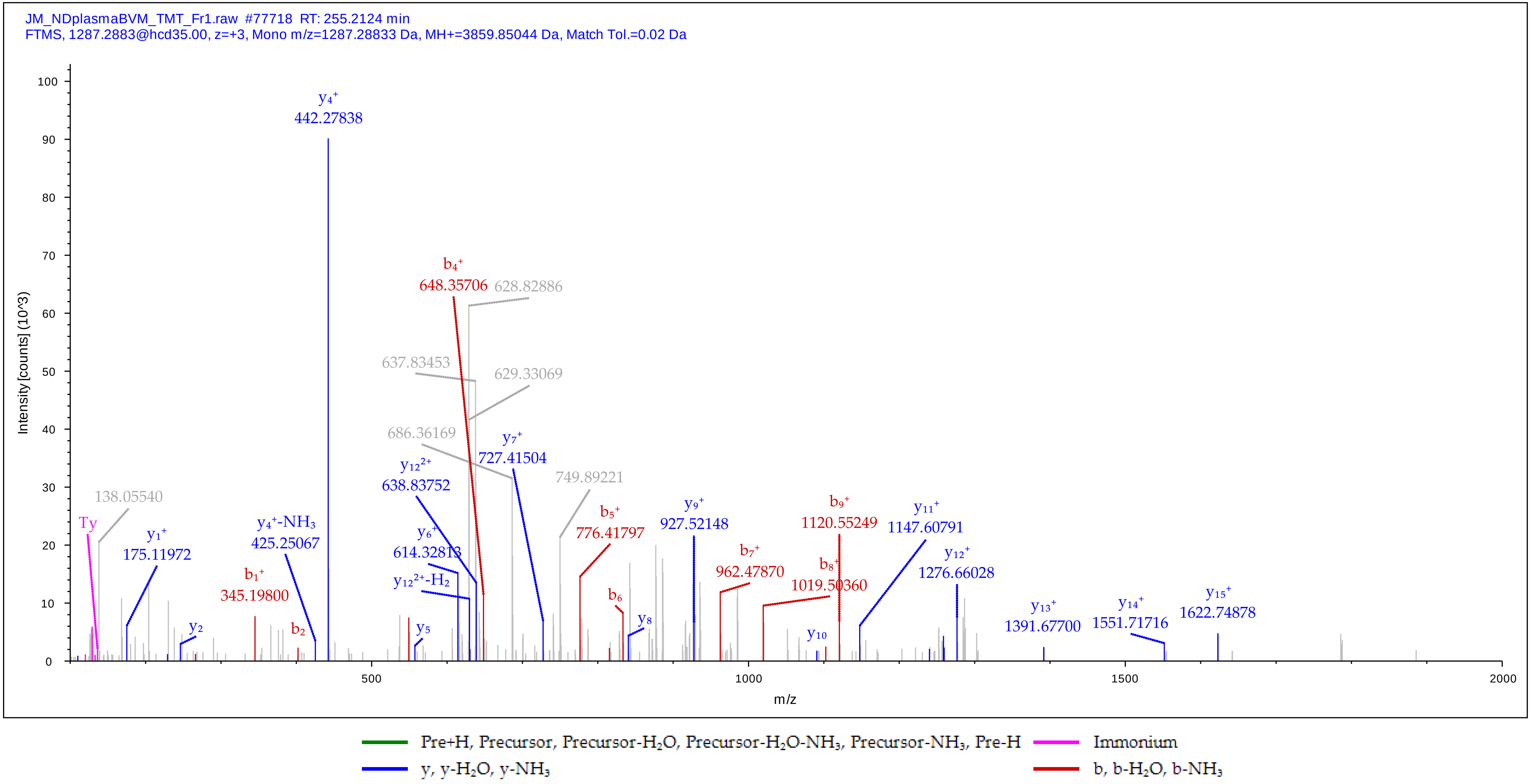
JM_NDplasmaBVM_TMT_Fr1.raw #77718 RT: 255.2124 min
FTMS, 1287.2883@hcd35.00, z=+3, Mono m/z=1287.28833 Da, MH+=3859.85044 Da, Match Tol.=0.02 Da
Intensity [counts] (10^3)
m/z
y4+ 442.27838
b4+ 648.35706
628.82886
637.83453
629.33069
686.36169
y7+ 727.41504
y12 2+ 638.83752
749.89221
138.05540
Ty
y1+ 175.11972
y4+-NH3 425.25067
y6+ 614.32813
y12 2+-H2
b1+ 345.19800
y2
b2
y5
b5+ 776.41797
b6
y8
y9+ 927.52148
b7+ 962.47870
b8+ 1019.50360
b9+ 1120.55249
y10
y11+ 1147.60791
y12+ 1276.66028
y13+ 1391.67700
y14+ 1551.71716
y15+ 1622.74878
Pre+H, Precursor, Precursor-H2O, Precursor-H2O-NH3, Precursor-NH3, Pre-H
Immonium
y, y-H2O, y-NH3
b, b-H2O, b-NH3

#1	Immonium	b^{+}	b^{2+}	b^{3+}	b^{4+}	b^{5+}	Seq.	y^{+}	y^{2+}	y^{3+}	y^{4+}	y^{5+}	#2
1	362.20594	390.20086	195.60407	130.73847	98.30567	78.84599	C-TMT6plex-Carbamidomethyl						37
2	30.03383	447.22232	224.11480	149.74562	112.56104	90.25029	G	4679.29036	2340.14882	1560.43497	1170.57805	936.66389	36
3	70.06513	544.27508	272.64118	182.09655	136.82423	109.66084	P	4622.26890	2311.63809	1541.42782	1156.32268	925.25960	35
4	70.06513	641.32785	321.16756	214.44747	161.08742	129.07139	P	4525.21613	2263.11170	1509.07690	1132.05949	905.84905	34
5	70.06513	738.38061	369.69394	246.79839	185.35061	148.48194	P	4428.16337	2214.58532	1476.72597	1107.79630	886.43850	33
6	70.06513	835.43338	418.22033	279.14931	209.61380	167.89250	P	4331.11060	2166.05894	1444.37505	1083.53311	867.02794	32
7	86.09643	948.51744	474.76236	316.84400	237.88482	190.50931	I	4234.05784	2117.53256	1412.02413	1059.26992	847.61739	31
8	88.03930	1063.54438	532.27583	355.18631	266.64155	213.51470	D	4120.97378	2060.99053	1374.32944	1030.99890	825.00058	30
9	87.05529	1177.58731	589.29729	393.20062	295.15229	236.32328	N	4005.94683	2003.47706	1335.98713	1002.24217	801.99519	29
10	30.03383	1234.60877	617.80803	412.20778	309.40765	247.72758	G	3891.90391	1946.45559	1297.97282	973.73143	779.18660	28
11	88.03930	1349.63572	675.32150	450.55009	338.16439	270.73296	D	3834.88244	1917.94486	1278.96567	959.47607	767.78231	27
12	86.09643	1462.71978	731.86353	488.24478	366.43540	293.34978	I	3719.85550	1860.43139	1240.62335	930.71933	744.77692	26
13	74.06004	1563.76746	782.38737	521.92734	391.69732	313.55931	T	3606.77144	1803.88936	1202.92866	902.44832	722.16011	25
14	60.04439	1650.79949	825.90338	550.93801	413.45533	330.96572	S	3505.72376	1753.36552	1169.24610	877.18640	701.95057	24
15	86.09643	1763.88355	882.44541	588.63270	441.72635	353.58253	L	3418.69173	1709.84950	1140.23543	855.42839	684.54417	23
16	60.04439	1850.91558	925.96143	617.64338	463.48435	370.98894	S	3305.60767	1653.30747	1102.54074	827.15737	661.92735	22
17	86.09643	1963.99964	982.50346	655.33807	491.75537	393.60575	L	3218.57564	1609.79146	1073.53006	805.39937	644.52095	21
18	70.06513	2061.05241	1031.02984	687.68899	516.01856	413.01630	P	3105.49157	1553.24942	1035.83538	777.12835	621.90414	20
19	102.05496	2190.09500	1095.55114	730.70318	548.27921	438.82482	E	3008.43881	1504.72304	1003.48445	752.86516	602.49358	19
20	136.07569	2353.15833	1177.08280	785.05763	589.04504	471.43749	Y	2879.39622	1440.20175	960.47026	720.60451	576.68506	18
21	102.05496	2482.20092	1241.60410	828.07183	621.30569	497.24601	E	2716.33289	1358.67008	906.11581	679.83868	544.07240	17
22	70.06513	2579.25369	1290.13048	860.42275	645.56888	516.65656	P	2587.29030	1294.14879	863.10162	647.57803	518.26388	16
23	120.08078	2726.32210	1363.66469	909.44555	682.33598	546.07024	F	2490.23753	1245.62240	830.75069	623.31484	498.85333	15
24	60.04439	2813.35413	1407.18070	938.45623	704.09399	563.47665	S	2343.16912	1172.08820	781.72789	586.54774	469.43964	14
25	60.04439	2900.38616	1450.69672	967.46690	725.85200	580.88305	S	2256.13709	1128.57218	752.71721	564.78973	452.03324	13
26	72.08078	2999.45457	1500.23092	1000.48971	750.61910	600.69674	V	2169.10506	1085.05617	723.70654	543.03172	434.62683	12
27	88.03930	3114.48151	1557.74439	1038.83202	779.37584	623.70212	D	2070.03665	1035.52196	690.68373	518.26462	414.81315	11
28	181.06077	3322.52992	1661.76860	1108.18149	831.38794	665.31181	Y-Nitro	1955.00970	978.00849	652.34142	489.50788	391.80776	10
29	101.07094	3450.58850	1725.79789	1150.86768	863.40258	690.92352	Q	1746.96130	873.98429	582.99195	437.49578	350.19808	9
30	133.04301	3610.61915	1805.81321	1204.21123	903.41024	722.92965	C-Carbamidomethyl	1618.90272	809.95500	540.30576	405.48114	324.58637	8
31	101.07094	3738.67772	1869.84250	1246.89743	935.42489	748.54137	Q	1458.87207	729.93967	486.96221	365.47348	292.58024	7
32	330.27026	4095.93562	2048.47145	1365.98339	1024.73936	819.99294	K-TMT6plex	1330.81349	665.91039	444.27602	333.45883	266.96852	6
33	181.06077	4303.98402	2152.49565	1435.33286	1076.75146	861.60263	Y-Nitro	973.55560	487.28144	325.19005	244.14436	195.51694	5
34	136.07569	4467.04735	2234.02731	1489.68730	1117.51730	894.21529	Y	765.50719	383.25723	255.84058	192.13226	153.90726	4
35	86.09643	4580.13142	2290.56935	1527.38199	1145.78831	916.83210	L	602.44386	301.72557	201.48614	151.36642	121.29459	3
36	86.09643	4693.21548	2347.11138	1565.07668	1174.05933	939.44892	L	489.35980	245.18354	163.79145	123.09541	98.67778	2
37	330.27026						K-TMT6plex	376.27574	188.64151	126.09676	94.82439	76.06097	1

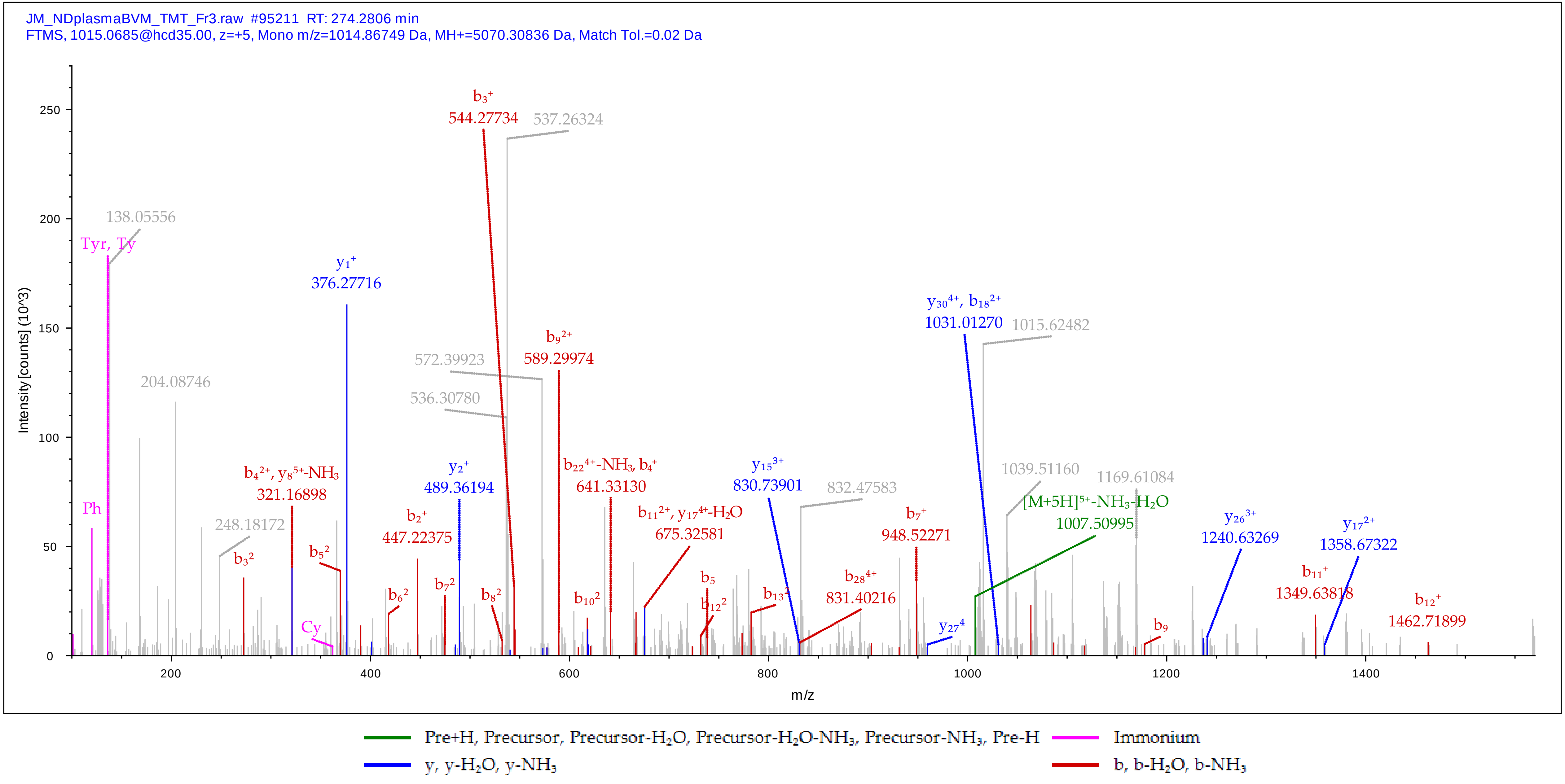

JM_NDplasmaBVM_TMT_Fr3.raw #95211 RT: 274.2806 min
FTMS, 1015.0685@hcd35.00, z=+5, Mono m/z=1014.86749 Da, MH+=5070.30836 Da, Match Tol.=0.02 Da
Intensity [counts] (10^3)
m/z
b3+ 544.27734
537.26324
138.05556
Tyr, Ty
y1+ 376.27716
204.08746
572.39923
536.30780
b9 2+ 589.29974
y30 4+, b18 2+ 1031.01270
1015.62482
b4 2+, y8 5+-NH3 321.16898
y2+ 489.36194
b22 4+-NH3, b4+ 641.33130
y15 3+ 830.73901
832.47583
1039.51160
1169.61084
[M+5H]5+-NH3-H2O 1007.50995
b2+ 447.22375
b11 2+, y17 4+-H2O 675.32581
b7+ 948.52271
y26 3+ 1240.63269
y17 2+ 1358.67322
Ph
248.18172
b3 2
b5 2
b6 2
b7 2
b8 2
b10 2
b5
b12 2
b13 2
b28 4+ 831.40216
b11+ 1349.63818
b12+ 1462.71899
Cy
y27 4
b9
Pre+H, Precursor, Precursor-H2O, Precursor-H2O-NH3, Precursor-NH3, Pre-H
Immonium
y, y-H2O, y-NH3
b, b-H2O, b-NH3

#1	Immonium	b⁺	b^{2+}	b^{3+}	b^{4+}	Seq.	y⁺	y^{2+}	y^{3+}	y^{4+}	#2
1	273.21241	301.20732	151.10730	101.07396	76.05729	A-TMT6plex					23
2	86.09643	414.29139	207.64933	138.76865	104.32830	I	2993.54428	1497.27578	998.51961	749.14153	22
3	101.07094	542.34996	271.67862	181.45484	136.34295	Q	2880.46021	1440.73375	960.82492	720.87051	21
4	72.08078	641.41838	321.21283	214.47764	161.11005	V	2752.40164	1376.70446	918.13873	688.85587	20
5	136.07569	804.48171	402.74449	268.83209	201.87588	Y	2653.33322	1327.17025	885.11593	664.08876	19
6	181.06077	1012.53011	506.76869	338.18156	253.88799	Y-Nitro	2490.26989	1245.63859	830.76148	623.32293	18
7	87.05529	1126.57304	563.79016	376.19586	282.39872	N	2282.22149	1141.61438	761.41201	571.31083	17
8	70.06513	1223.62580	612.31654	408.54679	306.66191	P	2168.17856	1084.59292	723.39770	542.80010	16
9	88.03930	1338.65275	669.83001	446.88910	335.41864	D	2071.12580	1036.06654	691.04678	518.53691	15
10	101.07094	1466.71132	733.85930	489.57529	367.43329	Q	1956.09885	978.55306	652.70447	489.78017	14
11	70.06513	1563.76409	782.38568	521.92621	391.69648	P	1828.04028	914.52378	610.01828	457.76553	13
12	70.06513	1660.81685	830.91206	554.27714	415.95967	P	1730.98751	865.99739	577.66736	433.50234	12
13	330.27026	2018.07475	1009.54101	673.36310	505.27414	K-TMT6plex	1633.93475	817.47101	545.31643	409.23914	11
14	70.06513	2115.12751	1058.06739	705.71402	529.53734	P	1276.67685	638.84206	426.23047	319.92467	10
15	30.03383	2172.14897	1086.57813	724.72118	543.79270	G	1179.62409	590.31568	393.87955	295.66148	9
16	104.05285	2303.18946	1152.09837	768.40134	576.55282	M	1122.60263	561.80495	374.87239	281.40611	8
17	86.09643	2416.27352	1208.64040	806.09603	604.82384	I	991.56214	496.28471	331.19223	248.64599	7
18	88.03930	2531.30047	1266.15387	844.43834	633.58057	D	878.47808	439.74268	293.49754	220.37498	6
19	60.04439	2618.33249	1309.66989	873.44902	655.33858	S	763.45113	382.22921	255.15523	191.61824	5
20	44.04948	2689.36961	1345.18844	897.12805	673.09786	A	676.41911	338.71319	226.14455	169.86023	4
21	74.06004	2790.41729	1395.71228	930.81061	698.35978	T	605.38199	303.19463	202.46552	152.10096	3
22	101.07094	2918.47586	1459.74157	973.49681	730.37442	Q	504.33431	252.67080	168.78296	126.83904	2
23	330.27026					K-TMT6plex	376.27574	188.64151	126.09676	94.82439	1

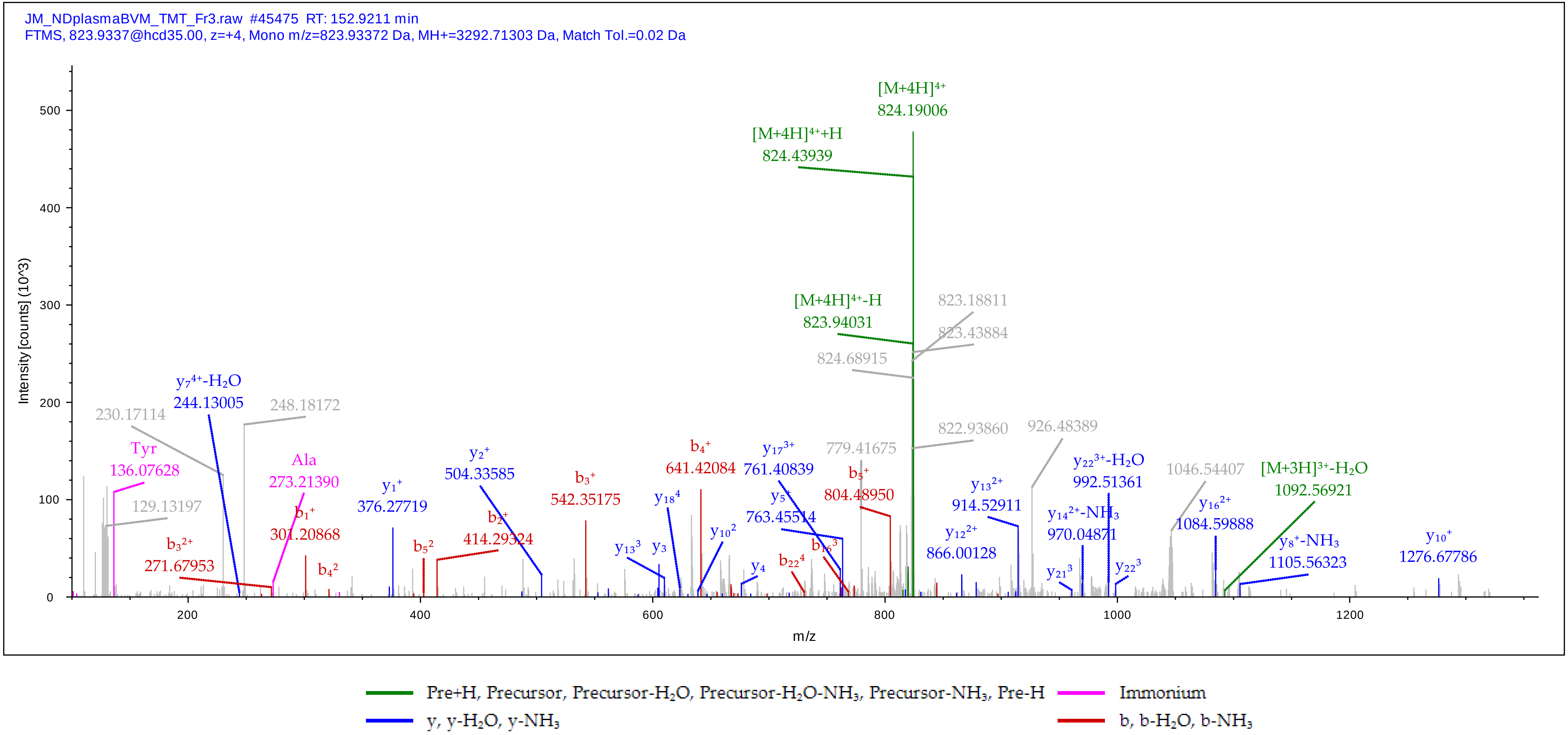
JM_NDplasmaBVM_TMT_Fr3.raw #45475 RT: 152.9211 min
FTMS, 823.9337@hcd35.00, z=+4, Mono m/z=823.93372 Da, MH+=3292.71303 Da, Match Tol.=0.02 Da
Intensity [counts] (10^3)
m/z
[M+4H]4+
824.19006
[M+4H]4++H
824.43939
[M+4H]4+-H
823.94031
823.18811
823.43884
824.68915
822.93860
926.48389
779.41675
y7 4+-H2O
244.13005
230.17114
248.18172
Tyr
136.07628
129.13197
Ala
273.21390
b1+
301.20868
b3 2+
271.67953
y1+
376.27719
y2+
504.33585
b2+
414.29324
b5 2
b4 2
b3+
542.35175
b4+
641.42084
y17 3+
761.40839
y5+
763.45514
b5+
804.48950
y18 4
y13 3
y3
y10 2
y4
b22 4
b16 3
y13 2+
914.52911
y12 2+
866.00128
y22 3+-H2O
992.51361
y14 2+-NH3
970.04871
y21 3
y22 3
1046.54407
y16 2+
1084.59888
[M+3H]3+-H2O
1092.56921
y8+-NH3
1105.56323
y10+
1276.67786
Pre+H, Precursor, Precursor-H2O, Precursor-H2O-NH3, Precursor-NH3, Pre-H
Immonium
y, y-H2O, y-NH3
b, b-H2O, b-NH3

#1	Immonium	b⁺	b²⁺	b³⁺	Seq.	y⁺	y²⁺	y³⁺	#2
1	330.23387	358.22879	179.61803	120.08111	Q-TMT6plex				21
2	101.07094	486.28736	243.64732	162.76731	Q	2588.26525	1294.63627	863.42660	20
3	120.08078	633.35578	317.18153	211.79011	F	2460.20668	1230.60698	820.74041	19
4	86.09643	746.43984	373.72356	249.48480	L	2313.13826	1157.07277	771.71761	18
5	72.08078	845.50826	423.25777	282.50760	V	2200.05420	1100.53074	734.02292	17
6	136.07569	1008.57158	504.78943	336.86205	Y	2100.98578	1050.99653	701.00011	16
7	133.04301	1168.60223	584.80475	390.20560	C-Carbamidomethyl	1937.92246	969.46487	646.64567	15
8	102.05496	1297.64483	649.32605	433.21979	E	1777.89181	889.44954	593.30212	14
9	86.09643	1410.72889	705.86808	470.91448	I	1648.84921	824.92825	550.28792	13
10	88.03930	1525.75583	763.38155	509.25680	D	1535.76515	768.38621	512.59323	12
11	30.03383	1582.77730	791.89229	528.26395	G	1420.73821	710.87274	474.25092	11
12	60.04439	1669.80932	835.40830	557.27463	S	1363.71674	682.36201	455.24377	10
13	30.03383	1726.83079	863.91903	576.28178	G	1276.68472	638.84600	426.23309	9
14	87.05529	1840.87372	920.94050	614.29609	N	1219.66325	610.33526	407.22594	8
15	30.03383	1897.89518	949.45123	633.30324	G	1105.62032	553.31380	369.21163	7
16	204.07675	2128.95957	1064.98342	710.32471	W-Nitro	1048.59886	524.80307	350.20447	6
17	74.06004	2230.00725	1115.50726	744.00727	T	817.53447	409.27087	273.18301	5
18	72.08078	2329.07566	1165.04147	777.03007	V	716.48679	358.74703	239.50045	4
19	86.09643	2442.15973	1221.58350	814.72476	L	617.41838	309.21283	206.47764	3
20	101.07094	2570.21830	1285.61279	857.41095	Q	504.33431	252.67080	168.78296	2
21	330.27026				K-TMT6plex	376.27574	188.64151	126.09676	1

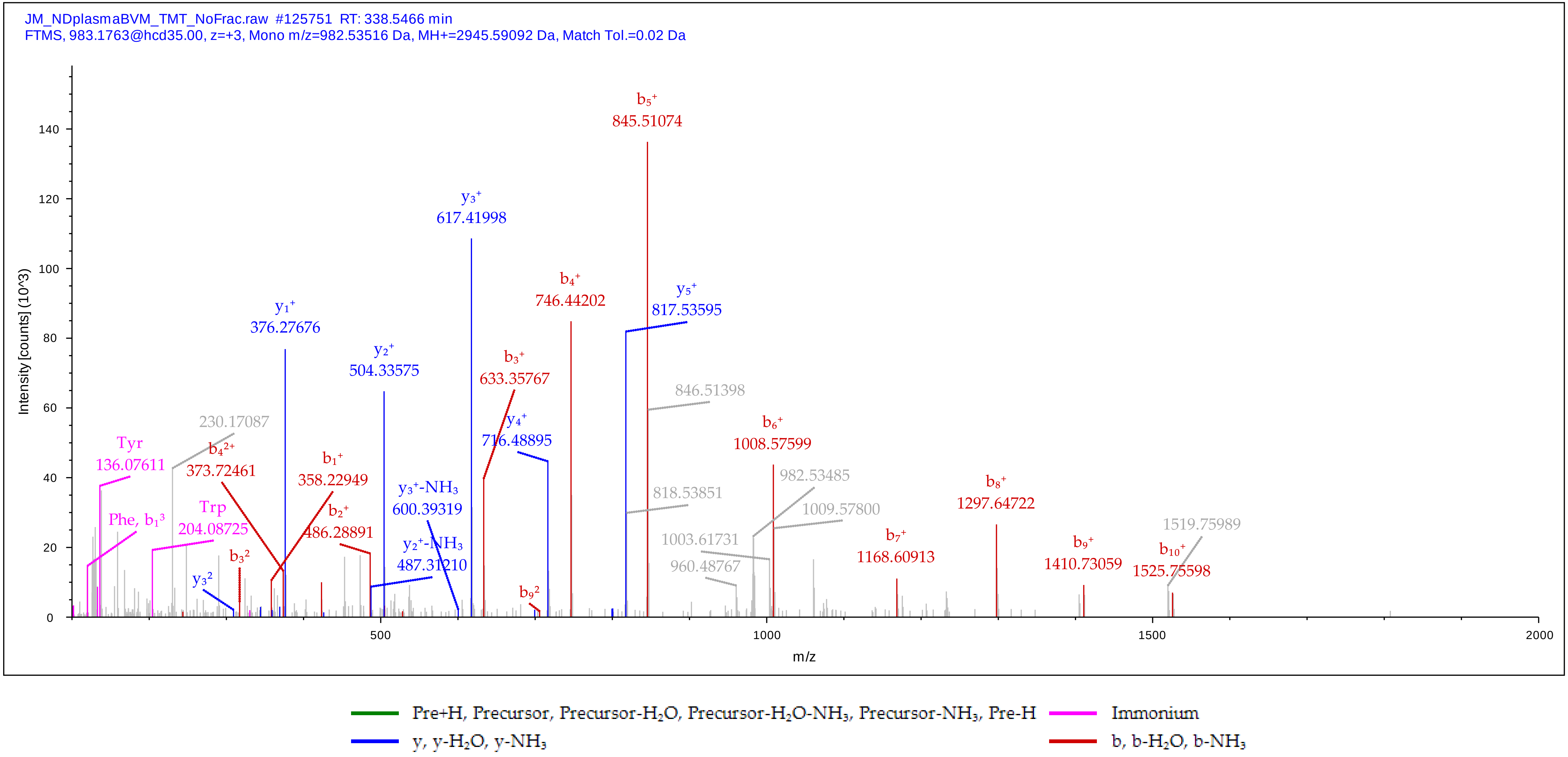

JM_NDplasmaBVM_TMT_NoFrac.raw #125751 RT: 338.5466 min
FTMS, 983.1763@hcd35.00, z=+3, Mono m/z=982.53516 Da, MH+=2945.59092 Da, Match Tol.=0.02 Da
Intensity [counts] (10^3)
m/z
b5+ 845.51074
y3+ 617.41998
b4+ 746.44202
y5+ 817.53595
y1+ 376.27676
y2+ 504.33575
b3+ 633.35767
846.51398
y4+ 716.48895
230.17087
b6+ 1008.57599
Tyr 136.07611
b4 2+ 373.72461
b1+ 358.22949
982.53485
y3+-NH3 600.39319
818.53851
b8+ 1297.64722
Trp 204.08725
b2+ 486.28891
1009.57800
1519.75989
Phe, b1 3
1003.61731
b7+ 1168.60913
b9+ 1410.73059
b10+ 1525.75598
b3 2
y2+-NH3 487.31210
960.48767
y3 2
b9 2
Pre+H, Precursor, Precursor-H2O, Precursor-H2O-NH3, Precursor-NH3, Pre-H
Immonium
y, y-H2O, y-NH3
b, b-H2O, b-NH3

#1	Immonium	b^{+}	b^{2+}	b^{3+}	b^{4+}	b^{5+}	Seq.	y^{+}	y^{2+}	y^{3+}	y^{4+}	y^{5+}	#2
1	317.20224	345.19715	173.10221	115.73723	87.05475	69.84525	D-TMT6plex						31
2	86.09643	458.28122	229.64425	153.43192	115.32576	92.46206	I	3941.04854	1971.02791	1314.35436	986.01759	789.01553	30
3	70.06513	555.33398	278.17063	185.78284	139.58895	111.87262	P	3827.96447	1914.48588	1276.65968	957.74658	766.39872	29
4	72.08078	654.40239	327.70483	218.80565	164.35606	131.68630	V	3730.91171	1865.95949	1244.30875	933.48338	746.98816	28
5	88.03930	769.42934	385.21831	257.14796	193.11279	154.69169	D	3631.84330	1816.42529	1211.28595	908.71628	727.17448	27
6	60.04439	856.46136	428.73432	286.15864	214.87080	172.09809	S	3516.81635	1758.91181	1172.94364	879.95955	704.16909	26
7	70.06513	953.51413	477.26070	318.50956	239.13399	191.50865	P	3429.78432	1715.39580	1143.93296	858.20154	686.76269	25
8	102.05496	1082.55672	541.78200	361.52376	271.39464	217.31717	E	3332.73156	1666.86942	1111.58204	833.93835	667.35213	24
9	86.09643	1195.64079	598.32403	399.21845	299.66565	239.93398	L	3203.68897	1602.34812	1068.56784	801.67770	641.54361	23
10	330.27026	1552.89868	776.95298	518.30441	388.98013	311.38556	K-TMT6plex	3090.60490	1545.80609	1030.87315	773.40668	618.92680	22
11	102.05496	1681.94127	841.47427	561.31861	421.24078	337.19408	E	2733.34701	1367.17714	911.78719	684.09221	547.47522	21
12	72.08078	1781.00969	891.00848	594.34141	446.00788	357.00776	V	2604.30442	1302.65585	868.77299	651.83156	521.66670	20
13	86.09643	1894.09375	947.55051	632.03610	474.27890	379.62457	I	2505.23600	1253.12164	835.75019	627.06446	501.85302	19
14	30.03383	1951.11521	976.06125	651.04326	488.53426	391.02886	G	2392.15194	1196.57961	798.05550	598.79344	479.23621	18
15	110.07127	2088.17413	1044.59070	696.72956	522.79899	418.44065	H	2335.13047	1168.06888	779.04834	584.53808	467.83192	17
16	60.04439	2175.20615	1088.10672	725.74024	544.55700	435.84705	S	2198.07156	1099.53942	733.36204	550.27335	440.42013	16
17	86.09643	2288.29022	1144.64875	763.43492	572.82801	458.46387	I	2111.03953	1056.02341	704.35136	528.51534	423.01373	15
18	44.04948	2359.32733	1180.16730	787.11396	590.58729	472.67129	A	1997.95547	999.48137	666.65667	500.24433	400.39692	14
19	101.07094	2487.38591	1244.19659	829.80015	622.60193	498.28300	Q	1926.91836	963.96282	642.97764	482.48505	386.18949	13
20	86.09643	2600.46997	1300.73863	867.49484	650.87295	520.89982	L	1798.85978	899.93353	600.29144	450.47040	360.57778	12
21	87.05529	2714.51290	1357.76009	905.50915	679.38368	543.70840	N	1685.77572	843.39150	562.59676	422.19939	337.96096	11
22	74.06004	2815.56058	1408.28393	939.19171	704.64560	563.91794	T	1571.73279	786.37003	524.58245	393.68865	315.15238	10
23	102.05496	2944.60317	1472.80522	982.20591	736.90625	589.72646	E	1470.68511	735.84619	490.89989	368.42673	294.94284	9
24	87.05529	3058.64610	1529.82669	1020.22022	765.41698	612.53504	N	1341.64252	671.32490	447.88569	336.16609	269.13432	8
25	88.03930	3173.67304	1587.34016	1058.56253	794.17372	635.54043	D	1227.59959	614.30343	409.87138	307.65535	246.32574	7
26	110.07127	3310.73195	1655.86962	1104.24884	828.43845	662.95221	H	1112.57265	556.78996	371.52907	278.89862	223.32035	6
27	70.06513	3407.78472	1704.39600	1136.59976	852.70164	682.36277	P	975.51373	488.26051	325.84276	244.63389	195.90857	5
28	120.08078	3554.85313	1777.93020	1185.62256	889.46874	711.77645	F	878.46097	439.73412	293.49184	220.37070	176.49802	4
29	181.06077	3762.90154	1881.95441	1254.97203	941.48084	753.38613	Y-Nitro	731.39256	366.19992	244.46904	183.60360	147.08433	3
30	120.08078	3909.96995	1955.48861	1303.99484	978.24795	782.79981	F	523.34415	262.17571	175.11957	131.59150	105.47465	2
31	330.27026						K-TMT6plex	376.27574	188.64151	126.09676	94.82439	76.06097	1

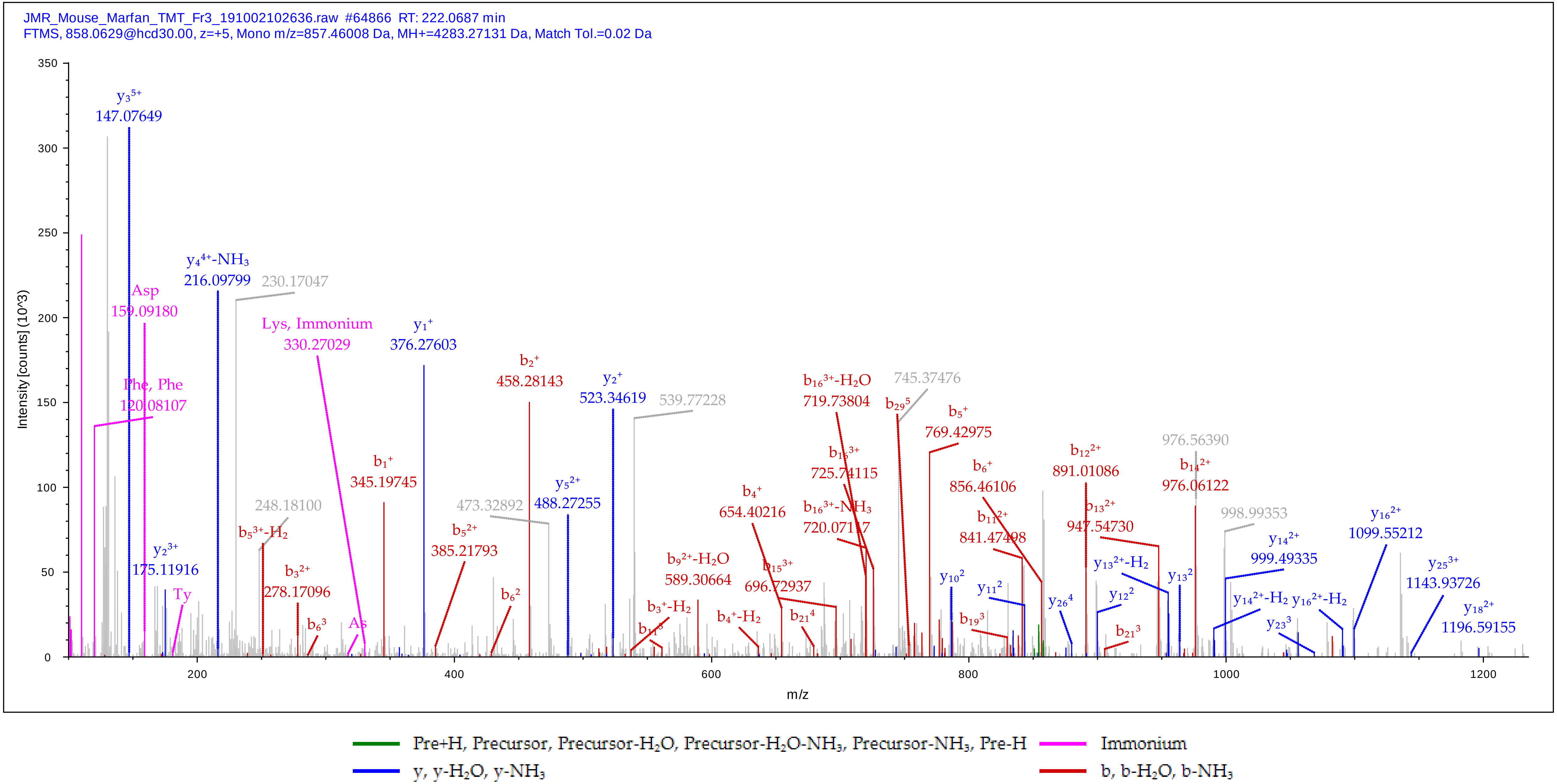
JMR_Mouse_Marfan_TMT_Fr3_191002102636.raw #64866 RT: 222.0687 min
FTMS, 858.0629@hcd30.00, z=+5, Mono m/z=857.46008 Da, MH+=4283.27131 Da, Match Tol.=0.02 Da
Intensity [counts] (10^3)
m/z
Pre+H, Precursor, Precursor-H2O, Precursor-H2O-NH3, Precursor-NH3, Pre-H
Immonium
y, y-H2O, y-NH3
b, b-H2O, b-NH3

#1	Immonium	b+	b2+	b3+	b4+	Seq.	y+	y2+	y3+	y4+	#2
1	331.21789	359.21280	180.11004	120.40912	90.55866	E-TMT6plex					21
2	72.08078	458.28122	229.64425	153.43192	115.32576	V	2604.30442	1302.65585	868.77299	651.83156	20
3	86.09643	571.36528	286.18628	191.12661	143.59678	L	2505.23600	1253.12164	835.75019	627.06446	19
4	30.03383	628.38674	314.69701	210.13377	157.85214	G	2392.15194	1196.57961	798.05550	598.79344	18
5	110.07127	765.44565	383.22647	255.82007	192.11687	H	2335.13047	1168.06888	779.04834	584.53808	17
6	60.04439	852.47768	426.74248	284.83075	213.87488	S	2198.07156	1099.53942	733.36204	550.27335	16
7	86.09643	965.56175	483.28451	322.52543	242.14589	I	2111.03953	1056.02341	704.35136	528.51534	15
8	44.04948	1036.59886	518.80307	346.20447	259.90517	A	1997.95547	999.48137	666.65667	500.24433	14
9	101.07094	1164.65744	582.83236	388.89066	291.91982	Q	1926.91836	963.96282	642.97764	482.48505	13
10	86.09643	1277.74150	639.37439	426.58535	320.19083	L	1798.85978	899.93353	600.29144	450.47040	12
11	87.05529	1391.78443	696.39585	464.59966	348.70156	N	1685.77572	843.39150	562.59676	422.19939	11
12	74.06004	1492.83211	746.91969	498.28222	373.96348	T	1571.73279	786.37003	524.58245	393.68865	10
13	102.05496	1621.87470	811.44099	541.29642	406.22413	E	1470.68511	735.84619	490.89989	368.42673	9
14	87.05529	1735.91763	868.46245	579.31073	434.73486	N	1341.64252	671.32490	447.88569	336.16609	8
15	88.03930	1850.94457	925.97592	617.65304	463.49160	D	1227.59959	614.30343	409.87138	307.65535	7
16	110.07127	1988.00348	994.50538	663.33935	497.75633	H	1112.57265	556.78996	371.52907	278.89862	6
17	70.06513	2085.05625	1043.03176	695.69027	522.01952	P	975.51373	488.26051	325.84276	244.63389	5
18	120.08078	2232.12466	1116.56597	744.71307	558.78662	F	878.46097	439.73412	293.49184	220.37070	4
19	181.06077	2440.17307	1220.59017	814.06254	610.79872	Y-Nitro	731.39256	366.19992	244.46904	183.60360	3
20	120.08078	2587.24148	1294.12438	863.08534	647.56583	F	523.34415	262.17571	175.11957	131.59150	2
21	330.27026					K-TMT6plex	376.27574	188.64151	126.09676	94.82439	1

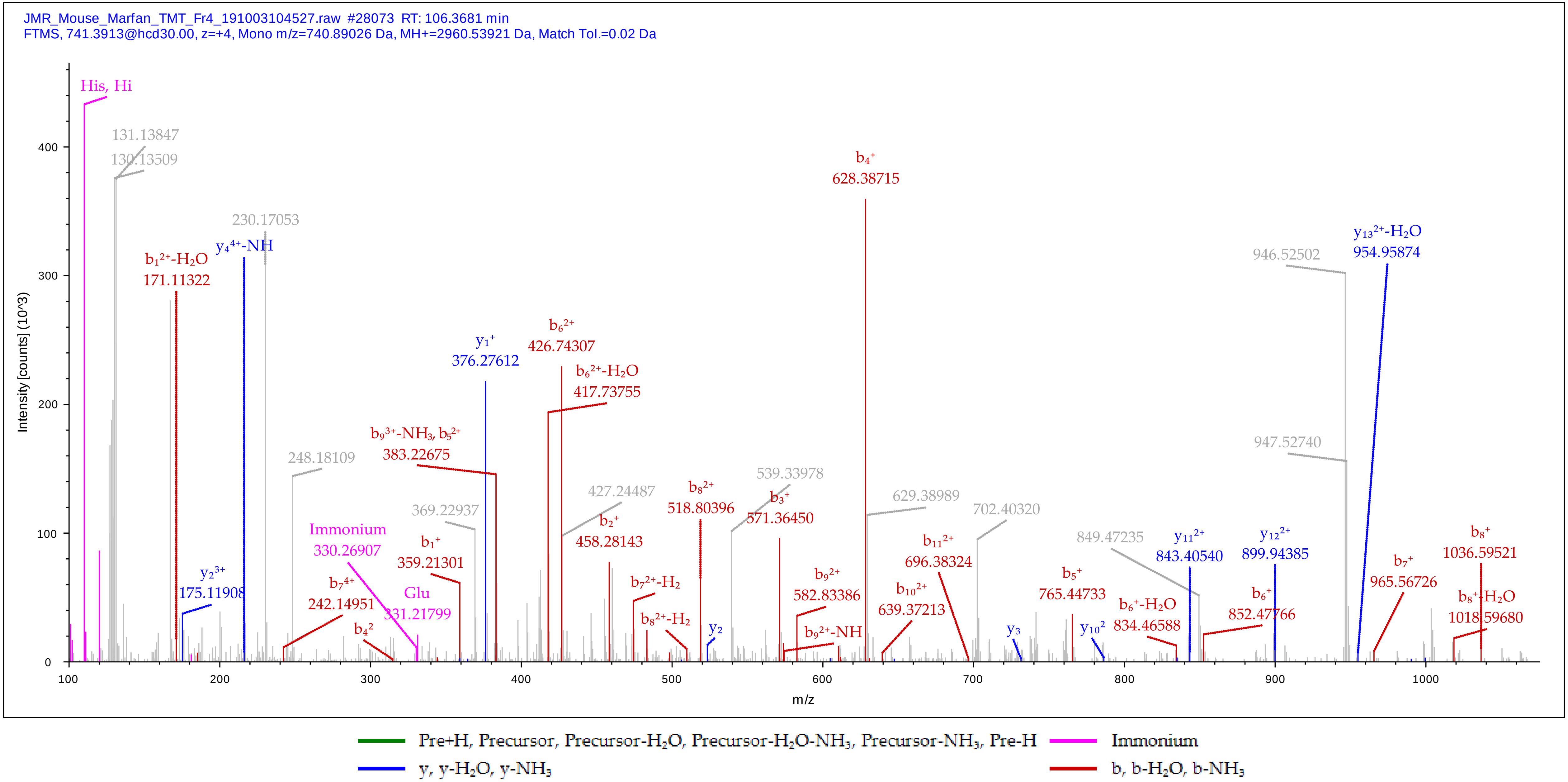
JMR_Mouse_Marfan_TMT_Fr4_191003104527.raw #28073 RT: 106.3681 min
FTMS, 741.3913@hcd30.00, z=+4, Mono m/z=740.89026 Da, MH+=2960.53921 Da, Match Tol.=0.02 Da
His, Hi
131.13847
130.13509
230.17053
b1 2+-H2O 171.11322
y4 4+-NH
y1+ 376.27612
b6 2+ 426.74307
b6 2+-H2O 417.73755
b4+ 628.38715
y13 2+-H2O 954.95874
946.52502
947.52740
248.18109
b9 3+-NH3, b5 2+ 383.22675
369.22937
427.24487
b2+ 458.28143
b8 2+ 518.80396
539.33978
b3+ 571.36450
629.38989
702.40320
849.47235
y11 2+ 843.40540
y12 2+ 899.94385
b8+ 1036.59521
Immonium 330.26907
b1+ 359.21301
b11 2+ 696.38324
b7+ 965.56726
y2 3+ 175.11908
b7 4+ 242.14951
Glu 331.21799
b7 2+-H2
b9 2+ 582.83386
b10 2+ 639.37213
b5+ 765.44733
b6+ 852.47766
b8+-H2O 1018.59680
b6+-H2O 834.46588
b4 2
b8 2+-H2
y2
b9 2+-NH
y3
y10 2
Intensity [counts] (10^3)
m/z
Pre+H, Precursor, Precursor-H2O, Precursor-H2O-NH3, Precursor-NH3, Pre-H
Immonium
y, y-H2O, y-NH3
b, b-H2O, b-NH3
