# Supplementary material for: Aortic disease in Marfan syndrome is caused by overactivation of sGC-PRKG signaling by NO
Source: Nat Commun. 2021 May 11;12:2628. doi: 10.1038/s41467-021-22933-3 (PMC8113458; doi:10.1038/s41467-021-22933-3)
Supplement: Supplementary file 7 — Supplementary Data 4 [file 41467_2021_22933_MOESM7_ESM.pdf]

#1	Immonium	b^+	b^{2+}	b^{3+}	Seq.	y^+	y^{2+}	y^{3+}	#2
1	315.25936	343.25427	172.13077	115.08961	L-TMT6plex				18
2	86.09643	456.33834	228.67281	152.78430	L	2206.16269	1103.58498	736.05908	17
3	86.09643	569.42240	285.21484	190.47898	L	2093.07862	1047.04295	698.36439	16
4	101.07094	697.48098	349.24413	233.16518	Q	1979.99456	990.50092	660.66970	15
5	101.07094	825.53956	413.27342	275.85137	Q	1851.93598	926.47163	617.98351	14
6	72.08078	924.60797	462.80762	308.87417	V	1723.87740	862.44234	575.29732	13
7	60.04439	1011.64000	506.32364	337.88485	S	1624.80899	812.90813	542.27451	12
8	86.09643	1124.72406	562.86567	375.57954	L	1537.77696	769.39212	513.26384	11
9	70.06513	1221.77683	611.39205	407.93046	P	1424.69290	712.85009	475.56915	10
10	102.05496	1350.81942	675.91335	450.94466	E	1327.64013	664.32370	443.21823	9
11	86.09643	1463.90348	732.45538	488.63935	L	1198.59754	599.80241	400.20403	8
12	70.06513	1560.95625	780.98176	520.99027	P	1085.51348	543.26038	362.50934	7
13	30.03383	1617.97771	809.49249	539.99742	G	988.46071	494.73399	330.15842	6
14	102.05496	1747.02030	874.01379	583.01162	E	931.43925	466.22326	311.15127	5
15	181.06077	1955.06871	978.03799	652.36109	Y-Nitro	802.39666	401.70197	268.13707	4
16	60.04439	2042.10074	1021.55401	681.37176	S	594.34825	297.67776	198.78760	3
17	104.05285	2173.14122	1087.07425	725.05193	M	507.31622	254.16175	169.77692	2
18	330.27026				K-TMT6plex	376.27574	188.64151	126.09676	1

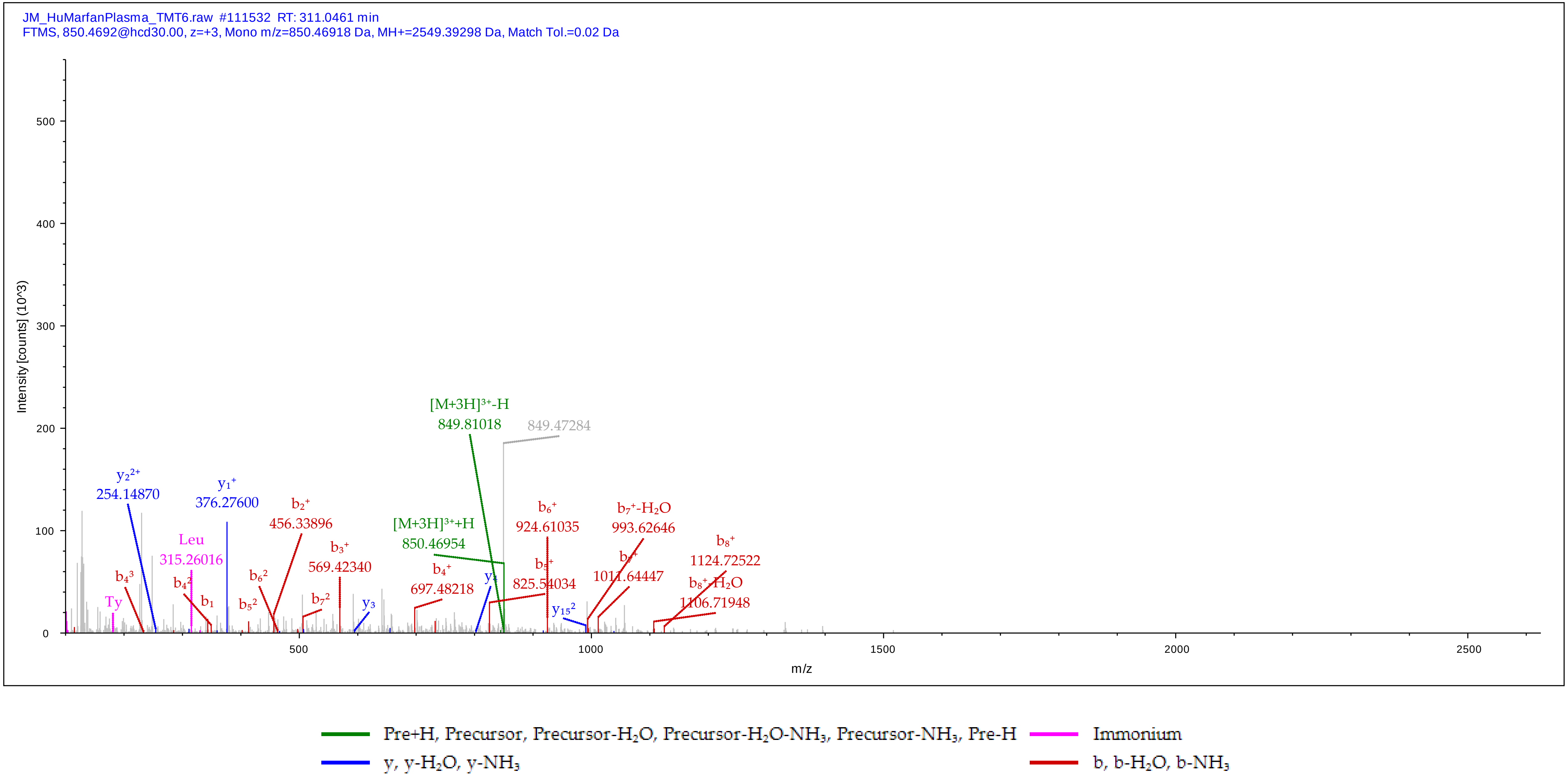
JM_HuMarfanPlasma_TMT6.raw #111532 RT: 311.0461 min
FTMS, 850.4692@hcd30.00, z=+3, Mono m/z=850.46918 Da, MH+=2549.39298 Da, Match Tol.=0.02 Da
Intensity [counts] (10^3)
m/z
0
100
200
300
400
500
1000
1500
2000
2500
y2 2+ 254.14870
y1+ 376.27600
b2+ 456.33896
b3+ 569.42340
Leu 315.26016
Ty
b4 3
b4 2
b1
b5 2
b6 2
b7 2
y3
[M+3H]3+-H 849.81018
849.47284
[M+3H]3++H 850.46954
b4+ 697.48218
b6+ 924.61035
b5+ 825.54034
y15 2
b7+-H2O 993.62646
b7+ 1011.64447
b8+ 1124.72522
b8+-H2O 1106.71948
Pre+H, Precursor, Precursor-H2O, Precursor-H2O-NH3, Precursor-NH3, Pre-H
Immonium
y, y-H2O, y-NH3
b, b-H2O, b-NH3

#1	Immonium	b+	b2+	b3+	b4+	Seq.	y+	y2+	y3+	y4+	#2
1	273.21241	301.20732	151.10730	101.07396	76.05729	A-TMT6plex					27
2	72.08078	400.27574	200.64151	134.09676	100.82439	V	3349.79653	1675.40190	1117.27036	838.20459	26
3	88.03930	515.30268	258.15498	172.43908	129.58113	D	3250.72811	1625.86770	1084.24756	813.43749	25
4	101.07094	643.36126	322.18427	215.12527	161.59577	Q	3135.70117	1568.35422	1045.90524	784.68075	24
5	60.04439	730.39328	365.70028	244.13595	183.35378	S	3007.64259	1504.32494	1003.21905	752.66611	23
6	72.08078	829.46170	415.23449	277.15875	208.12088	V	2920.61057	1460.80892	974.20837	730.90810	22
7	86.09643	942.54576	471.77652	314.85344	236.39190	L	2821.54215	1411.27471	941.18557	706.14100	21
8	86.09643	1055.62983	528.31855	352.54813	264.66291	L	2708.45809	1354.73268	903.49088	677.86998	20
9	104.05285	1186.67031	593.83879	396.22829	297.42304	M	2595.37402	1298.19065	865.79619	649.59896	19
10	330.27026	1543.92821	772.46774	515.31425	386.73751	K-TMT6plex	2464.33354	1232.67041	822.11603	616.83884	18
11	70.06513	1640.98097	820.99412	547.66517	411.00070	P	2107.07564	1054.04146	703.03007	527.52437	17
12	88.03930	1756.00791	878.50759	586.00749	439.75744	D	2010.02288	1005.51508	670.67914	503.26118	16
13	44.04948	1827.04503	914.02615	609.68653	457.51671	A	1894.99594	948.00161	632.33683	474.50444	15
14	102.05496	1956.08762	978.54745	652.70072	489.77736	E	1823.95882	912.48305	608.65779	456.74516	14
15	86.09643	2069.17168	1035.08948	690.39541	518.04838	L	1694.91623	847.96175	565.64359	424.48452	13
16	60.04439	2156.20371	1078.60549	719.40609	539.80639	S	1581.83217	791.41972	527.94891	396.21350	12
17	44.04948	2227.24083	1114.12405	743.08513	557.56566	A	1494.80014	747.90371	498.93823	374.45549	11
18	60.04439	2314.27285	1157.64007	772.09580	579.32367	S	1423.76303	712.38515	475.25919	356.69621	10
19	60.04439	2401.30488	1201.15608	801.10648	601.08168	S	1336.73100	668.86914	446.24852	334.93821	9
20	72.08078	2500.37330	1250.69029	834.12928	625.84878	V	1249.69897	625.35312	417.23784	313.18020	8
21	181.06077	2708.42170	1354.71449	903.47875	677.86088	Y-Nitro	1150.63055	575.81892	384.21504	288.41310	7
22	87.05529	2822.46463	1411.73595	941.49306	706.37162	N	942.58215	471.79471	314.86557	236.40099	6
23	86.09643	2935.54869	1468.27799	979.18775	734.64263	L	828.53922	414.77325	276.85126	207.89026	5
24	86.09643	3048.63276	1524.82002	1016.88244	762.91365	L	715.45516	358.23122	239.15657	179.61925	4
25	70.06513	3145.68552	1573.34640	1049.23336	787.17684	P	602.37109	301.68918	201.46188	151.34823	3
26	102.05496	3274.72811	1637.86770	1092.24756	819.43749	E	505.31833	253.16280	169.11096	127.08504	2
27	330.27026					K-TMT6plex	376.27574	188.64151	126.09676	94.82439	1

Nitro-Tyr immonium ion is detected in MS/MS spectra and added brown colored in the following spectrum

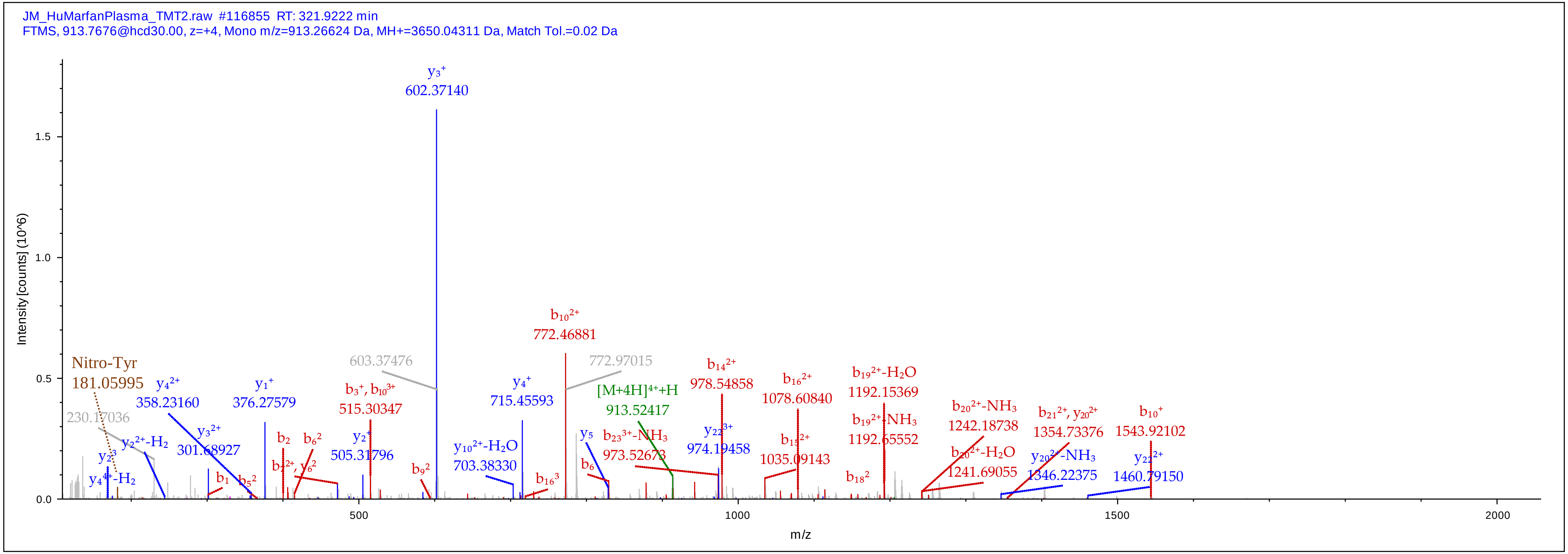

Pre+H, Precursor, Precursor-H_2O, Precursor-H_2O-NH_3, Precursor-NH_3, Pre-H

Immonium

y, y-H_2O, y-NH_3

b, b-H_2O, b-NH_3

#1	Immonium	b⁺	b²⁺	b³⁺	b⁴⁺	Seq.	y⁺	y²⁺	y³⁺	y⁴⁺	#2
1	362.20594	390.20086	195.60407	130.73847	98.30567	C-TMT6plex-Carbamidomethyl					13
2	133.04301	550.23151	275.61939	184.08202	138.31333	C-Carbamidomethyl	1666.71511	833.86119	556.24322	417.43423	12
3	44.04948	621.26862	311.13795	207.76106	156.07261	A	1506.68446	753.84587	502.89967	377.42657	11
4	44.04948	692.30573	346.65650	231.44010	173.83189	A	1435.64734	718.32731	479.22063	359.66729	10
5	44.04948	763.34285	382.17506	255.11913	191.59117	A	1364.61023	682.80875	455.54159	341.90802	9
6	88.03930	878.36979	439.68853	293.46145	220.34790	D	1293.57312	647.29020	431.86256	324.14874	8
7	70.06513	975.42255	488.21492	325.81237	244.61110	P	1178.54617	589.77673	393.52024	295.39200	7
8	110.07127	1112.48147	556.74437	371.49867	278.87582	H	1081.49341	541.25034	361.16932	271.12881	6
9	102.05496	1241.52406	621.26567	414.51287	311.13647	E	944.43450	472.72089	315.48302	236.86408	5
10	133.04301	1401.55471	701.28099	467.85642	351.14413	C-Carbamidomethyl	815.39190	408.19959	272.46882	204.60343	4
11	181.06077	1609.60311	805.30519	537.20589	403.15624	Y-Nitro	655.36126	328.18427	219.12527	164.59577	3
12	44.04948	1680.64023	840.82375	560.88493	420.91551	A	447.31285	224.16006	149.77580	112.58367	2
13	330.27026					K-TMT6plex	376.27574	188.64151	126.09676	94.82439	1

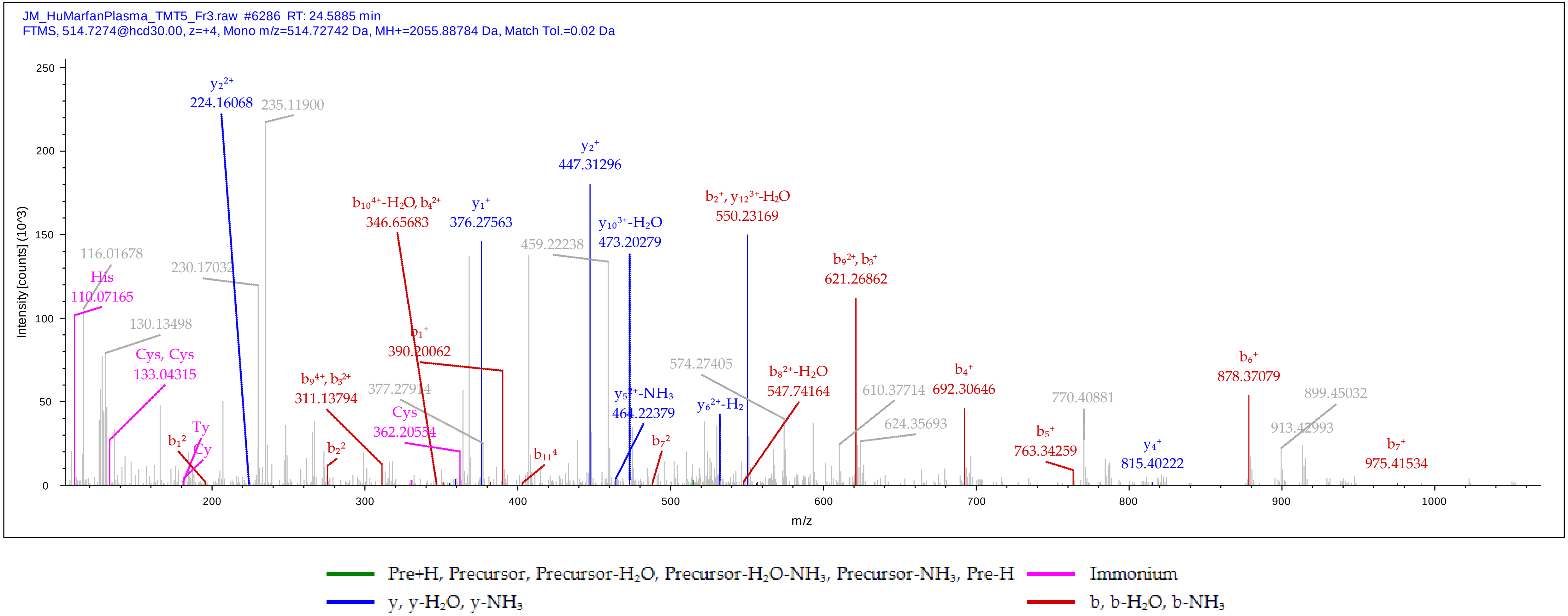
JM_HuMarfanPlasma_TMT5_Fr3.raw #6286 RT: 24.5885 min
FTMS, 514.7274@hcd30.00, z=+4, Mono m/z=514.72742 Da, MH+=2055.88784 Da, Match Tol.=0.02 Da
Intensity [counts] (10^3)
m/z
y2 2+ 224.16068
235.11900
y2+ 447.31296
b10 4+-H2O, b4 2+ 346.65683
y1+ 376.27563
y10 3+-H2O 473.20279
b2+, y12 3+-H2O 550.23169
459.22238
116.01678
230.17032
His 110.07165
b9 2+, b3+ 621.26862
130.13498
Cys, Cys 133.04315
b1+ 390.20062
574.27405
b8 2+-H2O 547.74164
b4+ 692.30646
b6+ 878.37079
b9 4+, b3 2+ 311.13794
377.27914
610.37714
770.40881
899.45032
y5 2+-NH3 464.22379
y6 2+-H2
Cys 362.20554
624.35693
913.42993
b5+ 763.34259
y4+ 815.40222
b7+ 975.41534
Pre+H, Precursor, Precursor-H2O, Precursor-H2O-NH3, Precursor-NH3, Pre-H
Immonium
y, y-H2O, y-NH3
b, b-H2O, b-NH3

#1	Immonium	b^+	b^{2+}	Seq.	y^+	y^{2+}	#2
1	410.22370	438.21862	219.61295	Y-TMT6plex-Nitro			7
2	86.09643	551.30268	276.15498	L	764.43012	382.71870	6
3	136.07569	714.36601	357.68664	Y	651.34605	326.17666	5
4	102.05496	843.40860	422.20794	E	488.28272	244.64500	4
5	86.09643	956.49266	478.74997	I	359.24013	180.12370	3
6	44.04948	1027.52978	514.26853	A	246.15607	123.58167	2
7	129.11347			R	175.11895	88.06311	1

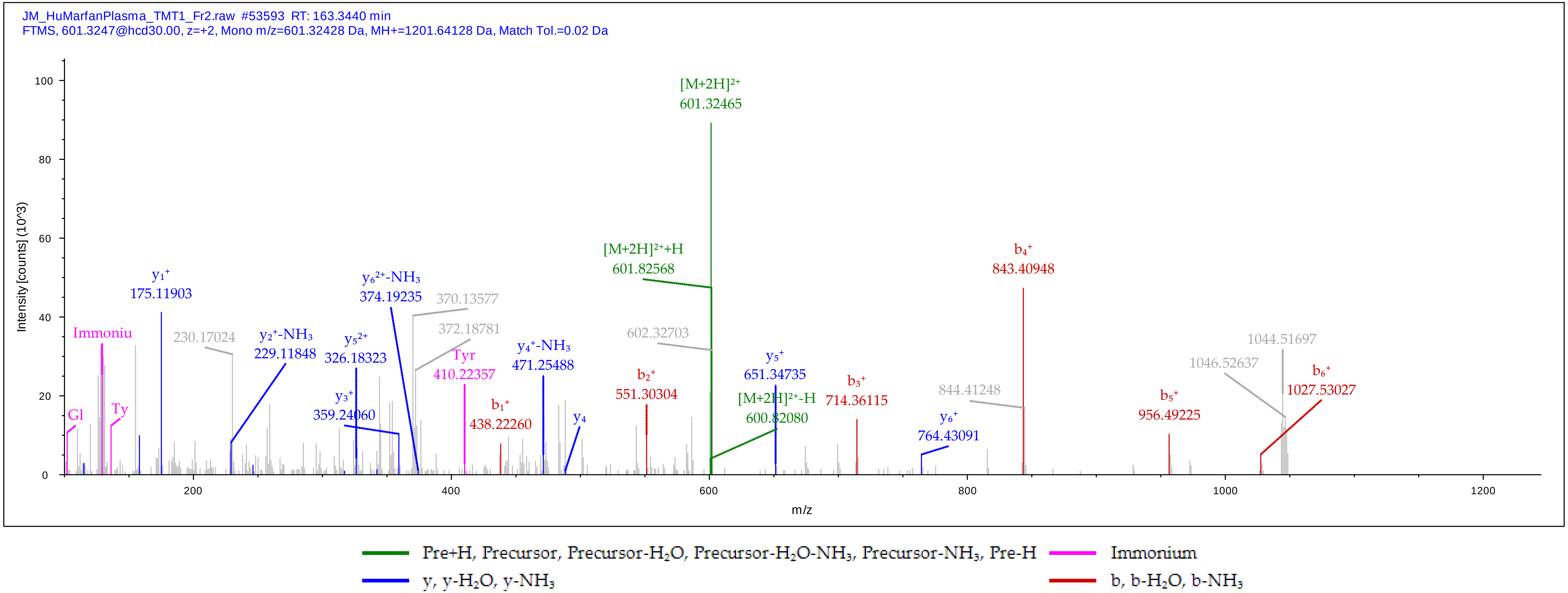

JM_HuMarfanPlasma_TMT1_Fr2.raw #53593 RT: 163.3440 min
FTMS, 601.3247@hcd30.00, z=+2, Mono m/z=601.32428 Da, MH+=1201.64128 Da, Match Tol.=0.02 Da
Intensity [counts] (10^3)
m/z
[M+2H]²⁺ 601.32465
[M+2H]²⁺+H 601.82568
b₄⁺ 843.40948
y₁⁺ 175.11903
y₆²⁺-NH₃ 374.19235
370.13577
372.18781
230.17024
y₂⁺-NH₃ 229.11848
y₅²⁺ 326.18323
Tyr 410.22357
y₄⁺-NH₃ 471.25488
602.32703
y₅⁺ 651.34735
b₂⁺ 551.30304
b₃⁺ 714.36115
[M+2H]²⁺-H 600.82080
y₃⁺ 359.24060
b₁⁺ 438.22260
y₄
844.41248
y₆⁺ 764.43091
1044.51697
1046.52637
b₆⁺ 1027.53027
b₅⁺ 956.49225
Immoniu
Gl
Ty
Pre+H, Precursor, Precursor-H₂O, Precursor-H₂O-NH₃, Precursor-NH₃, Pre-H
Immonium
y, y-H₂O, y-NH₃
b, b-H₂O, b-NH₃

#1	Immonium	b^+	b^{2+}	Seq.	y^+	y^{2+}	#2
1	410.22370	438.21862	219.61295	Y-TMT6plex-Nitro			12
2	86.09643	551.30268	276.15498	I	1509.74164	755.37446	11
3	133.04301	711.33333	356.17030	C-Carbamidomethyl	1396.65757	698.83243	10
4	102.05496	840.37592	420.69160	E	1236.62693	618.81710	9
5	87.05529	954.41885	477.71306	N	1107.58433	554.29580	8
6	101.07094	1082.47743	541.74235	Q	993.54141	497.27434	7
7	88.03930	1197.50437	599.25582	D	865.48283	433.24505	6
8	60.04439	1284.53640	642.77184	S	750.45589	375.73158	5
9	86.09643	1397.62046	699.31387	I	663.42386	332.21557	4
10	60.04439	1484.65249	742.82988	S	550.33979	275.67353	3
11	60.04439	1571.68452	786.34590	S	463.30776	232.15752	2
12	330.27026			K-TMT6plex	376.27574	188.64151	1

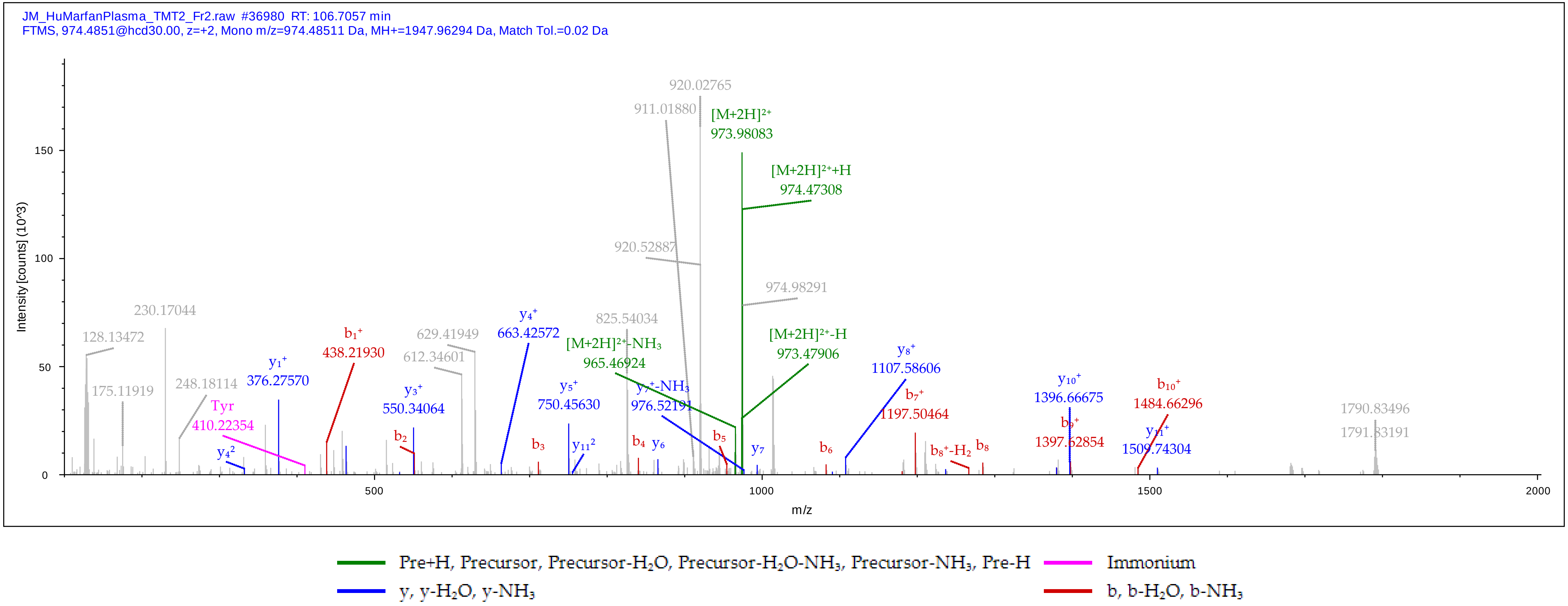

JM_HuMarfanPlasma_TMT2_Fr2.raw #36980 RT: 106.7057 min
FTMS, 974.4851@hcd30.00, z=+2, Mono m/z=974.48511 Da, MH+=1947.96294 Da, Match Tol.=0.02 Da
Intensity [counts] (10^3)
m/z
Pre+H, Precursor, Precursor-H2O, Precursor-H2O-NH3, Precursor-NH3, Pre-H
Immonium
y, y-H2O, y-NH3
b, b-H2O, b-NH3

#1	Immonium	b⁺	b²⁺	b³⁺	b⁴⁺	b⁵⁺	Seq.	y⁺	y²⁺	y³⁺	y⁴⁺	y⁵⁺	#2
1	358.27640	386.27132	193.63930	129.42862	97.32329	78.06009	R-TMT6plex						22
2	104.05285	517.31180	259.15954	173.10879	130.08341	104.26818	M	2792.36187	1396.68457	931.45881	698.84593	559.27820	21
3	70.06513	614.36457	307.68592	205.45971	154.34660	123.67873	P	2661.32139	1331.16433	887.77865	666.08580	533.07010	20
4	133.04301	774.39522	387.70125	258.80326	194.35426	155.68486	C-Carbamidomethyl	2564.26862	1282.63795	855.42773	641.82261	513.65955	19
5	44.04948	845.43233	423.21980	282.48229	212.11354	169.89229	A	2404.23798	1202.62263	802.08418	601.81495	481.65342	18
6	102.05496	974.47492	487.74110	325.49649	244.37419	195.70081	E	2333.20086	1167.10407	778.40514	584.05567	467.44599	17
7	88.03930	1089.50187	545.25457	363.83881	273.13092	218.70619	D	2204.15827	1102.58277	735.39094	551.79502	441.63748	16
8	181.06077	1297.55027	649.27877	433.18828	325.14303	260.31588	Y-Nitro	2089.13133	1045.06930	697.04863	523.03829	418.63209	15
9	86.09643	1410.63434	705.82081	470.88296	353.41404	282.93269	L	1881.08292	941.04510	627.69916	471.02619	377.02241	14
10	60.04439	1497.66636	749.33682	499.89364	375.17205	300.33909	S	1767.99886	884.50307	590.00447	442.75517	354.40559	13
11	72.08078	1596.73478	798.87103	532.91644	399.93915	320.15278	V	1680.96683	840.98705	560.99379	420.99716	336.99919	12
12	72.08078	1695.80319	848.40523	565.93925	424.70626	339.96646	V	1581.89841	791.45285	527.97099	396.23006	317.18550	11
13	86.09643	1808.88726	904.94727	603.63394	452.97727	362.58327	L	1482.83000	741.91864	494.94818	371.46296	297.37182	10
14	87.05529	1922.93018	961.96873	641.64825	481.48800	385.39186	N	1369.74594	685.37661	457.25350	343.19194	274.75501	9
15	101.07094	2050.98876	1025.99802	684.33444	513.50265	411.00357	Q	1255.70301	628.35514	419.23919	314.68121	251.94642	8
16	86.09643	2164.07283	1082.54005	722.02913	541.77366	433.62039	L	1127.64443	564.32585	376.55299	282.66657	226.33471	7
17	133.04301	2324.10347	1162.55538	775.37268	581.78133	465.62652	C-Carbamidomethyl	1014.56037	507.78382	338.85831	254.39555	203.71789	6
18	72.08078	2423.17189	1212.08958	808.39548	606.54843	485.44020	V	854.52972	427.76850	285.51476	214.38789	171.71177	5
19	86.09643	2536.25595	1268.63161	846.09017	634.81945	508.05701	L	755.46131	378.23429	252.49195	189.62078	151.89808	4
20	110.07127	2673.31486	1337.16107	891.77647	669.08417	535.46879	H	642.37724	321.69226	214.79726	161.34977	129.28127	3
21	102.05496	2802.35746	1401.68237	934.79067	701.34482	561.27731	E	505.31833	253.16280	169.11096	127.08504	101.86949	2
22	330.27026						K-TMT6plex	376.27574	188.64151	126.09676	94.82439	76.06097	1

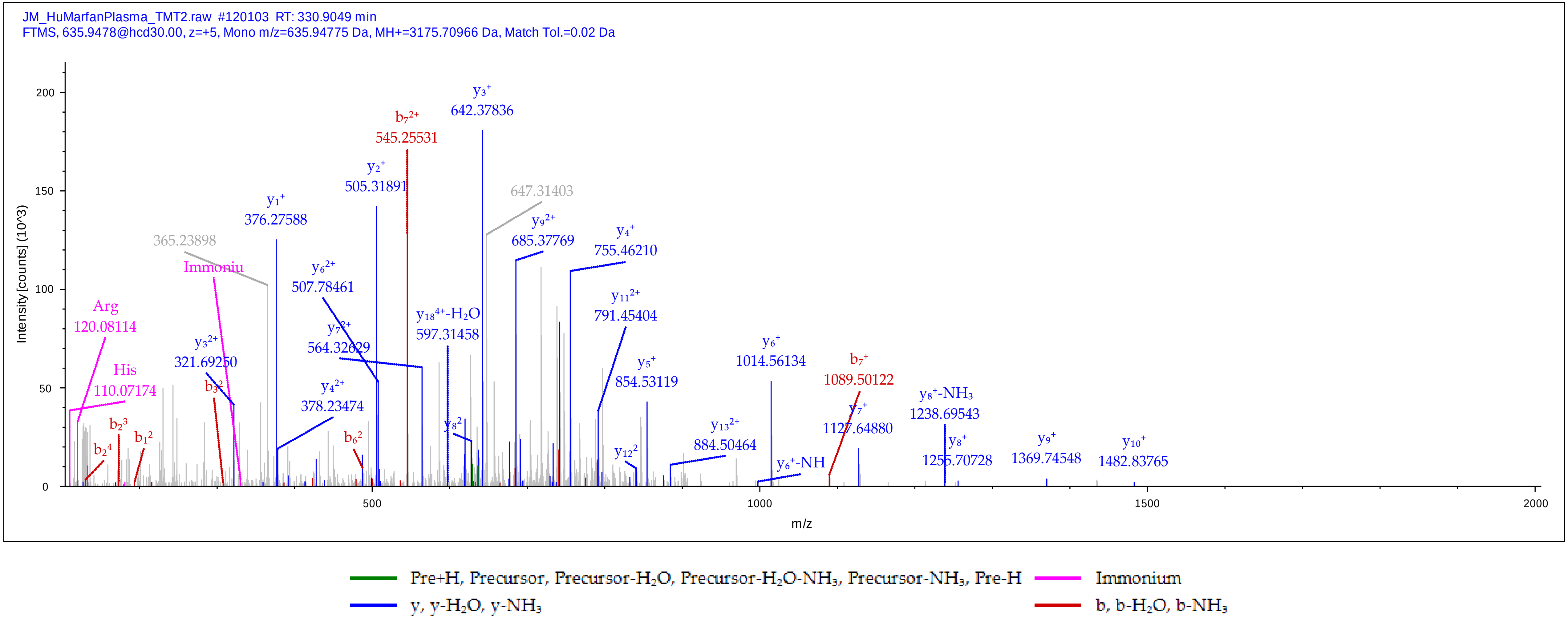

JM_HuMarfanPlasma_TMT2.raw #120103 RT: 330.9049 min
FTMS, 635.9478@hcd30.00, z=+5, Mono m/z=635.94775 Da, MH+=3175.70966 Da, Match Tol.=0.02 Da
Intensity [counts] (10^3)
m/z
Arg 120.08114
His 110.07174
Immoniu
365.23898
y1+ 376.27588
y2+ 505.31891
b72+ 545.25531
y3+ 642.37836
647.31403
y92+ 685.37769
y4+ 755.46210
y62+ 507.78461
y72+ 564.32629
y184+-H2O 597.31458
y112+ 791.45404
y32+ 321.69250
y42+ 378.23474
y5+ 854.53119
y6+ 1014.56134
b7+ 1089.50122
y7+ 1127.64880
y8+-NH3 1238.69543
y8+ 1255.70728
y9+ 1369.74548
y10+ 1482.83765
y132+ 884.50464
y6+-NH
y122
y82
b62
b32
b23
b24
b12
Pre+H, Precursor, Precursor-H2O, Precursor-H2O-NH3, Precursor-NH3, Pre-H
Immonium
y, y-H2O, y-NH3
b, b-H2O, b-NH3

#1	Immonium	b^{+}	b^{2+}	b^{3+}	b^{4+}	Seq.	y^{+}	y^{2+}	y^{3+}	y^{4+}	#2
1	358.27640	386.27132	193.63930	129.42862	97.32329	R-TMT6plex					16
2	70.06513	483.32408	242.16568	161.77955	121.58648	P	2028.97857	1014.99293	676.99771	508.00010	15
3	133.04301	643.35473	322.18100	215.12310	161.59414	C-Carbamidomethyl	1931.92581	966.46654	644.64679	483.73691	14
4	120.08078	790.42315	395.71521	264.14590	198.36124	F	1771.89516	886.45122	591.30324	443.72925	13
5	60.04439	877.45517	439.23123	293.15658	220.11925	S	1624.82675	812.91701	542.28043	406.96214	12
6	44.04948	948.49229	474.74978	316.83561	237.87853	A	1537.79472	769.40100	513.26976	385.20414	11
7	86.09643	1061.57635	531.29181	354.53030	266.14955	L	1466.75761	733.88244	489.59072	367.44486	10
8	102.05496	1190.61894	595.81311	397.54450	298.41019	E	1353.67354	677.34041	451.89603	339.17384	9
9	72.08078	1289.68736	645.34732	430.56730	323.17730	V	1224.63095	612.81911	408.88183	306.91319	8
10	88.03930	1404.71430	702.86079	468.90962	351.93403	D	1125.56253	563.28491	375.85903	282.14609	7
11	102.05496	1533.75689	767.38209	511.92382	384.19468	E	1010.53559	505.77143	337.51672	253.38936	6
12	74.06004	1634.80457	817.90592	545.60638	409.45660	T	881.49300	441.25014	294.50252	221.12871	5
13	181.06077	1842.85298	921.93013	614.95584	461.46870	Y-Nitro	780.44532	390.72630	260.81996	195.86679	4
14	72.08078	1941.92139	971.46433	647.97865	486.23581	V	572.39691	286.70210	191.47049	143.85469	3
15	70.06513	2038.97416	1019.99072	680.32957	510.49900	P	473.32850	237.16789	158.44768	119.08758	2
16	330.27026					K-TMT6plex	376.27574	188.64151	126.09676	94.82439	1

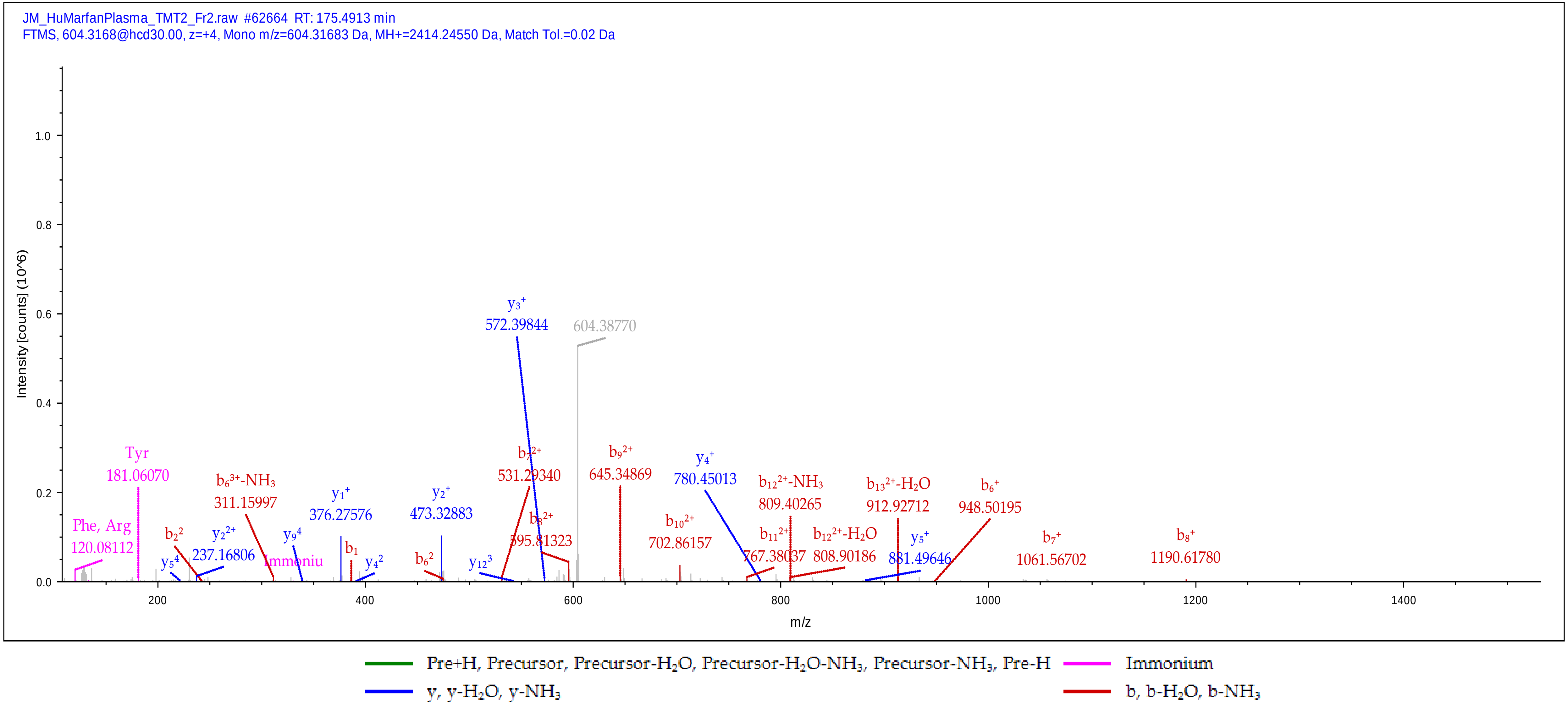

JM_HuMarfanPlasma_TMT2_Fr2.raw #62664 RT: 175.4913 min
FTMS, 604.3168@hcd30.00, z=+4, Mono m/z=604.31683 Da, MH+=2414.24550 Da, Match Tol.=0.02 Da
Intensity [counts] (10^6)
m/z
y3+ 572.39844
604.38770
Tyr 181.06070
b6 3+-NH3 311.15997
y1+ 376.27576
y2+ 473.32883
b7 2+ 531.29340
b9 2+ 645.34869
y4+ 780.45013
b12 2+-NH3 809.40265
b13 2+-H2O 912.92712
b6+ 948.50195
Phe, Arg 120.08112
b2 2
y2 2+ 237.16806
y9 4
b1
Immoniu
y5 4
y4 2
b6 2
y12 3
b8 2+ 595.81323
b10 2+ 702.86157
b11 2+ 767.38037
b12 2+-H2O 808.90186
y5+ 881.49646
b7+ 1061.56702
b8+ 1190.61780
Pre+H, Precursor, Precursor-H2O, Precursor-H2O-NH3, Precursor-NH3, Pre-H
Immonium
y, y-H2O, y-NH3
b, b-H2O, b-NH3

#1	Immonium	b+	b2+	b3+	Seq.	y+	y2+	y3+	#2
1	333.21578	361.21069	181.10898	121.07508	M-TMT6plex				21
2	70.06513	458.26346	229.63537	153.42600	P	2661.32139	1331.16433	887.77865	20
3	133.04301	618.29411	309.65069	206.76955	C-Carbamidomethyl	2564.26862	1282.63795	855.42773	19
4	44.04948	689.33122	345.16925	230.44859	A	2404.23798	1202.62263	802.08418	18
5	102.05496	818.37381	409.69054	273.46279	E	2333.20086	1167.10407	778.40514	17
6	88.03930	933.40076	467.20402	311.80510	D	2204.15827	1102.58277	735.39094	16
7	181.06077	1141.44916	571.22822	381.15457	Y-Nitro	2089.13133	1045.06930	697.04863	15
8	86.09643	1254.53323	627.77025	418.84926	L	1881.08292	941.04510	627.69916	14
9	60.04439	1341.56525	671.28627	447.85994	S	1767.99886	884.50307	590.00447	13
10	72.08078	1440.63367	720.82047	480.88274	V	1680.96683	840.98705	560.99379	12
11	72.08078	1539.70208	770.35468	513.90554	V	1581.89841	791.45285	527.97099	11
12	86.09643	1652.78615	826.89671	551.60023	L	1482.83000	741.91864	494.94818	10
13	87.05529	1766.82907	883.91817	589.61454	N	1369.74594	685.37661	457.25350	9
14	101.07094	1894.88765	947.94746	632.30073	Q	1255.70301	628.35514	419.23919	8
15	86.09643	2007.97171	1004.48950	669.99542	L	1127.64443	564.32585	376.55299	7
16	133.04301	2168.00236	1084.50482	723.33897	C-Carbamidomethyl	1014.56037	507.78382	338.85831	6
17	72.08078	2267.07078	1134.03903	756.36178	V	854.52972	427.76850	285.51476	5
18	86.09643	2380.15484	1190.58106	794.05646	L	755.46131	378.23429	252.49195	4
19	110.07127	2517.21375	1259.11051	839.74277	H	642.37724	321.69226	214.79726	3
20	102.05496	2646.25635	1323.63181	882.75697	E	505.31833	253.16280	169.11096	2
21	330.27026				K-TMT6plex	376.27574	188.64151	126.09676	1

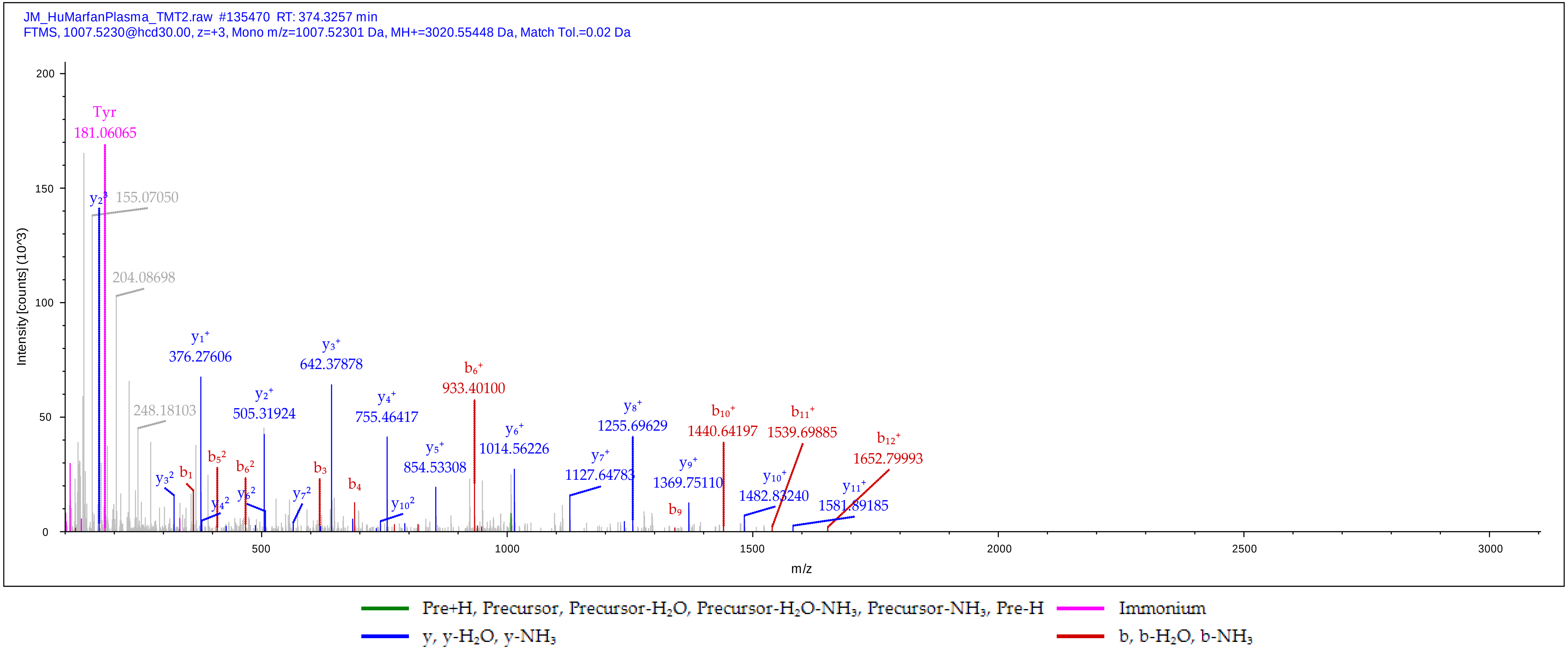
JM_HuMarfanPlasma_TMT2.raw #135470 RT: 374.3257 min
FTMS, 1007.5230@hcd30.00, z=+3, Mono m/z=1007.52301 Da, MH+=3020.55448 Da, Match Tol.=0.02 Da
Intensity [counts] (10^3)
m/z
Tyr
181.06065
155.07050
204.08698
248.18103
376.27606
505.31924
642.37878
755.46417
933.40100
854.53308
1014.56226
1127.64783
1255.69629
1369.75110
1440.64197
1539.69885
1482.83240
1581.89185
1652.79993
Pre+H, Precursor, Precursor-H2O, Precursor-H2O-NH3, Precursor-NH3, Pre-H
Immonium
y, y-H2O, y-NH3
b, b-H2O, b-NH3

#1	Immonium	b⁺	b²⁺	b³⁺	Seq.	y⁺	y²⁺	y³⁺	#2
1	358.27640	386.27132	193.63930	129.42862	R-TMT6plex				12
2	110.07127	523.33023	262.16875	175.11493	H	1356.72703	678.86715	452.91386	11
3	70.06513	620.38300	310.69514	207.46585	P	1219.66811	610.33769	407.22756	10
4	88.03930	735.40994	368.20861	245.80816	D	1122.61535	561.81131	374.87663	9
5	181.06077	943.45834	472.23281	315.15763	Y-Nitro	1007.58841	504.29784	336.53432	8
6	60.04439	1030.49037	515.74882	344.16831	S	799.54000	400.27364	267.18485	7
7	72.08078	1129.55879	565.28303	377.19111	V	712.50797	356.75762	238.17418	6
8	72.08078	1228.62720	614.81724	410.21392	V	613.43956	307.22342	205.15137	5
9	86.09643	1341.71126	671.35927	447.90861	L	514.37114	257.68921	172.12857	4
10	86.09643	1454.79533	727.90130	485.60329	L	401.28708	201.14718	134.43388	3
11	86.09643	1567.87939	784.44333	523.29798	L	288.20302	144.60515	96.73919	2
12	129.11347				R	175.11895	88.06311	59.04450	1

Nitro-Tyr immonium ion is detected in MS/MS spectra and added brown colored in the following spectrum

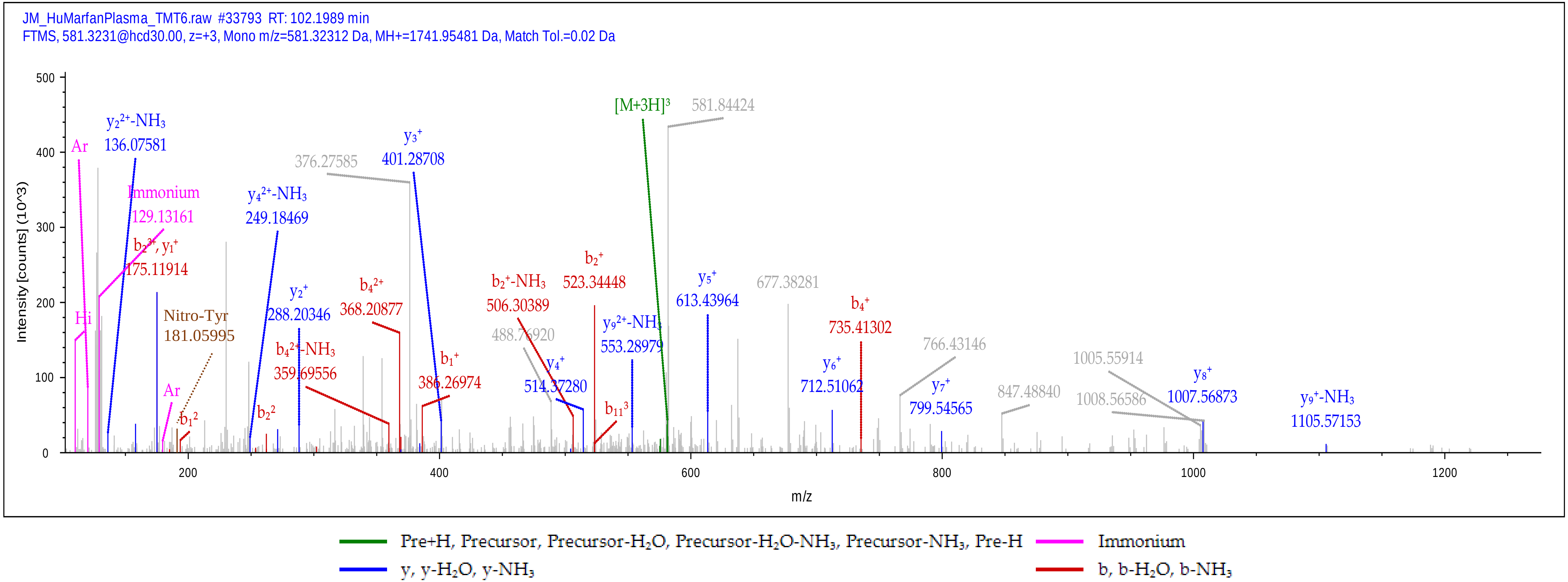
JM_HuMarfanPlasma_TMT6.raw #33793 RT: 102.1989 min
FTMS, 581.3231@hcd30.00, z=+3, Mono m/z=581.32312 Da, MH+=1741.95481 Da, Match Tol.=0.02 Da
Intensity [counts] (10^3)
m/z
Ar
Hi
Immonium 129.13161
Nitro-Tyr 181.05995
[M+3H]3 581.84424
376.27585
488.76920
677.38281
766.43146
847.48840
1005.55914
1008.56586
y2 2+-NH3 136.07581
y4 2+-NH3 249.18469
y2+ 288.20346
y3+ 401.28708
y4+ 514.37280
y9 2+-NH3 553.28979
y5+ 613.43964
y6+ 712.51062
y7+ 799.54565
y8+ 1007.56873
y9+-NH3 1105.57153
b2 3+, y1+ 175.11914
b4 2+ 368.20877
b4 2+-NH3 359.69556
b1+ 386.26974
b2+-NH3 506.30389
b2+ 523.34448
b4+ 735.41302
Pre+H, Precursor, Precursor-H2O, Precursor-H2O-NH3, Precursor-NH3, Pre-H
Immonium
y, y-H2O, y-NH3
b, b-H2O, b-NH3

#1	Immonium	b^+	b^{2+}	b^{3+}	Seq.	y^+	y^{2+}	y^{3+}	#2
1	330.23387	358.22879	179.61803	120.08111	Q-TMT6plex				13
2	87.05529	472.27171	236.63950	158.09542	N	1803.84208	902.42468	601.95221	12
3	133.04301	632.30236	316.65482	211.43897	C-Carbamidomethyl	1689.79915	845.40321	563.93790	11
4	102.05496	761.34495	381.17612	254.45317	E	1529.76850	765.38789	510.59435	10
5	86.09643	874.42902	437.71815	292.14786	L	1400.72591	700.86659	467.58015	9
6	120.08078	1021.49743	511.25235	341.17066	F	1287.64185	644.32456	429.88547	8
7	102.05496	1150.54003	575.77365	384.18486	E	1140.57343	570.79036	380.86266	7
8	101.07094	1278.59860	639.80294	426.87105	Q	1011.53084	506.26906	337.84846	6
9	86.09643	1391.68267	696.34497	464.56574	L	883.47226	442.23977	295.16227	5
10	30.03383	1448.70413	724.85570	483.57289	G	770.38820	385.69774	257.46758	4
11	102.05496	1577.74672	789.37700	526.58709	E	713.36674	357.18701	238.46043	3
12	181.06077	1785.79513	893.40120	595.93656	Y-Nitro	584.32414	292.66571	195.44623	2
13	330.27026				K-TMT6plex	376.27574	188.64151	126.09676	1

Nitro-Tyr immonium ion is detected in MS/MS spectra and added brown colored in the following spectrum

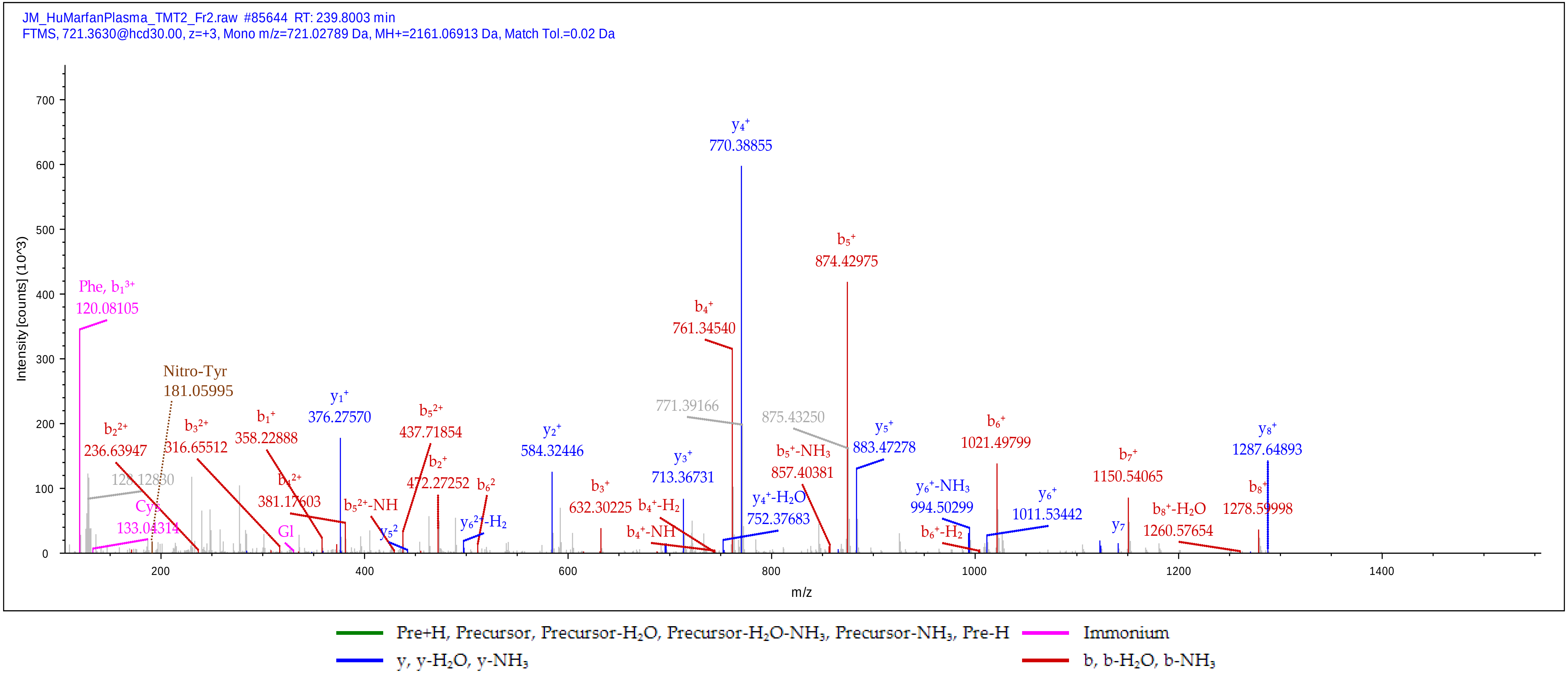

JM_HuMarfanPlasma_TMT2_Fr2.raw #85644 RT: 239.8003 min
FTMS, 721.3630@hcd30.00, z=+3, Mono m/z=721.02789 Da, MH+=2161.06913 Da, Match Tol.=0.02 Da
Intensity [counts] (10^3)
m/z
y4+ 770.38855
b5+ 874.42975
b4+ 761.34540
Phe, b13+ 120.08105
Nitro-Tyr 181.05995
y1+ 376.27570
b52+ 437.71854
b1+ 358.22888
b22+ 236.63947
b32+ 316.65512
y2+ 584.32446
771.39166
875.43250
y5+ 883.47278
b6+ 1021.49799
y8+ 1287.64893
b5+-NH3 857.40381
y3+ 713.36731
b7+ 1150.54065
b2+ 472.27252
b3+ 632.30225
b42+ 381.17603
128.12830
Cys 133.04314
y6+-NH3 994.50299
y6+ 1011.53442
b8+ 1278.59998
b8+-H2O 1260.57654
y4+-H2O 752.37683
b4+-H2
b4+-NH
b52+-NH
y62+-H2
b6+-H2
Pre+H, Precursor, Precursor-H2O, Precursor-H2O-NH3, Precursor-NH3, Pre-H
Immonium
y, y-H2O, y-NH3
b, b-H2O, b-NH3

#1	Immonium	b^+	b^{2+}	Seq.	y^+	y^{2+}	#2
1	317.20224	345.19715	173.10221	D-TMT6plex			13
2	181.06077	553.24556	277.12642	Y-Nitro	1559.79030	780.39879	12
3	72.08078	652.31397	326.66062	V	1351.74190	676.37459	11
4	60.04439	739.34600	370.17664	S	1252.67348	626.84038	10
5	101.07094	867.40458	434.20593	Q	1165.64145	583.32437	9
6	120.08078	1014.47299	507.74013	F	1037.58288	519.29508	8
7	102.05496	1143.51558	572.26143	E	890.51446	445.76087	7
8	30.03383	1200.53705	600.77216	G	761.47187	381.23957	6
9	60.04439	1287.56908	644.28818	S	704.45041	352.72884	5
10	44.04948	1358.60619	679.80673	A	617.41838	309.21283	4
11	86.09643	1471.69025	736.34877	L	546.38126	273.69427	3
12	30.03383	1528.71172	764.85950	G	433.29720	217.15224	2
13	330.27026			K-TMT6plex	376.27574	188.64151	1

Nitro-Tyr immonium ion is detected in MS/MS spectra and added brown colored in the following spectrum

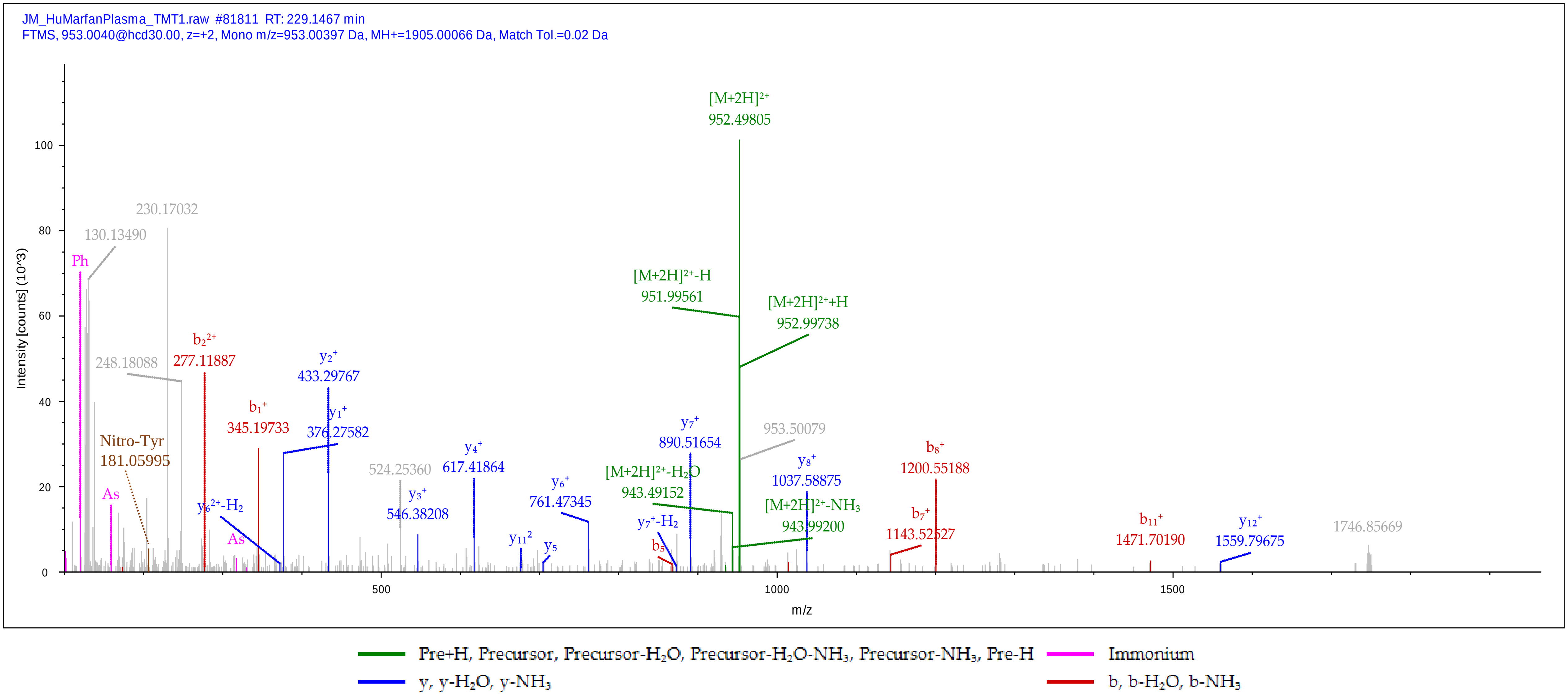
JM_HuMarfanPlasma_TMT1.raw #81811 RT: 229.1467 min
FTMS, 953.0040@hcd30.00, z=+2, Mono m/z=953.00397 Da, MH+=1905.00066 Da, Match Tol.=0.02 Da
Intensity [counts] (10^3)
m/z
[M+2H]2+ 952.49805
[M+2H]2+-H 951.99561
[M+2H]2+ +H 952.99738
230.17032
130.13490
Ph
248.18088
b2 2+ 277.11887
y2+ 433.29767
b1+ 345.19733
y1+ 376.27582
Nitro-Tyr 181.05995
As
y6 2+-H2
As
524.25360
y3+ 546.38208
y4+ 617.41864
y11 2
y5
y6+ 761.47345
[M+2H]2+-H2O 943.49152
y7+-H2
b5
y7+ 890.51654
953.50079
y8+ 1037.58875
[M+2H]2+-NH3 943.99200
b8+ 1200.55188
b7+ 1143.52527
b11+ 1471.70190
y12+ 1559.79675
1746.85669
Pre+H, Precursor, Precursor-H2O, Precursor-H2O-NH3, Precursor-NH3, Pre-H
Immonium
y, y-H2O, y-NH3
b, b-H2O, b-NH3

#1	Immonium	b^+	b^{2+}	Seq.	y^+	y^{2+}	#2
1	315.25936	343.25427	172.13077	L-TMT6plex			14
2	86.09643	456.33834	228.67281	L	1773.84927	887.42828	13
3	88.03930	571.36528	286.18628	D	1660.76521	830.88624	12
4	87.05529	685.40821	343.20774	N	1545.73827	773.37277	11
5	204.07675	916.47260	458.73994	W-Nitro	1431.69534	716.35131	10
6	88.03930	1031.49954	516.25341	D	1200.63095	600.81911	9
7	60.04439	1118.53157	559.76942	S	1085.60401	543.30564	8
8	72.08078	1217.59998	609.30363	V	998.57198	499.78963	7
9	74.06004	1318.64766	659.82747	T	899.50356	450.25542	6
10	60.04439	1405.67969	703.34348	S	798.45589	399.73158	5
11	74.06004	1506.72737	753.86732	T	711.42386	356.21557	4
12	120.08078	1653.79578	827.40153	F	610.37618	305.69173	3
13	60.04439	1740.82781	870.91754	S	463.30776	232.15752	2
14	330.27026			K-TMT6plex	376.27574	188.64151	1

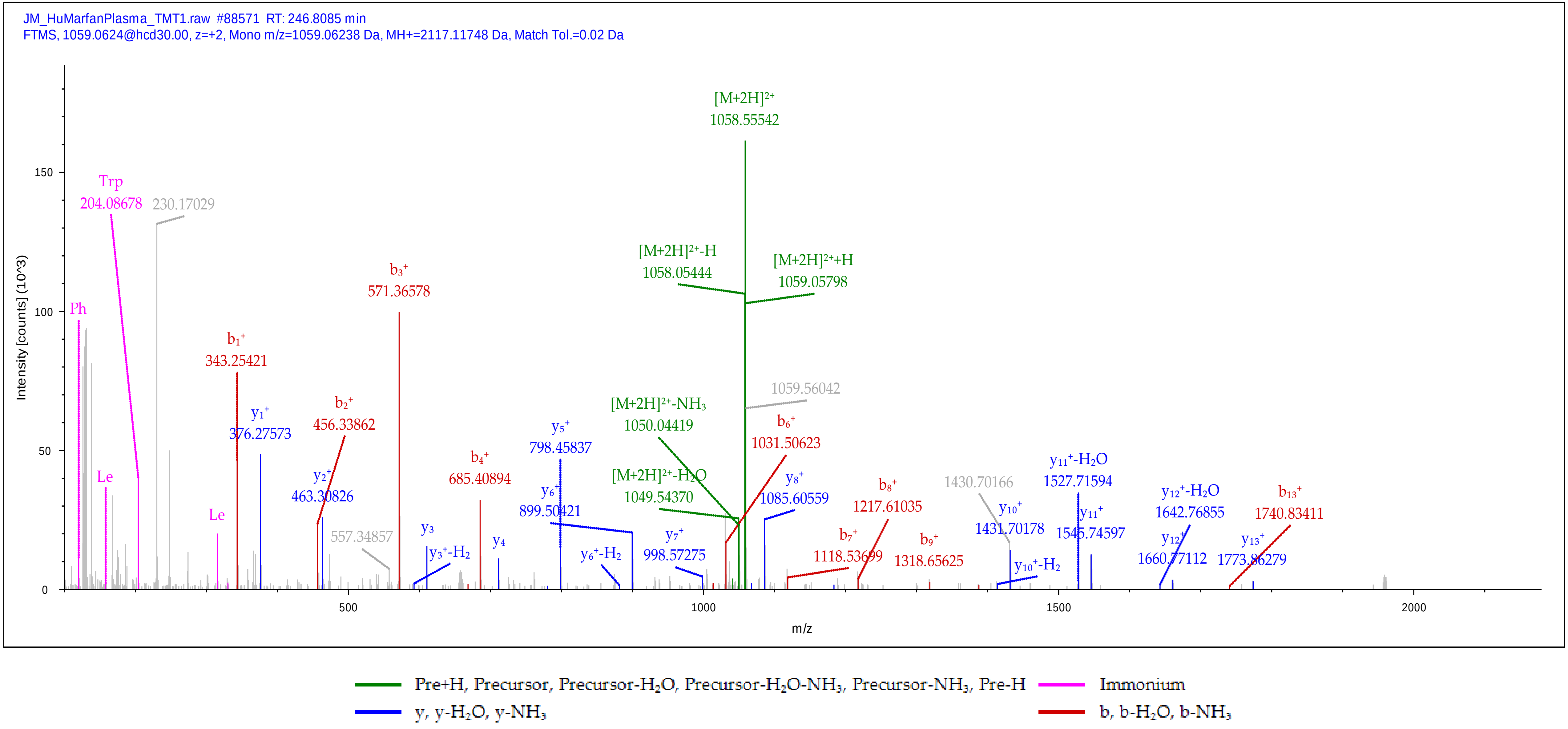

JM_HuMarfanPlasma_TMT1.raw #88571 RT: 246.8085 min
FTMS, 1059.0624@hcd30.00, z=+2, Mono m/z=1059.06238 Da, MH+=2117.11748 Da, Match Tol.=0.02 Da
Intensity [counts] (10^3)
m/z
[M+2H]2+ 1058.55542
Trp 204.08678
230.17029
Ph
Le
Le
b1+ 343.25421
y1+ 376.27573
b2+ 456.33862
y2+ 463.30826
557.34857
b3+ 571.36578
y3
y3+-H2
y4
b4+ 685.40894
y5+ 798.45837
y6+ 899.50421
y6+-H2
y7+ 998.57275
[M+2H]2+-H 1058.05444
[M+2H]2+-NH3 1050.04419
[M+2H]2+-H2O 1049.54370
[M+2H]2++H 1059.05798
1059.56042
b6+ 1031.50623
y8+ 1085.60559
b7+ 1118.53699
b8+ 1217.61035
b9+ 1318.65625
1430.70166
y10+ 1431.70178
y10+-H2
y11+-H2O 1527.71594
y11+ 1545.74597
y12+-H2O 1642.76855
y12+ 1660.77112
y13+ 1773.86279
b13+ 1740.83411
Pre+H, Precursor, Precursor-H2O, Precursor-H2O-NH3, Precursor-NH3, Pre-H
Immonium
y, y-H2O, y-NH3
b, b-H2O, b-NH3

#1	Immonium	b^+	b^{2+}	Seq.	y^+	y^{2+}	#2
1	331.21789	359.21280	180.11004	E-TMT6plex			16
2	101.07094	487.27138	244.13933	Q	2078.03920	1039.52324	15
3	86.09643	600.35544	300.68136	L	1949.98062	975.49395	14
4	30.03383	657.37691	329.19209	G	1836.89656	918.95192	13
5	70.06513	754.42967	377.71847	P	1779.87509	890.44119	12
6	72.08078	853.49808	427.25268	V	1682.82233	841.91480	11
7	74.06004	954.54576	477.77652	T	1583.75392	792.38060	10
8	101.07094	1082.60434	541.80581	Q	1482.70624	741.85676	9
9	102.05496	1211.64693	606.32710	E	1354.64766	677.82747	8
10	120.08078	1358.71535	679.86131	F	1225.60507	613.30617	7
11	204.07675	1589.77974	795.39351	W-Nitro	1078.53665	539.77197	6
12	88.03930	1704.80668	852.90698	D	847.47226	424.23977	5
13	87.05529	1818.84961	909.92844	N	732.44532	366.72630	4
14	86.09643	1931.93367	966.47047	L	618.40239	309.70483	3
15	102.05496	2060.97627	1030.99177	E	505.31833	253.16280	2
16	330.27026			K-TMT6plex	376.27574	188.64151	1

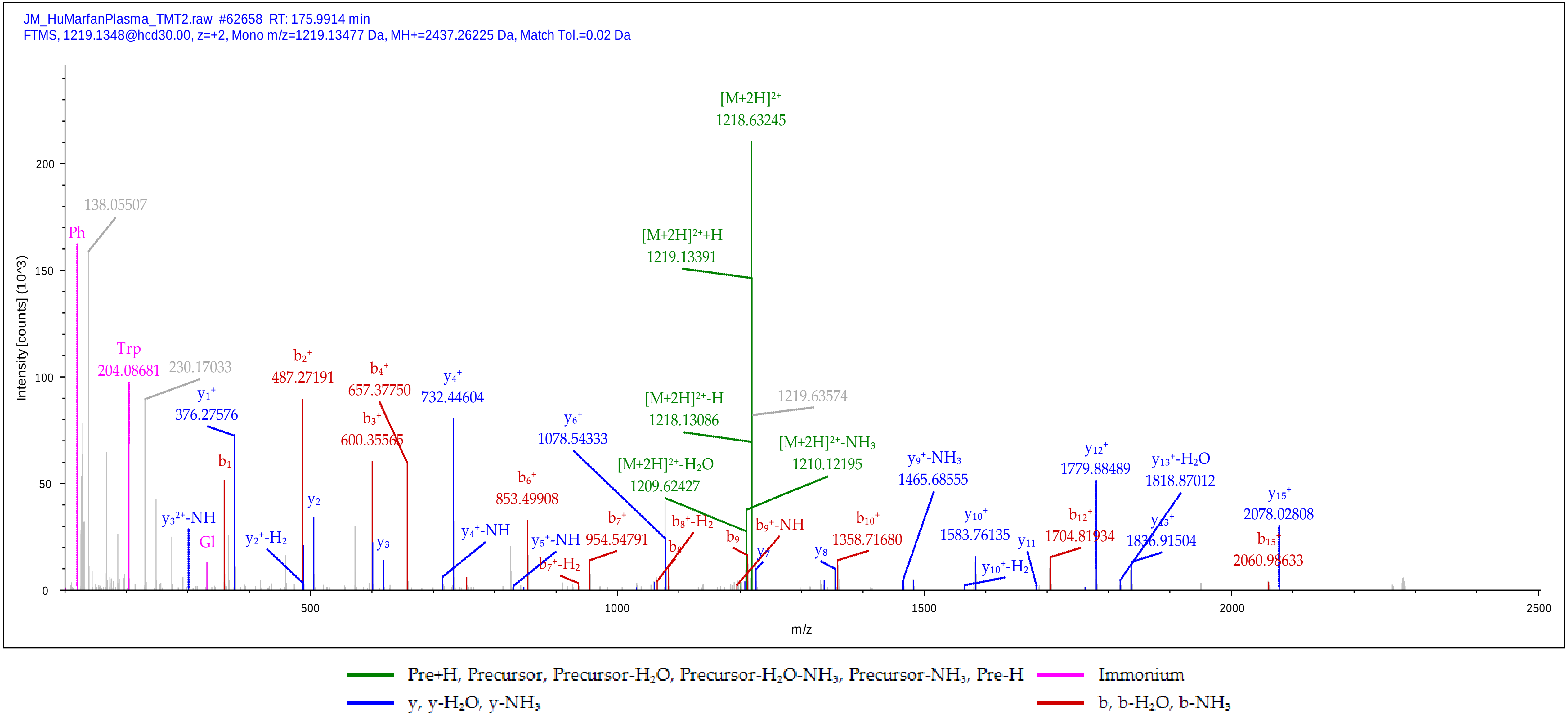

JM_HuMarfanPlasma_TMT2.raw #62658 RT: 175.9914 min
FTMS, 1219.1348@hcd30.00, z=+2, Mono m/z=1219.13477 Da, MH+=2437.26225 Da, Match Tol.=0.02 Da
Intensity [counts] (10^3)
m/z
$[M+2H]^{2+}$ 1218.63245
$[M+2H]^{2+}$+H 1219.13391
$[M+2H]^{2+}$-H 1218.13086
1219.63574
$[M+2H]^{2+}-NH_3$ 1210.12195
$[M+2H]^{2+}-H_2O$ 1209.62427
138.05507
Ph
Trp 204.08681
230.17033
y_1^+ 376.27576
b_2^+ 487.27191
b_4^+ 657.37750
b_3^+ 600.35565
y_4^+ 732.44604
y_6^+ 1078.54333
b_1
y_2
y_3^{2+}-NH
Gl
$y_2^+-H_2$
y_3
y_4^+-NH
b_6^+ 853.49908
y_5^+-NH
b_7^+ 954.54791
$b_7^+-H_2$
$b_8^+-H_2$
b_8
b_9
b_9^+-NH
y_7
y_8
b_{10}^+ 1358.71680
$y_9^+-NH_3$ 1465.68555
y_{10}^+ 1583.76135
$y_{10}^+-H_2$
y_{11}
b_{12}^+ 1704.81934
y_{12}^+ 1779.88489
$y_{13}^+-H_2O$ 1818.87012
y_{13}^+ 1836.91504
y_{15}^+ 2078.02808
b_{15}^+ 2060.98633
Pre+H, Precursor, Precursor-H_2O, Precursor-H_2O-NH_3, Precursor-NH_3, Pre-H
Immonium
y, y-H_2O, y-NH_3
b, b-H_2O, b-NH_3

#1	Immonium	b^{+}	b^{2+}	b^{3+}	Seq.	y^{+}	y^{2+}	y^{3+}	#2
1	301.24371	329.23862	165.12295	110.41773	V-TMT6plex				10
2	101.07094	457.29720	229.15224	153.10392	Q	1427.70043	714.35385	476.57166	9
3	70.06513	554.34996	277.67862	185.45484	P	1299.64185	650.32456	433.88547	8
4	181.06077	762.39837	381.70282	254.80431	Y-Nitro	1202.58908	601.79818	401.53455	7
5	86.09643	875.48243	438.24486	292.49900	L	994.54068	497.77398	332.18508	6
6	88.03930	990.50938	495.75833	330.84131	D	881.45661	441.23195	294.49039	5
7	88.03930	1105.53632	553.27180	369.18362	D	766.42967	383.71847	256.14807	4
8	120.08078	1252.60473	626.80601	418.20643	F	651.40273	326.20500	217.80576	3
9	101.07094	1380.66331	690.83529	460.89262	Q	504.33431	252.67080	168.78296	2
10	330.27026				K-TMT6plex	376.27574	188.64151	126.09676	1

Nitro-Tyr immonium ion is detected in MS/MS spectra and added brown colored in the following spectrum

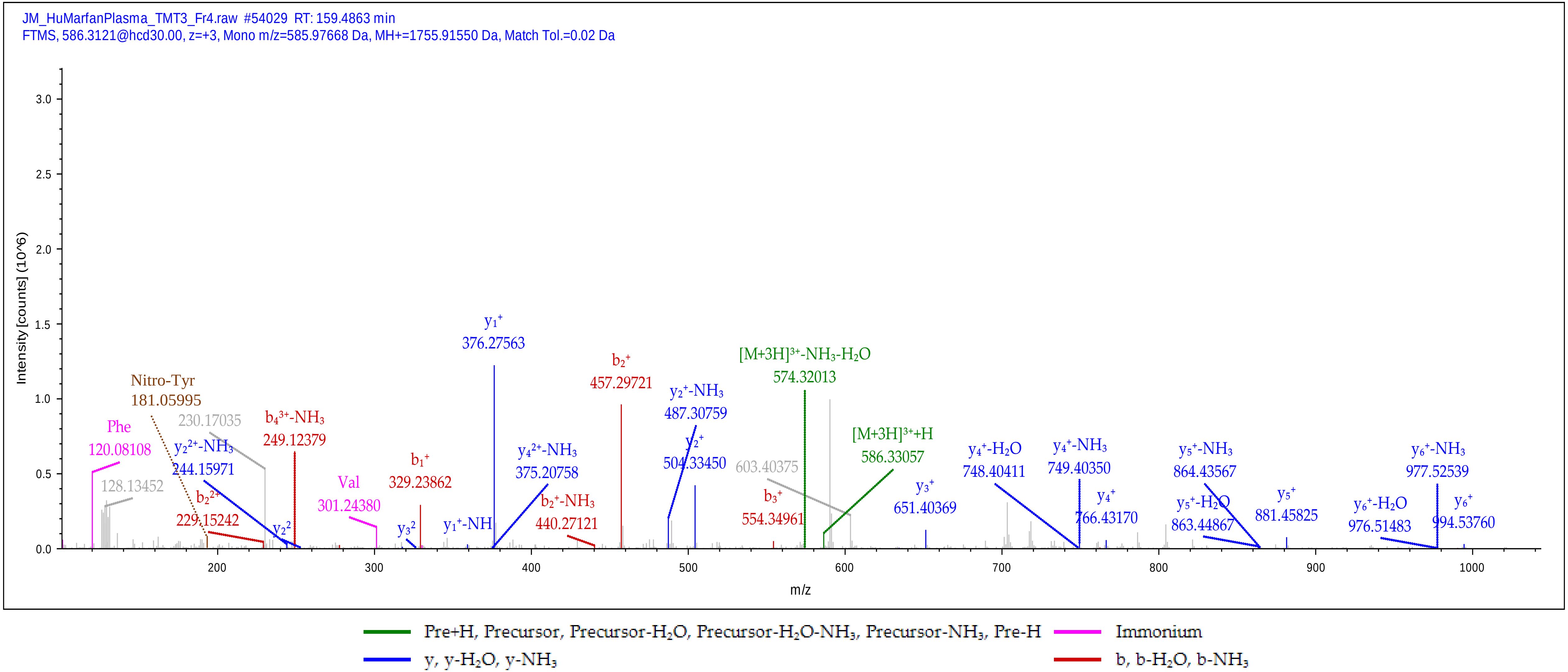
JM_HuMarfanPlasma_TMT3_Fr4.raw #54029 RT: 159.4863 min
FTMS, 586.3121@hcd30.00, z=+3, Mono m/z=585.97668 Da, MH+=1755.91550 Da, Match Tol.=0.02 Da
Intensity [counts] (10^6)
m/z
Nitro-Tyr 181.05995
Phe 120.08108
128.13452
230.17035
y_2^{2+}-NH_3 244.15971
b_2^{2+} 229.15242
b_4^{3+}-NH_3 249.12379
y_2^{2}
Val 301.24380
b_1^+ 329.23862
y_3^{2}
y_1^+-NH
y_1^+ 376.27563
y_4^{2+}-NH_3 375.20758
b_2^+-NH_3 440.27121
b_2^+ 457.29721
y_2^+-NH_3 487.30759
y_2^+ 504.33450
603.40375
b_3^+ 554.34961
$[M+3H]^{3+}$-NH_3-H_2O 574.32013
$[M+3H]^{3+}$+H 586.33057
y_3^+ 651.40369
y_4^+-H_2O 748.40411
y_4^+-NH_3 749.40350
y_4^+ 766.43170
y_5^+-NH_3 864.43567
y_5^+-H_2O 863.44867
y_5^+ 881.45825
y_6^+-H_2O 976.51483
y_6^+-NH_3 977.52539
y_6^+ 994.53760
Pre+H, Precursor, Precursor-H_2O, Precursor-H_2O-NH_3, Precursor-NH_3, Pre-H
Immonium
y, y-H_2O, y-NH_3
b, b-H_2O, b-NH_3

#1	Immonium	b^+	b^{2+}	b^{3+}	Seq.	y^+	y^{2+}	y^{3+}	#2
1	289.20732	317.20224	159.10476	106.40560	S-TMT6plex				23
2	30.03383	374.22370	187.61549	125.41275	G	2692.23983	1346.62355	898.08479	22
3	44.04948	445.26081	223.13405	149.09179	A	2635.21836	1318.11282	879.07764	21
4	30.03383	502.28228	251.64478	168.09894	G	2564.18125	1282.59426	855.39860	20
5	74.06004	603.32996	302.16862	201.78150	T	2507.15979	1254.08353	836.39145	19
6	102.05496	732.37255	366.68991	244.79570	E	2406.11211	1203.55969	802.70889	18
7	88.03930	847.39949	424.20338	283.13802	D	2277.06951	1139.03840	759.69469	17
8	60.04439	934.43152	467.71940	312.14869	S	2162.04257	1081.52492	721.35237	16
9	44.04948	1005.46863	503.23796	335.82773	A	2075.01054	1038.00891	692.34170	15
10	133.04301	1165.49928	583.25328	389.17128	C-Carbamidomethyl	2003.97343	1002.49035	668.66266	14
11	86.09643	1278.58335	639.79531	426.86597	I	1843.94278	922.47503	615.31911	13
12	70.06513	1375.63611	688.32169	459.21689	P	1730.85872	865.93300	577.62442	12
13	204.07675	1606.70050	803.85389	536.23835	W-Nitro	1633.80595	817.40661	545.27350	11
14	44.04948	1677.73762	839.37245	559.91739	A	1402.74156	701.87442	468.25204	10
15	136.07569	1840.80094	920.90411	614.27183	Y	1331.70445	666.35586	444.57300	9
16	136.07569	2003.86427	1002.43577	668.62628	Y	1168.64112	584.82420	390.21856	8
17	60.04439	2090.89630	1045.95179	697.63695	S	1005.57779	503.29253	335.86411	7
18	74.06004	2191.94398	1096.47563	731.31951	T	918.54576	459.77652	306.85344	6
19	72.08078	2291.01239	1146.00983	764.34232	V	817.49808	409.25268	273.17088	5
20	88.03930	2406.03934	1203.52331	802.68463	D	718.42967	359.71847	240.14807	4
21	101.07094	2534.09791	1267.55260	845.37082	Q	603.40273	302.20500	201.80576	3
22	72.08078	2633.16633	1317.08680	878.39363	V	475.34415	238.17571	159.11957	2
23	330.27026				K-TMT6plex	376.27574	188.64151	126.09676	1

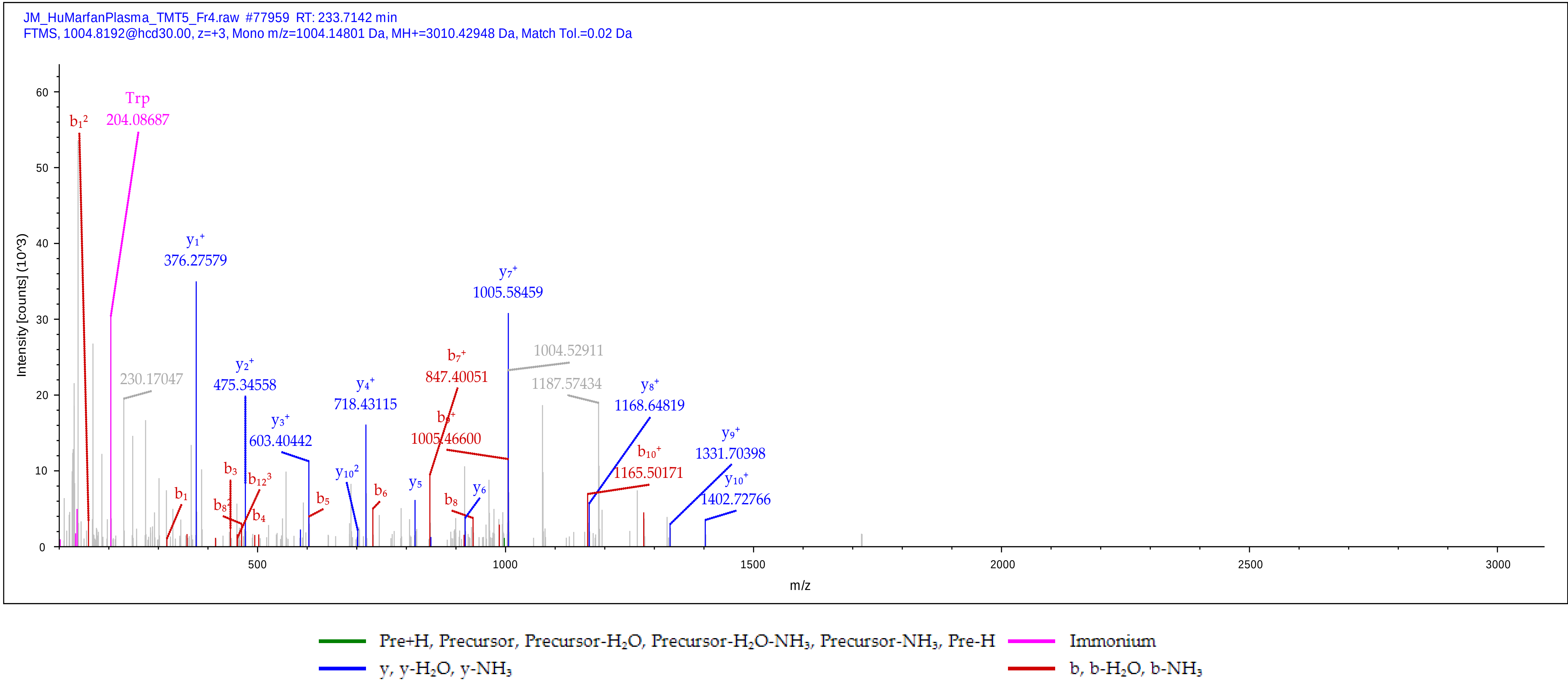

JM_HuMarfanPlasma_TMT5_Fr4.raw #77959 RT: 233.7142 min
FTMS, 1004.8192@hcd30.00, z=+3, Mono m/z=1004.14801 Da, MH+=3010.42948 Da, Match Tol.=0.02 Da
Intensity [counts] (10^3)
m/z
Trp
204.08687
b_1^2
y_1^+
376.27579
230.17047
y_2^+
475.34558
y_3^+
603.40442
y_4^+
718.43115
b_7^+
847.40051
b_8^+
1005.46600
y_7^+
1005.58459
1004.52911
1187.57434
y_8^+
1168.64819
b_{10}^+
1165.50171
y_9^+
1331.70398
y_{10}^+
1402.72766
b_1
b_3
b_8^2
b_{12}^3
b_4
b_5
y_{10}^2
b_6
y_5
b_8
y_6
Pre+H, Precursor, Precursor-H_2O, Precursor-H_2O-NH_3, Precursor-NH_3, Pre-H
Immonium
y, y-H_2O, y-NH_3
b, b-H_2O, b-NH_3

#1	Immonium	b^+	b^{2+}	Seq.	y^+	y^{2+}	#2
1	349.24371	377.23862	189.12295	F-TMT6plex			6
2	181.06077	585.28703	293.14715	Y-Nitro	1153.73452	577.37090	5
3	330.27026	942.54492	471.77610	K-TMT6plex	945.68611	473.34669	4
4	86.09643	1055.62899	528.31813	L	588.42821	294.71775	3
5	72.08078	1154.69740	577.85234	V	475.34415	238.17571	2
6	330.27026			K-TMT6plex	376.27574	188.64151	1

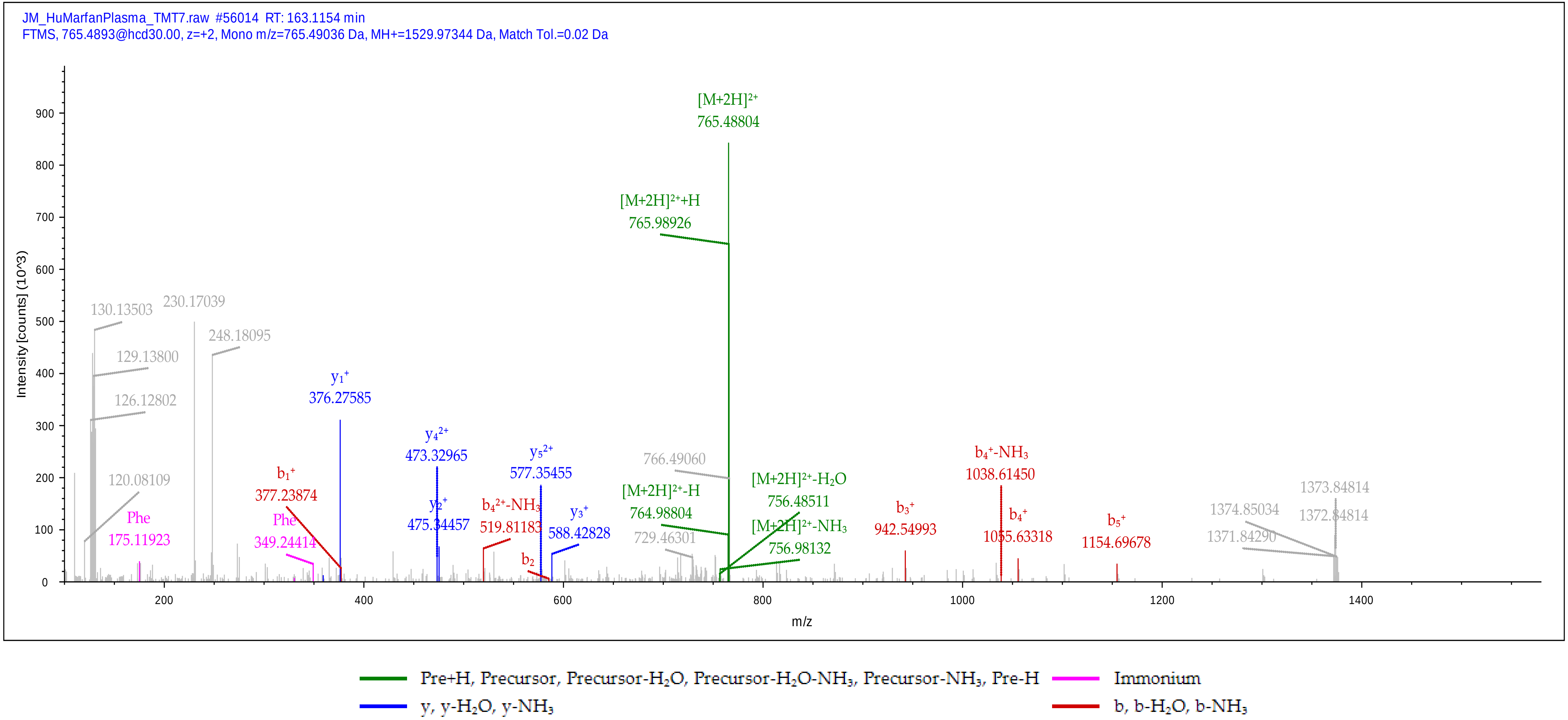

JM_HuMarfanPlasma_TMT7.raw #56014 RT: 163.1154 min
FTMS, 765.4893@hcd30.00, z=+2, Mono m/z=765.49036 Da, MH+=1529.97344 Da, Match Tol.=0.02 Da
Intensity [counts] (10^3)
m/z
[M+2H]2+ 765.48804
[M+2H]2++H 765.98926
130.13503
230.17039
248.18095
129.13800
126.12802
120.08109
Phe 175.11923
y1+ 376.27585
b1+ 377.23874
Phe 349.24414
y42+ 473.32965
y2+ 475.34457
b42+-NH3 519.81183
b2
y52+ 577.35455
y3+ 588.42828
766.49060
[M+2H]2+-H 764.98804
729.46301
[M+2H]2+-H2O 756.48511
[M+2H]2+-NH3 756.98132
b3+ 942.54993
b4+-NH3 1038.61450
b4+ 1055.63318
b5+ 1154.69678
1374.85034
1371.84290
1373.84814
1372.84814
Pre+H, Precursor, Precursor-H2O, Precursor-H2O-NH3, Precursor-NH3, Pre-H
Immonium
y, y-H2O, y-NH3
b, b-H2O, b-NH3

#1	Immonium	b^{+}	b^{2+}	b^{3+}	Seq.	y^{+}	y^{2+}	y^{3+}	#2
1	273.21241	301.20732	151.10730	101.07396	A-TMT6plex				23
2	87.05529	415.25025	208.12876	139.08827	N	2864.35111	1432.67919	955.45522	22
3	101.07094	543.30883	272.15805	181.77446	Q	2750.30818	1375.65773	917.44091	21
4	101.07094	671.36740	336.18734	224.46065	Q	2622.24960	1311.62844	874.75472	20
5	120.08078	818.43582	409.72155	273.48346	F	2494.19103	1247.59915	832.06853	19
6	86.09643	931.51988	466.26358	311.17815	L	2347.12261	1174.06494	783.04572	18
7	72.08078	1030.58830	515.79779	344.20095	V	2234.03855	1117.52291	745.35103	17
8	136.07569	1193.65162	597.32945	398.55539	Y	2134.97013	1067.98871	712.32823	16
9	133.04301	1353.68227	677.34477	451.89894	C-Carbamidomethyl	1971.90681	986.45704	657.97379	15
10	102.05496	1482.72487	741.86607	494.91314	E	1811.87616	906.44172	604.63024	14
11	86.09643	1595.80893	798.40810	532.60783	I	1682.83356	841.92042	561.61604	13
12	88.03930	1710.83587	855.92157	570.95014	D	1569.74950	785.37839	523.92135	12
13	30.03383	1767.85734	884.43231	589.95730	G	1454.72256	727.86492	485.57904	11
14	60.04439	1854.88937	927.94832	618.96797	S	1397.70109	699.35419	466.57188	10
15	30.03383	1911.91083	956.45905	637.97513	G	1310.66907	655.83817	437.56121	9
16	87.05529	2025.95376	1013.48052	675.98944	N	1253.64760	627.32744	418.55405	8
17	30.03383	2082.97522	1041.99125	694.99659	G	1139.60467	570.30598	380.53974	7
18	204.07675	2314.03961	1157.52344	772.01805	W-Nitro	1082.58321	541.79524	361.53259	6
19	74.06004	2415.08729	1208.04728	805.70061	T	851.51882	426.26305	284.51112	5
20	72.08078	2514.15570	1257.58149	838.72342	V	750.47114	375.73921	250.82856	4
21	120.08078	2661.22412	1331.11570	887.74622	F	651.40273	326.20500	217.80576	3
22	101.07094	2789.28269	1395.14499	930.43242	Q	504.33431	252.67080	168.78296	2
23	330.27026				K-TMT6plex	376.27574	188.64151	126.09676	1

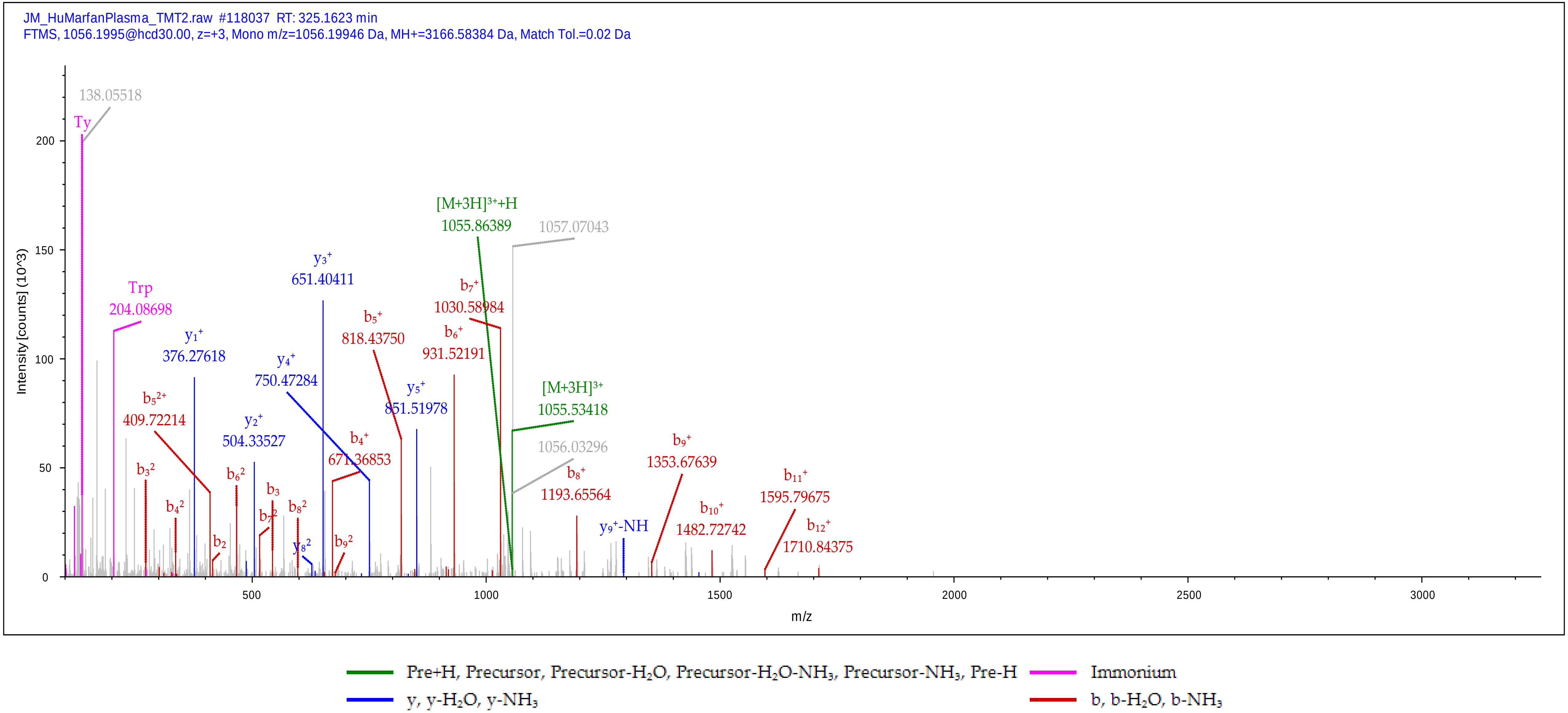
JM_HuMarfanPlasma_TMT2.raw #118037 RT: 325.1623 min
FTMS, 1056.1995@hcd30.00, z=+3, Mono m/z=1056.19946 Da, MH+=3166.58384 Da, Match Tol.=0.02 Da
138.05518
Ty
Trp
204.08698
y1+
376.27618
b5 2+
409.72214
b3 2
b4 2
b2
y2+
504.33527
b6 2
b3
b8 2
y8 2
y3+
651.40411
y4+
750.47284
b4+
671.36853
b9 2
b5+
818.43750
y5+
851.51978
b6+
931.52191
b7+
1030.58984
[M+3H]3++H
1055.86389
1057.07043
[M+3H]3+
1055.53418
1056.03296
b8+
1193.65564
y9+-NH
b9+
1353.67639
b10+
1482.72742
b11+
1595.79675
b12+
1710.84375
Intensity [counts] (10^3)
m/z
Pre+H, Precursor, Precursor-H2O, Precursor-H2O-NH3, Precursor-NH3, Pre-H
Immonium
y, y-H2O, y-NH3
b, b-H2O, b-NH3

#1	Immonium	b⁺	b^{2+}	b^{3+}	b^{4+}	Seq.	y⁺	y^{2+}	y^{3+}	y^{4+}	#2
1	273.21241	301.20732	151.10730	101.07396	76.05729	A-TMT6plex					23
2	87.05529	415.25025	208.12876	139.08827	104.56802	N	2864.35111	1432.67919	955.45522	716.84323	22
3	101.07094	543.30883	272.15805	181.77446	136.58266	Q	2750.30818	1375.65773	917.44091	688.33250	21
4	101.07094	671.36740	336.18734	224.46065	168.59731	Q	2622.24960	1311.62844	874.75472	656.31786	20
5	120.08078	818.43582	409.72155	273.48346	205.36441	F	2494.19103	1247.59915	832.06853	624.30321	19
6	86.09643	931.51988	466.26358	311.17815	233.63543	L	2347.12261	1174.06494	783.04572	587.53611	18
7	72.08078	1030.58830	515.79779	344.20095	258.40253	V	2234.03855	1117.52291	745.35103	559.26509	17
8	181.06077	1238.63670	619.82199	413.55042	310.41463	Y-Nitro	2134.97013	1067.98871	712.32823	534.49799	16
9	133.04301	1398.66735	699.83731	466.89397	350.42230	C-Carbamidomethyl	1926.92173	963.96450	642.97876	482.48589	15
10	102.05496	1527.70994	764.35861	509.90817	382.68294	E	1766.89108	883.94918	589.63521	442.47823	14
11	86.09643	1640.79401	820.90064	547.60285	410.95396	I	1637.84849	819.42788	546.62101	410.21758	13
12	88.03930	1755.82095	878.41411	585.94517	439.71070	D	1524.76442	762.88585	508.92633	381.94656	12
13	30.03383	1812.84241	906.92485	604.95232	453.96606	G	1409.73748	705.37238	470.58401	353.18983	11
14	60.04439	1899.87444	950.44086	633.96300	475.72407	S	1352.71602	676.86165	451.57686	338.93446	10
15	30.03383	1956.89591	978.95159	652.97015	489.97943	G	1265.68399	633.34563	422.56618	317.17645	9
16	87.05529	2070.93883	1035.97306	690.98446	518.49017	N	1208.66252	604.83490	403.55903	302.92109	8
17	30.03383	2127.96030	1064.48379	709.99162	532.74553	G	1094.61960	547.81344	365.54472	274.41036	7
18	159.09167	2314.03961	1157.52344	772.01805	579.26536	W	1037.59813	519.30270	346.53756	260.15499	6
19	74.06004	2415.08729	1208.04728	805.70061	604.52728	T	851.51882	426.26305	284.51112	213.63516	5
20	72.08078	2514.15570	1257.58149	838.72342	629.29438	V	750.47114	375.73921	250.82856	188.37324	4
21	120.08078	2661.22412	1331.11570	887.74622	666.06149	F	651.40273	326.20500	217.80576	163.60614	3
22	101.07094	2789.28269	1395.14499	930.43242	698.07613	Q	504.33431	252.67080	168.78296	126.83904	2
23	330.27026					K-TMT6plex	376.27574	188.64151	126.09676	94.82439	1

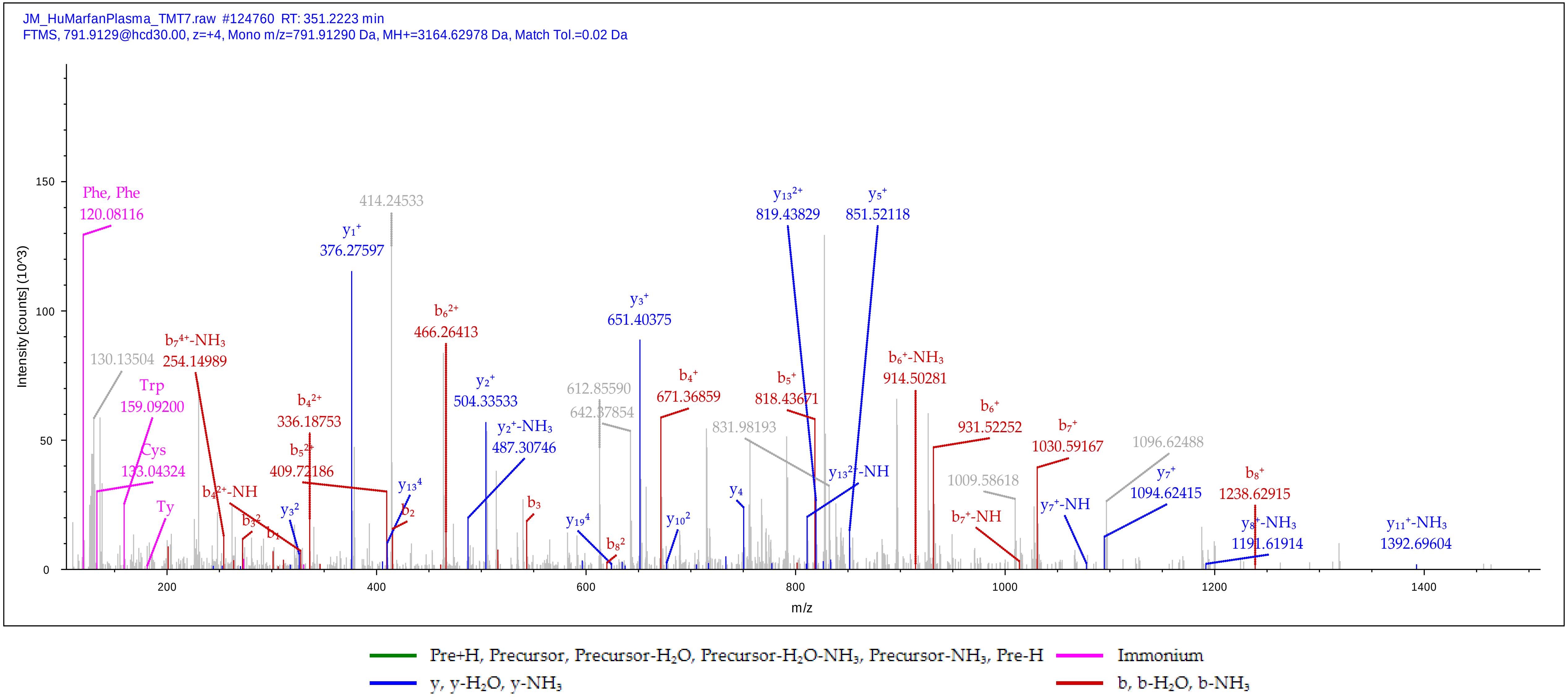
JM_HuMarfanPlasma_TMT7.raw #124760 RT: 351.2223 min
FTMS, 791.9129@hcd30.00, z=+4, Mono m/z=791.91290 Da, MH+=3164.62978 Da, Match Tol.=0.02 Da
Intensity [counts] (10^3)
m/z
Phe, Phe
120.08116
130.13504
Trp
159.09200
Cys
133.04324
Ty
b7 4+-NH3
254.14989
b4 2+-NH
b3 2
b1
b4 2+
336.18753
b5 2+
409.72186
y3 2
y1+
376.27597
414.24533
y13 4
b2
b6 2+
466.26413
y2+
504.33533
y2+-NH3
487.30746
b3
612.85590
642.37854
y19 4
b8 2
y3+
651.40375
b4+
671.36859
y10 2
831.98193
y4
b5+
818.43671
y13 2+
819.43829
y13 2+-NH
y5+
851.52118
b6+-NH3
914.50281
b6+
931.52252
1009.58618
b7+-NH
b7+
1030.59167
y7+-NH
1096.62488
y7+
1094.62415
b8+
1238.62915
y8+-NH3
1191.61914
y11+-NH3
1392.69604
Pre+H, Precursor, Precursor-H2O, Precursor-H2O-NH3, Precursor-NH3, Pre-H
Immonium
y, y-H2O, y-NH3
b, b-H2O, b-NH3

#1	Immonium	b^+	b^{2+}	Seq.	y^+	y^{2+}	#2
1	301.24371	329.23862	165.12295	V-TMT6plex			10
2	74.06004	430.28630	215.64679	T	1378.71641	689.86184	9
3	60.04439	517.31833	259.16280	S	1277.66873	639.33800	8
4	86.09643	630.40239	315.70483	I	1190.63670	595.82199	7
5	101.07094	758.46097	379.73412	Q	1077.55264	539.27996	6
6	88.03930	873.48791	437.24760	D	949.49406	475.25067	5
7	204.07675	1104.55230	552.77979	W-Nitro	834.46712	417.73720	4
8	72.08078	1203.62072	602.31400	V	603.40273	302.20500	3
9	101.07094	1331.67930	666.34329	Q	504.33431	252.67080	2
10	330.27026			K-TMT6plex	376.27574	188.64151	1

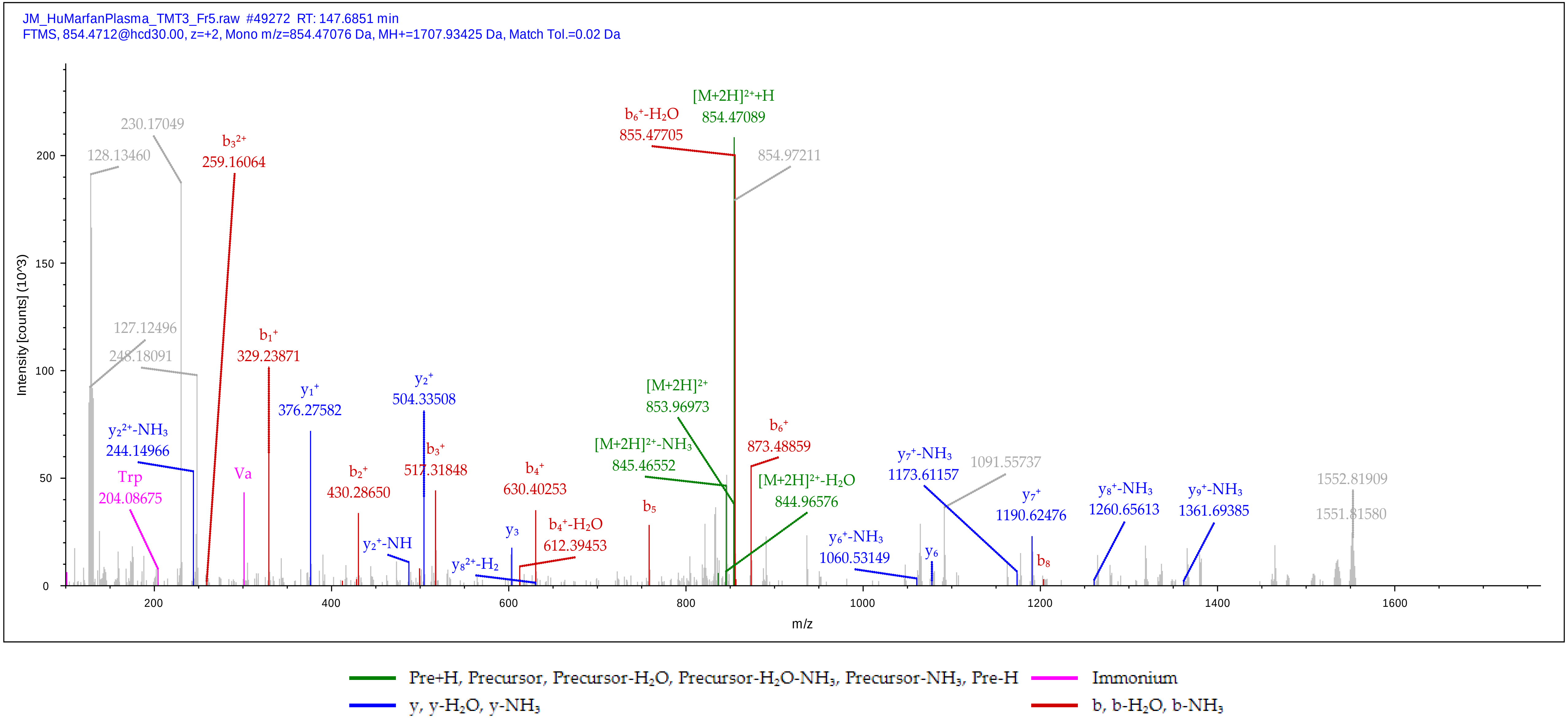
JM_HuMarfanPlasma_TMT3_Fr5.raw #49272 RT: 147.6851 min
FTMS, 854.4712@hcd30.00, z=+2, Mono m/z=854.47076 Da, MH+=1707.93425 Da, Match Tol.=0.02 Da
Intensity [counts] (10^3)
m/z
230.17049
128.13460
b3 2+ 259.16064
b6+-H2O 855.47705
[M+2H]2++H 854.47089
854.97211
127.12496
248.18091
b1+ 329.23871
y1+ 376.27582
y2+ 504.33508
[M+2H]2+ 853.96973
y2 2+-NH3 244.14966
Trp 204.08675
Va
b2+ 430.28650
b3+ 517.31848
b4+ 630.40253
[M+2H]2+-NH3 845.46552
b6+ 873.48859
y7+-NH3 1173.61157
1091.55737
[M+2H]2+-H2O 844.96576
y7+ 1190.62476
y8+-NH3 1260.65613
y9+-NH3 1361.69385
1552.81909
1551.81580
b5
y2+-NH
y3
b4+-H2O 612.39453
y8 2+-H2
y6+-NH3 1060.53149
y6
b8
Pre+H, Precursor, Precursor-H2O, Precursor-H2O-NH3, Precursor-NH3, Pre-H
Immonium
y, y-H2O, y-NH3
b, b-H2O, b-NH3

#1	Immonium	b⁺	b^{2+}	b^{3+}	Seq.	y^{+}	y^{2+}	y^{3+}	#2
1	301.24371	329.23862	165.12295	110.41773	V-TMT6plex				16
2	72.08078	428.30704	214.65716	143.44053	V	1983.08609	992.04668	661.70021	15
3	60.04439	515.33906	258.17317	172.45121	S	1884.01768	942.51248	628.67741	14
4	72.08078	614.40748	307.70738	205.47401	V	1796.98565	898.99646	599.66673	13
5	86.09643	727.49154	364.24941	243.16870	L	1697.91723	849.46226	566.64393	12
6	74.06004	828.53922	414.77325	276.85126	T	1584.83317	792.92022	528.94924	11
7	72.08078	927.60763	464.30746	309.87406	V	1483.78549	742.39638	495.26668	10
8	86.09643	1040.69170	520.84949	347.56875	L	1384.71708	692.86218	462.24388	9
9	110.07127	1177.75061	589.37894	393.25505	H	1271.63301	636.32015	424.54919	8
10	101.07094	1305.80919	653.40823	435.94125	Q	1134.57410	567.79069	378.86289	7
11	88.03930	1420.83613	710.92170	474.28356	D	1006.51553	503.76140	336.17669	6
12	204.07675	1651.90052	826.45390	551.30503	W-Nitro	891.48858	446.24793	297.83438	5
13	86.09643	1764.98459	882.99593	588.99971	L	660.42419	330.71573	220.81291	4
14	87.05529	1879.02751	940.01739	627.01402	N	547.34013	274.17370	183.11823	3
15	30.03383	1936.04898	968.52813	646.02118	G	433.29720	217.15224	145.10392	2
16	330.27026				K-TMT6plex	376.27574	188.64151	126.09676	1

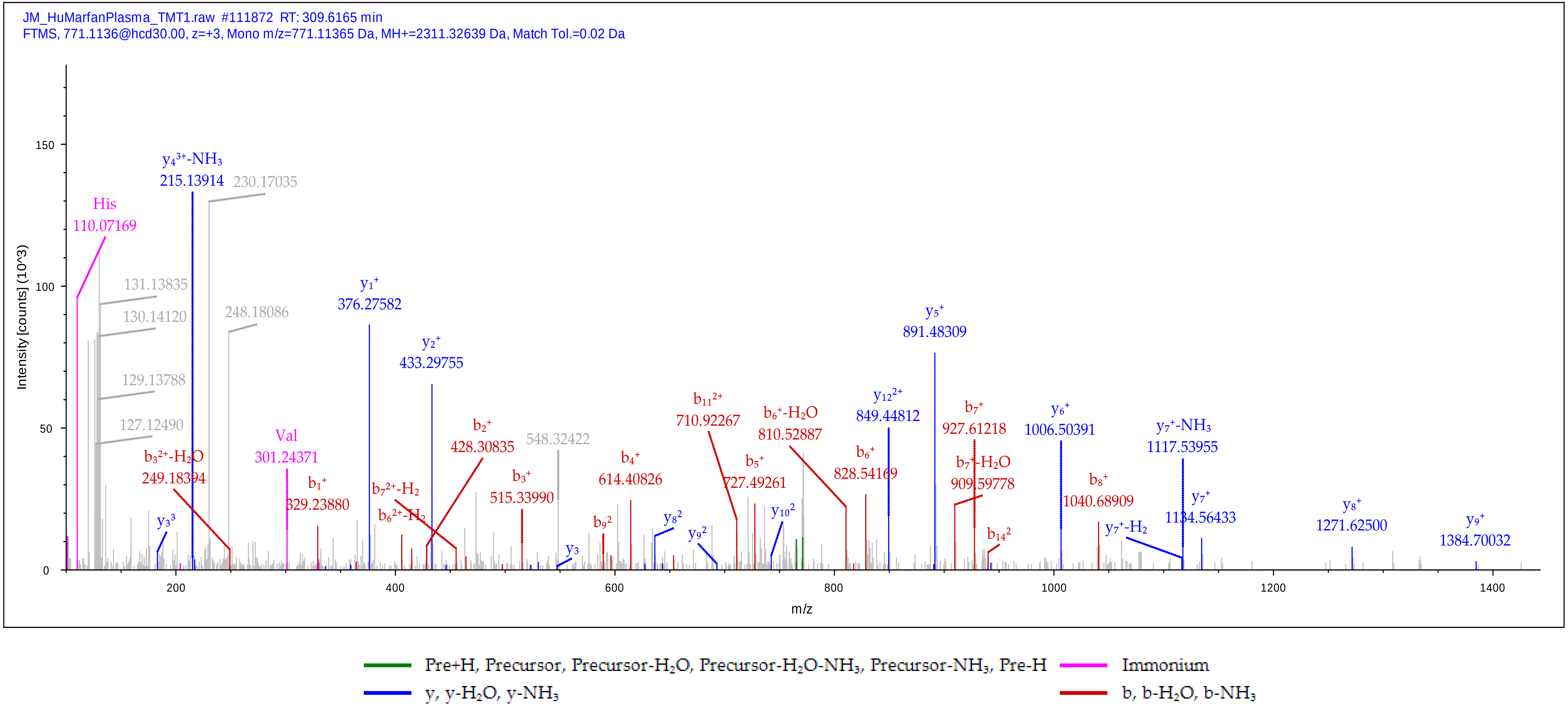

JM_HuMarfanPlasma_TMT1.raw #111872 RT: 309.6165 min
FTMS, 771.1136@hcd30.00, z=+3, Mono m/z=771.11365 Da, MH+=2311.32639 Da, Match Tol.=0.02 Da
Intensity [counts] (10^3)
m/z
His 110.07169
y4 3+-NH3 215.13914
230.17035
131.13835
130.14120
248.18086
129.13788
127.12490
b3 2+-H2O 249.18394
Val 301.24371
b1+ 329.23880
y1+ 376.27582
y2+ 433.29755
b2+ 428.30835
b7 2+-H2
b6 2+-H2
b3+ 515.33990
548.32422
b4+ 614.40826
b11 2+ 710.92267
b5+ 727.49261
b6+-H2O 810.52887
b6+ 828.54169
y12 2+ 849.44812
y5+ 891.48309
b7+ 927.61218
b7+-H2O 909.59778
y6+ 1006.50391
b8+ 1040.68909
y7+-NH3 1117.53955
y7+ 1134.56433
y8+ 1271.62500
y9+ 1384.70032
Pre+H, Precursor, Precursor-H2O, Precursor-H2O-NH3, Precursor-NH3, Pre-H
Immonium
y, y-H2O, y-NH3
b, b-H2O, b-NH3

#1	Immonium	b^+	b^{2+}	b^{3+}	Seq.	y^+	y^{2+}	y^{3+}	#2
1	259.19676	287.19167	144.09947	96.40208	G-TMT6plex				22
2	120.08078	434.26009	217.63368	145.42488	F	2761.25792	1381.13260	921.09082	21
3	136.07569	597.32341	299.16535	199.77932	Y	2614.18951	1307.59839	872.06802	20
4	70.06513	694.37618	347.69173	232.13024	P	2451.12618	1226.06673	817.71358	19
5	60.04439	781.40821	391.20774	261.14092	S	2354.07341	1177.54034	785.36266	18
6	88.03930	896.43515	448.72121	299.48323	D	2267.04138	1134.02433	756.35198	17
7	86.09643	1009.51921	505.26325	337.17792	I	2152.01444	1076.51086	718.00966	16
8	44.04948	1080.55633	540.78180	360.85696	A	2038.93038	1019.96883	680.31498	15
9	72.08078	1179.62474	590.31601	393.87976	V	1967.89326	984.45027	656.63594	14
10	102.05496	1308.66733	654.83731	436.89396	E	1868.82485	934.91606	623.61313	13
11	204.07675	1539.73173	770.36950	513.91543	W-Nitro	1739.78226	870.39477	580.59894	12
12	102.05496	1668.77432	834.89080	556.92962	E	1508.71787	754.86257	503.57747	11
13	60.04439	1755.80635	878.40681	585.94030	S	1379.67527	690.34127	460.56328	10
14	87.05529	1869.84927	935.42828	623.95461	N	1292.64324	646.82526	431.55260	9
15	30.03383	1926.87074	963.93901	642.96176	G	1178.60032	589.80380	393.53829	8
16	101.07094	2054.92932	1027.96830	685.64796	Q	1121.57885	561.29307	374.53114	7
17	70.06513	2151.98208	1076.49468	717.99888	P	993.52028	497.26378	331.84494	6
18	102.05496	2281.02467	1141.01597	761.01308	E	896.46751	448.73739	299.49402	5
19	87.05529	2395.06760	1198.03744	799.02738	N	767.42492	384.21610	256.47982	4
20	87.05529	2509.11053	1255.05890	837.04169	N	653.38199	327.19463	218.46552	3
21	136.07569	2672.17386	1336.59057	891.39614	Y	539.33906	270.17317	180.45121	2
22	330.27026				K-TMT6plex	376.27574	188.64151	126.09676	1

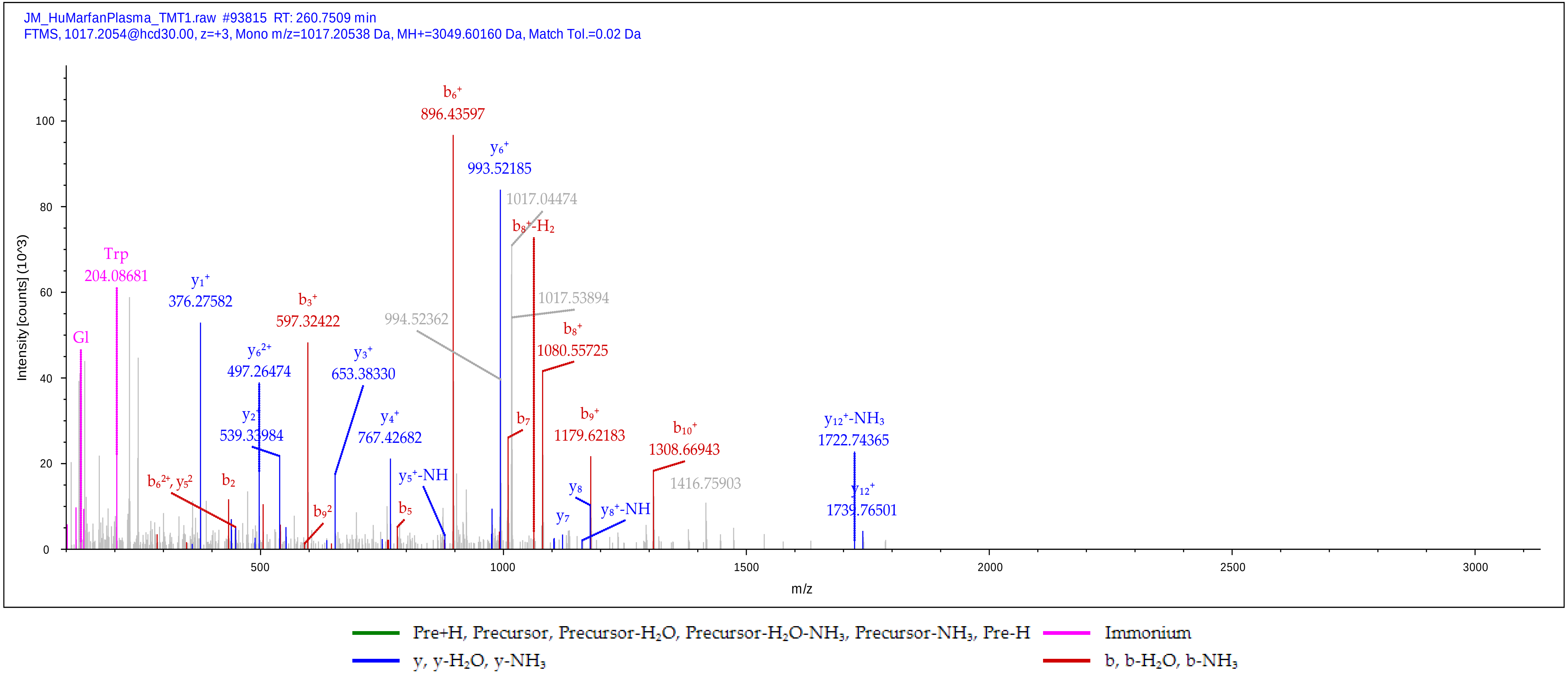
JM_HuMarfanPlasma_TMT1.raw #93815 RT: 260.7509 min
FTMS, 1017.2054@hcd30.00, z=+3, Mono m/z=1017.20538 Da, MH+=3049.60160 Da, Match Tol.=0.02 Da
Intensity [counts] (10^3)
m/z
Gl
Trp
204.08681
y1+
376.27582
y62+
497.26474
y2+
539.33984
b62+, y52
b2
b3+
597.32422
b92
y3+
653.38330
y4+
767.42682
y5+-NH
b5
b6+
896.43597
994.52362
y6+
993.52185
1017.04474
b8+-H2
1017.53894
b8+
1080.55725
b7
y7
y8
y8+-NH
b9+
1179.62183
b10+
1308.66943
1416.75903
y12+-NH3
1722.74365
y12+
1739.76501
Pre+H, Precursor, Precursor-H2O, Precursor-H2O-NH3, Precursor-NH3, Pre-H
Immonium
y, y-H2O, y-NH3
b, b-H2O, b-NH3

#1	Immonium	b^{+}	b^{2+}	b^{3+}	Seq.	y^{+}	y^{2+}	y^{3+}	#2
1	303.22297	331.21789	166.11258	111.07748	T-TMT6plex				17
2	74.06004	432.26557	216.63642	144.76004	T	2047.02215	1024.01471	683.01224	16
3	70.06513	529.31833	265.16280	177.11096	P	1945.97447	973.49088	649.32968	15
4	70.06513	626.37109	313.68918	209.46188	P	1848.92171	924.96449	616.97875	14
5	72.08078	725.43951	363.22339	242.48469	V	1751.86895	876.43811	584.62783	13
6	86.09643	838.52357	419.76542	280.17937	L	1652.80053	826.90390	551.60503	12
7	88.03930	953.55051	477.27890	318.52169	D	1539.71647	770.36187	513.91034	11
8	60.04439	1040.58254	520.79491	347.53237	S	1424.68953	712.84840	475.56803	10
9	88.03930	1155.60949	578.30838	385.87468	D	1337.65750	669.33239	446.55735	9
10	30.03383	1212.63095	606.81911	404.88183	G	1222.63055	611.81892	408.21504	8
11	60.04439	1299.66298	650.33513	433.89251	S	1165.60909	583.30818	389.20788	7
12	120.08078	1446.73139	723.86933	482.91531	F	1078.57706	539.79217	360.19721	6
13	120.08078	1593.79980	797.40354	531.93812	F	931.50865	466.25796	311.17440	5
14	86.09643	1706.88387	853.94557	569.63281	L	784.44024	392.72376	262.15160	4
15	181.06077	1914.93228	957.96978	638.98228	Y-Nitro	671.35617	336.18172	224.45691	3
16	60.04439	2001.96430	1001.48579	667.99295	S	463.30776	232.15752	155.10744	2
17	330.27026				K-TMT6plex	376.27574	188.64151	126.09676	1

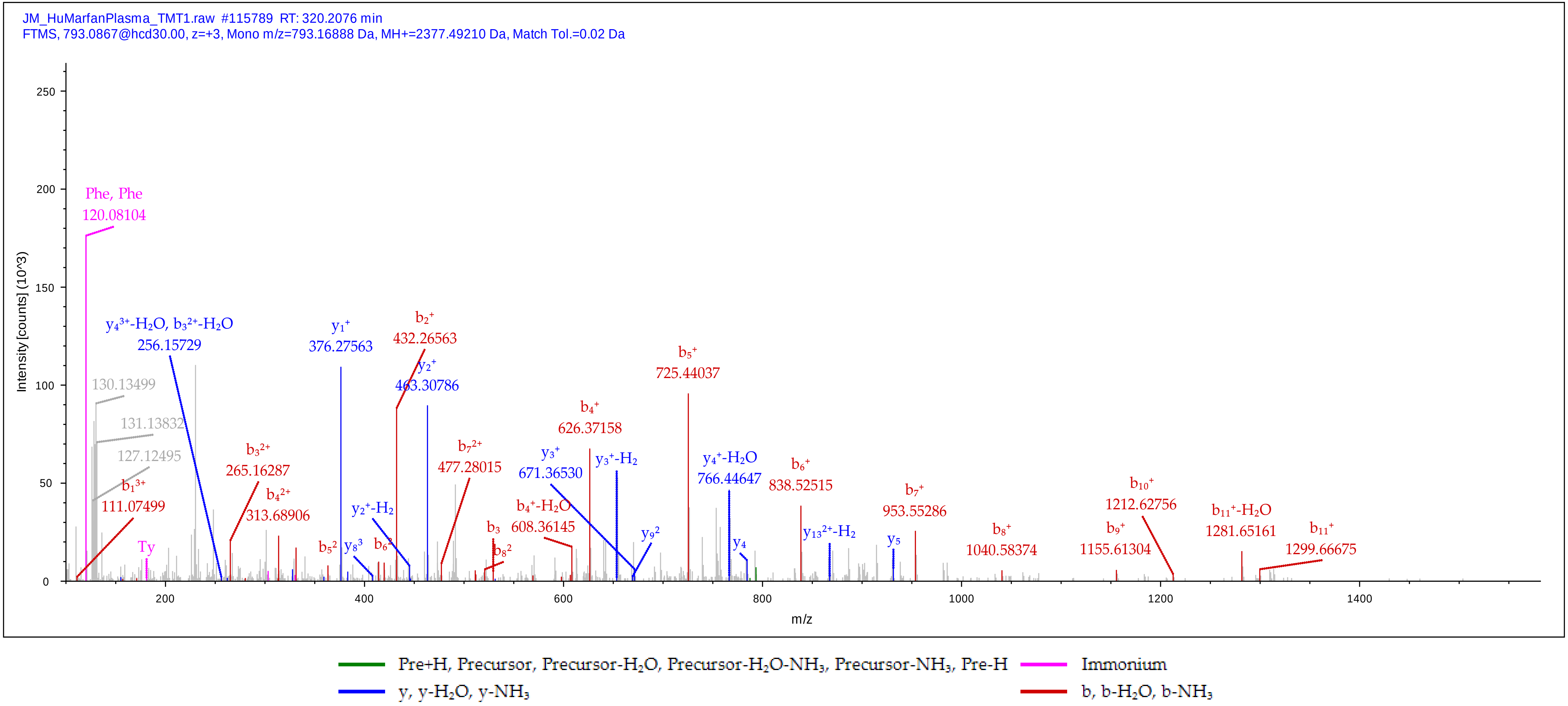

JM_HuMarfanPlasma_TMT1.raw #115789 RT: 320.2076 min
FTMS, 793.0867@hcd30.00, z=+3, Mono m/z=793.16888 Da, MH+=2377.49210 Da, Match Tol.=0.02 Da
Intensity [counts] (10^3)
m/z
Phe, Phe 120.08104
130.13499
131.13832
127.12495
b_1^{3+} 111.07499
Ty
y_4^{3+}-H_2O, b_3^{2+}-H_2O 256.15729
b_3^{2+} 265.16287
b_4^{2+} 313.68906
y_1^+ 376.27563
y_2^+-H_2
b_5^2
y_8^3
b_6^2
b_2^+ 432.26563
y_2^+ 463.30786
b_7^{2+} 477.28015
b_3
b_8^2
b_4^+-H_2O 608.36145
b_4^+ 626.37158
y_3^+ 671.36530
y_3^+-H_2
y_9^2
b_5^+ 725.44037
y_4^+-H_2O 766.44647
y_4
b_6^+ 838.52515
y_{13}^{2+}-H_2
y_5
b_7^+ 953.55286
b_8^+ 1040.58374
b_9^+ 1155.61304
b_{10}^+ 1212.62756
b_{11}^+-H_2O 1281.65161
b_{11}^+ 1299.66675
Pre+H, Precursor, Precursor-H_2O, Precursor-H_2O-NH_3, Precursor-NH_3, Pre-H
Immonium
y, y-H_2O, y-NH_3
b, b-H_2O, b-NH_3

#1	Immonium	b^{+}	b^{2+}	b^{3+}	b^{4+}	b^{5+}	Seq.	y^{+}	y^{2+}	y^{3+}	y^{4+}	y^{5+}	#2
1	303.22297	331.21789	166.11258	111.07748	83.55993	67.04940	T-TMT6plex						33
2	70.06513	428.27065	214.63896	143.42840	107.82312	86.45995	P	4245.05991	2123.03359	1415.69149	1062.02043	849.81780	32
3	102.05496	557.31324	279.16026	186.44260	140.08377	112.26847	E	4148.00714	2074.50721	1383.34057	1037.75724	830.40725	31
4	72.08078	656.38166	328.69447	219.46540	164.85087	132.08215	V	4018.96455	2009.98591	1340.32637	1005.49659	804.59873	30
5	74.06004	757.42934	379.21831	253.14796	190.11279	152.29169	T	3919.89614	1960.45171	1307.30356	980.72949	784.78505	29
6	133.04301	917.45998	459.23363	306.49151	230.12045	184.29782	Carbamidometl	3818.84846	1909.92787	1273.62100	955.46757	764.57551	28
7	72.08078	1016.52840	508.76784	339.51432	254.88756	204.11150	V	3658.81781	1829.91254	1220.27745	915.45991	732.56938	27
8	72.08078	1115.59681	558.30204	372.53712	279.65466	223.92518	V	3559.74940	1780.37834	1187.25465	890.69281	712.75570	26
9	72.08078	1214.66523	607.83625	405.55993	304.42176	243.73887	V	3460.68098	1730.84413	1154.23184	865.92570	692.94202	25
10	88.03930	1329.69217	665.34972	443.90224	333.17850	266.74426	D	3361.61257	1681.30992	1121.20904	841.15860	673.12833	24
11	72.08078	1428.76058	714.88393	476.92505	357.94560	286.55794	V	3246.58562	1623.79645	1082.86673	812.40186	650.12295	23
12	60.04439	1515.79261	758.39994	505.93572	379.70361	303.96434	S	3147.51721	1574.26224	1049.84392	787.63476	630.30926	22
13	110.07127	1652.85152	826.92940	551.62203	413.96834	331.37613	H	3060.48518	1530.74623	1020.83325	765.87675	612.90286	21
14	102.05496	1781.89412	891.45070	594.63622	446.22899	357.18464	E	2923.42627	1462.21677	975.14694	731.61203	585.49108	20
15	88.03930	1896.92106	948.96417	632.97854	474.98572	380.19003	D	2794.38368	1397.69548	932.13274	699.35138	559.68256	19
16	70.06513	1993.97382	997.49055	665.32946	499.24891	399.60059	P	2679.35673	1340.18201	893.79043	670.59464	536.67717	18
17	102.05496	2123.01642	1062.01185	708.34366	531.50956	425.40910	E	2582.30397	1291.65562	861.43951	646.33145	517.26662	17
18	72.08078	2222.08483	1111.54605	741.36646	556.27666	445.22279	V	2453.26138	1227.13433	818.42531	614.07080	491.45810	16
19	330.27026	2579.34272	1290.17500	860.45243	645.59114	516.67437	K-TMT6plex	2354.19296	1177.60012	785.40251	589.30370	471.64441	15
20	120.08078	2726.41114	1363.70921	909.47523	682.35824	546.08805	F	1996.93507	998.97117	666.31654	499.98922	400.19284	14
21	87.05529	2840.45407	1420.73067	947.48954	710.86897	568.89663	N	1849.86666	925.43697	617.29374	463.22212	370.77915	13
22	204.07675	3071.51846	1536.26287	1024.51100	768.63507	615.10951	W-Nitro	1735.82373	868.41550	579.27943	434.71139	347.97057	12
23	181.06077	3279.56686	1640.28707	1093.86047	820.64717	656.71919	Y-Nitro	1504.75934	752.88331	502.25796	376.94529	301.75769	11
24	72.08078	3378.63528	1689.82128	1126.88328	845.41428	676.53288	V	1296.71093	648.85910	432.90849	324.93319	260.14801	10
25	88.03930	3493.66222	1747.33475	1165.22559	874.17101	699.53827	D	1197.64252	599.32490	399.88569	300.16609	240.33432	9
26	30.03383	3550.68368	1775.84548	1184.23275	888.42638	710.94256	G	1082.61557	541.81143	361.54338	271.40935	217.32894	8
27	72.08078	3649.75210	1825.37969	1217.25555	913.19348	730.75624	V	1025.59411	513.30069	342.53622	257.15398	205.92464	7
28	102.05496	3778.79469	1889.90098	1260.26975	945.45413	756.56476	E	926.52570	463.76649	309.51342	232.38688	186.11096	6
29	72.08078	3877.86310	1939.43519	1293.29255	970.22123	776.37844	V	797.48310	399.24519	266.49922	200.12623	160.30244	5
30	110.07127	4014.92202	2007.96465	1338.97886	1004.48596	803.79022	H	698.41469	349.71098	233.47641	175.35913	140.48876	4
31	87.05529	4128.96494	2064.98611	1376.99317	1032.99669	826.59881	N	561.35578	281.18153	187.79011	141.09440	113.07698	3
32	44.04948	4200.00206	2100.50467	1400.67220	1050.75597	840.80623	A	447.31285	224.16006	149.77580	112.58367	90.26839	2
33	330.27026						K-TMT6plex	376.27574	188.64151	126.09676	94.82439	76.06097	1

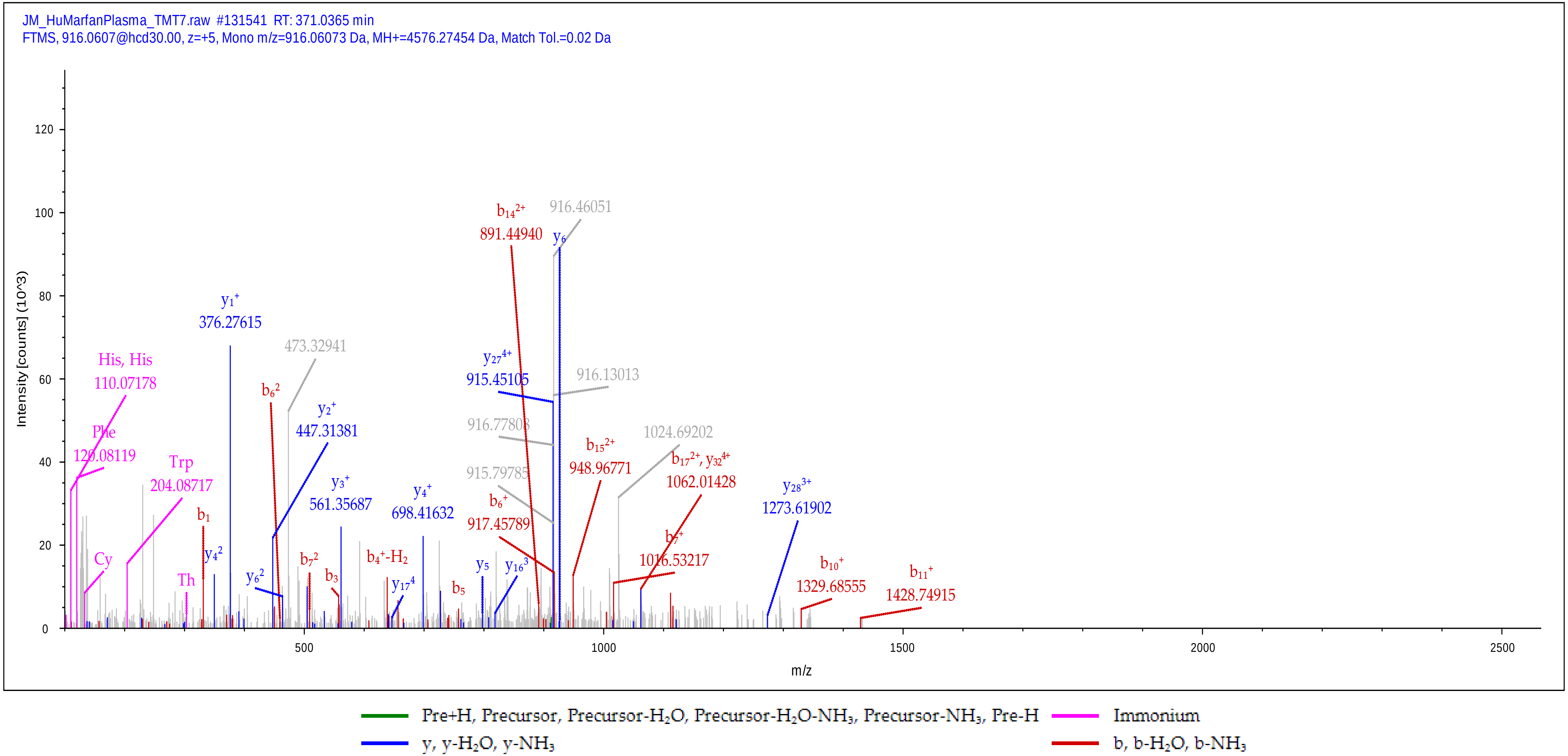

JM_HuMarfanPlasma_TMT7.raw #131541 RT: 371.0365 min
FTMS, 916.0607@hcd30.00, z=+5, Mono m/z=916.06073 Da, MH+=4576.27454 Da, Match Tol.=0.02 Da
Intensity [counts] (10^3)
m/z
His, His
110.07178
Phe
120.08119
Trp
204.08717
Cy
Th
b_1
y_1^+
376.27615
y_4^2
y_6^2
473.32941
b_6^2
y_2^+
447.31381
b_7^2
b_3
y_3^+
561.35687
b_4^+-H_2
y_{17}^4
y_4^+
698.41632
b_5
y_5
y_{16}^3
y_{27}^{4+}
915.45105
916.77808
915.79785
b_6^+
917.45789
b_{14}^{2+}
891.44940
916.46051
y_6
916.13013
b_{15}^{2+}
948.96771
1024.69202
b_{17}^{2+}, y_{32}^{4+}
1062.01428
b_7^+
1016.53217
y_{28}^{3+}
1273.61902
b_{10}^+
1329.68555
b_{11}^+
1428.74915
Pre+H, Precursor, Precursor-H_2O, Precursor-H_2O-NH_3, Precursor-NH_3, Pre-H
Immonium
y, y-H_2O, y-NH_3
b, b-H_2O, b-NH_3

#1	Immonium	b+	b2+	b3+	b4+	Seq.	y+	y2+	y3+	y4+	#2
1	316.21822	344.21314	172.61021	115.40923	86.80874	N-TMT6plex					30
2	120.08078	491.28155	246.14441	164.43203	123.57584	F	3328.62047	1664.81387	1110.21167	832.91057	29
3	70.06513	588.33431	294.67080	196.78296	147.83904	P	3181.55205	1591.27966	1061.18887	796.14347	28
4	70.06513	685.38708	343.19718	229.13388	172.10223	P	3084.49929	1542.75328	1028.83795	771.88028	27
5	60.04439	772.41911	386.71319	258.14455	193.86023	S	2987.44652	1494.22690	996.48703	747.61709	26
6	101.07094	900.47768	450.74248	300.83075	225.87488	Q	2900.41450	1450.71089	967.47635	725.85908	25
7	88.03930	1015.50463	508.25595	339.17306	254.63161	D	2772.35592	1386.68160	924.79016	693.84444	24
8	44.04948	1086.54174	543.77451	362.85210	272.39089	A	2657.32898	1329.16813	886.44784	665.08770	23
9	60.04439	1173.57377	587.29052	391.86277	294.14890	S	2586.29186	1293.64957	862.76881	647.32842	22
10	30.03383	1230.59523	615.80125	410.86993	308.40427	G	2499.25983	1250.13356	833.75813	625.57042	21
11	88.03930	1345.62217	673.31473	449.21224	337.16100	D	2442.23837	1221.62282	814.75097	611.31505	20
12	86.09643	1458.70624	729.85676	486.90693	365.43202	L	2327.21143	1164.10935	776.40866	582.55831	19
13	181.06077	1666.75465	833.88096	556.25640	417.44412	Y-Nitro	2214.12736	1107.56732	738.71397	554.28730	18
14	74.06004	1767.80232	884.40480	589.93896	442.70604	T	2006.07896	1003.54312	669.36450	502.27520	17
15	74.06004	1868.85000	934.92864	623.62152	467.96796	T	1905.03128	953.01928	635.68194	477.01328	16
16	60.04439	1955.88203	978.44465	652.63219	489.72597	S	1803.98360	902.49544	601.99938	451.75136	15
17	60.04439	2042.91406	1021.96067	681.64287	511.48397	S	1716.95157	858.97942	572.98871	429.99335	14
18	101.07094	2170.97264	1085.98996	724.32906	543.49862	Q	1629.91954	815.46341	543.97803	408.23534	13
19	86.09643	2284.05670	1142.53199	762.02375	571.76963	L	1501.86097	751.43412	501.29184	376.22070	12
20	74.06004	2385.10438	1193.05583	795.70631	597.03155	T	1388.77690	694.89209	463.59715	347.94968	11
21	86.09643	2498.18844	1249.59786	833.40100	625.30257	L	1287.72922	644.36825	429.91459	322.68776	10
22	70.06513	2595.24121	1298.12424	865.75192	649.56576	P	1174.64516	587.82622	392.21990	294.41675	9
23	44.04948	2666.27832	1333.64280	889.43096	667.32504	A	1077.59240	539.29984	359.86898	270.15356	8
24	74.06004	2767.32600	1384.16664	923.11352	692.58696	T	1006.55528	503.78128	336.18995	252.39428	7
25	101.07094	2895.38458	1448.19593	965.79971	724.60160	Q	905.50760	453.25744	302.50739	227.13236	6
26	133.04301	3055.41522	1528.21125	1019.14326	764.60926	C-Carbamidomethyl	777.44903	389.22815	259.82119	195.11771	5
27	86.09643	3168.49929	1584.75328	1056.83795	792.88028	L	617.41838	309.21283	206.47764	155.11005	4
28	44.04948	3239.53640	1620.27184	1080.51699	810.63956	A	504.33431	252.67080	168.78296	126.83904	3
29	30.03383	3296.55787	1648.78257	1099.52414	824.89492	G	433.29720	217.15224	145.10392	109.07976	2
30	330.27026					K-TMT6plex	376.27574	188.64151	126.09676	94.82439	1

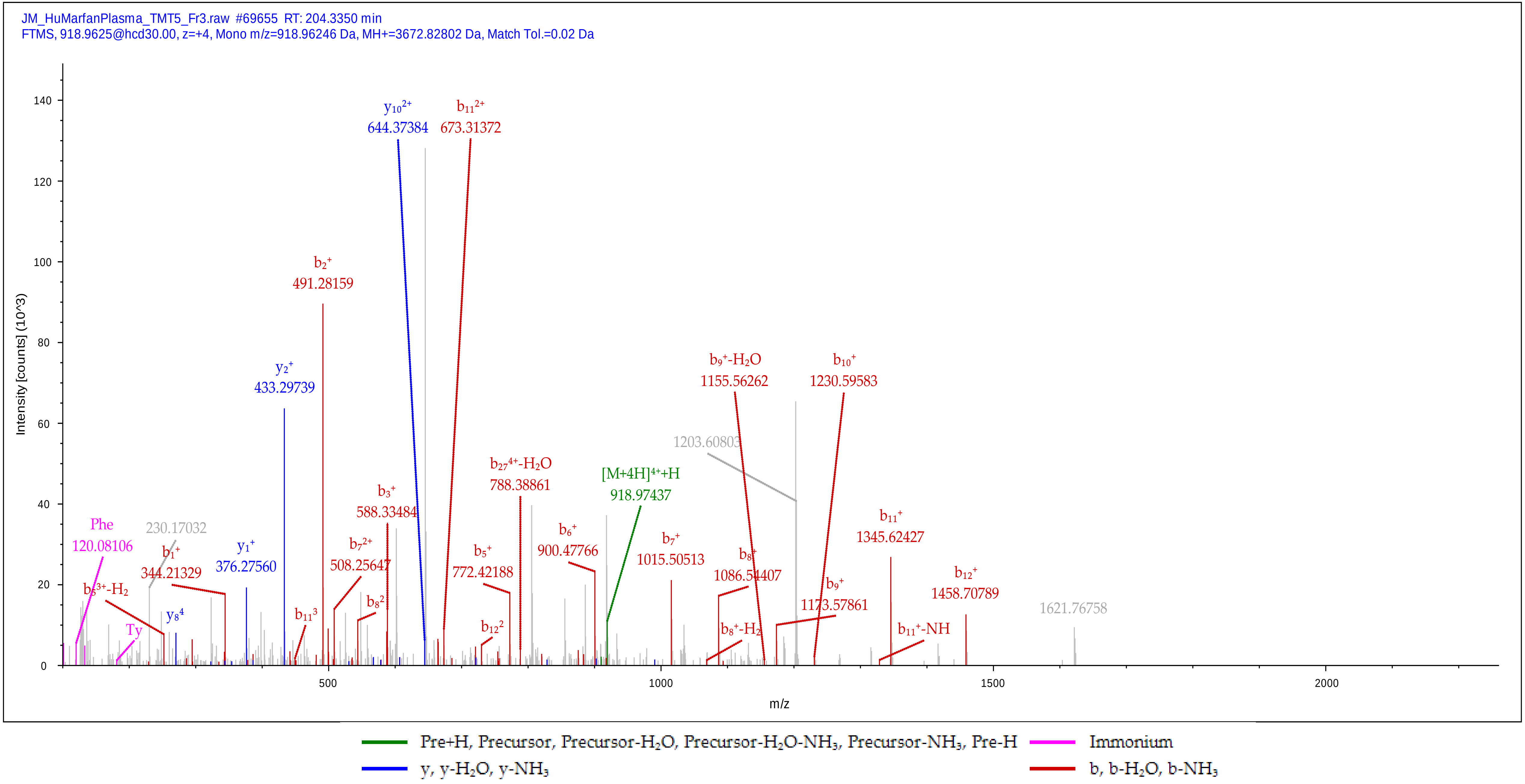
JM_HuMarfanPlasma_TMT5_Fr3.raw #69655 RT: 204.3350 min
FTMS, 918.9625@hcd30.00, z=+4, Mono m/z=918.96246 Da, MH+=3672.82802 Da, Match Tol.=0.02 Da
Intensity [counts] (10^3)
m/z
0
20
40
60
80
100
120
140
500
1000
1500
2000
y_{10}^{2+} 644.37384
b_{11}^{2+} 673.31372
b_2^+ 491.28159
y_2^+ 433.29739
b_9^+-H_2O 1155.56262
b_{10}^+ 1230.59583
1203.60803
b_{27}^{4+}-H_2O 788.38861
[M+4H]$^{4+}$+H 918.97437
b_3^+ 588.33484
Phe 120.08106
230.17032
b_1^+ 344.21329
y_1^+ 376.27560
b_7^{2+} 508.25647
b_5^+ 772.42188
b_6^+ 900.47766
b_7^+ 1015.50513
b_8^+ 1086.54407
b_{11}^+ 1345.62427
b_9^+ 1173.57861
b_{12}^+ 1458.70789
1621.76758
b_5^{3+}-H_2
y_8^4
Ty
b_{11}^3
b_8^2
b_{12}^2
b_8^+-H_2
b_{11}^+-NH
Pre+H, Precursor, Precursor-H_2O, Precursor-H_2O-NH_3, Precursor-NH_3, Pre-H
Immonium
y, y-H_2O, y-NH_3
b, b-H_2O, b-NH_3

#1	Immonium	b+	b2+	b3+	Seq.	y+	y2+	y3+	#2
1	259.19676	287.19167	144.09947	96.40208	G-TMT6plex				22
2	120.08078	434.26009	217.63368	145.42488	F	2777.25283	1389.13006	926.42246	21
3	136.07569	597.32341	299.16535	199.77932	Y	2630.18442	1315.59585	877.39966	20
4	70.06513	694.37618	347.69173	232.13024	P	2467.12109	1234.06418	823.04521	19
5	60.04439	781.40821	391.20774	261.14092	S	2370.06833	1185.53780	790.69429	18
6	88.03930	896.43515	448.72121	299.48323	D	2283.03630	1142.02179	761.68362	17
7	86.09643	1009.51921	505.26325	337.17792	I	2168.00936	1084.50832	723.34130	16
8	60.04439	1096.55124	548.77926	366.18860	S	2054.92529	1027.96628	685.64662	15
9	72.08078	1195.61966	598.31347	399.21140	V	1967.89326	984.45027	656.63594	14
10	102.05496	1324.66225	662.83476	442.22560	E	1868.82485	934.91606	623.61313	13
11	204.07675	1555.72664	778.36696	519.24706	W-Nitro	1739.78226	870.39477	580.59894	12
12	102.05496	1684.76923	842.88825	562.26126	E	1508.71787	754.86257	503.57747	11
13	60.04439	1771.80126	886.40427	591.27194	S	1379.67527	690.34127	460.56328	10
14	87.05529	1885.84419	943.42573	629.28625	N	1292.64324	646.82526	431.55260	9
15	30.03383	1942.86565	971.93646	648.29340	G	1178.60032	589.80380	393.53829	8
16	101.07094	2070.92423	1035.96575	690.97959	Q	1121.57885	561.29307	374.53114	7
17	70.06513	2167.97699	1084.49214	723.33052	P	993.52028	497.26378	331.84494	6
18	102.05496	2297.01959	1149.01343	766.34471	E	896.46751	448.73739	299.49402	5
19	87.05529	2411.06251	1206.03490	804.35902	N	767.42492	384.21610	256.47982	4
20	87.05529	2525.10544	1263.05636	842.37333	N	653.38199	327.19463	218.46552	3
21	136.07569	2688.16877	1344.58802	896.72777	Y	539.33906	270.17317	180.45121	2
22	330.27026				K-TMT6plex	376.27574	188.64151	126.09676	1

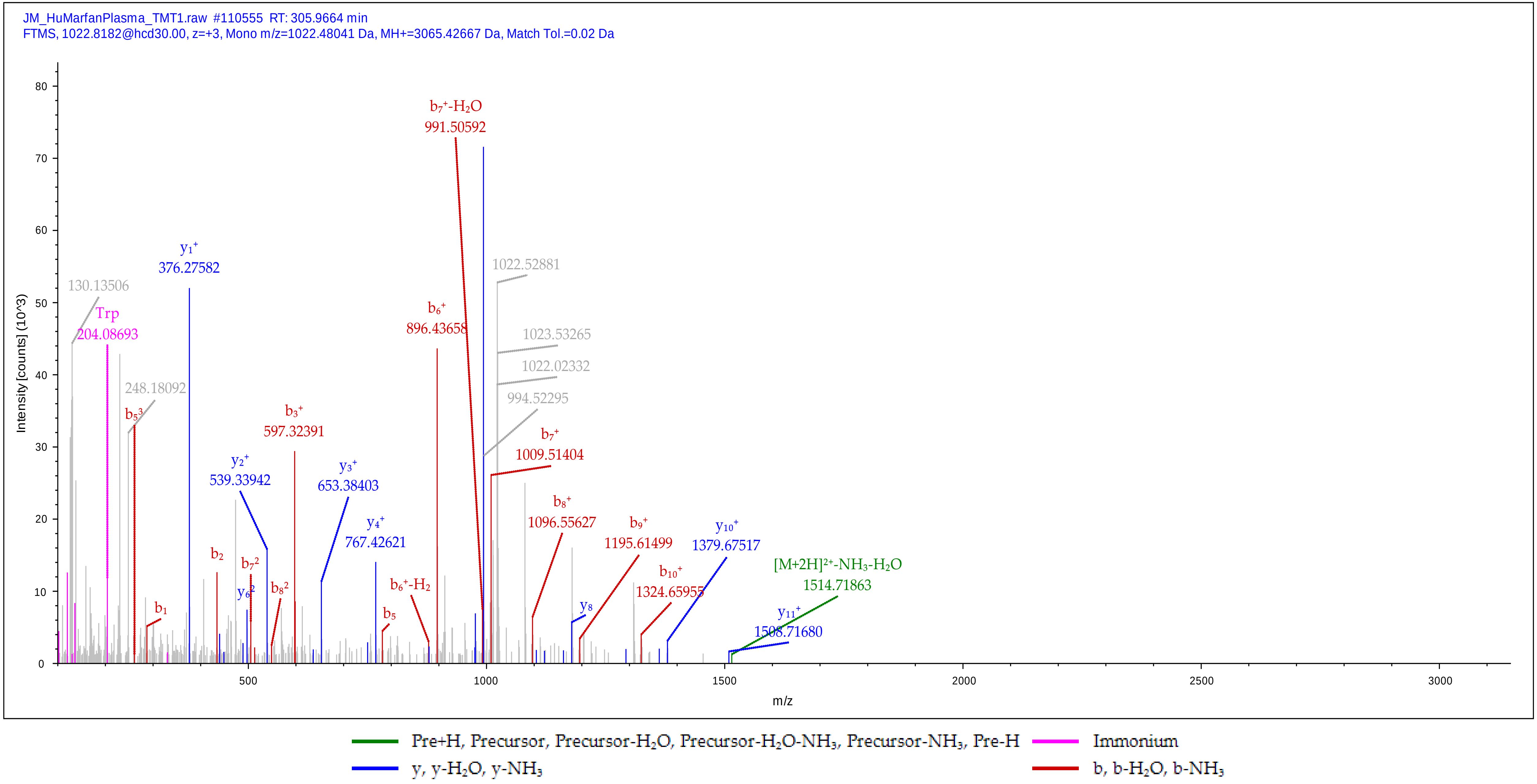

JM_HuMarfanPlasma_TMT1.raw #110555 RT: 305.9664 min
FTMS, 1022.8182@hcd30.00, z=+3, Mono m/z=1022.48041 Da, MH+=3065.42667 Da, Match Tol.=0.02 Da
Intensity [counts] (10^3)
m/z
130.13506
Trp
204.08693
248.18092
b_5^3
b_1
y_1^+
376.27582
b_2
y_2^+
539.33942
y_6^2
b_7^2
b_8^2
b_3^+
597.32391
y_3^+
653.38403
y_4^+
767.42621
b_5
b_6^+-H_2
b_6^+
896.43658
b_7^+-H_2O
991.50592
1022.52881
1023.53265
1022.02332
994.52295
b_7^+
1009.51404
b_8^+
1096.55627
y_8
b_9^+
1195.61499
b_{10}^+
1324.65955
y_{10}^+
1379.67517
y_{11}^+
1508.71680
$[M+2H]^{2+}$-NH_3-H_2O
1514.71863
Pre+H, Precursor, Precursor-H_2O, Precursor-H_2O-NH_3, Precursor-NH_3, Pre-H
Immonium
y, y-H_2O, y-NH_3
b, b-H_2O, b-NH_3

#1	Immonium	b^+	b^{2+}	b^{3+}	Seq.	y^+	y^{2+}	y^{3+}	#2
1	303.22297	331.21789	166.11258	111.07748	T-TMT6plex				17
2	74.06004	432.26557	216.63642	144.76004	T	2078.99422	1040.00075	693.66959	16
3	70.06513	529.31833	265.16280	177.11096	P	1977.94655	989.47691	659.98703	15
4	70.06513	626.37109	313.68918	209.46188	P	1880.89378	940.95053	627.63611	14
5	104.05285	757.41158	379.20943	253.14204	M	1783.84102	892.42415	595.28519	13
6	86.09643	870.49564	435.75146	290.83673	L	1652.80053	826.90390	551.60503	12
7	88.03930	985.52258	493.26493	329.17905	D	1539.71647	770.36187	513.91034	11
8	60.04439	1072.55461	536.78094	358.18972	S	1424.68953	712.84840	475.56803	10
9	88.03930	1187.58156	594.29442	396.53204	D	1337.65750	669.33239	446.55735	9
10	30.03383	1244.60302	622.80515	415.53919	G	1222.63055	611.81892	408.21504	8
11	60.04439	1331.63505	666.32116	444.54987	S	1165.60909	583.30818	389.20788	7
12	120.08078	1478.70346	739.85537	493.57267	F	1078.57706	539.79217	360.19721	6
13	120.08078	1625.77188	813.38958	542.59548	F	931.50865	466.25796	311.17440	5
14	86.09643	1738.85594	869.93161	580.29016	L	784.44024	392.72376	262.15160	4
15	181.06077	1946.90435	973.95581	649.63963	Y-Nitro	671.35617	336.18172	224.45691	3
16	60.04439	2033.93637	1017.47183	678.65031	S	463.30776	232.15752	155.10744	2
17	330.27026				K-TMT6plex	376.27574	188.64151	126.09676	1

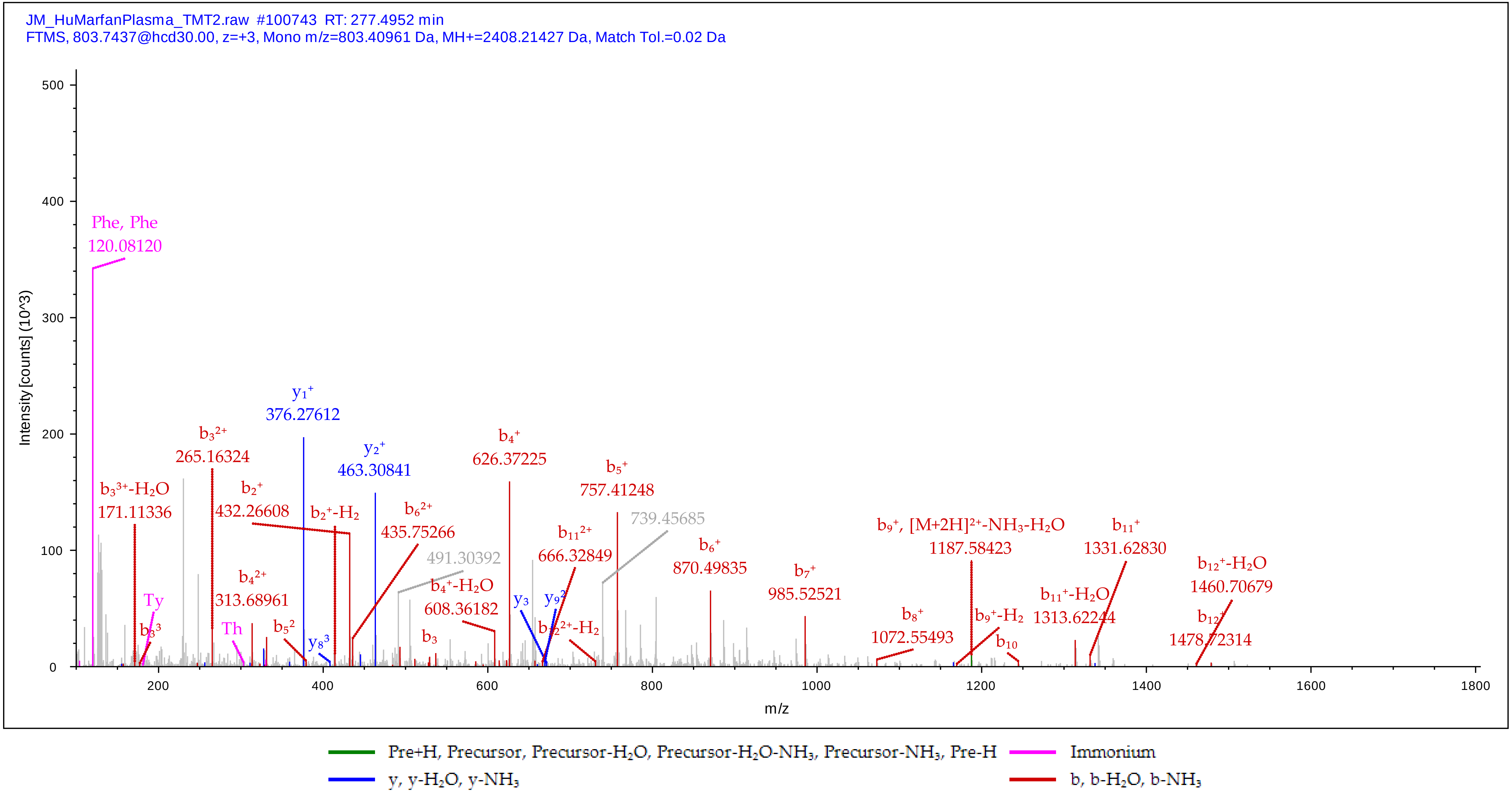
JM_HuMarfanPlasma_TMT2.raw #100743 RT: 277.4952 min
FTMS, 803.7437@hcd30.00, z=+3, Mono m/z=803.40961 Da, MH+=2408.21427 Da, Match Tol.=0.02 Da
Intensity [counts] (10^3)
m/z
Phe, Phe
120.08120
y_1^+
376.27612
y_2^+
463.30841
b_3^{2+}
265.16324
b_3^{3+}-H_2O
171.11336
b_2^+
432.26608
b_2^+-H_2
b_6^{2+}
435.75266
b_4^+
626.37225
b_5^+
757.41248
739.45685
491.30392
b_{11}^{2+}
666.32849
b_4^{2+}
313.68961
b_4^+-H_2O
608.36182
b_6^+
870.49835
b_7^+
985.52521
b_9^+, $[M+2H]^{2+}$-NH_3-H_2O
1187.58423
b_{11}^+
1331.62830
b_{12}^+-H_2O
1460.70679
b_8^+
1072.55493
b_9^+-H_2
b_{11}^+-H_2O
1313.62244
b_{10}
1478.72314
Ty
Th
b_5^2
y_8^3
y_3
b_3
Pre+H, Precursor, Precursor-H_2O, Precursor-H_2O-NH_3, Precursor-NH_3, Pre-H
Immonium
y, y-H_2O, y-NH_3
b, b-H_2O, b-NH_3

#1	Immonium	b⁺	b²⁺	b³⁺	Seq.	y⁺	y²⁺	y³⁺	#2
1	301.24371	329.23862	165.12295	110.41773	V-TMT6plex				16
2	72.08078	428.30704	214.65716	143.44053	V	1969.07044	985.03886	657.02833	15
3	60.04439	515.33906	258.17317	172.45121	S	1870.00203	935.50465	624.00553	14
4	72.08078	614.40748	307.70738	205.47401	V	1782.97000	891.98864	594.99485	13
5	86.09643	727.49154	364.24941	243.16870	L	1683.90158	842.45443	561.97205	12
6	74.06004	828.53922	414.77325	276.85126	T	1570.81752	785.91240	524.27736	11
7	72.08078	927.60763	464.30746	309.87406	V	1469.76984	735.38856	490.59480	10
8	72.08078	1026.67605	513.84166	342.89687	V	1370.70143	685.85435	457.57199	9
9	110.07127	1163.73496	582.37112	388.58317	H	1271.63301	636.32015	424.54919	8
10	101.07094	1291.79354	646.40041	431.26936	Q	1134.57410	567.79069	378.86289	7
11	88.03930	1406.82048	703.91388	469.61168	D	1006.51553	503.76140	336.17669	6
12	204.07675	1637.88487	819.44607	546.63314	W-Nitro	891.48858	446.24793	297.83438	5
13	86.09643	1750.96894	875.98811	584.32783	L	660.42419	330.71573	220.81291	4
14	87.05529	1865.01186	933.00957	622.34214	N	547.34013	274.17370	183.11823	3
15	30.03383	1922.03333	961.52030	641.34929	G	433.29720	217.15224	145.10392	2
16	330.27026				K-TMT6plex	376.27574	188.64151	126.09676	1

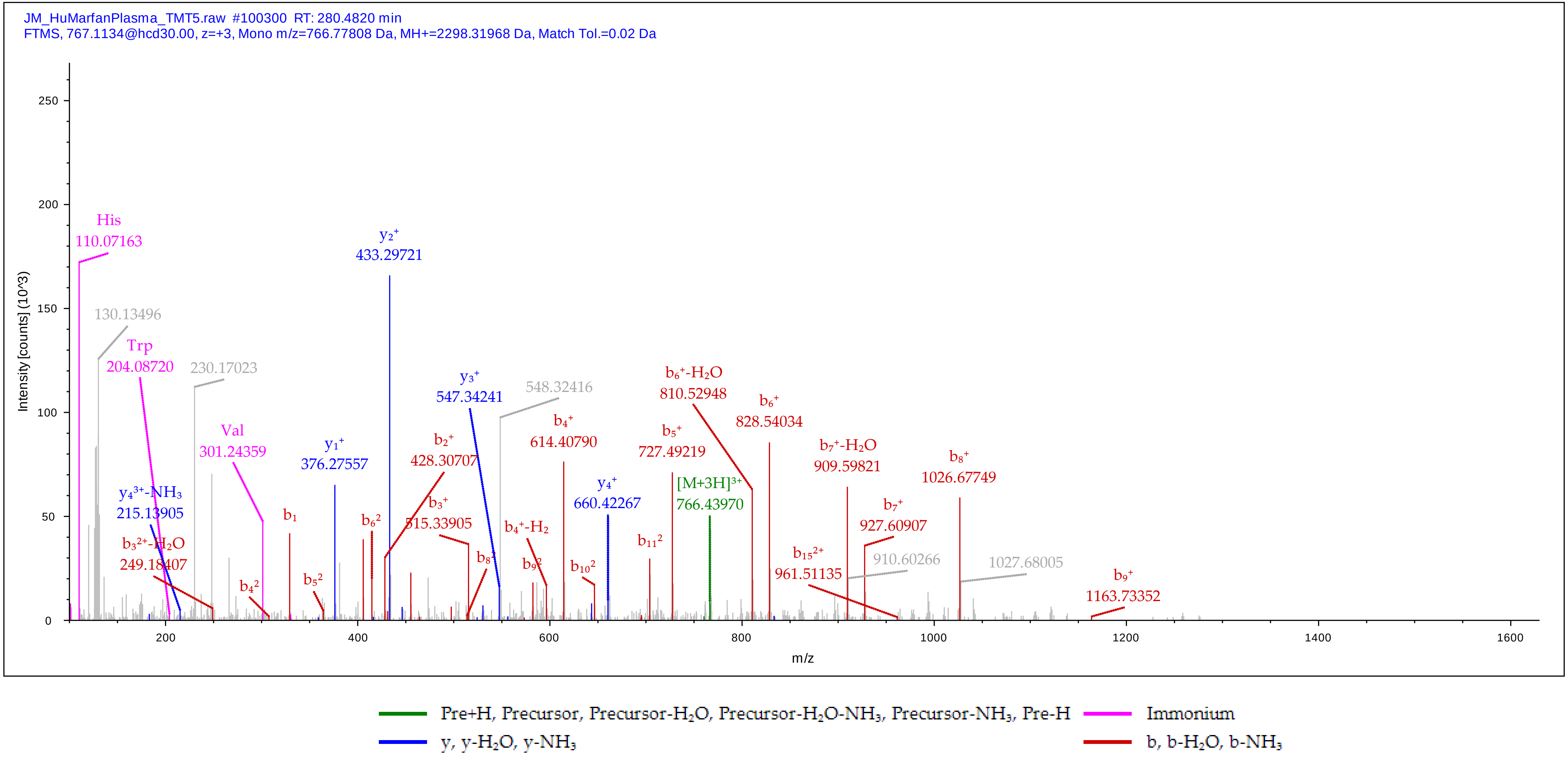

JM_HuMarfanPlasma_TMT5.raw #100300 RT: 280.4820 min
FTMS, 767.1134@hcd30.00, z=+3, Mono m/z=766.77808 Da, MH+=2298.31968 Da, Match Tol.=0.02 Da
Intensity [counts] (10^3)
m/z
His 110.07163
130.13496
Trp 204.08720
230.17023
Val 301.24359
y2+ 433.29721
y3+ 547.34241
548.32416
y1+ 376.27557
b2+ 428.30707
b4+ 614.40790
b6+-H2O 810.52948
b6+ 828.54034
b5+ 727.49219
b7+-H2O 909.59821
b8+ 1026.67749
y4+ 660.42267
[M+3H]3+ 766.43970
y43+-NH3 215.13905
b32+-H2O 249.18407
b1
b62
b3+ 315.33905
b4+-H2
b7+ 927.60907
b112
b152+ 961.51135
910.60266
1027.68005
b42
b52
b82
b92
b102
b9+ 1163.73352
Pre+H, Precursor, Precursor-H2O, Precursor-H2O-NH3, Precursor-NH3, Pre-H
Immonium
y, y-H2O, y-NH3
b, b-H2O, b-NH3

#1	Immonium	b^{+}	b^{2+}	b^{3+}	b^{4+}	b^{5+}	Seq.	y^{+}	y^{2+}	y^{3+}	y^{4+}	y^{5+}	#2
1	303.22297	331.21789	166.11258	111.07748	83.55993	67.04940	T-TMT6plex						33
2	70.06513	428.27065	214.63896	143.42840	107.82312	86.45995	P	4015.86059	2008.43393	1339.29171	1004.72060	803.97794	32
3	102.05496	557.31324	279.16026	186.44260	140.08377	112.26847	E	3918.80783	1959.90755	1306.94079	980.45741	784.56739	31
4	72.08078	656.38166	328.69447	219.46540	164.85087	132.08215	V	3789.76523	1895.38625	1263.92660	948.19677	758.75887	30
5	74.06004	757.42934	379.21831	253.14796	190.11279	152.29169	T	3690.69682	1845.85205	1230.90379	923.42966	738.94519	29
6	133.04301	917.45998	459.23363	306.49151	230.12045	184.29782	C-Carbamidomethyl	3589.64914	1795.32821	1197.22123	898.16774	718.73565	28
7	72.08078	1016.52840	508.76784	339.51432	254.88756	204.11150	V	3429.61849	1715.31288	1143.87768	858.16008	686.72952	27
8	72.08078	1115.59681	558.30204	372.53712	279.65466	223.92518	V	3330.55008	1665.77868	1110.85488	833.39298	666.91584	26
9	72.08078	1214.66523	607.83625	405.55993	304.42176	243.73887	V	3231.48166	1616.24447	1077.83207	808.62587	647.10215	25
10	88.03930	1329.69217	665.34972	443.90224	333.17850	266.74426	D	3132.41325	1566.71026	1044.80927	783.85877	627.28847	24
11	72.08078	1428.76058	714.88393	476.92505	357.94560	286.55794	V	3017.38631	1509.19679	1006.46695	755.10203	604.28308	23
12	60.04439	1515.79261	758.39994	505.93572	379.70361	303.96434	S	2918.31789	1459.66258	973.44415	730.33493	584.46940	22
13	110.07127	1652.85152	826.92940	551.62203	413.96834	331.37613	H	2831.28586	1416.14657	944.43347	708.57692	567.06299	21
14	102.05496	1781.89412	891.45070	594.63622	446.22899	357.18464	E	2694.22695	1347.61711	898.74717	674.31220	539.65121	20
15	88.03930	1896.92106	948.96417	632.97854	474.98572	380.19003	D	2565.18436	1283.09582	855.73297	642.05155	513.84269	19
16	70.06513	1993.97382	997.49055	665.32946	499.24891	399.60059	P	2450.15742	1225.58235	817.39066	613.29481	490.83730	18
17	102.05496	2123.01642	1062.01185	708.34366	531.50956	425.40910	E	2353.10465	1177.05597	785.03974	589.03162	471.42675	17
18	72.08078	2222.08483	1111.54605	741.36646	556.27666	445.22279	V	2224.06206	1112.53467	742.02554	556.77097	445.61823	16
19	101.07094	2350.14341	1175.57534	784.05265	588.29131	470.83450	Q	2124.99365	1063.00046	709.00273	532.00387	425.80455	15
20	120.08078	2497.21182	1249.10955	833.07546	625.05841	500.24819	F	1996.93507	998.97117	666.31654	499.98922	400.19284	14
21	87.05529	2611.25475	1306.13101	871.08977	653.56914	523.05677	N	1849.86666	925.43697	617.29374	463.22212	370.77915	13
22	204.07675	2842.31914	1421.66321	948.11123	711.33524	569.26965	W-Nitro	1735.82373	868.41550	579.27943	434.71139	347.97057	12
23	181.06077	3050.36755	1525.68741	1017.46070	763.34734	610.87933	Y-Nitro	1504.75934	752.88331	502.25796	376.94529	301.75769	11
24	72.08078	3149.43596	1575.22162	1050.48350	788.11445	630.69301	V	1296.71093	648.85910	432.90849	324.93319	260.14801	10
25	88.03930	3264.46290	1632.73509	1088.82582	816.87118	653.69840	D	1197.64252	599.32490	399.88569	300.16609	240.33432	9
26	30.03383	3321.48437	1661.24582	1107.83297	831.12655	665.10269	G	1082.61557	541.81143	361.54338	271.40935	217.32894	8
27	72.08078	3420.55278	1710.78003	1140.85578	855.89365	684.91638	V	1025.59411	513.30069	342.53622	257.15398	205.92464	7
28	102.05496	3549.59537	1775.30132	1183.86998	888.15430	710.72490	E	926.52570	463.76649	309.51342	232.38688	186.11096	6
29	72.08078	3648.66379	1824.83553	1216.89278	912.92140	730.53858	V	797.48310	399.24519	266.49922	200.12623	160.30244	5
30	110.07127	3785.72270	1893.36499	1262.57908	947.18613	757.95036	H	698.41469	349.71098	233.47641	175.35913	140.48876	4
31	87.05529	3899.76563	1950.38645	1300.59339	975.69686	780.75895	N	561.35578	281.18153	187.79011	141.09440	113.07698	3
32	44.04948	3970.80274	1985.90501	1324.27243	993.45614	794.96637	A	447.31285	224.16006	149.77580	112.58367	90.26839	2
33	330.27026						K-TMT6plex	376.27574	188.64151	126.09676	94.82439	76.06097	1

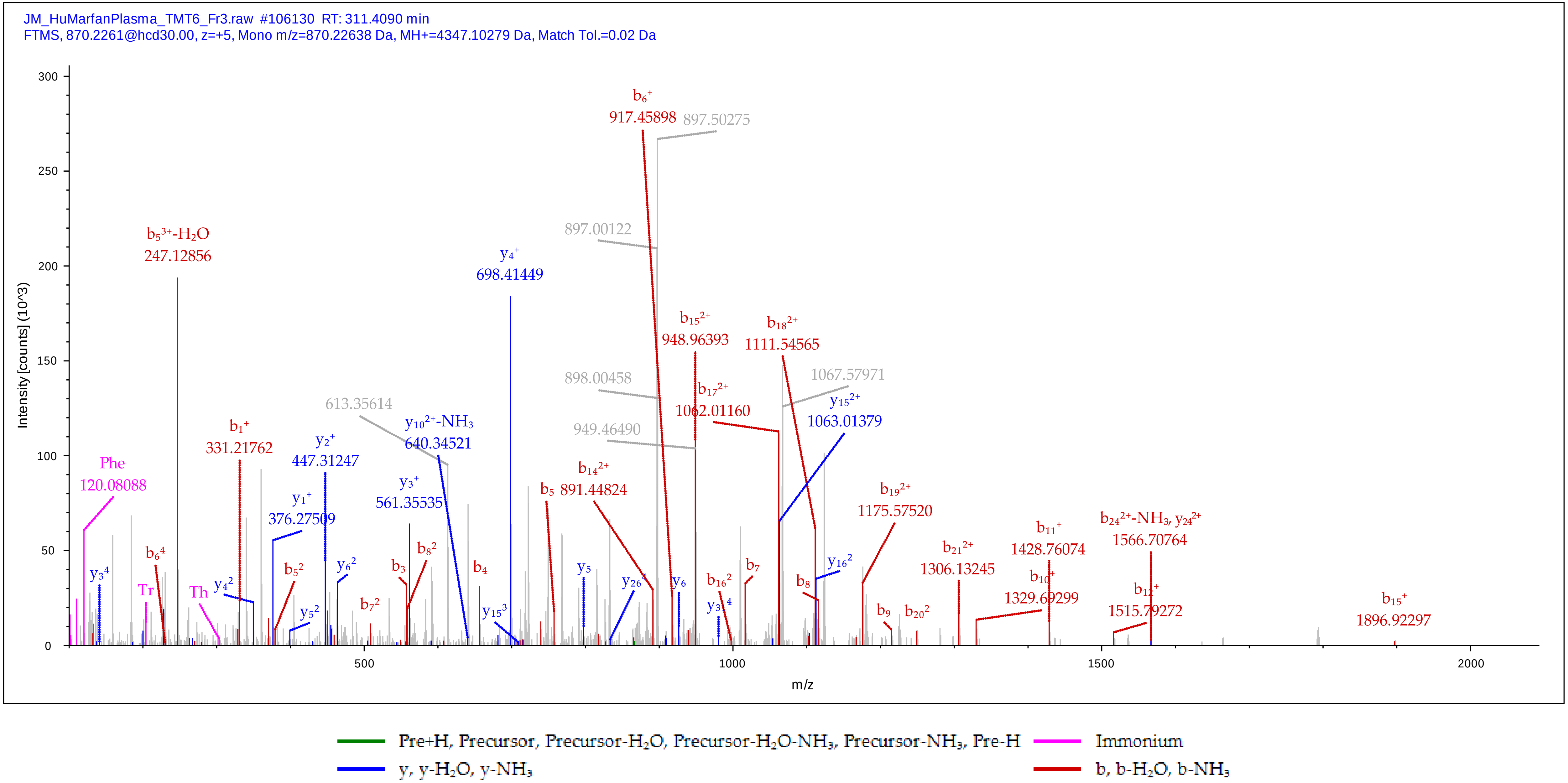

JM_HuMarfanPlasma_TMT6_Fr3.raw #106130 RT: 311.4090 min
FTMS, 870.2261@hcd30.00, z=+5, Mono m/z=870.22638 Da, MH+=4347.10279 Da, Match Tol.=0.02 Da
Intensity [counts] (10^3)
m/z
Phe 120.08088
b5 3+-H2O 247.12856
b1+ 331.21762
y1+ 376.27509
y2+ 447.31247
613.35614
y10 2+-NH3 640.34521
y3+ 561.35535
y4+ 698.41449
b5 891.44824
b14 2+
b6+ 917.45898
897.50275
897.00122
898.00458
949.46490
b15 2+ 948.96393
b17 2+ 1062.01160
b18 2+ 1111.54565
1067.57971
y15 2+ 1063.01379
b19 2+ 1175.57520
b21 2+ 1306.13245
b11+ 1428.76074
b10+ 1329.69299
b24 2+-NH3, y24 2+ 1566.70764
b12+ 1515.79272
b15+ 1896.92297
Pre+H, Precursor, Precursor-H2O, Precursor-H2O-NH3, Precursor-NH3, Pre-H
Immonium
y, y-H2O, y-NH3
b, b-H2O, b-NH3

#1	Immonium	b⁺	b²⁺	Seq.	y⁺	y²⁺	#2
1	433.23968	461.23460	231.12094	W-TMT6plex-Nitro			12
2	181.06077	669.28301	335.14514	Y-Nitro	1504.75934	752.88331	11
3	72.08078	768.35142	384.67935	V	1296.71093	648.85910	10
4	88.03930	883.37836	442.19282	D	1197.64252	599.32490	9
5	30.03383	940.39983	470.70355	G	1082.61557	541.81143	8
6	72.08078	1039.46824	520.23776	V	1025.59411	513.30069	7
7	102.05496	1168.51083	584.75906	E	926.52570	463.76649	6
8	72.08078	1267.57925	634.29326	V	797.48310	399.24519	5
9	110.07127	1404.63816	702.82272	H	698.41469	349.71098	4
10	87.05529	1518.68109	759.84418	N	561.35578	281.18153	3
11	44.04948	1589.71820	795.36274	A	447.31285	224.16006	2
12	330.27026			K-TMT6plex	376.27574	188.64151	1

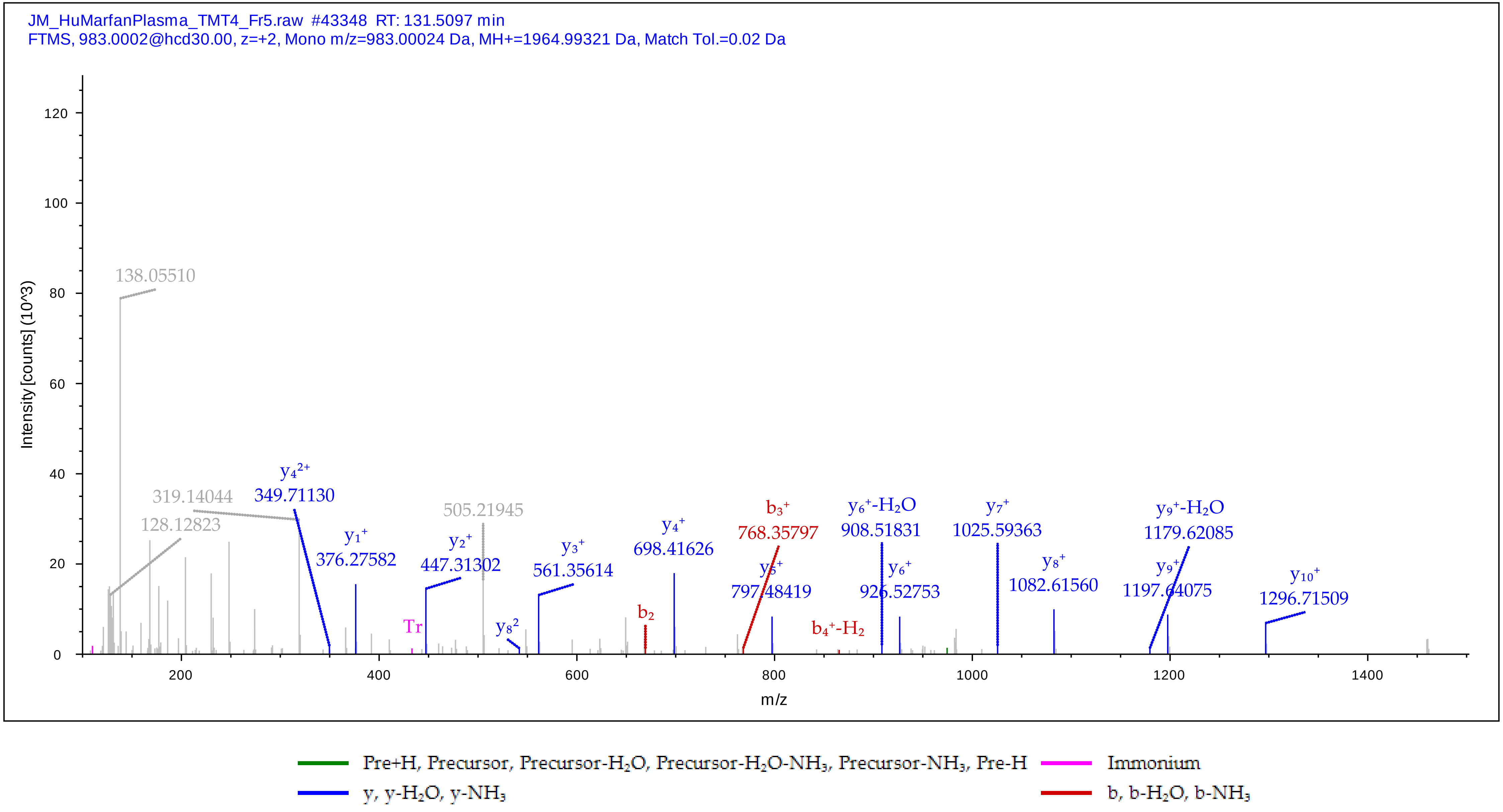

JM_HuMarfanPlasma_TMT4_Fr5.raw #43348 RT: 131.5097 min
FTMS, 983.0002@hcd30.00, z=+2, Mono m/z=983.00024 Da, MH+=1964.99321 Da, Match Tol.=0.02 Da
Intensity [counts] (10^3)
m/z
138.05510
319.14044
128.12823
349.71130
376.27582
447.31302
505.21945
561.35614
698.41626
768.35797
797.48419
908.51831
926.52753
1025.59363
1082.61560
1179.62085
1197.64075
1296.71509
Tr
Pre+H, Precursor, Precursor-H2O, Precursor-H2O-NH3, Precursor-NH3, Pre-H
Immonium
y, y-H2O, y-NH3
b, b-H2O, b-NH3

#1	Immonium	b^{+}	b^{2+}	b^{3+}	b^{4+}	Seq.	y^{+}	y^{2+}	y^{3+}	y^{4+}	#2
1	303.22297	331.21789	166.11258	111.07748	83.55993	T-TMT6plex					33
2	70.06513	428.27065	214.63896	143.42840	107.82312	P	4006.86025	2003.93377	1336.29160	1002.47052	32
3	102.05496	557.31324	279.16026	186.44260	140.08377	E	3909.80749	1955.40738	1303.94068	978.20733	31
4	72.08078	656.38166	328.69447	219.46540	164.85087	V	3780.76490	1890.88609	1260.92648	945.94668	30
5	74.06004	757.42934	379.21831	253.14796	190.11279	T	3681.69648	1841.35188	1227.90368	921.17958	29
6	133.04301	917.45998	459.23363	306.49151	230.12045	C-Carbamidomethyl	3580.64881	1790.82804	1194.22112	895.91766	28
7	72.08078	1016.52840	508.76784	339.51432	254.88756	V	3420.61816	1710.81272	1140.87757	855.91000	27
8	72.08078	1115.59681	558.30204	372.53712	279.65466	V	3321.54974	1661.27851	1107.85477	831.14289	26
9	72.08078	1214.66523	607.83625	405.55993	304.42176	V	3222.48133	1611.74430	1074.83196	806.37579	25
10	88.03930	1329.69217	665.34972	443.90224	333.17850	D	3123.41292	1562.21010	1041.80916	781.60869	24
11	72.08078	1428.76058	714.88393	476.92505	357.94560	V	3008.38597	1504.69662	1003.46684	752.85195	23
12	60.04439	1515.79261	758.39994	505.93572	379.70361	S	2909.31756	1455.16242	970.44404	728.08485	22
13	101.07094	1643.85119	822.42923	548.62191	411.71825	Q	2822.28553	1411.64640	941.43336	706.32684	21
14	102.05496	1772.89378	886.95053	591.63611	443.97890	E	2694.22695	1347.61711	898.74717	674.31220	20
15	88.03930	1887.92072	944.46400	629.97843	472.73564	D	2565.18436	1283.09582	855.73297	642.05155	19
16	70.06513	1984.97349	992.99038	662.32935	496.99883	P	2450.15742	1225.58235	817.39066	613.29481	18
17	102.05496	2114.01608	1057.51168	705.34354	529.25948	E	2353.10465	1177.05597	785.03974	589.03162	17
18	72.08078	2213.08450	1107.04589	738.36635	554.02658	V	2224.06206	1112.53467	742.02554	556.77097	16
19	101.07094	2341.14307	1171.07517	781.05254	586.04123	Q	2124.99365	1063.00046	709.00273	532.00387	15
20	120.08078	2488.21149	1244.60938	830.07535	622.80833	F	1996.93507	998.97117	666.31654	499.98922	14
21	87.05529	2602.25441	1301.63085	868.08966	651.31906	N	1849.86666	925.43697	617.29374	463.22212	13
22	204.07675	2833.31881	1417.16304	945.11112	709.08516	W-Nitro	1735.82373	868.41550	579.27943	434.71139	12
23	181.06077	3041.36721	1521.18724	1014.46059	761.09726	Y-Nitro	1504.75934	752.88331	502.25796	376.94529	11
24	72.08078	3140.43563	1570.72145	1047.48339	785.86436	V	1296.71093	648.85910	432.90849	324.93319	10
25	88.03930	3255.46257	1628.23492	1085.82571	814.62110	D	1197.64252	599.32490	399.88569	300.16609	9
26	30.03383	3312.48403	1656.74565	1104.83286	828.87647	G	1082.61557	541.81143	361.54338	271.40935	8
27	72.08078	3411.55245	1706.27986	1137.85567	853.64357	V	1025.59411	513.30069	342.53622	257.15398	7
28	102.05496	3540.59504	1770.80116	1180.86986	885.90422	E	926.52570	463.76649	309.51342	232.38688	6
29	72.08078	3639.66345	1820.33536	1213.89267	910.67132	V	797.48310	399.24519	266.49922	200.12623	5
30	110.07127	3776.72236	1888.86482	1259.57897	944.93605	H	698.41469	349.71098	233.47641	175.35913	4
31	87.05529	3890.76529	1945.88628	1297.59328	973.44678	N	561.35578	281.18153	187.79011	141.09440	3
32	44.04948	3961.80241	1981.40484	1321.27232	991.20606	A	447.31285	224.16006	149.77580	112.58367	2
33	330.27026					K-TMT6plex	376.27574	188.64151	126.09676	94.82439	1

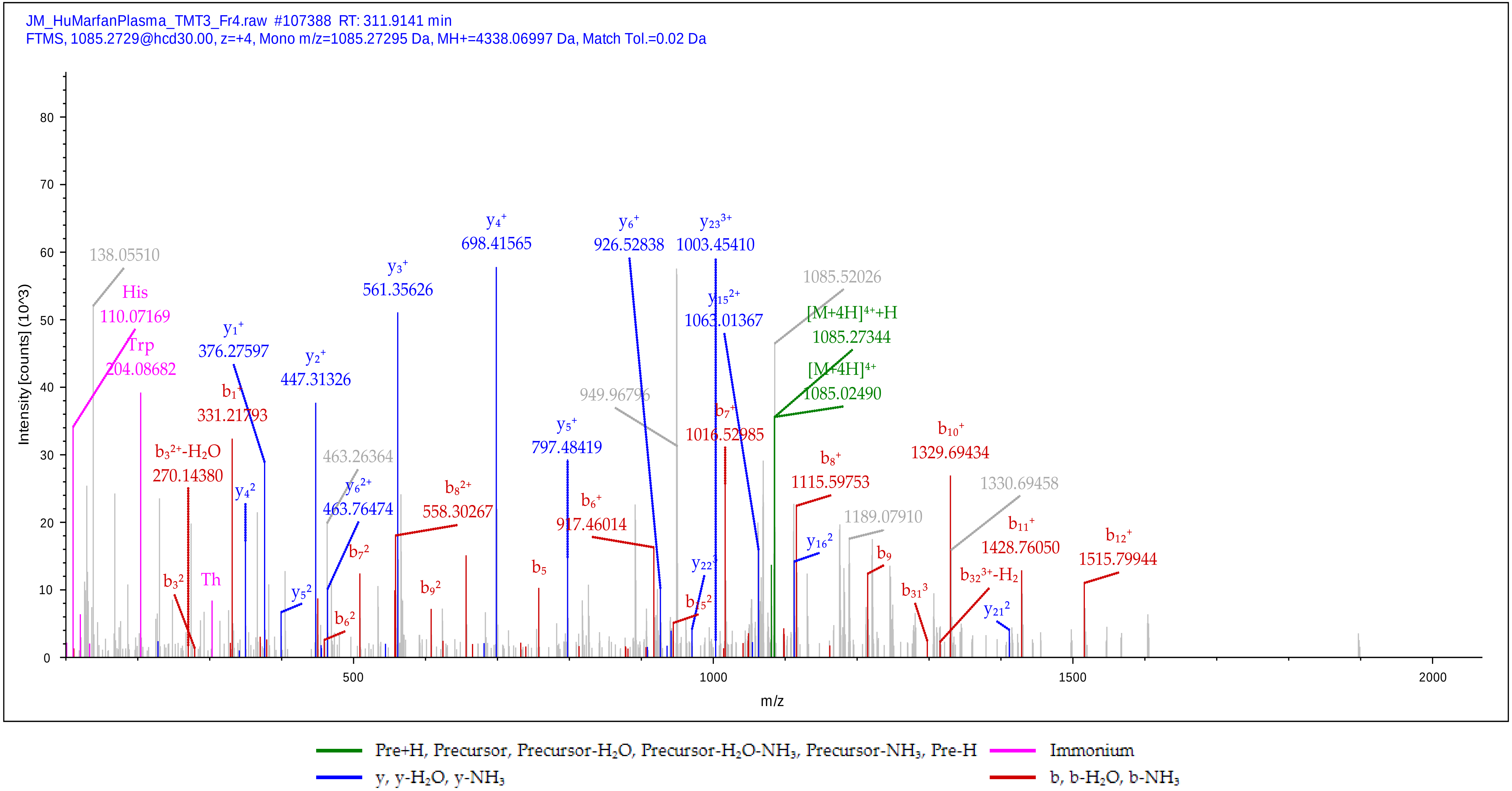
JM_HuMarfanPlasma_TMT3_Fr4.raw #107388 RT: 311.9141 min
FTMS, 1085.2729@hcd30.00, z=+4, Mono m/z=1085.27295 Da, MH+=4338.06997 Da, Match Tol.=0.02 Da
Intensity [counts] (10^3)
m/z
138.05510
His 110.07169
Trp 204.08682
y_1^+ 376.27597
b_1^+ 331.21793
b_3^{2+}-H_2O 270.14380
b_3^2
Th
y_4^2
y_2^+ 447.31326
y_3^+ 561.35626
y_5^2
463.26364
y_6^{2+} 463.76474
b_7^2
b_6^2
b_8^{2+} 558.30267
b_9^2
y_4^+ 698.41565
b_5
y_5^+ 797.48419
b_6^+ 917.46014
949.96796
y_6^+ 926.52838
y_{23}^{3+} 1003.45410
y_{15}^{2+} 1063.01367
y_{22}^2
b_{15}^2
b_7^+ 1016.52985
1085.52026
$[M+4H]^{4+}$+H 1085.27344
$[M+4H]^{4+}$ 1085.02490
b_8^+ 1115.59753
y_{16}^2
1189.07910
b_9
b_{31}^3
b_{10}^+ 1329.69434
1330.69458
b_{32}^{3+}-H_2
y_{21}^2
b_{11}^+ 1428.76050
b_{12}^+ 1515.79944
Pre+H, Precursor, Precursor-H_2O, Precursor-H_2O-NH_3, Precursor-NH_3, Pre-H
Immonium
y, y-H_2O, y-NH_3
b, b-H_2O, b-NH_3

#1	Immonium	b^{+}	b^{2+}	b^{3+}	b^{4+}	b^{5+}	Seq.	y^{+}	y^{2+}	y^{3+}	y^{4+}	y^{5+}	#2
1	301.24371	329.23862	165.12295	110.41773	83.06511	66.65355	V-TMT6plex						34
2	88.03930	444.26557	222.63642	148.76004	111.82185	89.65893	D	4023.95186	2012.47957	1341.98880	1006.74342	805.59619	33
3	87.05529	558.30849	279.65788	186.77435	140.33258	112.46752	N	3908.92491	1954.96609	1303.64649	977.98669	782.59080	32
4	44.04948	629.34561	315.17644	210.45339	158.09186	126.67494	A	3794.88199	1897.94463	1265.63218	949.47595	759.78222	31
5	86.09643	742.42967	371.71847	248.14807	186.36288	149.29176	L	3723.84487	1862.42607	1241.95314	931.71668	745.57480	30
6	101.07094	870.48825	435.74776	290.83427	218.37752	174.90347	Q	3610.76081	1805.88404	1204.25845	903.44566	722.95798	29
7	60.04439	957.52028	479.26378	319.84494	240.13553	192.30988	S	3482.70223	1741.85475	1161.57226	871.43101	697.34627	28
8	30.03383	1014.54174	507.77451	338.85210	254.39089	203.71417	G	3395.67020	1698.33874	1132.56158	849.67301	679.93986	27
9	87.05529	1128.58467	564.79597	376.86641	282.90162	226.52275	N	3338.64874	1669.82801	1113.55443	835.41764	668.53557	26
10	60.04439	1215.61670	608.31199	405.87708	304.65963	243.92916	S	3224.60581	1612.80654	1075.54012	806.90691	645.72698	25
11	101.07094	1343.67527	672.34127	448.56328	336.67428	269.54088	Q	3137.57378	1569.29053	1046.52945	785.14890	628.32058	24
12	102.05496	1472.71787	736.86257	491.57747	368.93492	295.34939	E	3009.51520	1505.26124	1003.84325	753.13426	602.70886	23
13	60.04439	1559.74989	780.37859	520.58815	390.69293	312.75580	S	2880.47261	1440.73994	960.82906	720.87361	576.90034	22
14	72.08078	1658.81831	829.91279	553.61095	415.46003	332.56948	V	2793.44058	1397.22393	931.81838	699.11560	559.49394	21
15	74.06004	1759.86599	880.43663	587.29351	440.72195	352.77902	T	2694.37217	1347.68972	898.79557	674.34850	539.68026	20
16	102.05496	1888.90858	944.95793	630.30771	472.98260	378.58754	E	2593.32449	1297.16588	865.11301	649.08658	519.47072	19
17	101.07094	2016.96716	1008.98722	672.99390	504.99725	404.19925	Q	2464.28190	1232.64459	822.09882	616.82593	493.66220	18
18	88.03930	2131.99410	1066.50069	711.33622	533.75398	427.20464	D	2336.22332	1168.61530	779.41262	584.81129	468.05049	17
19	60.04439	2219.02613	1110.01670	740.34689	555.51199	444.61105	S	2221.19638	1111.10183	741.07031	556.05455	445.04510	16
20	330.27026	2576.28402	1288.64565	859.43286	644.82646	516.06263	K-TMT6plex	2134.16435	1067.58581	712.05963	534.29654	427.63869	15
21	88.03930	2691.31097	1346.15912	897.77517	673.58320	539.06801	D	1776.90645	888.95687	592.97367	444.98207	356.18711	14
22	60.04439	2778.34299	1389.67514	926.78585	695.34121	556.47442	S	1661.87951	831.44339	554.63135	416.22534	333.18172	13
23	74.06004	2879.39067	1440.19897	960.46841	720.60313	576.68396	T	1574.84748	787.92738	525.62068	394.46733	315.77532	12
24	181.06077	3087.43908	1544.22318	1029.81788	772.61523	618.29364	Y-Nitro	1473.79980	737.40354	491.93812	369.20541	295.56578	11
25	60.04439	3174.47111	1587.73919	1058.82855	794.37323	635.70004	S	1265.75140	633.37934	422.58865	317.19331	253.95610	10
26	86.09643	3287.55517	1644.28122	1096.52324	822.64425	658.31686	L	1178.71937	589.86332	393.57797	295.43530	236.54970	9
27	60.04439	3374.58720	1687.79724	1125.53392	844.40226	675.72326	S	1065.63531	533.32129	355.88329	267.16428	213.93288	8
28	60.04439	3461.61923	1731.31325	1154.54459	866.16026	693.12967	S	978.60328	489.80528	326.87261	245.40628	196.52648	7
29	74.06004	3562.66691	1781.83709	1188.22715	891.42218	713.33920	T	891.57125	446.28926	297.86193	223.64827	179.12007	6
30	86.09643	3675.75097	1838.37912	1225.92184	919.69320	735.95602	L	790.52357	395.76542	264.17937	198.38635	158.91054	5
31	74.06004	3776.79865	1888.90296	1259.60440	944.95512	756.16555	T	677.43951	339.22339	226.48469	170.11533	136.29372	4
32	86.09643	3889.88271	1945.44499	1297.29909	973.22614	778.78236	L	576.39183	288.69955	192.80213	144.85341	116.08419	3
33	60.04439	3976.91474	1988.96101	1326.30976	994.98414	796.18877	S	463.30776	232.15752	155.10744	116.58240	93.46737	2
34	330.27026						K-TMT6plex	376.27574	188.64151	126.09676	94.82439	76.06097	1

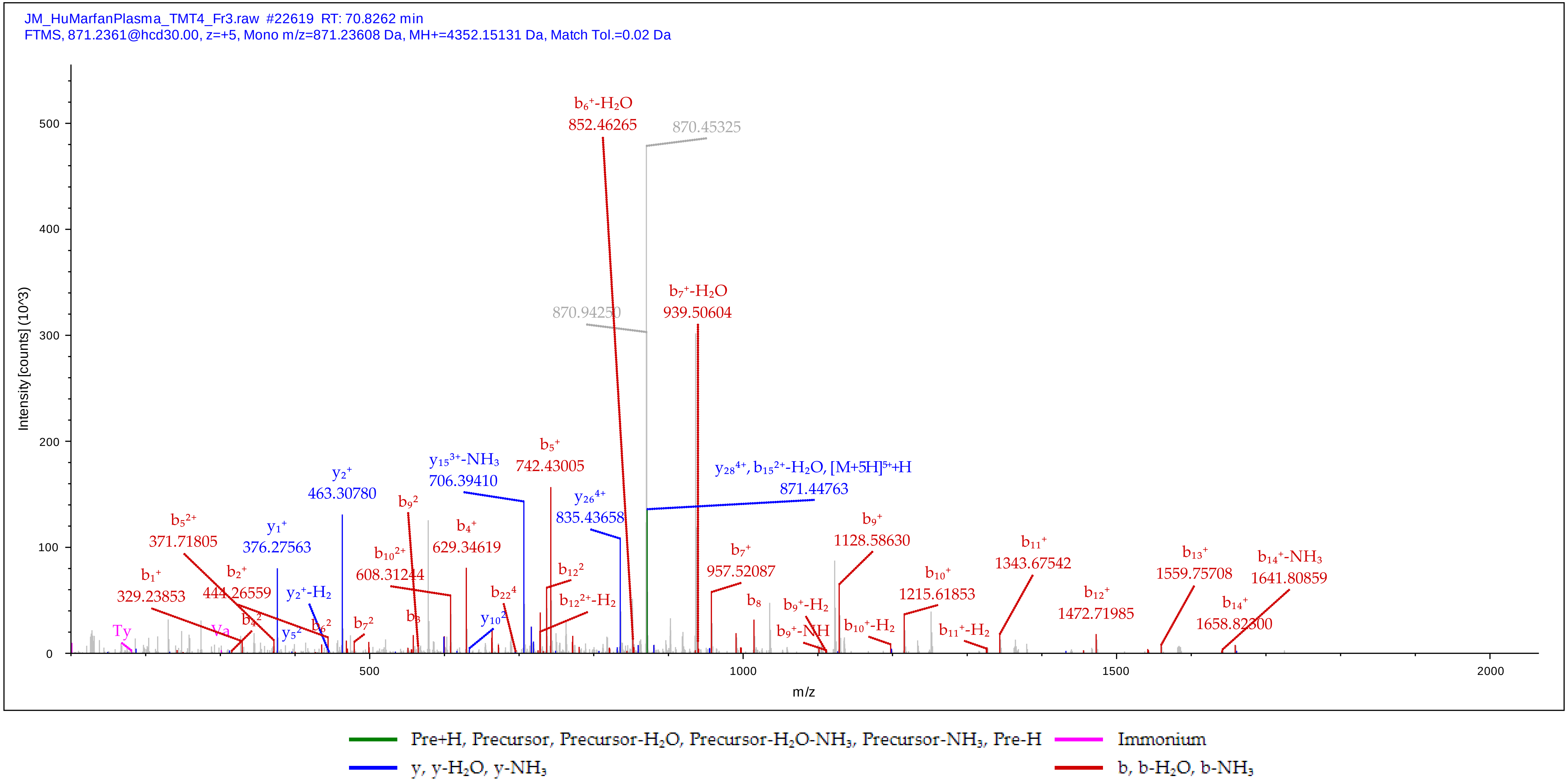
JM_HuMarfanPlasma_TMT4_Fr3.raw #22619 RT: 70.8262 min
FTMS, 871.2361@hcd30.00, z=+5, Mono m/z=871.23608 Da, MH+=4352.15131 Da, Match Tol.=0.02 Da
Intensity [counts] (10^3)
m/z
0
100
200
300
400
500
500
1000
1500
2000
b6+-H2O
852.46265
870.45325
870.94250
b7+-H2O
939.50604
y15 3+-NH3
706.39410
b5+
742.43005
y28 4+, b15 2+-H2O, [M+5H]5+H
871.44763
y2+
463.30780
y26 4+
835.43658
b9+
1128.58630
b5 2+
371.71805
y1+
376.27563
b4+
629.34619
b10 2+
608.31244
b7+
957.52087
b11+
1343.67542
b13+
1559.75708
b14+-NH3
1641.80859
b1+
329.23853
b2+
444.26559
y2+-H2
b12 2
b12 2+-H2
b10+
1215.61853
b12+
1472.71985
b14+
1658.82300
b8
b9+-H2
b9+-NH
b10+-H2
b11+-H2
Ty
Va
y5 2
b4 2
b6 2
b7 2
b3
y10 2
b22 4
b9 2
Pre+H, Precursor, Precursor-H2O, Precursor-H2O-NH3, Precursor-NH3, Pre-H
Immonium
y, y-H2O, y-NH3
b, b-H2O, b-NH3

#1	Immonium	b^+	b^{2+}	b^{3+}	Seq.	y^+	y^{2+}	y^{3+}	#2
1	289.20732	317.20224	159.10476	106.40560	S-TMT6plex				16
2	30.03383	374.22370	187.61549	125.41275	G	1756.83229	878.91979	586.28228	15
3	74.06004	475.27138	238.13933	159.09531	T	1699.81083	850.40905	567.27513	14
4	44.04948	546.30849	273.65788	182.77435	A	1598.76315	799.88521	533.59257	13
5	60.04439	633.34052	317.17390	211.78502	S	1527.72604	764.36666	509.91353	12
6	72.08078	732.40894	366.70811	244.80783	V	1440.69401	720.85064	480.90285	11
7	72.08078	831.47735	416.24231	277.83063	V	1341.62560	671.31644	447.88005	10
8	133.04301	991.50800	496.25764	331.17418	C-Carbamidomethyl	1242.55718	621.78223	414.85725	9
9	86.09643	1104.59206	552.79967	368.86887	L	1082.52653	541.76691	361.51370	8
10	86.09643	1217.67613	609.34170	406.56356	L	969.44247	485.22487	323.81901	7
11	88.03930	1332.70307	666.85517	444.90587	D	856.35841	428.68284	286.12432	6
12	87.05529	1446.74600	723.87664	482.92018	N	741.33146	371.16937	247.78201	5
13	120.08078	1593.81441	797.41084	531.94299	F	627.28854	314.14791	209.76770	4
14	181.06077	1801.86282	901.43505	601.29246	Y-Nitro	480.22012	240.61370	160.74489	3
15	70.06513	1898.91558	949.96143	633.64338	P	272.17172	136.58950	91.39542	2
16	129.11347				R	175.11895	88.06311	59.04450	1

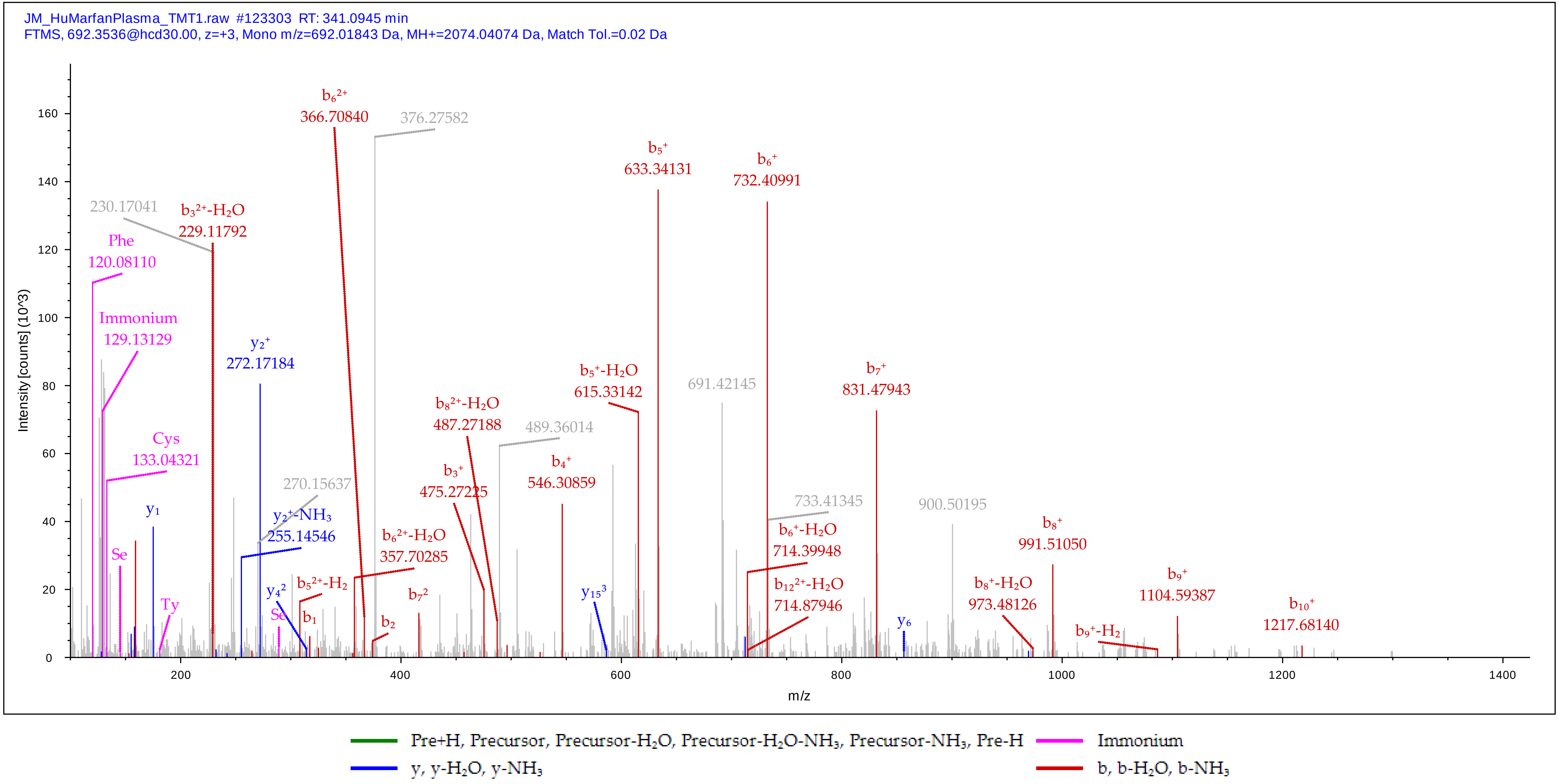

JM_HuMarfanPlasma_TMT1.raw #123303 RT: 341.0945 min
FTMS, 692.3536@hcd30.00, z=+3, Mono m/z=692.01843 Da, MH+=2074.04074 Da, Match Tol.=0.02 Da
Intensity [counts] (10^3)
m/z
0
20
40
60
80
100
120
140
160
200
400
600
800
1000
1200
1400
b_6^{2+} 366.70840
376.27582
b_5^+ 633.34131
b_6^+ 732.40991
230.17041
b_3^{2+}-H_2O 229.11792
Phe 120.08110
Immonium 129.13129
y_2^+ 272.17184
b_5^+-H_2O 615.33142
691.42145
b_7^+ 831.47943
b_8^{2+}-H_2O 487.27188
489.36014
Cys 133.04321
b_3^+ 475.27225
b_4^+ 546.30859
270.15637
y_1
y_2^+-NH_3 255.14546
733.41345
900.50195
b_6^+-H_2O 714.39948
b_8^+ 991.51050
b_6^{2+}-H_2O 357.70285
Se
b_{12}^{2+}-H_2O 714.87946
y_{15}^3
b_8^+-H_2O 973.48126
b_9^+ 1104.59387
y_4^2
b_5^{2+}-H_2
b_7^2
Ty
Se
b_1
b_2
y_6
b_{10}^+ 1217.68140
b_9^+-H_2
Pre+H, Precursor, Precursor-H_2O, Precursor-H_2O-NH_3, Precursor-NH_3, Pre-H
Immonium
y, y-H_2O, y-NH_3
b, b-H_2O, b-NH_3

#1	Immonium	b+	b2+	Seq.	y+	y2+	#2
1	315.25936	343.25427	172.13077	L-TMT6plex			16
2	86.09643	456.33834	228.67281	L	1607.83876	804.42302	15
3	86.09643	569.42240	285.21484	I	1494.75470	747.88099	14
4	181.06077	777.47081	389.23904	Y-Nitro	1381.67063	691.33895	13
5	44.04948	848.50792	424.75760	A	1173.62223	587.31475	12
6	44.04948	919.54503	460.27616	A	1102.58511	551.79619	11
7	60.04439	1006.57706	503.79217	S	1031.54800	516.27764	10
8	74.06004	1107.62474	554.31601	T	944.51597	472.76162	9
9	86.09643	1220.70881	610.85804	L	843.46829	422.23778	8
10	101.07094	1348.76738	674.88733	Q	730.38423	365.69575	7
11	60.04439	1435.79941	718.40334	S	602.32565	301.66646	6
12	30.03383	1492.82087	746.91408	G	515.29362	258.15045	5
13	72.08078	1591.88929	796.44828	V	458.27216	229.63972	4
14	70.06513	1688.94205	844.97466	P	359.20374	180.10551	3
15	60.04439	1775.97408	888.49068	S	262.15098	131.57913	2
16	129.11347			R	175.11895	88.06311	1

Nitro-Tyr immonium ion is detected in MS/MS spectra and added brown colored in the following spectrum

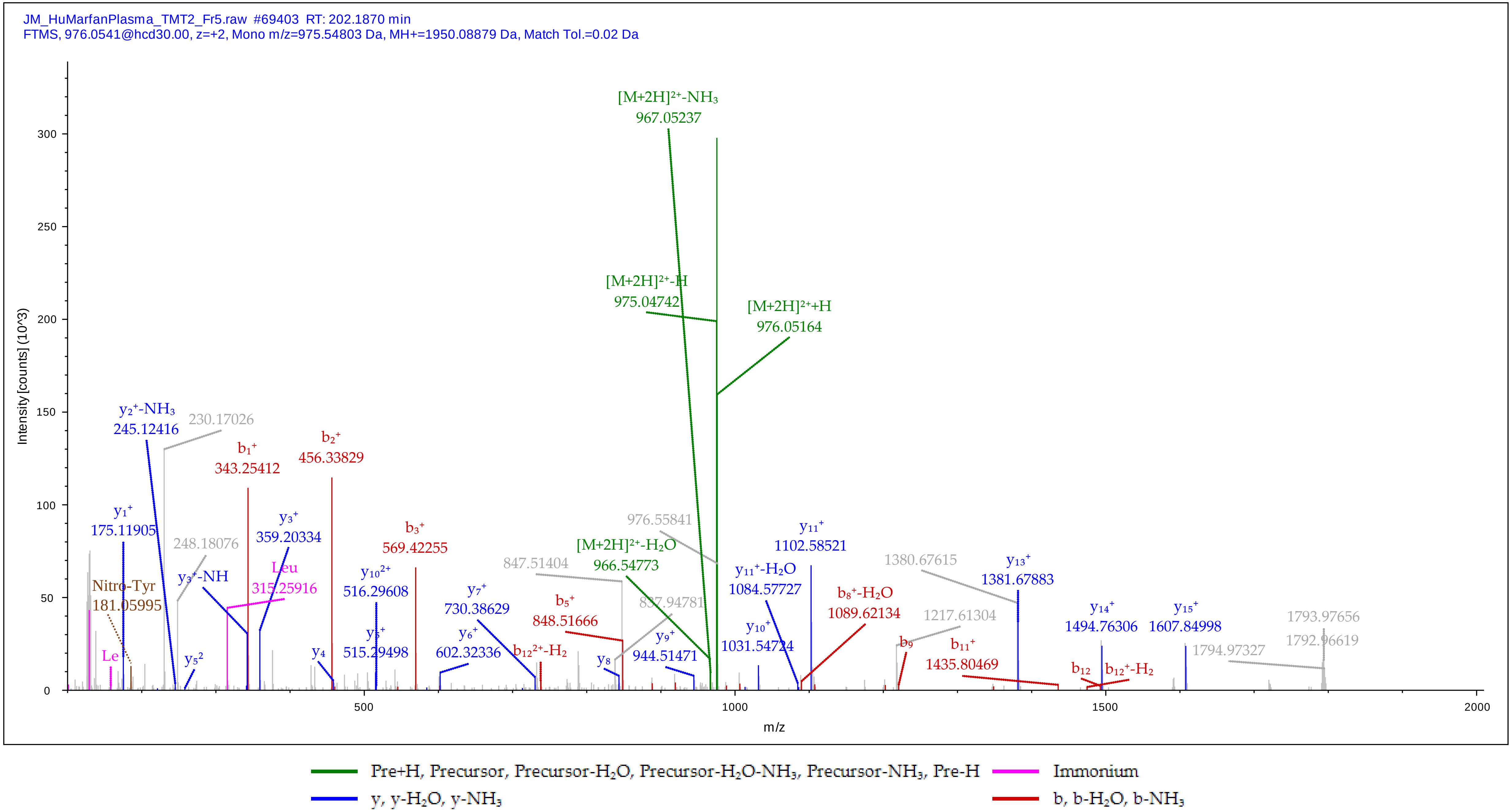
JM_HuMarfanPlasma_TMT2_Fr5.raw #69403 RT: 202.1870 min
FTMS, 976.0541@hcd30.00, z=+2, Mono m/z=975.54803 Da, MH+=1950.08879 Da, Match Tol.=0.02 Da
Intensity [counts] (10^3)
m/z
[M+2H]2+-NH3 967.05237
[M+2H]2+-H 975.04742
[M+2H]2+ +H 976.05164
[M+2H]2+-H2O 966.54773
y2+-NH3 245.12416
230.17026
b1+ 343.25412
b2+ 456.33829
y1+ 175.11905
248.18076
y3+ 359.20334
b3+ 569.42255
Leu 315.25916
Nitro-Tyr 181.05995
y3+-NH
y10 2+ 516.29608
y5+ 515.29498
y7+ 730.38629
y6+ 602.32336
847.51404
b5+ 848.51666
b12 2+-H2
976.55841
y9+ 944.51471
y11+ 1102.58521
y11+-H2O 1084.57727
y10+ 1031.54724
b8+-H2O 1089.62134
1380.67615
y13+ 1381.67883
1217.61304
b11+ 1435.80469
b12
b12+-H2
y14+ 1494.76306
y15+ 1607.84998
1793.97656
1792.96619
1794.97327
Pre+H, Precursor, Precursor-H2O, Precursor-H2O-NH3, Precursor-NH3, Pre-H
Immonium
y, y-H2O, y-NH3
b, b-H2O, b-NH3

#1	Immonium	b+	b2+	b3+	b4+	Seq.	y+	y2+	y3+	y4+	#2
1	303.22297	331.21789	166.11258	111.07748	83.55993	T-TMT6plex					16
2	136.07569	494.28122	247.64425	165.43192	124.32576	Y	2439.28614	1220.14671	813.76690	610.57699	15
3	86.09643	607.36528	304.18628	203.12661	152.59678	I	2276.22281	1138.61504	759.41245	569.81116	14
4	120.08078	754.43369	377.72048	252.14942	189.36388	F	2163.13874	1082.07301	721.71777	541.54014	13
5	44.04948	825.47081	413.23904	275.82845	207.12316	A	2016.07033	1008.53880	672.69496	504.77304	12
6	30.03383	882.49227	441.74977	294.83561	221.37853	G	1945.03322	973.02025	649.01592	487.01376	11
7	88.03930	997.51921	499.26325	333.17792	250.13526	D	1888.01175	944.50951	630.00877	472.75840	10
8	330.27026	1354.77711	677.89219	452.26389	339.44973	K-TMT6plex	1772.98481	886.99604	591.66645	444.00166	9
9	120.08078	1501.84552	751.42640	501.28669	376.21684	F	1415.72691	708.36710	472.58049	354.68719	8
10	159.09167	1687.92484	844.46606	563.31313	422.73667	W	1268.65850	634.83289	423.55768	317.92008	7
11	129.11347	1844.02595	922.51661	615.34683	461.76194	R	1082.57919	541.79323	361.53125	271.40025	6
12	181.06077	2052.07435	1026.54081	684.69630	513.77405	Y-Nitro	926.47808	463.74268	309.49754	232.37498	5
13	87.05529	2166.11728	1083.56228	722.71061	542.28478	N	718.42967	359.71847	240.14807	180.36288	4
14	102.05496	2295.15987	1148.08357	765.72481	574.54543	E	604.38674	302.69701	202.13377	151.85214	3
15	72.08078	2394.22829	1197.61778	798.74761	599.31253	V	475.34415	238.17571	159.11957	119.59150	2
16	330.27026					K-TMT6plex	376.27574	188.64151	126.09676	94.82439	1

Nitro-Tyr immonium ion is detected in MS/MS spectra and added brown colored in the following spectrum

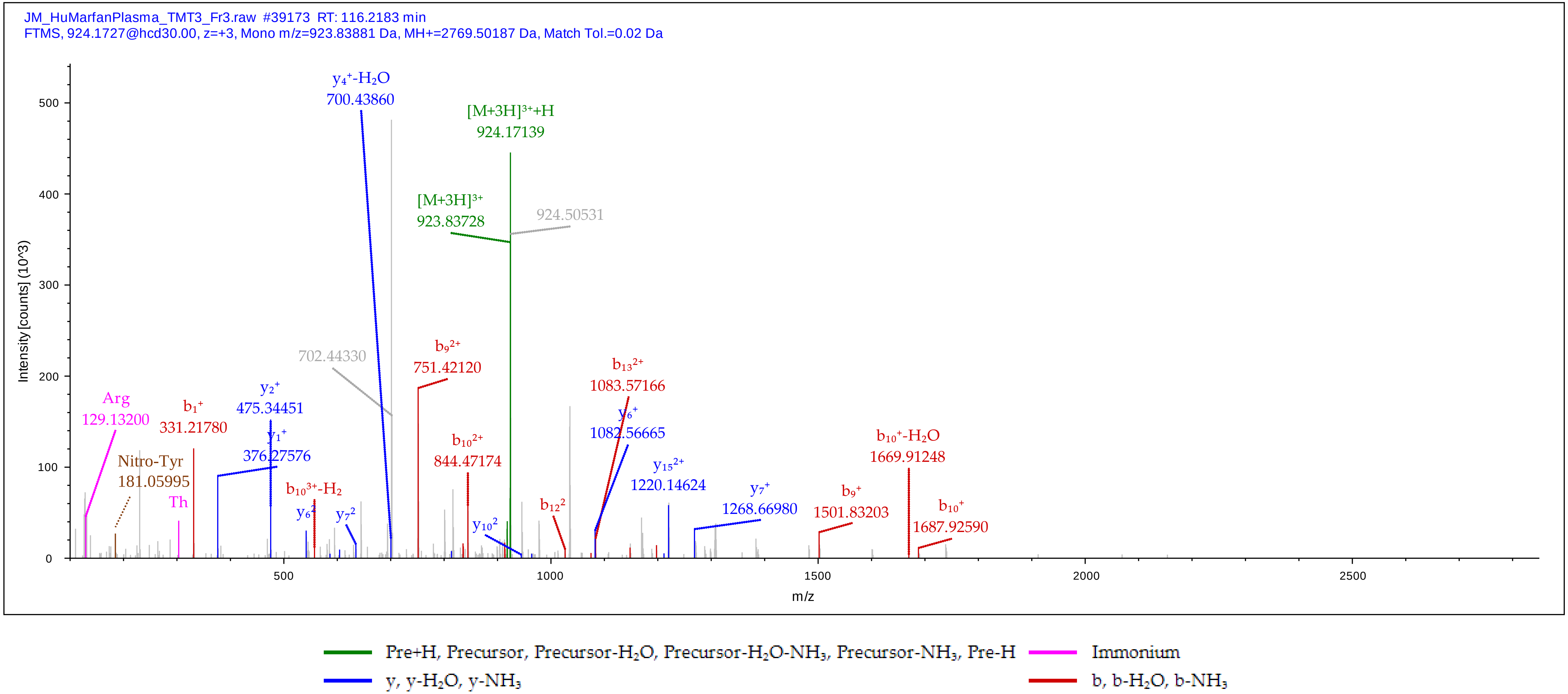
JM_HuMarfanPlasma_TMT3_Fr3.raw #39173 RT: 116.2183 min
FTMS, 924.1727@hcd30.00, z=+3, Mono m/z=923.83881 Da, MH+=2769.50187 Da, Match Tol.=0.02 Da
Intensity [counts] (10^3)
m/z
0
100
200
300
400
500
1000
1500
2000
2500
y4+-H2O 700.43860
[M+3H]3++H 924.17139
[M+3H]3+ 923.83728
924.50531
702.44330
b92+ 751.42120
b132+ 1083.57166
y6+ 1082.56665
y2+ 475.34451
Arg 129.13200
b1+ 331.21780
y1+ 376.27576
b102+ 844.47174
b103+-H2
Nitro-Tyr 181.05995
Th
y62
y72
y102
b122
y152+ 1220.14624
y7+ 1268.66980
b9+ 1501.83203
b10+-H2O 1669.91248
b10+ 1687.92590
Pre+H, Precursor, Precursor-H2O, Precursor-H2O-NH3, Precursor-NH3, Pre-H
Immonium
y, y-H2O, y-NH3
b, b-H2O, b-NH3

#1	Immonium	b^{+}	b^{2+}	Seq.	y^{+}	y^{2+}	#2
1	289.20732	317.20224	159.10476	S-TMT6plex			11
2	44.04948	388.23935	194.62331	A	1436.72189	718.86458	10
3	60.04439	475.27138	238.13933	S	1365.68478	683.34603	9
4	88.03930	590.29832	295.65280	D	1278.65275	639.83001	8
5	86.09643	703.38239	352.19483	L	1163.62580	582.31654	7
6	74.06004	804.43006	402.71867	T	1050.54174	525.77451	6
7	204.07675	1035.49446	518.25087	W-Nitro	949.49406	475.25067	5
8	88.03930	1150.52140	575.76434	D	718.42967	359.71847	4
9	87.05529	1264.56433	632.78580	N	603.40273	302.20500	3
10	86.09643	1377.64839	689.32783	L	489.35980	245.18354	2
11	330.27026			K-TMT6plex	376.27574	188.64151	1

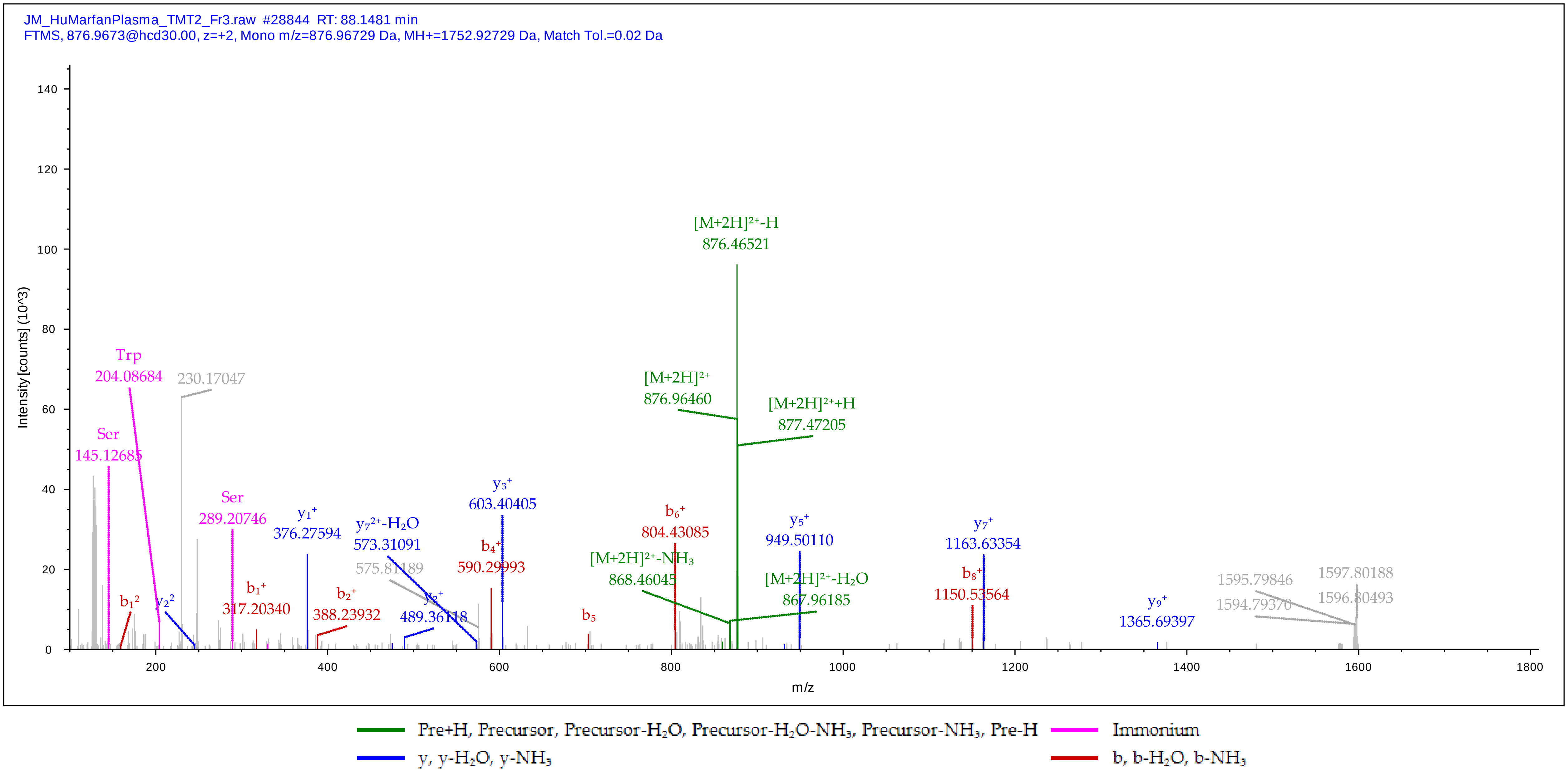
JM_HuMarfanPlasma_TMT2_Fr3.raw #28844 RT: 88.1481 min
FTMS, 876.9673@hcd30.00, z=+2, Mono m/z=876.96729 Da, MH+=1752.92729 Da, Match Tol.=0.02 Da
Intensity [counts] (10^3)
m/z
[M+2H]²⁺-H
876.46521
[M+2H]²⁺
876.96460
[M+2H]²⁺+H
877.47205
Trp
204.08684
230.17047
Ser
145.12685
Ser
289.20746
y₁⁺
376.27594
y₇²⁺-H₂O
573.31091
575.81189
y₃⁺
603.40405
b₄⁺
590.29993
b₆⁺
804.43085
[M+2H]²⁺-NH₃
868.46045
y₅⁺
949.50110
[M+2H]²⁺-H₂O
867.96185
y₇⁺
1163.63354
b₈⁺
1150.53564
b₁²
y₂²
b₁⁺
317.20340
b₂⁺
388.23932
y₂⁺
489.36118
b₅
y₉⁺
1365.69397
1595.79846
1594.79370
1597.80188
1596.80493
Pre+H, Precursor, Precursor-H₂O, Precursor-H₂O-NH₃, Precursor-NH₃, Pre-H
Immonium
y, y-H₂O, y-NH₃
b, b-H₂O, b-NH₃

#1	Immonium	b+	b2+	b3+	Seq.	y+	y2+	y3+	#2
1	289.20732	317.20224	159.10476	106.40560	S-TMT6plex				19
2	44.04948	388.23935	194.62331	130.08463	A	2129.04844	1065.02786	710.35433	18
3	30.03383	445.26081	223.13405	149.09179	G	2058.01132	1029.50930	686.67529	17
4	204.07675	676.32521	338.66624	226.11325	W-Nitro	2000.98986	1000.99857	667.66814	16
5	87.05529	790.36813	395.68770	264.12756	N	1769.92547	885.46637	590.64667	15
6	86.09643	903.45220	452.22974	301.82225	I	1655.88254	828.44491	552.63236	14
7	70.06513	1000.50496	500.75612	334.17317	P	1542.79848	771.90288	514.93768	13
8	86.09643	1113.58902	557.29815	371.86786	I	1445.74571	723.37649	482.58676	12
9	30.03383	1170.61049	585.80888	390.87501	G	1332.66165	666.83446	444.89207	11
10	86.09643	1283.69455	642.35091	428.56970	L	1275.64018	638.32373	425.88491	10
11	86.09643	1396.77862	698.89295	466.26439	L	1162.55612	581.78170	388.19022	9
12	136.07569	1559.84194	780.42461	520.61883	Y	1049.47206	525.23967	350.49554	8
13	133.04301	1719.87259	860.43993	573.96238	C-Carbamidomethyl	886.40873	443.70800	296.14109	7
14	88.03930	1834.89954	917.95341	612.30470	D	726.37808	363.69268	242.79754	6
15	86.09643	1947.98360	974.49544	649.99938	L	611.35114	306.17921	204.45523	5
16	70.06513	2045.03636	1023.02182	682.35031	P	498.26707	249.63717	166.76054	4
17	102.05496	2174.07896	1087.54312	725.36450	E	401.21431	201.11079	134.40962	3
18	70.06513	2271.13172	1136.06950	757.71542	P	272.17172	136.58950	91.39542	2
19	129.11347				R	175.11895	88.06311	59.04450	1

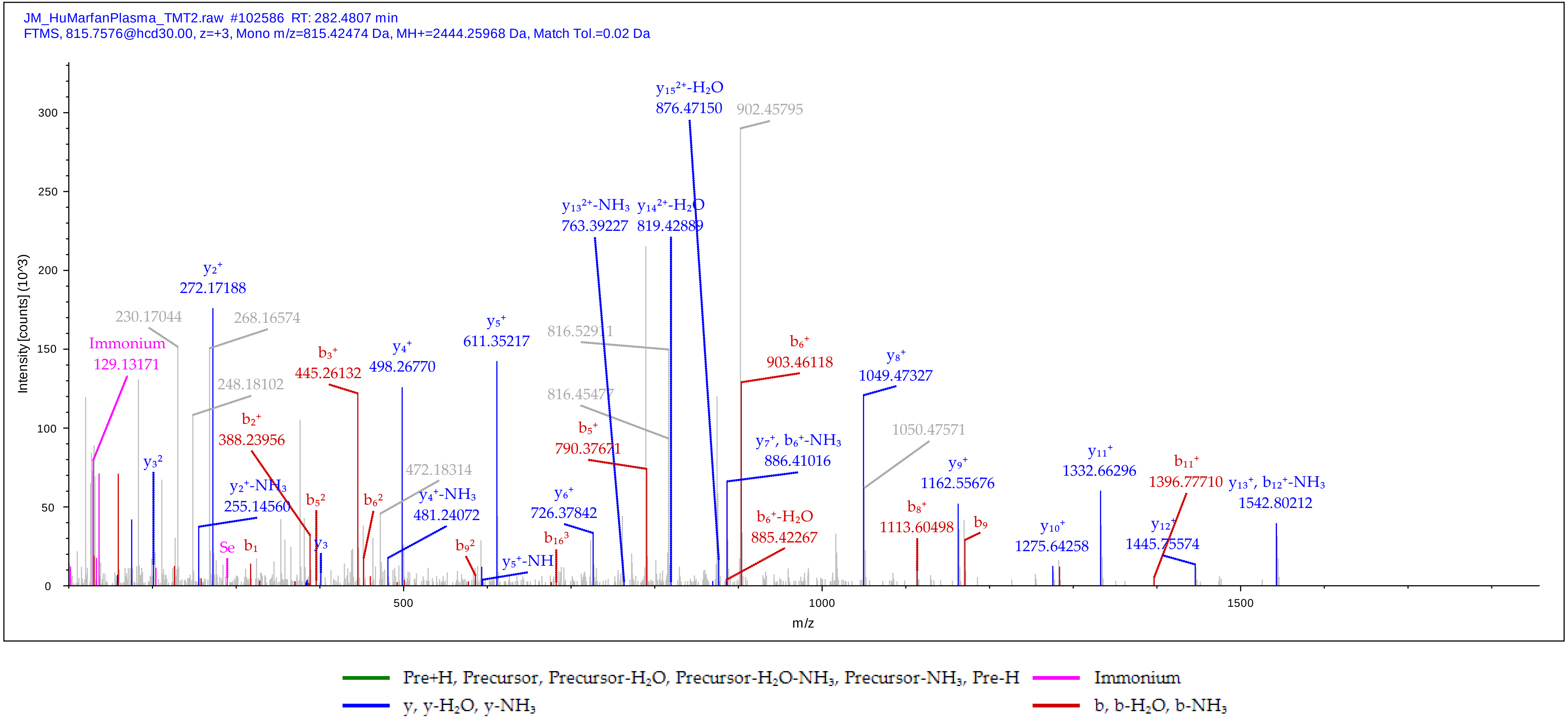

JM_HuMarfanPlasma_TMT2.raw #102586 RT: 282.4807 min
FTMS, 815.7576@hcd30.00, z=+3, Mono m/z=815.42474 Da, MH+=2444.25968 Da, Match Tol.=0.02 Da
Intensity [counts] (10^3)
m/z
0
50
100
150
200
250
300
500
1000
1500
Immonium
129.13171
230.17044
268.16574
248.18102
y2+
272.17188
y3 2
y2+-NH3
255.14560
Se
b1
b2+
388.23956
b3+
445.26132
b5 2
y3
b6 2
y4+
498.26770
472.18314
y4+-NH3
481.24072
y5+
611.35217
b9 2
y5+-NH
y6+
726.37842
b16 3
816.52911
816.45477
b5+
790.37671
y13 2+-NH3
763.39227
y14 2+-H2O
819.42889
y15 2+-H2O
876.47150
902.45795
b6+
903.46118
y7+, b6+-NH3
886.41016
b6+-H2O
885.42267
y8+
1049.47327
1050.47571
b8+
1113.60498
y9+
1162.55676
b9
y10+
1275.64258
y11+
1332.66296
b11+
1396.77710
y12+
1445.75574
y13+, b12+-NH3
1542.80212
Pre+H, Precursor, Precursor-H2O, Precursor-H2O-NH3, Precursor-NH3, Pre-H
Immonium
y, y-H2O, y-NH3
b, b-H2O, b-NH3

#1	Immonium	b^{+}	b^{2+}	b^{3+}	Seq.	y^{+}	y^{2+}	y^{3+}	#2
1	316.21822	344.21314	172.61021	115.40923	N-TMT6plex				16
2	86.09643	457.29720	229.15224	153.10392	L	2113.04330	1057.02529	705.01928	15
3	87.05529	571.34013	286.17370	191.11823	N	1999.95924	1000.48326	667.32460	14
4	102.05496	700.38272	350.69500	234.13242	E	1885.91631	943.46179	629.31029	13
5	330.27026	1057.64062	529.32395	353.21839	K-TMT6plex	1756.87371	878.94050	586.29609	12
6	88.03930	1172.66756	586.83742	391.56070	D	1399.61582	700.31155	467.21012	11
7	181.06077	1380.71596	690.86162	460.91017	Y-Nitro	1284.58888	642.79808	428.86781	10
8	102.05496	1509.75856	755.38292	503.92437	E	1076.54047	538.77387	359.51834	9
9	86.09643	1622.84262	811.92495	541.61906	L	947.49788	474.25258	316.50414	8
10	86.09643	1735.92669	868.46698	579.31375	L	834.41381	417.71055	278.80946	7
11	133.04301	1895.95733	948.48231	632.65730	C-Carbamidomethyl	721.32975	361.16851	241.11477	6
12	86.09643	2009.04140	1005.02434	670.35198	L	561.29910	281.15319	187.77122	5
13	88.03930	2124.06834	1062.53781	708.69430	D	448.21504	224.61116	150.07653	4
14	30.03383	2181.08980	1091.04854	727.70145	G	333.18809	167.09769	111.73422	3
15	74.06004	2282.13748	1141.57238	761.38401	T	276.16663	138.58695	92.72706	2
16	129.11347				R	175.11895	88.06311	59.04450	1

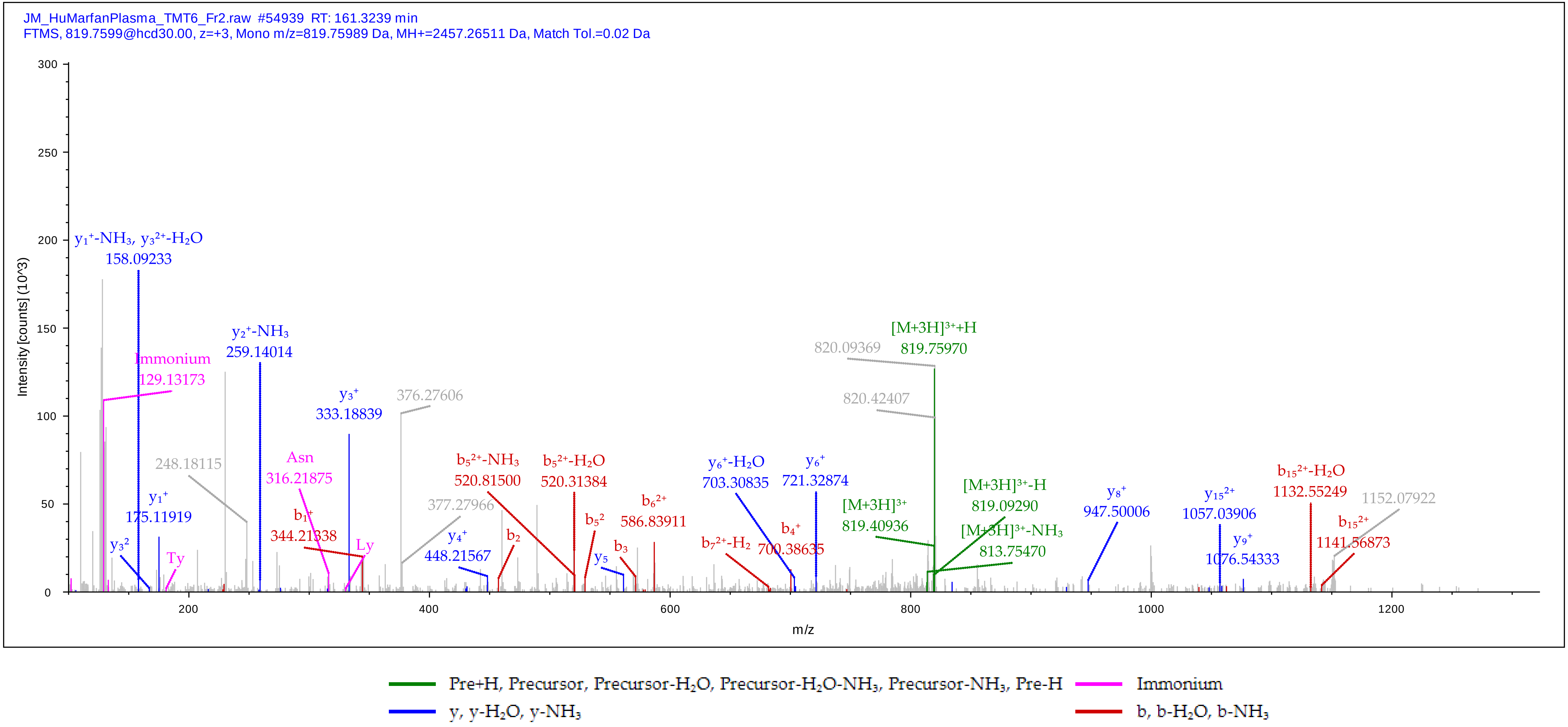

JM_HuMarfanPlasma_TMT6_Fr2.raw #54939 RT: 161.3239 min
FTMS, 819.7599@hcd30.00, z=+3, Mono m/z=819.75989 Da, MH+=2457.26511 Da, Match Tol.=0.02 Da
Intensity [counts] (10^3)
m/z
y_1^+-NH_3, y_3^{2+}-H_2O 158.09233
Immonium 129.13173
y_2^+-NH_3 259.14014
y_3^+ 333.18839
376.27606
248.18115
Asn 316.21875
y_1^+ 175.11919
b_1^+ 344.21338
y_3^2
Ty
Ly
377.27966
y_4^+ 448.21567
b_2
b_5^{2+}-NH_3 520.81500
b_5^{2+}-H_2O 520.31384
b_5^2
b_6^{2+} 586.83911
b_3
y_5
y_6^+-H_2O 703.30835
y_6^+ 721.32874
b_7^{2+}-H_2
b_4^+ 700.38635
820.09369
820.42407
$[M+3H]^{3+}$+H 819.75970
$[M+3H]^{3+}$ 819.40936
$[M+3H]^{3+}$-H 819.09290
$[M+3H]^{3+}$-NH_3 813.75470
y_8^+ 947.50006
y_{15}^{2+} 1057.03906
y_9^+ 1076.54333
b_{15}^{2+}-H_2O 1132.55249
b_{15}^{2+} 1141.56873
1152.07922
Pre+H, Precursor, Precursor-H_2O, Precursor-H_2O-NH_3, Precursor-NH_3, Pre-H
Immonium
y, y-H_2O, y-NH_3
b, b-H_2O, b-NH_3

#1	Immonium	b^{+}	b^{2+}	b^{3+}	b^{4+}	b^{5+}	Seq.	y^{+}	y^{2+}	y^{3+}	y^{4+}	y^{5+}	#2
1	559.43319	587.42810	294.21769	196.48089	147.61248	118.29144	K-TMT6plex-TMT6plex						22
2	70.06513	684.48087	342.74407	228.83181	171.87567	137.70199	P	2695.34596	1348.17662	899.12017	674.59195	539.87501	21
3	72.08078	783.54928	392.27828	261.85461	196.64278	157.51568	V	2598.29320	1299.65024	866.76925	650.32876	520.46446	20
4	88.03930	898.57622	449.79175	300.19693	225.39951	180.52107	D	2499.22479	1250.11603	833.74645	625.56165	500.65078	19
5	102.05496	1027.61882	514.31305	343.21112	257.66016	206.32958	E	2384.19784	1192.60256	795.40413	596.80492	477.64539	18
6	181.06077	1235.66722	618.33725	412.56059	309.67226	247.93927	Y-Nitro	2255.15525	1128.08126	752.38993	564.54427	451.83687	17
7	330.27026	1592.92512	796.96620	531.64656	398.98674	319.39085	K-TMT6plex	2047.10684	1024.05706	683.04047	512.53217	410.22719	16
8	88.03930	1707.95206	854.47967	569.98887	427.74347	342.39623	D	1689.84895	845.42811	563.95450	423.21769	338.77561	15
9	133.04301	1867.98271	934.49499	623.33242	467.75113	374.40236	C-Carbamidomethyl	1574.82201	787.91464	525.61219	394.46096	315.77022	14
10	110.07127	2005.04162	1003.02445	669.01873	502.01586	401.81415	H	1414.79136	707.89932	472.26864	354.45330	283.76409	13
11	86.09643	2118.12569	1059.56648	706.71341	530.28688	424.43096	L	1277.73245	639.36986	426.58233	320.18857	256.35231	12
12	44.04948	2189.16280	1095.08504	730.39245	548.04616	438.63838	A	1164.64838	582.82783	388.88764	291.91755	233.73550	11
13	101.07094	2317.22138	1159.11433	773.07864	580.06080	464.25010	Q	1093.61127	547.30927	365.20861	274.15827	219.52807	10
14	72.08078	2416.28979	1208.64853	806.10145	604.82791	484.06378	V	965.55269	483.27998	322.52241	242.14363	193.91636	9
15	70.06513	2513.34255	1257.17492	838.45237	629.09110	503.47433	P	866.48428	433.74578	289.49961	217.37653	174.10268	8
16	60.04439	2600.37458	1300.69093	867.46305	650.84910	520.88074	S	769.43151	385.21939	257.14869	193.11334	154.69212	7
17	110.07127	2737.43349	1369.22039	913.14935	685.11383	548.29252	H	682.39948	341.70338	228.13801	171.35533	137.28572	6
18	74.06004	2838.48117	1419.74422	946.83191	710.37575	568.50206	T	545.34057	273.17392	182.45171	137.09060	109.87394	5
19	72.08078	2937.54959	1469.27843	979.85471	735.14285	588.31574	V	444.29289	222.65009	148.76915	111.82868	89.66440	4
20	72.08078	3036.61800	1518.81264	1012.87752	759.90996	608.12942	V	345.22448	173.11588	115.74634	87.06158	69.85072	3
21	44.04948	3107.65511	1554.33120	1036.55656	777.66924	622.33684	A	246.15607	123.58167	82.72354	62.29447	50.03703	2
22	129.11347						R	175.11895	88.06311	59.04450	44.53520	35.82961	1

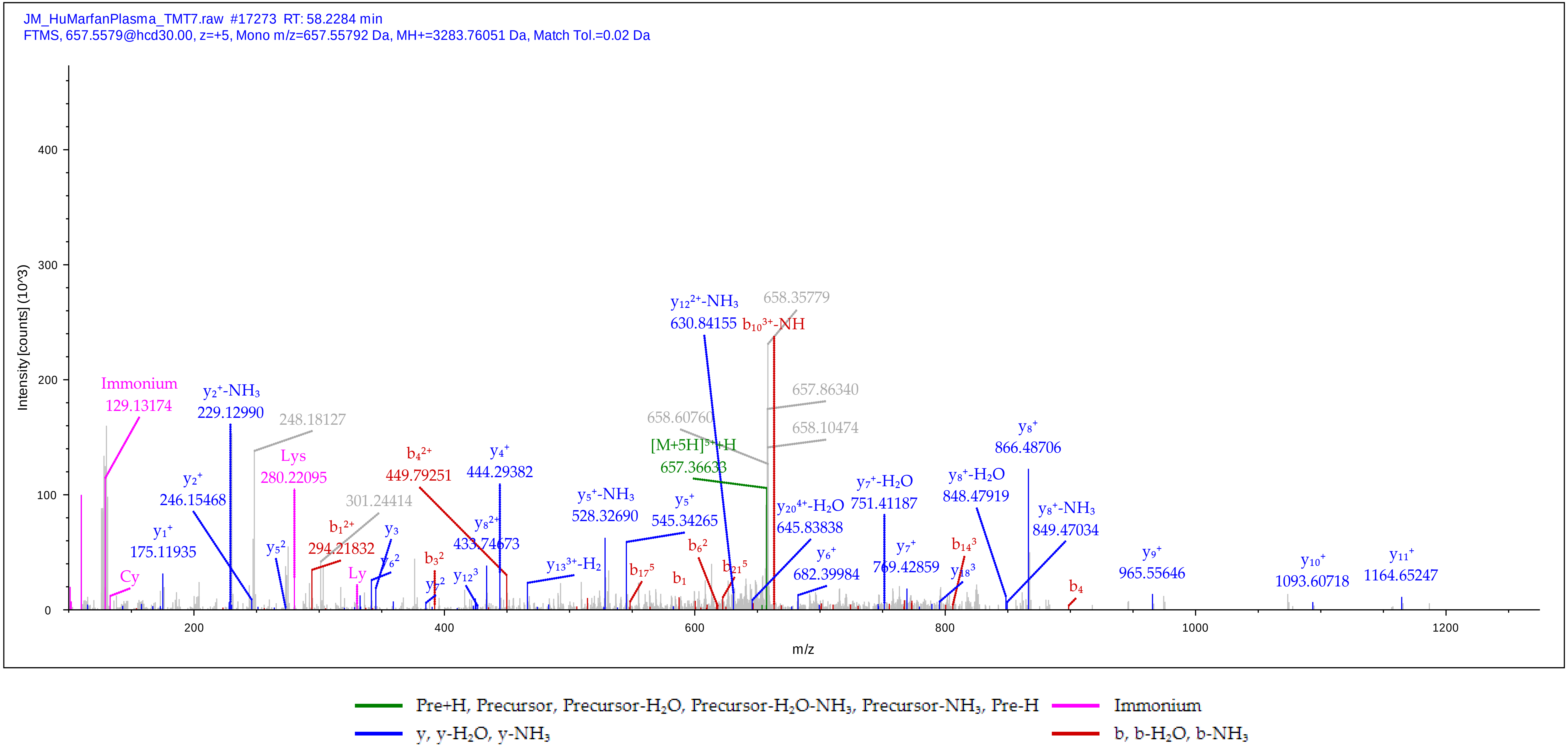
JM_HuMarfanPlasma_TMT7.raw #17273 RT: 58.2284 min
FTMS, 657.5579@hcd30.00, z=+5, Mono m/z=657.55792 Da, MH+=3283.76051 Da, Match Tol.=0.02 Da
Intensity [counts] (10^3)
400
300
200
100
0
200
400
600
800
1000
1200
m/z
Immonium 129.13174
Cy
y_1^+ 175.11935
y_2^+-NH_3 229.12990
y_2^+ 246.15468
248.18127
y_5^2
Lys 280.22095
b_1^{2+} 294.21832
301.24414
Ly
y_3
y_6^2
b_3^2
y_7^2
y_{12}^3
b_4^{2+} 449.79251
y_8^{2+} 433.74673
y_4^+ 444.29382
y_{13}^{3+}-H_2
y_5^+-NH_3 528.32690
y_5^+ 545.34265
b_{17}^5
b_1
b_6^2
b_{21}^5
y_{12}^{2+}-NH_3 630.84155
658.35779
b_{10}^{3+}-NH
658.60760
$[M+5H]^{5+}$+H 657.36633
657.86340
658.10474
y_{20}^{4+}-H_2O 645.83838
y_6^+ 682.39984
y_7^+-H_2O 751.41187
y_7^+ 769.42859
y_{18}^3
b_{14}^3
y_8^+-H_2O 848.47919
y_8^+ 866.48706
y_8^+-NH_3 849.47034
b_4
y_9^+ 965.55646
y_{10}^+ 1093.60718
y_{11}^+ 1164.65247
Pre+H, Precursor, Precursor-H_2O, Precursor-H_2O-NH_3, Precursor-NH_3, Pre-H
Immonium
y, y-H_2O, y-NH_3
b, b-H_2O, b-NH_3

#1	Immonium	b^{+}	b^{2+}	b^{3+}	b^{4+}	Seq.	y^{+}	y^{2+}	y^{3+}	y^{4+}	#2
1	289.20732	317.20224	159.10476	106.40560	80.05602	S-TMT6plex					22
2	104.05285	448.24272	224.62500	150.08576	112.81614	M	2946.51840	1473.76284	982.84432	737.38506	21
3	30.03383	505.26419	253.13573	169.09291	127.07150	G	2815.47791	1408.24259	939.16416	704.62494	20
4	30.03383	562.28565	281.64646	188.10007	141.32687	G	2758.45645	1379.73186	920.15700	690.36957	19
5	330.27026	919.54354	460.27541	307.18603	230.64134	K-TMT6plex	2701.43499	1351.22113	901.14985	676.11420	18
6	102.05496	1048.58614	524.79671	350.20023	262.90199	E	2344.17709	1172.59218	782.06388	586.79973	17
7	88.03930	1163.61308	582.31018	388.54254	291.65873	D	2215.13450	1108.07089	739.04968	554.53908	16
8	86.09643	1276.69714	638.85221	426.23723	319.92974	L	2100.10755	1050.55742	700.70737	525.78235	15
9	86.09643	1389.78121	695.39424	463.93192	348.20076	I	1987.02349	994.01538	663.01268	497.51133	14
10	204.07675	1620.84560	810.92644	540.95338	405.96686	W-Nitro	1873.93943	937.47335	625.31799	469.24031	13
11	102.05496	1749.88819	875.44773	583.96758	438.22751	E	1642.87504	821.94116	548.29653	411.47422	12
12	86.09643	1862.97226	931.98977	621.66227	466.49852	L	1513.83244	757.41986	505.28233	379.21357	11
13	86.09643	1976.05632	988.53180	659.35696	494.76954	L	1400.74838	700.87783	467.58764	350.94255	10
14	87.05529	2090.09925	1045.55326	697.37127	523.28027	N	1287.66431	644.33580	429.89296	322.67154	9
15	101.07094	2218.15782	1109.58255	740.05746	555.29491	Q	1173.62139	587.31433	391.87865	294.16080	8
16	44.04948	2289.19494	1145.10111	763.73650	573.05419	A	1045.56281	523.28504	349.19245	262.14616	7
17	101.07094	2417.25352	1209.13040	806.42269	605.06884	Q	974.52570	487.76649	325.51342	244.38688	6
18	102.05496	2546.29611	1273.65169	849.43689	637.32948	E	846.46712	423.73720	282.82722	212.37224	5
19	110.07127	2683.35502	1342.18115	895.12319	671.59421	H	717.42453	359.21590	239.81303	180.11159	4
20	120.08078	2830.42343	1415.71536	944.14600	708.36132	F	580.36561	290.68645	194.12672	145.84686	3
21	30.03383	2887.44490	1444.22609	963.15315	722.61668	G	433.29720	217.15224	145.10392	109.07976	2
22	330.27026					K-TMT6plex	376.27574	188.64151	126.09676	94.82439	1

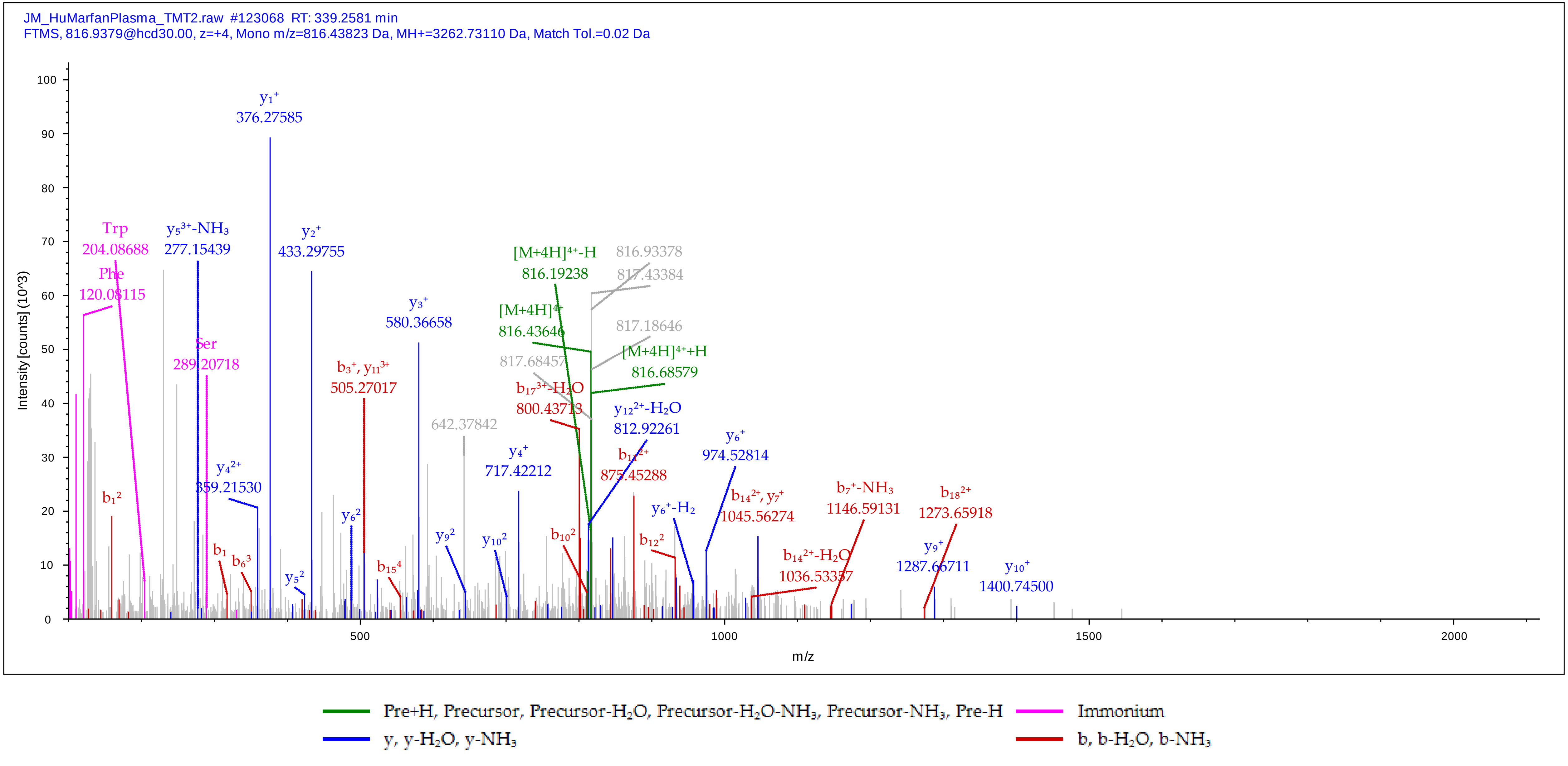

JM_HuMarfanPlasma_TMT2.raw #123068 RT: 339.2581 min
FTMS, 816.9379@hcd30.00, z=+4, Mono m/z=816.43823 Da, MH+=3262.73110 Da, Match Tol.=0.02 Da
Intensity [counts] (10^3)
m/z
y1+ 376.27585
Trp 204.08688
Phe 120.08115
y5 3+-NH3 277.15439
y2+ 433.29755
Ser 289.20718
y3+ 580.36658
b3+, y11 3+ 505.27017
[M+4H]4+-H 816.19238
[M+4H]4+ 816.43646
[M+4H]4++H 816.68579
816.93378
817.43384
817.18646
817.68457
b17 3+-H2O 800.43713
642.37842
y12 2+-H2O 812.92261
y4+ 717.42212
y4 2+ 359.21530
b11 2+ 875.45288
y6+ 974.52814
b14 2+, y7+ 1045.56274
b7+-NH3 1146.59131
b18 2+ 1273.65918
y6+-H2
b14 2+-H2O 1036.53357
y9+ 1287.66711
y10+ 1400.74500
Pre+H, Precursor, Precursor-H2O, Precursor-H2O-NH3, Precursor-NH3, Pre-H
Immonium
y, y-H2O, y-NH3
b, b-H2O, b-NH3

#1	Immonium	b⁺	b²⁺	b³⁺	b⁴⁺	Seq.	y⁺	y²⁺	y³⁺	y⁴⁺	#2
1	273.21241	301.20732	151.10730	101.07396	76.05729	A-TMT6plex					20
2	88.03930	416.23427	208.62077	139.41627	104.81402	D	2274.14914	1137.57821	758.72123	569.29274	19
3	44.04948	487.27138	244.13933	163.09531	122.57330	A	2159.12220	1080.06474	720.37892	540.53601	18
4	72.08078	586.33979	293.67353	196.11812	147.34041	V	2088.08509	1044.54618	696.69988	522.77673	17
5	74.06004	687.38747	344.19737	229.80067	172.60233	T	1989.01667	995.01198	663.67708	498.00963	16
6	86.09643	800.47154	400.73941	267.49536	200.87334	L	1887.96900	944.48814	629.99452	472.74771	15
7	88.03930	915.49848	458.25288	305.83768	229.63008	D	1774.88493	887.94610	592.29983	444.47669	14
8	30.03383	972.51994	486.76361	324.84483	243.88544	G	1659.85799	830.43263	553.95751	415.71995	13
9	30.03383	1029.54141	515.27434	343.85199	258.14081	G	1602.83652	801.92190	534.95036	401.46459	12
10	120.08078	1176.60982	588.80855	392.87479	294.90791	F	1545.81506	773.41117	515.94320	387.20922	11
11	86.09643	1289.69388	645.35058	430.56948	323.17893	I	1398.74665	699.87696	466.92040	350.44212	10
12	181.06077	1497.74229	749.37478	499.91895	375.19103	Y-Nitro	1285.66258	643.33493	429.22571	322.17110	9
13	102.05496	1626.78488	813.89608	542.93315	407.45168	E	1077.61418	539.31073	359.87624	270.15900	8
14	44.04948	1697.82200	849.41464	566.61218	425.21096	A	948.57158	474.78943	316.86205	237.89835	7
15	30.03383	1754.84346	877.92537	585.61934	439.46632	G	877.53447	439.27087	293.18301	220.13907	6
16	86.09643	1867.92752	934.46740	623.31403	467.73734	L	820.51301	410.76014	274.17585	205.88371	5
17	44.04948	1938.96464	969.98596	646.99306	485.49662	A	707.42894	354.21811	236.48117	177.61269	4
18	70.06513	2036.01740	1018.51234	679.34399	509.75981	P	636.39183	318.69955	212.80213	159.85341	3
19	136.07569	2199.08073	1100.04400	733.69843	550.52564	Y	539.33906	270.17317	180.45121	135.59022	2
20	330.27026					K-TMT6plex	376.27574	188.64151	126.09676	94.82439	1

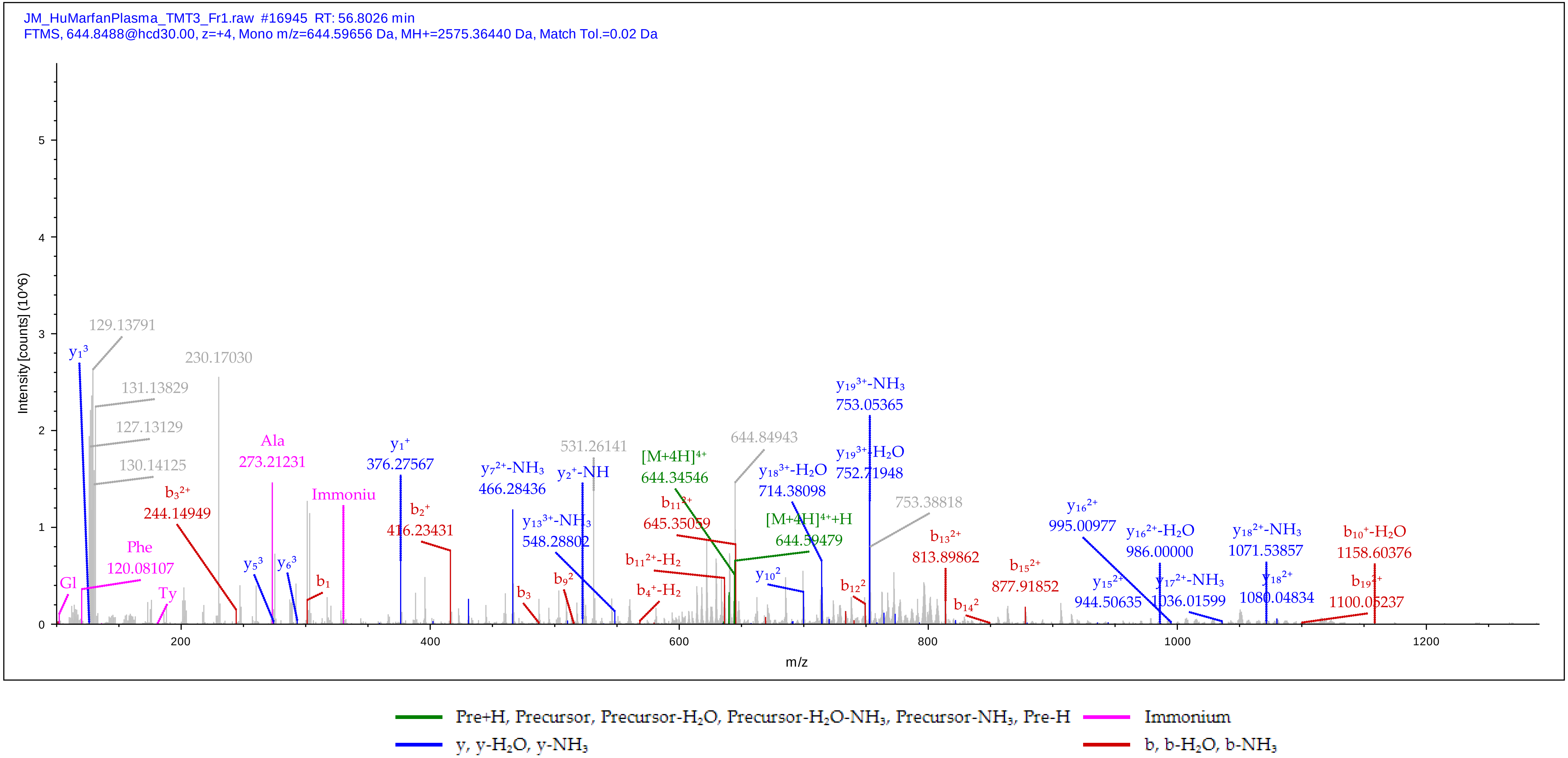
JM_HuMarfanPlasma_TMT3_Fr1.raw #16945 RT: 56.8026 min
FTMS, 644.8488@hcd30.00, z=+4, Mono m/z=644.59656 Da, MH+=2575.36440 Da, Match Tol.=0.02 Da
Intensity [counts] (10^6)
m/z
Pre+H, Precursor, Precursor-H2O, Precursor-H2O-NH3, Precursor-NH3, Pre-H
Immonium
y, y-H2O, y-NH3
b, b-H2O, b-NH3

#1	Immonium	b^{+}	b^{2+}	b^{3+}	b^{4+}	Seq.	y^{+}	y^{2+}	y^{3+}	y^{4+}	#2
1	331.21789	359.21280	180.11004	120.40912	90.55866	E-TMT6plex					29
2	72.08078	458.28122	229.64425	153.43192	115.32576	V	3321.48709	1661.24718	1107.83388	831.12723	28
3	72.08078	557.34963	279.17845	186.45473	140.09286	V	3222.41867	1611.71297	1074.81108	806.36013	27
4	60.04439	644.38166	322.69447	215.46540	161.85087	S	3123.35026	1562.17877	1041.78827	781.59302	26
5	86.09643	757.46572	379.23650	253.16009	190.12189	L	3036.31823	1518.66275	1012.77759	759.83501	25
6	74.06004	858.51340	429.76034	286.84265	215.38381	T	2923.23417	1462.12072	975.08291	731.56400	24
7	102.05496	987.55599	494.28163	329.85685	247.64446	E	2822.18649	1411.59688	941.40035	706.30208	23
8	44.04948	1058.59311	529.80019	353.53589	265.40373	A	2693.14389	1347.07559	898.38615	674.04143	22
9	133.04301	1218.62376	609.81552	406.87944	305.41140	C-Carbamidomethyl	2622.10678	1311.55703	874.70711	656.28215	21
10	133.04301	1378.65440	689.83084	460.22299	345.41906	C-Carbamidomethyl	2462.07613	1231.54170	821.36356	616.27449	20
11	44.04948	1449.69152	725.34940	483.90202	363.17834	A	2302.04548	1151.52638	768.02001	576.26683	19
12	102.05496	1578.73411	789.87069	526.91622	395.43899	E	2231.00837	1116.00782	744.34097	558.50755	18
13	30.03383	1635.75557	818.38143	545.92338	409.69435	G	2101.96578	1051.48653	701.32678	526.24690	17
14	44.04948	1706.79269	853.89998	569.60241	427.45363	A	2044.94431	1022.97580	682.31962	511.99154	16
15	88.03930	1821.81963	911.41345	607.94473	456.21037	D	1973.90720	987.45724	658.64058	494.23226	15
16	70.06513	1918.87239	959.93984	640.29565	480.47356	P	1858.88026	929.94377	620.29827	465.47552	14
17	88.03930	2033.89934	1017.45331	678.63796	509.23029	D	1761.82749	881.41738	587.94735	441.21233	13
18	133.04301	2193.92999	1097.46863	731.98151	549.23795	C-Carbamidomethyl	1646.80055	823.90391	549.60503	412.45559	12
19	181.06077	2401.97839	1201.49283	801.33098	601.25006	Y-Nitro	1486.76990	743.88859	496.26148	372.44793	11
20	88.03930	2517.00534	1259.00631	839.67330	630.00679	D	1278.72150	639.86439	426.91202	320.43583	10
21	74.06004	2618.05301	1309.53015	873.35586	655.26871	T	1163.69455	582.35091	388.56970	291.67910	9
22	129.11347	2774.15412	1387.58070	925.38956	694.29399	R	1062.64687	531.82708	354.88714	266.41718	8
23	74.06004	2875.20180	1438.10454	959.07212	719.55591	T	906.54576	453.77652	302.85344	227.39190	7
24	60.04439	2962.23383	1481.62055	988.08279	741.31392	S	805.49808	403.25268	269.17088	202.12998	6
25	44.04948	3033.27095	1517.13911	1011.76183	759.07319	A	718.46606	359.73667	240.16020	180.37197	5
26	86.09643	3146.35501	1573.68114	1049.45652	787.34421	L	647.42894	324.21811	216.48117	162.61269	4
27	60.04439	3233.38704	1617.19716	1078.46720	809.10222	S	534.34488	267.67608	178.78648	134.34168	3
28	44.04948	3304.42415	1652.71571	1102.14623	826.86150	A	447.31285	224.16006	149.77580	112.58367	2
29	330.27026					K-TMT6plex	376.27574	188.64151	126.09676	94.82439	1

Nitro-Tyr immonium ion is detected in MS/MS spectra and added brown colored in the following spectrum

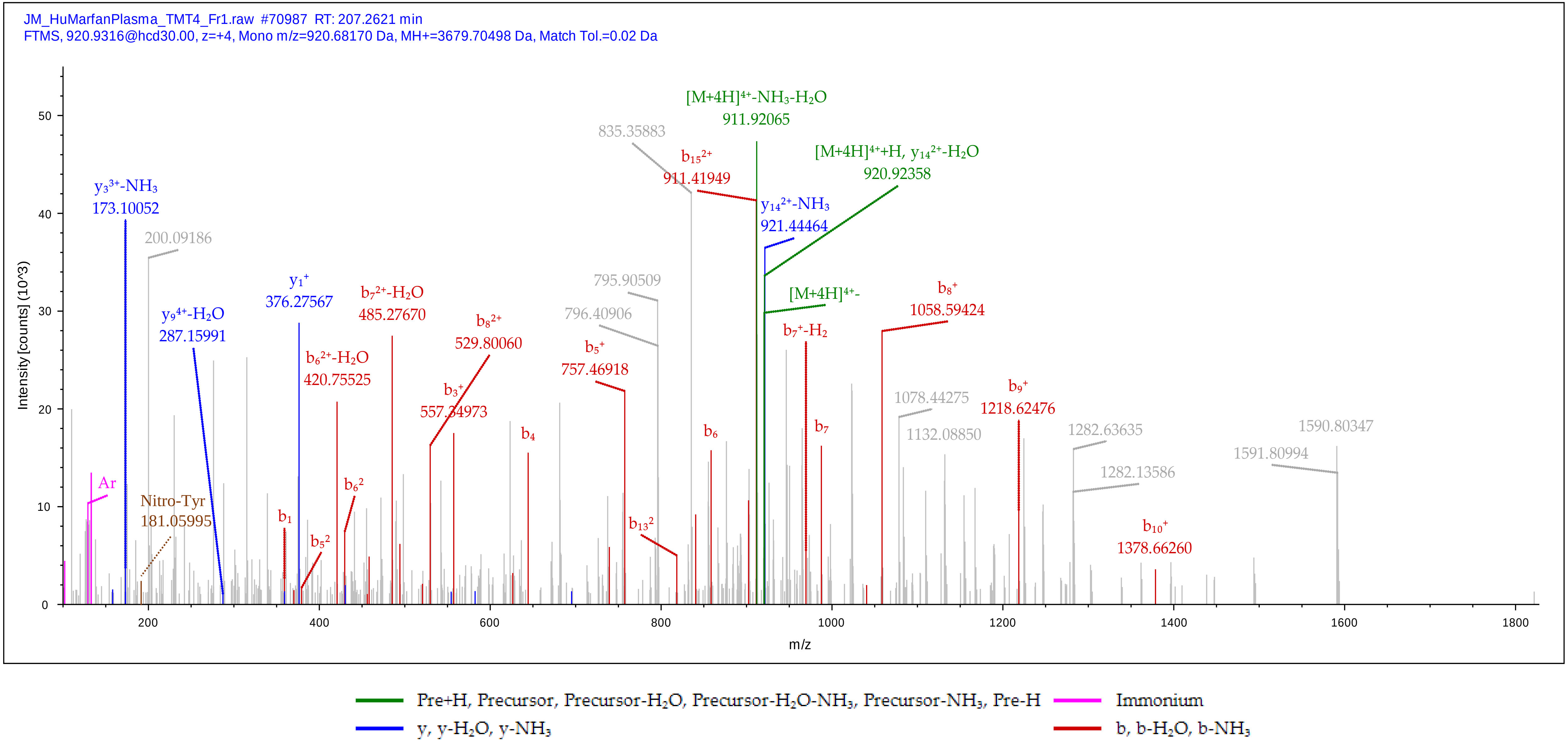

JM_HuMarfanPlasma_TMT4_Fr1.raw #70987 RT: 207.2621 min
FTMS, 920.9316@hcd30.00, z=+4, Mono m/z=920.68170 Da, MH+=3679.70498 Da, Match Tol.=0.02 Da
Intensity [counts] (10^3)
m/z
[M+4H]4+-NH3-H2O
911.92065
835.35883
b15 2+
911.41949
[M+4H]4++H, y14 2+-H2O
920.92358
y3 3+-NH3
173.10052
y14 2+-NH3
921.44464
200.09186
795.90509
[M+4H]4+-
b8+
1058.59424
y1+
376.27567
b7 2+-H2O
485.27670
y9 4+-H2O
287.15991
796.40906
b8 2+
529.80060
b7+-H2
b6 2+-H2O
420.75525
b5+
757.46918
b3+
557.34973
1078.44275
b9+
1218.62476
b7
b4
b6
1132.08850
1282.63635
1590.80347
1591.80994
1282.13586
Ar
Nitro-Tyr
181.05995
b6 2
b1
b13 2
b5 2
b10+
1378.66260
Pre+H, Precursor, Precursor-H2O, Precursor-H2O-NH3, Precursor-NH3, Pre-H
Immonium
y, y-H2O, y-NH3
b, b-H2O, b-NH3
