# Supplementary material for: Aortic disease in Marfan syndrome is caused by overactivation of sGC-PRKG signaling by NO
Source: Nat Commun. 2021 May 11;12:2628. doi: 10.1038/s41467-021-22933-3 (PMC8113458; doi:10.1038/s41467-021-22933-3)
Supplement: Supplementary file 9 — Reporting Summary [file 41467_2021_22933_MOESM9_ESM.pdf]

nature research

Corresponding author(s): Miguel R. Campanero and Juan Miguel Redondo

Last updated by author(s): Mar 8, 2021

Reporting Summary

Nature Research wishes to improve the reproducibility of the work that we publish. This form provides structure for consistency and transparency in reporting. For further information on Nature Research policies, see Authors & Referees and the Editorial Policy Checklist.

Statistics

For all statistical analyses, confirm that the following items are present in the figure legend, table legend, main text, or Methods section.

n/a	Confirmed	
☐	☒	The exact sample size (*n*) for each experimental group/condition, given as a discrete number and unit of measurement
☐	☒	A statement on whether measurements were taken from distinct samples or whether the same sample was measured repeatedly
☐	☒	The statistical test(s) used AND whether they are one- or two-sided<br>*Only common tests should be described solely by name; describe more complex techniques in the Methods section.*
☐	☒	A description of all covariates tested
☐	☒	A description of any assumptions or corrections, such as tests of normality and adjustment for multiple comparisons
☐	☒	A full description of the statistical parameters including central tendency (e.g. means) or other basic estimates (e.g. regression coefficient) AND variation (e.g. standard deviation) or associated estimates of uncertainty (e.g. confidence intervals)
☐	☒	For null hypothesis testing, the test statistic (e.g. *F*, *t*, *r*) with confidence intervals, effect sizes, degrees of freedom and *P* value noted<br>*Give P values as exact values whenever suitable.*
☒	☐	For Bayesian analysis, information on the choice of priors and Markov chain Monte Carlo settings
☐	☒	For hierarchical and complex designs, identification of the appropriate level for tests and full reporting of outcomes
☐	☒	Estimates of effect sizes (e.g. Cohen's *d*, Pearson's *r*), indicating how they were calculated

Our web collection on statistics for biologists contains articles on many of the points above.

Software and code

Policy information about availability of computer code

Data collection	Histological images were acquired using Leica Application Suite V3.5.0 acquisition software (Leica) or with a scanner NanoZoomer-2.ORS C110730 and NDP.view 2 V2.7.43 software.<br>Fluorescence or immunofluorescence images were acquired using LAS-AF V2.7.3. acquisition software (Leica).<br>Blood pressure measurements were acquired with a BP-2000 2011.11.15 analysis software (Visitech Systems).<br>Ultrasound data were collected using a Vevo 2100 1.5.0 software (Visual Sonics)<br>Flow cytometry data were collected using a Canto 3L HTS cytometer and the BD FACSDiva Software Version 6.1.3<br>qPCR data were acquired using the Bio-Rad CFX Manager 3.1 software.<br>Proteomics data were collected using either a Q-Exactive HF mass spectrometer (Termo Fisher Scientific) or an Orbitrap Fusion mass spectrometer (Thermo Fisher Scientific).
Data analysis	Images were analyzed and quantified using ImageJ V1.52a software. NDP.view 2 V2.7.43 software was used to visualize images from the scanner.<br>Flow cytometry data were analyzed using FlowJo 10.7.1 software.<br>Data from mass spectrometry were analyzed using Proteome Discoverer version 2.1 and SEQUEST HT (Thermo Fisher Scientific), DecoyPYrat and SanXot released version 2019 and limma 3.41.4 software package.<br>qPCR data were analyzed using the Bio-Rad CFX Manager 3.1 software and further analysis was performed in Excel software.<br>Statistics analysis was performed using GraphPad Prism 7.05 software.<br>Additional layout was made using Adobe Photoshop software.

For manuscripts utilizing custom algorithms or software that are central to the research but not yet described in published literature, software must be made available to editors/reviewers. We strongly encourage code deposition in a community repository (e.g. GitHub). See the Nature Research guidelines for submitting code & software for further information.

Data

Policy information about availability of data

All manuscripts must include a data availability statement. This statement should provide the following information, where applicable:
- Accession codes, unique identifiers, or web links for publicly available datasets
- A list of figures that have associated raw data
- A description of any restrictions on data availability

Source data are provided with this paper for all figures. The mass spectrometry raw data that support the findings of this study, including nitrated peptide MS/MS spectra (Supplementary Data 2 and 4), mouse reference proteome database December 2016 and human reference proteome database July 2018, are publicly available in the Peptide Atlas repository (http://www.peptideatlas.org/PASS/PASS01528). The following databases were used: UniProtKB, Swiss-Prot and TrEMBL (https://www.uniprot.org/uniprot/), Nucleotide database (https://www.ncbi.nlm.nih.gov/nucleotide/), Protein Data Bank, PDB (https://www.rcsb.org/). All mass spectrometry raw data obtained in this study, including nitrated peptide MS/MS spectra, have been deposited in the Peptide Atlas (http://www.peptideatlas.org/PASS/PASS01528). Other relevant datasets generated and/or analyzed during the current study are available from the corresponding authors on reasonable request.

Field-specific reporting

Please select the one below that is the best fit for your research. If you are not sure, read the appropriate sections before making your selection.

☒ Life sciences ☐ Behavioural & social sciences ☐ Ecological, evolutionary & environmental sciences

For a reference copy of the document with all sections, see nature.com/documents/nr-reporting-summary-flat.pdf

Life sciences study design

All studies must disclose on these points even when the disclosure is negative.

Sample size	The initial estimation of sample size was performed using software Gpower3.1.94. In brief, with a 0.8 experiment power, a significance level of 0.05, the possible mortality associated to a genotype or treatment and considering that differences around 1 SD would be informative when comparing strains and/or treatments, the estimated number of animals per condition would be 17 mice, pooled from independent experiments. After this inital estimation, the number of animals was reduced based on the results obtained from previous experiments. The specific numbers of animals used in each type of experiment are indicated in the corresponding figure legends.
Data exclusions	No data were excluded
Replication	All attempts on replication were successfull. All experiments were done with at least three biological replicates.
Randomization	No randomization was performed to allocate animals into experimental groups. Experimental groups were balanced in terms of sex, age, weight and Ascending/Abdominal basal aorta diameter criteria, so mice were allocated in experimental groups based on these criteria.
Blinding	All ex vivo experiments and proteomics analysis performed were blinded. In the case of in vivo experiments, it was not always possible to blind the experimenter to the identity of the animals, particularly during the Covid-19 pandemic. To decrease the possibility of introducing a bias in the analysis, each independent experiment was performed by a different investigator, when possible.

Reporting for specific materials, systems and methods

We require information from authors about some types of materials, experimental systems and methods used in many studies. Here, indicate whether each material, system or method listed is relevant to your study. If you are not sure if a list item applies to your research, read the appropriate section before selecting a response.

Materials & experimental systems

n/a	Involved in the study	
☐	☒	Antibodies
☐	☒	Eukaryotic cell lines
☒	☐	Palaeontology
☐	☒	Animals and other organisms
☐	☒	Human research participants
☒	☐	Clinical data

Methods

n/a	Involved in the study	
☒	☐	ChIP-seq
☐	☒	Flow cytometry
☒	☐	MRI-based neuroimaging

Antibodies

Antibodies used	-mouse monoclonal anti-pVASP (1:50; sc-101439, Santa Cruz Biotechnology, Santa Cruz, CA, USA)<br>-rabbit polyclonal anti-p-VASP (1:25; SAB4300129, Sigma-Aldrich, St. Louis, MO, USA)

	-rabbit polyclonal anti-PRKG (1:50 for immunofluorescence and 1:1000 for immunoblot, ADI-KAP-PK005-F, Enzo Life Sciences)<br>-rabbit polyclonal anti-GFP (1:100 for immunohistochemistry, A11122, Invitrogen; Carlsbad, CA, USA)<br>-pAb 9543 rabbit anti-fibrillin-1 polyclonal antibody (1:2000 for immunofluorescence)<br>-Texas Red-X-conjugated phalloidin (1:2000 for immunofluorescence, T7471,Thermo Fisher Scientific; Bremen, Germany)<br>-mouse monoclonal [6C5 clone] anti-Gapdh (1:10,000 for immunoblot; ab8245 Abcam, Cambridge, UK)<br>-rabbit polyclonal anti-GUCY1A3 (1:1000 for immunoblot, 12605-1-AP, Proteintech; Rosemont, IL, USA)<br>-rabbit polyclonal anti-GUCY1B3 GUCY1A3 (1:500 for immunoblot, 19011-1-AP, Proteintech; Rosemont, IL, USA)<br>-mouse monoclonal anti-α-Tubulin (1:40,000 for immunoblot, T 6074, Sigma-Aldrich; St. Louis, MO, USA)<br>-secondary antibodies for immunofluorescence were polyclonal Alexa-Fluor-647-conjugated goat anti-rabbit (1:500, A-21245, Molecular probes) or polyclonal Alexa-Fluor-647-conjugated chicken anti-mouse (1:500, A-21463, Molecular Probes).<br>-secondary antibodies for immunofluorescence were polyclonal AlexaFluor568-conjugated goat anti-mouse (1:500, A-11031, Molecular Probes)
Validation	All antibodies used were validated in the manufacturer's website for our species and application. Specificity was determined by substituting the primary antibody with unrelated IgG at the same dilutions as the antigen-specific antibodies.<br>-mouse monoclonal anti-pVASP (1:50; sc-101439, Santa Cruz Biotechnology, Santa Cruz, CA, USA). From manufactures website: 'p-VASP (16C2) is recommended for detection of Ser 239 phosphorylatedVASP of mouse, rat and human origin by Western Blotting (starting dilution1:200, dilution range 1:100-1:1000), immunoprecipitation [1-2 µg per 100-500 µg of total protein (1 ml of cell lysate)], immunofluorescence (startingdilution 1:50, dilution range 1:50-1:500), flow cytometry (1 µg per 1 x 106cells) and solid phase ELISA (starting dilution 1:30, dilution range 1:30-1:3000).'<br>-rabbit polyclonal anti-p-VASP (1:25; SAB4300129, Sigma-Aldrich, St. Louis, MO, USA). From manufacturer website: 'application(s): immunohistochemistry (formalin-fixed, paraffin-embedded sections): 1:50-1:100; western blot: 1:500-1:1000. Species reactivity: human, mouse, rat.'<br>-rabbit polyclonal anti-PRKG (1:50 for immunofluorescence and 1:1000 for immunoblot, ADI-KAP-PK005-F, Enzo Life Sciences). From manufacturer website: 'Species reactivity: Human, Mouse, Rat, Porcine. Applications: IHC (PS), WB'. We tested its specificity in immunoflurescence by substituting the primary antibody with unrelated IgG at the same dilutions as the antigen-specific antibodies.<br>-rabbit polyclonal anti-GFP (1:100 for immunohistochemistry, A11122, Invitrogen; Carlsbad, CA, USA). From manufacturer website: 'Published species: Fruit fly, Human, Mouse, Rat, Sheep, Tag, Zebrafish. Immunohistochemistry (IHC) 1:200-1:2000'. In this case, we used it 1:100 after a trial with different dilutions and testing its specificity by substituting the primary antibody with unrelated IgG at the same dilutions as the antigen-specific antibodies.<br>-pAb 9543 rabbit anti-fibrillin-1 polyclonal antibody (1:2000 for immunofluorescence). This antibody was kindly donated by Dr. L. Sakai, so we used the same conditions as they use in their laboratory.<br>-Texas Red-X-conjugated phalloidin (1:2000 for immunofluorescence, T7471,Thermo Fisher Scientific; Bremen, Germany). From manufacturer website: 'Selectively stains F-actin, outstanding fluorescence performance, superior to antibody staining, optimal for fixed and permeabilized samples.' Dilution was empirically determined after a trial.<br>-mouse monoclonal [6C5 clone] anti-Gapdh (1:10,000 for immunoblot; ab8245 Abcam, Cambridge, UK). From manufacturer website: 'Suitable for: WB, ICC/IF. Reacts with: Mouse, Rat, Human. WB use: 1:500-1:10000).'<br>-rabbit polyclonal anti-GUCY1A3 (1:1000 for immunoblot, 12605-1-AP, Proteintech; Rosemont, IL, USA). from manufacturer website: 'Reactivity: human, mouse, rat. Applications: WB, IP, IHC, ELISA. WB : 1:500-1:2000'.<br>-rabbit polyclonal anti-GUCY1B3 GUCY1A3 (1:500 for immunoblot, 19011-1-AP, Proteintech; Rosemont, IL, USA). From manufacturer website: 'Reactivity: human, mouse, rat. Applications: WB, IP, IHC, IF,ELISA. WB : 1:1000-1:4000'.<br>-mouse monoclonal anti-α-Tubulin (1:40,000 for immunoblot, T 6074, Sigma-Aldrich; St. Louis, MO, USA). From manufacturer website: 'Species reactivity: chicken, kangaroo rat, sea urchin, rat, Chlamydomonas, bovine, human, African green monkey, mouse. Application(s): western blot: 0.25-0.5 µg/mL using total cell extract of human foreskin fibroblast cell line (FS11).'

Eukaryotic cell lines

Policy information about cell lines

Cell line source(s)	Primary mouse vascular smooth muscle cells (VSMCs) were isolated and grown as described. All experiments were performed using cells from passages 3–7. HEK-293T (CRL-1573) and Jurkat (Clone E6-1, TIB-152) cell lines used in this study were used only after receipt from ATCC or after resucitation from early stocks at low passage number.
Authentication	HEK293T and Jurkat were not authenticated. Instead, cells were used after receipt or after resucitation from early stocks at low passage number.
Mycoplasma contamination	All cells were mycoplasma-negative
Commonly misidentified lines<br>(See ICLAC register)	HEK293 is one of the cell lines misidentified when no authentic stock is known. However, the HEK293T cell line used in this study was obtained from ATCC or after resucitation from early stocks at low passage number. HEK-293T were used in this study for lentiviral production because it is the established cell line for this purpose.

Animals and other organisms

Policy information about studies involving animals; ARRIVE guidelines recommended for reporting animal research

Laboratory animals	Fbn1C1039G/+ mice were obtained from Jackson Laboratories (JAX mice stock #012885). This strain had been previously backcrossed to the C57BL/6 background for more than nine generations. Wild-type (WT) mice were on the C57BL/6 background. 12–15-week-old males and females were used for all experiments. Mice were housed in a pathogen-free animal facility under a 12 h light/dark cycle at constant temperature and humidity, and fed standard rodent chow and water ad libitum.

Wild animals	No wild animals were used in the study.
Field-collected samples	No field collected samples were used in the study.
Ethics oversight	Animal procedures were approved by the CNIC Ethics Committee and the Madrid regional authorities (ref. PROEX 80/16) and conformed to EU Directive 2010/63EU and Recommendation 2007/526/EC regarding the protection of animals used for experimental and other scientific purposes, enforced in Spanish law under Real Decreto 1201/2005.

Note that full information on the approval of the study protocol must also be provided in the manuscript.

Human research participants

Policy information about studies involving human research participants

Population characteristics	The covariate-relevant population characteristics of the research participants from which samples were obtained, are:<br>Age: 18-70 years<br>Sex: both males and females were included in these studies<br>Genotipic information and diagnosis: these studies were conformed by healthy donors and Marfan patients. In the later case, patients were either phenotipically diagnosed or genetically genotyped as Marfan Syndrome Patients.
Recruitment	Aorta, plasma and serum samples from patients with Marfan Syndrome were obtained from biobanks of Hospital Puerta de Hierro, Hospital Vall D'Hebron, and Ghent University Hospital. Age- and sex-matched ascending aorta samples used as controls were obtained anonymously from multiorgan transplant donors after written informed consent was obtained from their families, and age- and sex-matched control plasma samples were obtained from healthy volunteers. No potential selection biases that could impact the results were present.
Ethics oversight	The study complied with all relevant ethical regulations and was approved by the Research Ethics Committee of Cantabria (ref. 27/2013), the Ethics Committee of Ghent University Hospital (B65020111160), and the Ethics Committee of the Instituto de Salud Carlos III (CEI PI91¬_2018-v2-Enmienda_2019). Informed consent was obtained from all human participants or their families. Patient clinical data were retrieved while maintaining anonymity.

Note that full information on the approval of the study protocol must also be provided in the manuscript.

Flow Cytometry

Plots

Confirm that:

- [ ] The axis labels state the marker and fluorochrome used (e.g. CD4-FITC).
- [x] The axis scales are clearly visible. Include numbers along axes only for bottom left plot of group (a 'group' is an analysis of identical markers).
- [ ] All plots are contour plots with outliers or pseudocolor plots.
- [ ] A numerical value for number of cells or percentage (with statistics) is provided.

Methodology

Sample preparation	Jurkat cells were used for lentiviral titration. Jurkat cells were plated in a 96-well plate and infected with lentivirus at various dilutions (1:10-1:100000). After 48 hours, cells were centrifuged 5 minutes at 1800g and resuspended in 200microL of ice-cold PBS with 1:1000 propidium iodide.
Instrument	Flow cytometry data were collected using a Canto 3L HTS cytometer and the BD FACSDiva Software Version 6.1.3
Software	Flow cytometry data were collected using the BD FACSDiva Software Version 6.1.3 and analyzed using FlowJo 10.7.1 software.
Cell population abundance	All sorted cells were Jurkat cells. The percentage of infected Jurkat varies from 99% to 2% depending on the lentiviral dilution (1:10-1:100000).
Gating strategy	The gating strategy is illustrated in the Supplementary Figure 14. An initial FSC-A and SSC-A density plot was used to identify Jurkat cell population. This population was gated in a FSC-H vs FSC-A plot to select single cells and exclude doublet cells. Then, alive cells (Propidium Iodide negative cells) were identified with a PI (FI3)-A) histogram. Finally, the alive Jurkat population was gated for GFP-A in a histogram. The threshold for the histogram was determined using non-infected Jurkat cells as negative control. To determine the percentage of infected cells (GFP+), these gates were applied to all the samples acquired.

- [x] Tick this box to confirm that a figure exemplifying the gating strategy is provided in the Supplementary Information.
